# Supplementary material for: Effect of a brief motivational interview and text message intervention targeting tobacco smoking, alcohol use and medication adherence to improve tuberculosis treatment outcomes in adult patients with tuberculosis: a multicentre, randomised controlled trial of the ProLife programme in South Africa
Source: BMJ Open. 2022 Feb 14;12(2):e056496. doi: 10.1136/bmjopen-2021-056496 (PMC8845202; doi:10.1136/bmjopen-2021-056496)
Supplement: Supplementary data [file bmjopen-2021-056496supp001.pdf]

SUPPLEMENTARY TABLES

Supplementary table 1 Detailed baseline descriptive statistics for socio-demographic, socio-economic, and clinical characteristics by study arm

	Control (N = 291) n (%)*	Intervention (N = 283) n (%)*	Total (N=574) n (%)*
**Marital status**			
Married or living together	102 (35.1)	95 (33.6)	197 (34.3)
Divorced/separated	20 (6.9)	18 (6.4)	38 (6.6)
Widowed	10 (3.4)	7 (2.5)	17 (3.0)
Never married and never lived together	144 (49.5)	150 (53.0)	294 (51.2)
Declined to answer	15 (5.2)	13 (4.6)	28 (4.9)
**Employment**			
Self-employed (full-time)	30 (10.3)	36 (12.7)	66 (11.5)
Employed full-time (30 hrs a week or more)	62 (21.3)	54 (19.1)	116 (20.2)
Employed part-time (less than 30 hrs a week)	19 (6.5)	29 (10.2)	48 (8.4)
Retired	17 (5.8)	16 (5.7)	33 (5.7)
Unemployed (but able to work)	125 (43.0)	120 (42.4)	245 (42.7)
Unable to work because of long-term disability or ill health	9 (3.1)	8 (2.8)	17 (3.0)
Full-time student	12 (4.1)	4 (1.4)	16 (2.8)
Caring from my home and family/doing household work/housewife	0 (0.0)	2 (0.7)	2 (0.3)
Occasional work ("piece job")	17 (5.8)	12 (4.2)	29 (5.1)
Declined to answer	0 (0.0)	2 (0.7)	2 (0.3)
**Ever worked or spent time in mines**			
No	244 (83.8)	237 (83.7)	481 (83.8)
Yes	46 (15.8)	45 (15.9)	91 (15.9)
**Household items (Yes is displayed)**			
A radio	249 (85.6)	234 (82.7)	483 (84.1)
A television	255 (87.6)	255 (90.1)	510 (88.9)
A landline telephone	21 (7.2)	22 (7.8)	43 (7.5)
A desktop or laptop computer	67 (23.0)	48 (17.0)	115 (20.0)
A refrigerator	248 (85.2)	240 (84.8)	488 (85.0)
A vacuum cleaner or floor pol	49 (16.8)	35 (12.4)	84 (14.6)
A microwave oven	198 (68.0)	189 (66.8)	387 (67.4)
An electric or gas stove	254 (87.3)	238 (84.1)	492 (85.7)
A washing machine	153 (52.6)	136 (48.1)	289 (50.3)
Total number of assets: mean (SD)	5.14 (1.96)	4.94 (1.77)	5.04 (1.87)
**In the past month, number of days you or people in the household went to bed hungry because there was no food to eat**			
0 days	244 (83.8)	238 (84.1)	482 (84.0)
1-7 days	45 (15.5)	34 (12.0)	79 (13.8)
More than 7 days	2 (0.7)	9 (3.2)	11 (1.9)
Declined to answer	0 (0.0)	2 (0.7)	2 (0.3)
**Number of pre-treatment smear tests recorded?**			

One	197 (92.1)	169 (87.6)	366 (89.9)
Two	17 (7.9)	24 (12.4)	41 (10.1)
**Number of Gene XPert results recorded**			
One	225 (97.8)	210 (97.2)	435 (97.5)
Two	5 (2.2)	6 (2.8)	11 (2.5)
**Number of culture results recorded on the TB Treatment record**			
One	54 (94.7)	42 (97.7)	96 (96.0)
Two	3 (5.3)	1 (2.3)	4 (4.0)
**Co-morbidities**			
Hypertension	19 (6.93)	11 (4.1)	30 (5.54)
Diabetes	5 (1.84)	4 (1.49)	9 (1.66)
Epilepsy	3 (1.09)	4 (1.49)	7 (1.29)
Mental illness	3 (1.09)	0 (0)	3 (0.55)
Liver disease	1 (0.36)	1 (0.38)	2 (0.37)
Renal insufficiency	1 (0.36)	1 (0.38)	2 (0.37)
Allergies	2 (0.76)	0 (0)	2 (0.38)
Other	1 (0.36)	1 (0.38)	2 (0.37)
**Depression score (CESD 10): mean (SD)**	8.44 (4.38)	8.74 (4.8)	8.59 (4.59)

*Counts and percentages unless otherwise indicated

Supplementary table 2 Detailed descriptive statistics for smoking history, alcohol history at baseline by study arm

	Control<br>n (%)*	Intervention<br>n (%)*	Total<br>n (%)*
Smoking History (current smokers only) N=	181	191	372
**On the days that you smoke, how soon after you wake up do you have your first cigarette?**			
After 60 minutes	30 (16.6)	28 (14.7)	58 (15.6)
31-60 minutes	24 (13.3)	16 (8.4)	40 (10.8)
6- 30 minutes	60 (33.1)	65 (34.0)	125 (33.6)
Within 5 minutes	67 (37.0)	82 (42.9)	149 (40.1)
**Duration of smoking in months: mean (SD)**	212.09 (134.03)	224.93 (127.82)	218.68 (130.86)
**Duration of smoking in months: median (IQR)**	186 (110, 282)	206 (135, 294)	200.5 (123, 287)
**Age started smoking in years: mean (SD)**	19.2 (6.3)	19.3 (6.3)	19.3 (6.3)
**Age started smoking in years: median (IQR)**	18 (15-20)	18 (16-21)	18 (15.5-20.5)
**Form of tobacco used**			
**Manufactured cigarettes (Yes)**	166 (91.7)	179 (93.7)	345 (92.7)
Number of days you smoked in the past 7days: mean (SD)	5.3 (2.65)	5.53 (2.33)	5.42 (2.49)
Average number of cigarettes smoked daily: mean (SD)	6.18 (6.43)	6.48 (8.21)	6.34 (7.39)
**Hand-rolled cigarettes (Yes)**	14 (7.7)	21 (11.0)	35 (9.4)
Number of days you smoked in the past 7days: mean (SD)	4.71 (2.84)	3.71 (2.95)	4.11 (2.91)
Average number of handrolled cigarettes smoked daily: mean (SD)	3.71 (3.97)	3.81 (2.82)	3.77 (3.27)
**Pipe (Yes)**	4 (2.2)	2 (1.0)	6 (1.6)
Number of days in the past 7days you smoked: median (IQR)	1 (0, 2.5)	4.5 (2, 7)	2 (0, 3)
Average number of daily sessions: median (IQR)	1 (0, 3.5)	3 (1, 5)	1.5 (0, 5)
Length of one session (on average) in **minutes**: median (IQR)	90 (60, 107.5)	60 (30, 90)	90 (30, 90)
**Cigars, cheroots or cigarillos (Yes)**	1 (0.55)	0 (0)	1(0.27)
**Water pipe (Yes)**	3 (1.7)	2 (1.0)	5 (1.3)
**Other**	10 (5.5)	4 (2.1)	14 (3.8)
**Heaviness of smoking index >= 4**	134(74.03)	158(82.72)	292(78.49)
Smoking inside your home restrictions			
Total: Not allowed	96 (53.0)	108 (56.5)	204 (54.8)
Some rules: where/when it is allowed	61 (33.7)	58 (30.4)	119 (32.0)
No rules	24 (13.3)	25 (13.1)	49 (13.2)

Attempts to quit smoking (current smokers only)			
**Ever attempted to quit in the past (Yes)**	52 (28.7)	64 (33.5)	116 (31.2)
Number of attempts to quit: mean (SD)	2.46 (2.98)	2.64 (1.62)	2.56 (2.32)
Time elapsed since attempt to quit last time, in months: mean (SD)	36.65 (94.62)	25.13 (37.91)	30.29 (69.22)
Longest duration abstinent in previous quit attempts: mean (SD)	6.15 (13.48)	4.22 (8.9)	5.09 (11.18)
**Likelihood to TRY TO QUIT smoking completely and permanently in the next three months**			
Definitely will not	6 (3.3)	5 (2.6)	11 (3.0)
Probably will not	10 (5.5)	12 (6.3)	22 (5.9)
Probably will	104 (57.5)	109 (57.1)	213 (57.3)
Definitely will	61 (33.7)	65 (34.0)	126 (33.9)
**Likelihood that I WILL QUIT smoking completely and permanently in the next three months**			
Definitely will not	6 (3.3)	5 (2.6)	11 (3.0)
Probably will not	11 (6.1)	13 (6.8)	24 (6.5)
Probably will	103 (56.9)	104 (54.5)	207 (55.6)
Definitely will	61 (33.7)	69 (36.1)	130 (34.9)
**Ever used any methods to help you stop smoking tobacco in the past 3-months?** (Yes)	23 (12.7)	16 (8.4)	39 (10.5)
Smokeless tobacco use (all participants)			
**In the past month, have you used smokeless tobacco (Snuff) on a daily basis**			
Not at all	275 (94.5)	275 (97.2)	550 (95.8)
Daily	12 (4.1)	6 (2.1)	18 (3.1)
Less than Daily	4 (1.4)	2 (0.7)	6 (1.0)
**Duration of using ST in months: mean(SD)**	113.69 (112.7)	152 (91.16)	126.46 (105.61)
**Age started using ST in years: mean (SD)**	27.56 (10.57)	25.88 (13.43)	27 (11.33)
**Form of ST used (for SLT users)**			
Snuff (by mouth)	2 (12.5)	0 (0.0)	2 (8.3)
Snuff (by nose)	11 (68.8)	5 (62.5)	16 (66.7)
Chewing tobacco leaves	0(0)	0(0)	0(0)
Other	1 (6.3)	0 (0.0)	1 (4.2)
**Help to stop drinking (N= , had a drink in past 12 months)**	208	223	431
**Ever used any methods to stop drinking alcohol in the past 3-months**	21 (11.2)	22 (10.7)	43 (10.9)

* Frequencies and (percentages) are presented unless otherwise stated

Supplementary table 3 Detailed descriptive statistics for primary outcome by study arm at 6-months

TB treatment status detailed	Control<br>n (%)	Intervention<br>n (%)	Total<br>n (%)
Cured	108 (37.1)	105 (37.1)	213 (37.1)
Treatment completed	96 (33.0)	87 (30.7)	183 (31.9)
Treatment interrupted > 2 months	15 (5.2)	29 (10.2)	44 (7.7)
Treatment failure	5 (1.7)	2 (0.7)	7 (1.2)
Acquired drug resistance	1 (0.3)	4 (1.4)	5 (0.87)
Died	11 (3.8)	15 (5.3)	26 (4.5)
Transfer out	8 (2.75)	11 (3.9)	19 (3.31)
Unknown	42 (14.4)	25 (8.8)	67 (11.7)

**Supplementary table 4** MI treatment fidelity scores of counselling sessions delivered by 17 lay health workers*		
**Ratings**	Mean (SD)	Range
**Global ratings****		
**Cultivating change talk**	3.2 (1.17)	1-5
**Softening sustain talk**	3.4 (0.96)	1-4
**Partnership**	3.4 (1.12)	2-5
**Empathy**	2.9 (1.34)	1-5
**Behaviour counts**[&]		
**Giving information**	4.8 (8.13)	0-5
**Persuade**	0.8 (1.48)	0-5
**Persuade with permission**	0.1 (0.24)	0-1
**Question**	24.2 (10.42)	12-51
**Simple reflection**	4.1 (3.21)	0-10
**Complex reflection**	1.6 (2.69)	0-9
**Affirm**	5.5 (3.47)	1-12
**Seeking collaboration**	2.4 (1.46)	0-6
**Emphasising autonomy**	1.4 (1.37)	0-4
**Confront**	0.4 (0.74)	0-3
**Summary measures**		
**Total MI non-adherent †**	1.2 (2.28)	0-7
**Total MI adherent†**	9.3 (4.74)	1-16
**Technical global ratings****	3.3 (0.97)	1.5-4.5
**Relational global ratings****	3.1 (1.19)	1.5-5
**Reflection to question ratio††**	0.23 (0.24)	0-0.83
**Percentage of complex reflections*****	20.4 (21.9)	0-67

*The recordings of 17 counsellors (one each) were transcribed verbatim and then assessed. In order to assess the fidelity of the counsellors' delivery of motivational interviewing during the trial, we are used the validated Motivational Interviewing Treatment Integrity Coding Manual 4.2.1 (MITI) tool. The table shows the results of the fidelity assessment that was conducted by one rater who is proficient in English, seSotho and seTswana and in motivational interviewing. The rater listened to the recordings and coded a randomly selected 20-min portion of the written transcript. In the case of shorter counselling sessions, the entire recording was assessed.

**The "global ratings" involve assessing, on a 5-point Likert scale (1 for low and 5 for high), how well or poorly the counsellor adheres to the MI practice. Ratings are conducted on four items, two each making up Technical Components (Cultivating change talk and Softening sustain talk) and Relational Components (Partnership and Empathy).

**Scores on Cultivating Change Talk and Softening Sustain Talk are averaged to obtain the Technical global scores.

***Scores on Partnership and Empathy are averaged to obtain Relational global scores. The basic competency threshold scores for fair and good proficiency are 3 and 4, respectively for Technical scores and 3.5 and 4, respectively for Relational scores.

[&]The "behaviour counts" involve counting 10 verbal behaviours of the counsellor during the intervention.

† "MI adherence" is determined by adding up the following verbal behaviours: Seeking Collaboration, Affirm and Emphasising autonomy. "MI non-adherence" is determined by summing instances of Confront and Persuade. No thresholds for MI adherence or non-adherence are specified in the MITI 4.2.1.

††The Reflection-to-Question (R:Q) ratio is the total reflections divided by the total questions asked. One reflection to each question is considered a "fair" practice level while two reflections to each question is considered a "good" practice level.

*** The Percentage of Complex Reflections (% CRs) is calculated by dividing the number of complex reflections by the sum of complex reflections and simple reflections. A fair and a good % CRs are 40% and 50%, respectively.

Supplementary table 5 SMS delivery

	Intervention (N=283)		Control (N=291)	
No. of participants who received ALL due IMB messages INDEPENDENT OF WHETHER they completed MI 1	99/283 (35%)		1/291 (0.3%)	
**Completion of first MI and initiation of SMS-sequence**				
Completed first MI	227/283 (80.2%)		0/291 (0%)	
No. of participants who received ALL due messages after receipt of MI1	95/227 (41.9%)		0/0 (0%)	
**SMS delivery for participants for whom the SMS-sequence was initiated (after receipt of first MI)**				
	Mean (SD)	Median (IQR)<br>Range	Mean (SD)	Median (IQR)<br>Range
Average no. adherence messages received per participant (n=227)	7.9 (3.5)	10 (8-10)<br>0-11	0	0
Average no. tobacco-related messages received (n=153)	5.4 (2.5)	7 (5-7)<br>0-7	0	0
Average no. alcohol related messages received (n=171)	5.5 (2.5)	7 (5-7)<br>0-7	0	0
Average no. IMB messages received (n= 227)	15.7 (7.3)	17 (13-22)<br>0-25		

Louwagie G, *et al. BMJ Open* 2022; 12:e056496. doi: 10.1136/bmjopen-2021-056496

Supplementary table 6 Unit costs used in the analysis (Presented in Rand as sources, $1=R14.448 in 2019)

Item	Unit costs
**Personnel**	LHWs: R3000/month, 160 hours/month, R18.75/hour; District coordinators: R7500/month, 160 hours/month, R46.88/hour; MRC appointed supervisor: R205.79/hour; Trainers: R373.17/hour; Administrative staff: R333.24/hour
**Materials**	Training manual: R3250; Printing: R3000; Additional printing & stationary: R500
**Accommodation / Travel / Refreshments**	LHWs accommodation: R540 000; Trainers accommodation/travel: R36 000; Supervision accommodation/travel: R30 000; Refreshment: R12 500
**SMS system**	Monthly subscription at R433.44 ($30) for 3-months: R1300
**MI sessions**	Session 1 (17 minutes): R5.31; Session 2 (16 minutes): R5.00; Session 3 (17 minutes): R5.31
**Biochemical investigations**	Cost of TB smear microscopy: R28.37; culture: R79.22 and GeneXpert: R201.56 (Source: 2019/20 National Health Laboratory Service pricing schedule)*
**ART**	Atroiza: R3.78/dose; Dumiva: R5.55/dose; Tenemine: R2.34/dose; Zovilam: R1.72/dose; Kavimun: R1.89/dose; Ricovir: R1.36/dose; Zidomat: R1.38/dose; Lazena: R0.55/dose; Efrin:R0.63/dose; Efamat: R0.63/dose; Acriptaz: R0.61/dose (Source: Western Cape Department of Health. Antiretroviral and TB Stockmaster Worksheet. 2019)
**TB medication**	Month's supply of RHZE (intensive) @ R65.80; RH (continuation) @ R55.56 (Source: Western Cape Department of Health. Antiretroviral and TB Stockmaster Worksheet. 2019)
