# Supplementary material for: Effect of a brief motivational interview and text message intervention targeting tobacco smoking, alcohol use and medication adherence to improve tuberculosis treatment outcomes in adult patients with tuberculosis: a multicentre, randomised controlled trial of the ProLife programme in South Africa
Source: BMJ Open. 2022 Feb 14;12(2):e056496. doi: 10.1136/bmjopen-2021-056496 (PMC8845202; doi:10.1136/bmjopen-2021-056496)

PROLIFE

Im*pro*ving TB outcomes by modifying *life*-style behaviours through a brief motivational intervention followed by short text messages (Phase II)

A multi-centre randomised controlled trial looking at the effect of a complex behavioural intervention on TB and lifestyle-related outcomes in South Africa

Version 1.2

Trial Registration number/registry: ISRCTN62728852/ISRCTN registry http://www.isrctn.com/ISRCTN62728852

Version date: ~~**14 Feb 2020**~~ 25 Oct 2020

Author(s): Mona Kanaan

Department of Health Sciences
University of York
York, YO10 5DD

Principal Investigator: Olalekan Ayo-Yusuf and Kamran Siddiqi
Trial Coordinator: Goedele M Louwagie

**Sponsor**	**Olalekan Ayo-Yusuf, Sefako Makgatho University/ University of Pretoria**
**Funder**	**Newton Fund/MRC**
**Funder Reference Number**	**MRC-RFA-02: TB -05-2015**

Louwagie G, *et al. BMJ Open* 2022; 12:e056496. doi: 10.1136/bmjopen-2021-056496

STATISTICAL ANALYSIS PLAN
V1.2

Contents

1. Definition of terms 4
2. Trial Objectives 5
3. Design 6
4. Sample Size 7
5. Randomisation 7
6. Outcomes 8
6.1 Primary outcome 8
6.2 Secondary outcomes 9
6.2.1 Monitoring adverse events 9
6.3 Trial assessment schedule 11
6.4 Other important information 12
6.5 Fidelity of the intervention 12
7. Data 13
7.1 Data collection methods 13
7.2 Data management 16
8. Analysis 17
8.1 Participants Flow 18
8.2 Baseline data 19
8.3 Primary analysis 24
8.3.1 Primary outcome definition: 24
8.3.2 Primary outcome Analysis 24
8.3.3 Primary outcome Analysis Results 25
8.4 Secondary analyses 26
8.4.1 Secondary outcome definitions that involve defining a positive baseline test: 27
8.4.2 Continuous Abstinence 29
8.4.4 Change in harmful or hazardous drinking at month 3 and 6 follow-ups 32
8.4.5 Proportion of ART uptake of HIV-positive participants at month 3 and 6 follow-ups . 34
8.5 Subgroup analyses (See above analyses) 35
8.5 Sensitivity analyses 35
8.6 Adverse events 36
8.7 Planned interim review and analyses 36

8.8 List of Tables and Graphs 37
9. SAP amendment log 38
10. Signatures of approval 38
11. References 39
12. Appendix A (Results tables) 40
Table 1: 40
13. Appendix B (Scoring notes +) ..... **Error! Bookmark not defined.**
13. Additional descriptive Statistics 65

This analysis plan deals only with the statistical analysis of the trial.

Louwagie G, *et al. BMJ Open* 2022; 12:e056496. doi: 10.1136/bmjopen-2021-056496

1. Definition of terms

Abbreviation	Expansion
ACTG	AIDS Clinical Trials Group
AE	Adverse Event
ART	Anti-retroviral therapy
AUDIT	Alcohol Use Disorders Identification Test
CI	Confidence Interval
CO	Carbon monoxide
CONSORT	Consolidated Standards of Reporting Trials
CRF	Case Report Form
EPTB	Extra-pulmonary TB
HIV	Human immunodeficiency virus
LHW	Lay health worker
MI	Motivational interviewing
PTB	Pulmonary TB
SMS	Short Message Service
SA	South Africa
SADHS	South Africa Demographic and Health Survey
SAE	Serious Adverse Event
SD	Standard deviation
SOPs	Standard Operating Procedures
TB	Tuberculosis

2. Trial Objectives

The PROLIFE model is a complex behavioural intervention comprised of a brief motivational interviewing (MI) counselling strategy augmented with subsequent SMS messaging. To be delivered in three brief sessions, the MI intervention will target three main areas, as appropriate:

- Tobacco smoking
- Alcohol drinking
- Tuberculosis (TB) and Anti-retroviral therapy (ART) adherence or ART initiation

Primary objective:

- To assess the effectiveness of the PROLIFE model delivered by lay health workers (LWH) compared to usual care in improving Pulmonary TB (PTB) treatment outcomes

Secondary objective: (**this element will not be addressed in this SAP**)

- To estimate the cost-effectiveness of the PROLIFE model

3. Design

This is a pragmatic, prospective, multicentre, two-arm, parallel, individual RCT taking place in 27 purposively selected primary care clinics with the highest TB case-load in three districts in South Africa: Welkom in the Free State; Bojanala in the North West province; and Sedibeng in Gauteng province. The intervention will be delivered by LHWs and three district coordinators who will each cover 1–2 clinics.

This is a pragmatic parallel superiority individually randomised controlled trial. There are two treatment arms:

<u>The control arm (Arm1):</u> Intervention arm – participants will receive the PROLIFE programme;

<u>The intervention arm (Arm2):</u> Control arm – participants will receive usual treatment and support provided to TB patients in TB treatment clinics in South Africa ('usual care').

Full details of the background and design of the trial are presented in the protocol (version 1.2 Prolife Protocol_15 Dec 17_with markup) and the published protocol in Moriarty et al (2019) https://doi.org/10.1186/s13063-019-3551-9.

Participants

The inclusion criteria for participants are:

- adult patients (aged ≥ 18 years)
    - with drug-sensitive (bacteriologically or clinically confirmed) PTB;
- initiating TB treatment or on TB treatment for < 1 month (these include both 'new' and 'retreatment' patients);
- current smokers and/or
- hazardous/harmful drinkers who are not alcohol dependent (Alcohol Use Disorders Identification Test [AUDIT] score ≥ 8 for men or ≥ 7 for women but < 20);
- access to a functional mobile phone; and
- understand one of the four languages used for the trial (Sesotho, Setswana, Isizulu or English).

Exclusion criteria:

- alcohol-dependent participants (AUDIT score ≥ 20);
- Extrapulmonary TB without PTB; or
- Resistance to one or more TB drugs at baseline

4. Sample Size

We will recruit 696 participants (348 per study arm). The sample size calculations were based on the following assumptions:

- Detection a 10% difference in TB treatment success rates (0.86 vs 0.76) in the ProLife group versus the control group
- 80% power,
- a significance level of 0.05, and
- 25% attrition.

The sample size per clinic was in the range of 14–74 participants per clinic with a median of 24. The assumed success rates in the control group are based on actual success rates in TB patients in the studied provinces that were available at the time of sample size calculations in 2015.

5. Randomisation

Patients will be randomised using a randomised sequence generator performed by the trial statistician (MK) who will remain blind to the arm allocation. We will use block randomisation with varying block sizes stratified by the clinic to achieve equal numbers in intervention and control groups within each clinic. Allocation concealment will be done with consecutively numbered, sealed, opaque envelopes.

Lay health workers delivering the intervention, field researchers, and participants cannot be blinded to the intervention. However, the determination of the primary outcome will be completed by TB nurses who are blinded to the intervention status of the participants based on routinely collected data.

The statistician will be blinded to the intervention or control arm allocation of participants during the analysis stage.

6. Outcomes

6.1 Primary outcome

The primary outcome is TB treatment success at six to nine months of follow-up. This is a binary variable defined as

- Success: cured or treatment completed
- Failure: failed treatment, death, acquired drug resistance, loss to follow-up or 'default', or not outcome evaluated.

The different mutually exclusive treatment outcomes are summarised here

Treatment outcome	Definition
Cure	Patient in whom baseline smear or culture was positive at beginning of treatment AND is smear/culture negative in the last month of treatment and on at least one previous occasion at least 30 days prior<br>According to local protocol, a patient who is diagnosed using Gene Xpert and is sputum negative for TB at 11 and 23 weeks is considered 'Cured'.
Treatment completed	Patient whose baseline smear or culture was positive at the beginning and has completed treatment but does not have a negative smear/culture in the last month of treatment and on at least one previous occasion > 30 days prior. Patients diagnosed with PTB whose baseline smear (or culture) result was negative and who started treatment based on clinical and radiological findings who have shown clinical improvement and completed the prescribed course of treatment.<br>*N.B. The smear examination may not have been done or the results may not be available at the end of treatment.*
Treatment failure	Patient whose baseline smear or culture was positive and remains or becomes positive again at 5 months or later during treatment.<br>Patients who were negative at baseline but were later found to be positive.<br>*N.B. This definition excludes those patients who are diagnosed with RR-TB or MDR-TB during treatment.*
Died	Patient who dies for any reason during the course of TB treatment.
Treatment default	Patient whose treatment was interrupted for two consecutive months or more during the treatment period.
Transfer out	Patient who was referred to a facility in another district to continue treatment and for whom the treatment outcome is not known.
Acquired resistance	Participants who are subsequently referred for MDR treatment.

6.2 Secondary outcomes

The following outcome measures will be recorded at the six-month follow-up:

- sputum conversion at the end of treatment in the group of participants who had bacteriology confirmed PTB at baseline [1]
- continuous smoking abstinence for identified smokers at baseline[2]

Whereas, the following will be assessed at three and six months follow-up:

- reduction in harmful or hazardous drinking[3]
- TB and ART medication adherence will be measured using a modified version of the AIDS Clinical Trials Group (ACTG) Adherence Questionnaire[4]; using an adherence index calculated by the formula (using the four-day recall table):

  [Total number of doses taken/Total number of doses prescribed] x 100

  Patients with at least 95% of adherence will be considered as having optimal adherence otherwise will be considered as having low (or suboptimal) adherence.
- increase in proportion of HIV-positive participants on ART at three and six months from baseline using standardised questions on the CRF.

6.2.1 Monitoring adverse events

Adverse events (AE) and serious adverse events (SAE) will be defined *apriori* and relevant information will be collected.

A **Serious Adverse Event (SAE)** is actually a special case of an adverse event where adverse outcomes are severe. It includes following events: Death of any of the participants associated with a clinical trial. **Examples of events**: Death, a life-threatening adverse event, inpatient hospitalization or prolongation of existing hospitalization, a persistent or significant.

An **adverse event (AE)** is any untoward medical occurrence in a patient or clinical investigation subject administered a pharmaceutical product and which does not necessarily have a causal relationship with this treatment.

The events are reported to ethics committee within 72 hours

[1] i.e. cure rates in intervention group versus control group for participants who initially had sputum AFB-positive, culture-positive or GeneXpert-positive PTB

[2] defined as a self-report of not smoking > 5 cigarettes six months from the start of the abstinence period, supported by a negative biochemical test CO < 7 ppm

[3] alcohol use will be measured using the AUDIT questionnaire. The questionnaire will be administered at screening (which will take place on the same day or shortly after the baseline assessment) and again at three months and six months.

[4] The questionnaire is a validated tool for measuring adherence specifically to ART and we will use an adapted version to also measure TB medication adherence [40].

The following information were collected to report the events:

Participants identification number, Gender, Age, Date of Enrollment, Arm (Control or intervention), Date of death notification to staff, Date of death , If death is related or not related to study.

6.3 Trial assessment schedule

Table 2 details the trial assessment schedule

Table 2: *Trial assessment schedule*

Assessment	*Items*	Pre randomisation	Timeline (post randomisation)				
		Eligibility (Day 0)	*Baseline (Day 0)*	*2 months*	*3 months*	*6-9 months*	*Trial end*
**ELIGIBILITY**							
Smoking status		X					
Smoking profile			X				
Alcohol profile		X					
Medical eligibility		X					
Eligible, consenting		X					
**MEASURES**							
Trial ID, visit date			X				
Socio-demographic history			X				
Depression screen			X		X	X	
Clinical review of TB Treatment record for disease information			X				X
Smoking history			X				
Smoking abstinence (self-report)					X	X	
Exhaled CO					X	X	
Record sputum culture or smear or Gene Expert result			X	X	X	X	
HIV Status			X				
ART Status (if HIV positive)			X		X	X	
AUDIT		X			X	X	
Modified ACTG (Follow up)					X	X	
Economic evaluation			X		X	X	

BMJ Publishing Group Limited (BMJ) disclaims all liability and responsibility arising from any reliance placed on this supplemental material which has been supplied by the author(s)

6.4 Other important information

In addition to the above, the following information was collected at baseline. Socio-demographic history included age, gender, marital status, education, employment status, and comorbidities. For details about these variables, see Table 3.

6.5 Fidelity of the intervention

The main fidelity analysis will be published somewhere else. Some descriptive statistics regarding fidelity will be added here once the main analysis is completed and the statistician is unblinded to the treatment arm.

Louwagie G, *et al. BMJ Open* 2022; 12:e056496. doi: 10.1136/bmjopen-2021-056496

7. Data

7.1 Data collection methods

The fieldworkers will screen all TB patients for eligibility immediately after the TB nurse at the clinic has initiated TB treatment and opened the TB “blue card”. Consent will be obtained for this screening phase as the alcohol related questions are sensitive and the fieldworkers must gain insight in the patient files.

Eligible and consenting patients will be enrolled in the trial and the baseline questionnaire and record review completed. Patients will be given a unique Trial Number that will be used on all research documents. Data will be collected and recorded by field workers equipped with Android phones with a mobile data collection application installed.
Participants in the control arm will continue with the routine TB care. Intervention arm study participants will be referred by the fieldworker to the lay counsellor for motivational interviewing. The first MI session will be on the same day of the completion of the screening questionnaire, where possible (with a 2-week window period). The second and third MI session will be scheduled 4 weeks and 8 weeks from the first counselling session respectively each time with a 2-week window period.
MI counselling and data collection will take place in a well-ventilated private area inside or outside the clinic, and audio-recorded after consent obtained. Fieldworkers and LHWs will be provided with high particulate respirator masks to minimise the risk of infection.
Fieldworkers will follow-up all participants in both arms at 3 and 6 months within a window period of 2-weeks before and 2-weeks after the ideal 3 and 6-month visit. Participants will receive SMS reminders 3 days before each planned visit. Participants will also be in a position to send “please call me “messages to the fieldworkers or district coordinators, who will then call the participant to solve problems that may have arisen with the appointment.
Patients who did not return for the planned 3 and 6 months visit will be contacted by telephone up to 3-times, as needed. Home visits will also be undertaken by existing clinic “tracer teams” or Community Based Outreach teams -where feasible - for participants who cannot be traced by telephone. The data collection process is illustrated in Table 3.

Table 3: Data Collection Process by time-point and details of data collection method

**Time-point**	**Information required at time-point**	**Data collection method**
**Baseline interview**	1. **<u>Socioeconomic and demographic status (to include history of mine work)</u>**<br>● Age<br>● Gender<br>● Marital status<br>● Educational level achieved<br>● Employment<br>● Mine work/type of mine work<br><br>2. Clinical information:<br>● Patient category (First episode vs recurrence)<br>● Site of disease<br>● Results of sputum smear, culture and Gene Xpert<br>● HIV status<br>● ART information<br>● Co-morbidities<br><br>3. Current smoking status and quit history, second hand smoke exposure<br><br>4. Alcohol history<br>5. Depression	TB Treatment Record<br><br>CRF – Questions to participant – demographic details<br><br>CRF – Questions to participant and information from the TB Treatment Record (as indicated in CRF)<br><br>Questions adapted from Global Adult Tobacco Survey Questionnaire<br><br>Baseline AUDIT score<br>CES-D
**3 months**	1. ART information<br><br>2. TB and ART medication adherence (modified ACTCG)<br><br>3. Alcohol history (repeat AUDIT)<br><br>4. Smoking history<br>5. SLT use	CRF – Question to patient<br><br>ACTG questionnaire for both TB medication and ART<br><br>AUDIT score at 3 month

Louwagie G, *et al. BMJ Open* 2022; 12:e056496. doi: 10.1136/bmjopen-2021-056496

	6. Depression	Follow-up questions as per Russell's Standard and exhaled CO<br><br>CES-D
**6 months**	1. TB treatment status <u>(Primary outcome)</u><br><br>2. Sputum smear or culture result<br><br>3. ART information<br><br>4. TB and ART medication adherence<br><br>5. Alcohol history (repeat AUDIT)<br><br>6. Smoking history and exhaled CO<br><br>7. Depression	TB treatment outcome from TB Treatment Record combined with information from TB record on cultures and smear results.<br><br>CRF- Question to patient<br><br>Follow-up ACTG questionnaire at 6 months<br><br>AUDIT at 6 months<br><br>Questions as per Russell's Standard and exhaled CO<br><br>CES-D

7.2 Data management

Sefako Makgatho University (SMU) appointed a data manager who will utilise an electronic platform for data collection, ensuring data quality, and facilitating the SMS messages.

Fieldworkers collecting research data will be equipped with Android mobile phones, which will have a mobile application installed on them to allow for data collection in areas with poor internet connectivity. The electronic data captured will be stored on secure and password protected storage servers and mobile phones, which ensure data privacy through only allowing authorised research staff access to the data.

The electronic data collection system used for the study requires an SMS gateway to send and receive messages to the research participants. Consenting participants' phone numbers, participant IDs, and associated SMS messages will be stored on the SMS gateway's secured and password-protected server.

Data quality will be ensured by providing fieldworkers with standard operating procedures (SOPs), training, and ongoing support on the importance of data quality, data collection, and data collection problem-solving. The data manager will continuously monitor the captured data for missing variables and inconsistencies in order to resolve any data problems.

The data manager will export the data from the secured server, conceal the participants study arm allocation, and de-identify the data before sharing the data in STATA and R compatible formats. The exported de-identified data will be stored in Dropbox, a secure cloud storage platform, for sharing with the lead trial statistician at the University of York for analysis.

All research data and documents referring to the PROLIFE trial will be stored and maintained in a secured storage space at SMU for a minimum of 15 years from the end of the PROLIFE trial. Study materials will be destroyed 15 years after the study.

Louwagie G, *et al. BMJ Open* 2022; 12:e056496. doi: 10.1136/bmjopen-2021-056496

8. Analysis

The computer packages STATA 16 (StataCorp. 2019) and R 3.5.3 (ref) will be used. Significance tests will be two-sided and the significance level is set at 0.05. The statistician will remain blind to allocation until results are finalised. We will follow the CONSORT statement guidelines in reporting.

Below, we detail the analyses that we will carry out for the data collected at baseline, the primary outcome, the secondary outcomes, and adverse events. We also list the sensitivity analyses that we might perform and subgroup analyses.

8.1 Participants Flow

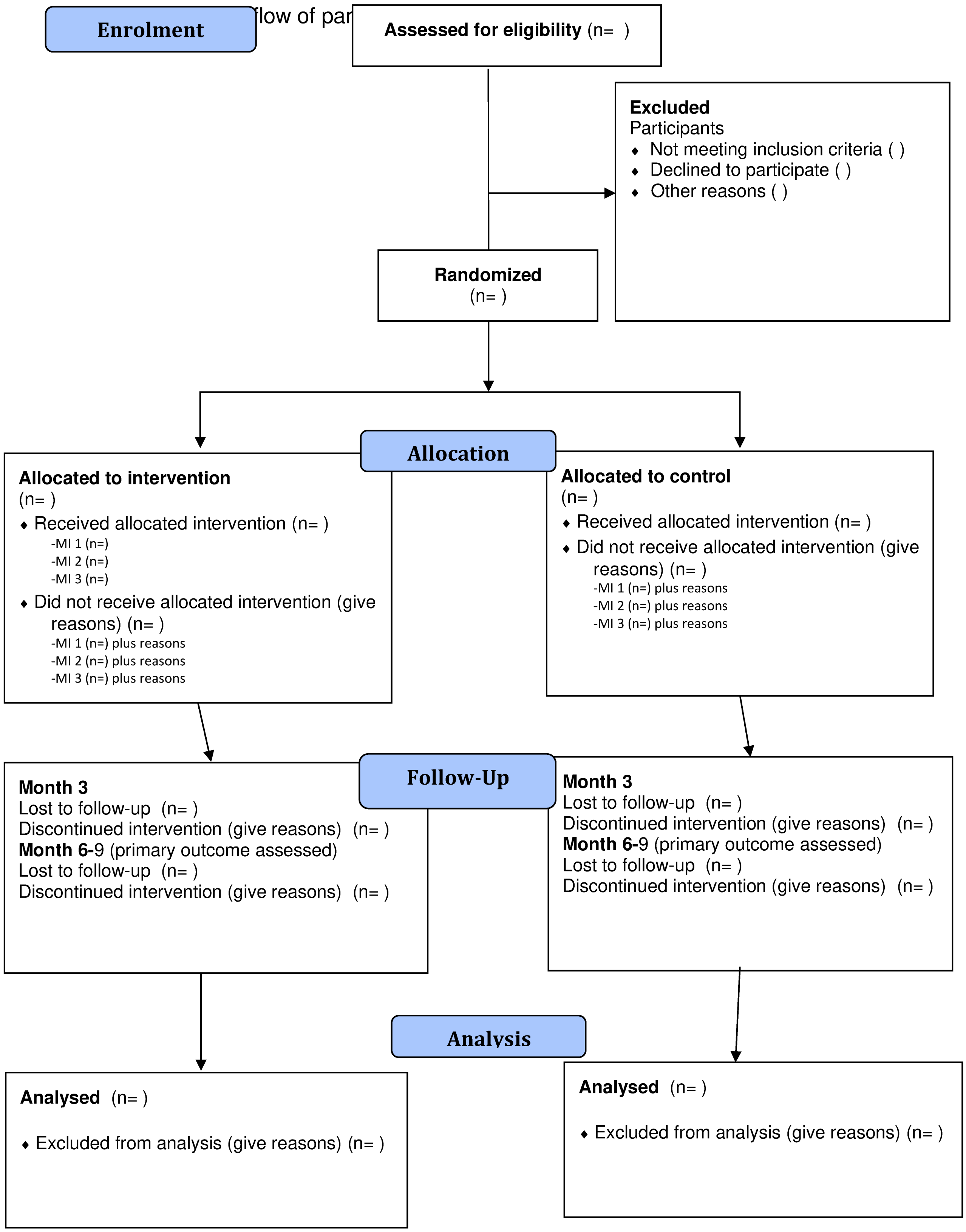

Figure 1 CONSORT DIAGRAM SHOWING FLOW OF PARTICIPANTS

8.2 Baseline data

Baseline data Analysis Plan:

Baseline data including demographic variables will be summarised descriptively by trial arm, but no formal statistical comparisons will be undertaken. Continuous measures will be reported as means and standard deviations (SD) while categorical data will be reported as counts and percentages, see Tables 1 to 4 in Appendix A. For skewed continuous measures, we will also provide medians and interquartile ranges.

Baseline data results:

Consent:

In the control arm (Arm1) 286 gave written consent and 5 verbal consent for participation in the study and for access to their medical records
In the intervention arm (Arm2) 279 gave written consent and 4 verbal consent for participation in the study and for access to their medical records

Baseline Imbalances

Education:

It seems that for education there is imbalance between the two arms for Grades 8-11/Grade 12/Higher; the control arm (Arm1) had a higher educated group the difference in percentage points 8.2% as opposed to 2.8% in the intervention arm, see Table 2 for further details. There are 9 participants who are not literate in the study (7 in the control arm (Arm1) and 5 in the intervention arm (Arm2)).

Drinking

In the intervention arm, 223 (78.8%) had a drink in the past 12-months compared to 208 (71.5%) in the control arm, see Table 3.
In the control arm (Arm1), 110 (37.8%) were drinkers only, 83 (28.5%) were smokers only, and 98 (33.7%) were smokers and drinkers compared to 92 (32.5%), 60 (21.2%), and 131 (46.3%) in the intervention arm (Arm2), respectively.

Louwagie G, et al. BMJ Open 2022; 12:e056496. doi: 10.1136/bmjopen-2021-056496

Assets:

The vast majority had a radio (84%), a television (89%), a refrigerator (85%), an electric or gas stove (86%), and a microwave (67%), however, only 50% had a washing machine. Furthermore, a minority had a landline telephone (7.5%), a desktop or laptop computer (20%:), and a vacuum cleaner or floor polisher (15%). Figure 2 is a boxplot of the total number of assets by study arm. The spread in the control arm (Arm1) is greater than that in the intervention arm (Arm2). However, the mean number of assets is similar across the two groups of 5 (SD: 1.87).

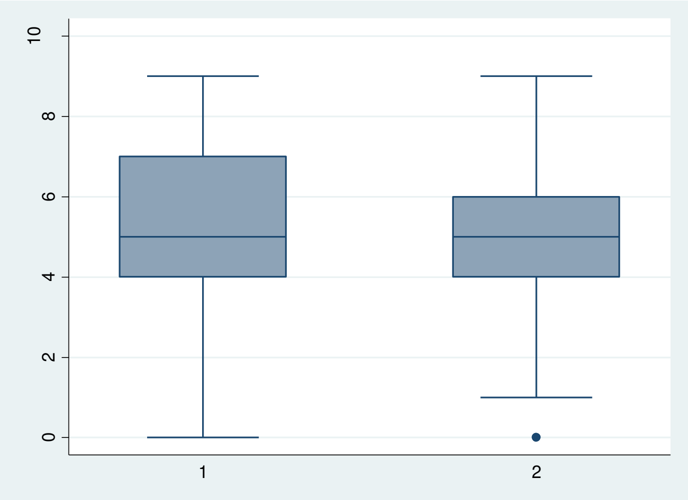

Figure 2: A boxplot of the total number of assets by study arm

Louwagie G, et al. BMJ Open 2022; 12:e056496. doi: 10.1136/bmjopen-2021-056496

Medical History:

There were 305 (53.2%) **HIV positive** participants out of which 204 (65.38%) were using Cotrimoxazole and 257 (82.37%) were undergoing anti-retroviral therapy, see Table 2.

TB History: The vast majority of participants were New TB patients 513(91.3%), with Pulmonary only (ICD-10 A15) being the site of disease for the vast majority 553 (98.9%). Among those with results known, the majority had one pre-treatment smear result 366 (89.9%), one Gene XPert recorded 435 (97.5%), and one culture result recorded 96 (96%).

Among those with results available, 220 (58.51%) had at least **one positive smear** result,362 (87.23%) had at least **one positive Gene XPert result**, and 35 (47.95%) at least **one positive culture result**.

However, 85 (53.46%) in the control arm had at least one positive smear result within 60 days of the TB treatment start date compared to 96 (61.15%) in the intervention arm.

In addition, only 34 had their culture results within 60 days from the TB start dates of which 21 had positive results; 11 in the control arm compared to 10 in the intervention arm.

The vast majority of participants did not have any **co-morbidities** 525 (96.3%) and 18 (3.3%) had one.

Baseline Smoking Related variables:

Thirty five percent (202) did not smoke in the past month, whereas 52% smoked daily and the remaining 13% smoked in the past month but less frequently than on a daily basis, see Table 3.

In the past 30 days, the vast majority were exposed to smoke for seven days or less at: home 462 (80.5%); cafes/restaurants 464 (80.8%); Shebeens, bars or clubs 432 (75.3%); Bus/train/taxi/ vehicle 448 (78.0%); and Shops/shopping mall 461 (80.3%) and where applicable at the workplace 173 (66.8%).

Among those who smoked in the past month 372: 181 in the control arm (Arm1) vs 191 in the intervention arm (Arm2),

345 (92.7%) **smoked manufactured cigarettes** of which 225(65%) did so on a daily basis in the past seven days whereas 40 (11.6%) did not smoke in the past week. The mean number of days smoked was 5.42 days in the past week (SD: 2.49) with 6.34 cigarettes (SD: 7.39) smoked daily on average.

Hand-Rolled cigarettes were used by 35 participants of which 16 (45.7%) smoked daily in the past week. They smoked on average for 4.11 (SD:2.91) days in the past week and on average smoked 3.77 (SD: 3.27) hand-rolled cigarettes per day.

There was only one person who exclusively smoked waterpipe, two who exclusively smoked pipe, and seven who exclusively used other formats of tobacco other than the ones that are listed here.

For the vast majority the total number of cigarettes smoked is based on their answer to manufactured cigarettes. In 27 cases, they supplemented this with other sources and 8 used only other forms to report the number smoked on average per day.

Over the past 3 months, they **spent** on average 174.58 (SD: 181.61) Rands per week on tobacco products.

Supplemental material BMJ Publishing Group Limited (BMJ) disclaims all liability and responsibility arising from any reliance placed on this supplemental material which has been supplied by the author(s) BMJ Open

Smoking restrictions

No smoking was allowed **inside home** for 204(54.8%) participants, whereas 119(32.0%) had some rule where/when it is allowed and 49(13.2%) had no rules in place.

Quit Attempts:

Among smokers, 116 (31.2%) made an **attempt to quit**; the mean number of attempts to quit was 2.56 (SD: 2.32).

Furthermore, 213 (57.3%) said they will probably **try to quit** smoking completely and permanently in the next three months and 126 (33.9%) said that they definitely will.

Whereas, 207 (55.6%) said they will probably **quit** smoking completely and permanently in the next three months and 130 (34.9%) said that they definitely will.

Only 39 (10.5%) have ever used any methods in the past 3 months to help them stop smoking tobacco. These spent on average 136.64 (SD: 205.36) Rands on methods to help you stop smoking in the past 3 months.

Smokeless tobacco was used by only 24 participants (4.2%). They have been using ST for an average of 10.5 years (SD: 8.8) and have started using it at the age of 27 (SD: 11.33) years on average.

Heaviness of smoking:

In the control arm (Arm1), 67(37.0%) of smokers reported smoking within 5 minutes of waking up whereas 82(42.9%) did so in the intervention arm (Arm2).

Among those who smoked and who reported the number of cigarettes/pipes/cigars they used on average per day, 134(74.03%) in the control arm (Arm1) and 158(82.72%) in the intervention arm (Arm2) were considered as heavy smokers.

Louwagie G, *et al. BMJ Open* 2022; 12:e056496. doi: 10.1136/bmjopen-2021-056496

8.3 Primary analysis

8.3.1 Primary outcome definition:

As per section 6.1, we would define the primary outcome as those who had a successful treatment versus not. Where successful treatment is considered if the patient is considered to have been cured or treatment completed; all other categories will be deemed as not successful. The successful treatment[5] categories are defined as follows:

Cure	Patient in whom baseline smear or culture was positive at beginning of treatment AND is smear/culture negative in the last month of treatment and on at least one previous occasion at least 30 days prior According to local protocol, a patient who is diagnosed using Gene Xpert and is sputum negative for TB at 11 and 23 weeks is considered 'Cured'.
Treatment completed	Patient whose baseline smear or culture was positive at the beginning and has completed treatment but does not have a negative smear/culture in the last month of treatment and on at least one previous occasion > 30 days prior. Patients diagnosed with PTB whose baseline smear (or culture) result was negative and who started treatment based on clinical and radiological findings who have shown clinical improvement and completed the prescribed course of treatment. *N.B. The smear examination may not have been done or the results may not be available at the end of treatment.*

8.3.2 Primary outcome Analysis

For the primary outcome, we will conduct analysis on an intention-to-treat basis. We will use binary logistic regression to compare the main outcome between the

[5] Following discussion with the team, we will take this at face value as it is not possible to query some of the anomalies found in the recording of dates of tests.

intervention and the usual care arm. We will also investigate any potential clustering at the centre level and account for it. We will present the results for this analysis in Table 6.

We will also adjust for HIV status, sex, alcohol versus tobacco versus both, and district; if these differ between trial arms at baseline.

8.3.3 Primary outcome Analysis Results

Table 4 gives the descriptive statistics for the initial outcome and the derived - dichotomised outcome; these indicate that overall, 69% were classified as successful treatment based on the medical professional assessment (cured/treatment completed). This percentage was similar for the two arms with the control arm (Arm1) having a slightly higher percentage of success of 70.1% compared to 67.8% in the intervention arm (Arm2)[6]. Table 9 gives the distribution per centre by study arm. Generally, these are fairly balanced any imbalance observed is most probably due to the early termination of the study.

For 203 participants, the TB treatment outcome date was not available, with those participants more likely to not have been cured (59% not cured), however, the percentage was similar across the two study arms for those with missing TB treatment outcome date.

The odds of successful treatment is 0.9 (95% CI: (0.64,1.27)) in the intervention arm (Arm2) compared to the control arm (Arm1). This estimate is very similar to the estimate adjusting for district and drinking/smoking status. This is also the case if you further adjust for sex and HIV status at baseline where the OR is 0.86 (95% CI: (0.60,1.24)); see Table 6 for further details.

[6] Primary outcome Control: 204/291, 95% CI proportion 0.70 (0.64,0.75)
Primary outcome Intervention:192/283, 95% CI proportion 0.68 (0.62,0.73)

Supplemental material BMJ Publishing Group Limited (BMJ) disclaims all liability and responsibility arising from any reliance placed on this supplemental material which has been supplied by the author(s) BMJ Open

8.4 Secondary analyses

Secondary analyses plan

In the group of participants who had bacteriology confirmed at baseline, we will use logistic regression to compare cured versus not cured, as indicated by the outcome at the end of treatment, between the two study arms. We will also control for baseline characteristics and other covariates such as sex, alcohol use, HIV-status, district, and account for any potential clustering by centre.

We will use a similar approach for the six-months continuous smoking abstinence outcome. This analysis will be performed on the group of participants who were current tobacco smokers at baseline. We will also control for baseline characteristics and other covariates such as age, duration of smoking, alcohol problem (hazardous, harmful, non-drinker/light drinker), heaviness of smoking index[7], depression, and potentially HIV-status.

For the reduction in harmful or hazardous drinking, we will use linear regression to measure difference in total AUDIT score between control and intervention groups accounting for the baseline AUDIT scores. Separate analyses for the AUDIT at 3 and 6 months will be performed.

The AUDIT is a 10-items questionnaire with a range between 0 and 40 where higher values indicate higher dependency.[8] It is worth mentioning that a score of 8 or more

[7] [Goedele's Comment on an earlier Version]Definitely a measure of severity of smoking and duration of smoking. For example Heaviness of Smoking Index which can be derived as follows:

HSI=Heaviness of smoking index ≥ 4, calculated based on sum of time to first cigarette (0: 61+min, 1:31-60 min, 2: 6-30 min, 3: ≤5 min) and number of cigarettes smoked per day (0: 0-10 cigarettes per day [CPD]

Also: age, duration of smoking, alcohol problem (hazardous, harmful, non-drinker/light drinker), maybe HIV-status, (adding this may reduce your sample size too much, because of missing HIV-status, unless you include HIVstatus unknown as a category of HIV-status). Depression

[8] Scoring the audit
Scores for each question range from 0 to 4, with the first response for each question (eg never) scoring 0, the second (eg less than monthly) scoring 1, the third (eg monthly) scoring 2, the fourth (eg weekly) scoring 3, and the last response (eg. daily or almost daily) scoring 4. For questions 9 and 10, which only have three responses, the scoring is 0, 2 and 4 (from left to right).
A score of 8 or more is associated with harmful or hazardous drinking, a score of 13 or more in women, and 15 or more in men, is likely to indicate alcohol dependence.

Louwagie G, et al. BMJ Open 2022; 12:e056496. doi: 10.1136/bmjopen-2021-056496

is associated with harmful or hazardous drinking, a score of 13 or more in women, and 15 or more in men, is likely to indicate alcohol dependence. Eligibility criteria for our study is an AUDIT score ≥ 8 for men or ≥ 7 for women but <20. However, if assumptions of linear regression are not met we will either transform the data or use alternative regression analyses such as ordinal logistic regression.

Adherence to TB and ART medication will be measured using an adherence index based on a modified version of the AIDS Clinical Trials Group (ACTG) Adherence Questionnaire; where patients with at least 95% of adherence will be considered as having optimal adherence otherwise will be considered as having low (or suboptimal) adherence. We will use logistic regression to model patient's characteristics (age, sex, alcohol, smoking status, depression) that might influence adherence at 6-month; we will also compare adherence between study arms. Similar to the previous outcomes, we will account for any potential clustering by centre.

We will also report the proportion of HIV-positive participants on ART at six months and compare these to the baseline using standardised questions on the CRF.

8.4.1 Secondary outcome definitions that involve defining a positive baseline test:

To operationalise the above we need to define how we determine if someone has a positive baseline smear or culture. Each participant might have up to two tests of the following: smear test, GeneExpert test, and culture test.

If a test was administered two times then to be considered negative both tests should be negative, otherwise it is considered positive. If a test was administered only once, then the result of that instance is taken as is.

1Saunders JB, Aasland OG, Babor TF et al. Development of the alcohol use disorders identification test (AUDIT): WHO collaborative project on early detection of persons with harmful alcohol consumption — II. Addiction 1993, 88: 791–803

If for a patient more than one test was administered, then to be considered negative the result should be negative under both tests.

This is illustrated in the following table.

Baseline	P=+, N = -		
		XXXX Test at run 2 (Result 2)	
XXXX Test run 1 (Result 1)		N	P
	N	NN	NP
	P	PN	PP
XXXX=Smear or Culture or GeneXpert			
Smear test at baseline	N1	NN	
	P1	NP, PN, PP	
Genexpert	N2	NN	
	P2	NP, PN, PP	
culture	N3	NN	
	P3	NP, PN, PP	
Positive at baseline	PB	P1 or P2 or P3	
Negative at baseline	NB	N1 and N2 and N3	

To define a conversion among those who were positive at baseline, we used the primary outcome response category cured to indicate a negative result at month 6 per the nurse’s assessment.

Among the 403 participants who were positive at baseline 168(41.69%) were recorded as cured by 6-month of these 83 (39.9%) in the control arm compared to 85 (43.59%) see Table 4. The odds ratio of conversion is 1.16 (95% CI: (0.83,1.63)) comparing the intervention arm to the control arm. When adjusting for district, sex, and smoking/drinking status and HIV status at baseline, the OR reduces to 1.07 (95% CI: (0.76,1.51))

Louwagie G, et al. BMJ Open 2022; 12:e056496. doi: 10.1136/bmjopen-2021-056496

8.4.2 6-months Continuous Abstinence

For this outcome, those who smoked cigarettes at baseline were considered as the analytic sample. In addition, those where the following could not be ascertained: self-report of not smoking > 5 cigarettes six months from the start of the abstinence period and supported by a negative biochemical test CO < 7 ppm were considered as smokers for the analysis of this variable. The number of participants who identified as cigarette smokers at baseline were 345 (60.1%)[9].

23 (85.19%) out of 27 with three measurements available[10] managed to abstain continuously for six months. These were similarly distributed across the two study arms, see Table 4. Among those who identified as cigarette smokers, 10 (5.59%) participants in the intervention arm continuously abstained. In the control arm there were 12 (7.23%) who continuously abstained for 6-months.

The crude odds of 6-months continuous abstinence is 0.76 (95% CI: (0.35,1.63)) in the intervention arm compared to the control arm among baseline cigarette smokers. Given the limited number of those who were identified as continually abstained, we were only able to adjust for one additional variable at a time. Adding one of the following variables: heaviness of smoking, type of drinker at baseline, age when started smoking, and the duration of smoking at baseline, the adjusted odds ratio of continuous abstinence comparing the intervention to the control arm did not differ much from the crude estimate of 0.76. The adjusted estimate for the various models ranged between 0.73 and 0.76 with similar confidence limits as for the crude estimate. Furthermore, we did not have evidence that any of the adjusting variables were statistically significantly correlated to continuous abstinence in these models.

We carried an additional analysis where those who died and were smokers at baseline, 22 in total, 20 were cigarette smokers and were removed from the analytical sample for the continuous abstinence outcome. This resulted in a crude

[9] Any type of tobacco smoking at baseline 372 (64.8%). The numbers reflect those who used manufactured cigarettes which were the vast majority.
[10] We had only 27 participants who had self-report of not smoking at 3 months, 6 months and a carbon monoxide reading at 6 months. Continuous abstinence was defined as a self-report of not smoking > 5 cigarettes six months from the start of the abstinence period and supported by a negative biochemical test CO < 7 ppm

OR of 0.78 which is similar to the OR when the larger analytic sample was considered; this was also the case for the associated 95% CI. Similar estimates were observed when adjusting for the aforementioned variables.

We carried an additional analysis where the analytic sample was all those who were smokers regardless of the type used. There were a total of 372 that identified as smokers. The crude odds ratio in this case changes to 0.86 (95% CI: (0.38,1.95)). The 95% CI is similar to that of the smaller analytic sample. When adjusting for the heaviness of smoking, type of drinker at baseline, age when started smoking, and the duration of smoking at baseline, the adjusted odds ratio of continuous abstinence comparing the intervention to the control arm did not differ much from the crude estimate.

3-months Continuous Abstinence

Among those who identified as cigarette smokers, 20 (11.17%) participants in the intervention arm continuously abstained for 3-months while in the control arm there were 27 (16.27%) who continuously abstained for 3-months.

The crude odds of 3-months continuous abstinence is 0.65 (95% CI: (0.37,1.14)) in the intervention arm compared to the control arm among baseline cigarette smokers. Given the limited number of those who were identified as continually abstained, we were only able to adjust for one additional variable at a time. Adding one of the following variables: heaviness of smoking, type of drinker at baseline, age when started smoking, and the duration of smoking at baseline, the adjusted odds ratio of continuous abstinence comparing the intervention to the control arm did not differ much from the crude estimate of 0.65. The adjusted estimate for the various models ranged between 0.63 and 0.66 with similar confidence limits as for the crude estimate.

Louwagie G, *et al. BMJ Open* 2022; 12:e056496. doi: 10.1136/bmjopen-2021-056496

Supplemental material BMJ Publishing Group Limited (BMJ) disclaims all liability and responsibility arising from any reliance placed on this supplemental material which has been supplied by the author(s) BMJ Open

Point Abstinence

There were 57 participants who indicated that they stopped smoking tobacco completely at the 3-month follow-up, of which one had more than 5 cigarettes in the past 3 months. There were 81 participants who indicated that they stopped smoking tobacco completely at the 6-month follow-up, of which three had more than 5 cigarettes in the past 3 months. Only 30 participants had information for the entirety of the 6-month period, of which none consumed more than 5 cigarettes over the past 6-month period. Of these, 23 had a confirmed CO < 7 ppm, 4 had these levels >= 7, and 3 were missing.

182 responded that they continued smoking either as usual or at a reduced rate but regularly at month 3; whereas 133 done so at month 6 of these we had 97 that had measurements at both time points thus resulting in a total of 218 where they have smoked on a regular basis over the past 6 month period. 11 had responded as not smoking in the past three months at month 6 but had missing information for the first three months; 9 of these had carbon monoxide readings available at month 6. Of these nine, two had their CO >= 7 (in fact these were 10 & 10.1).

Louwagie G, *et al. BMJ Open* 2022; 12:e056496. doi: 10.1136/bmjopen-2021-056496

8.4.4 **Change in harmful or hazardous drinking at month 3 and 6 follow-ups**

Alcohol use was measured using the AUDIT questionnaire. We will assess whether there has been a reduction in alcohol consumption three months and six months following recruitment. This analysis will be performed on the group of participants who were harmful or hazardous drinkers at baseline. Figure YYY provides histograms of the AUDIT score at baseline, 3-months and 6-months, respectively, among those who were considered as hazardous or harmful drinkers. It also presents scatterplots of the 3-months and 6-months scores versus the baseline scores for this group by trial arm, respectively.

Among those who were harmful or hazardous drinkers at baseline those in the intervention arm had on average a reduction of 0.04 points (95% CI: (-2,1.91)) on the AUDIT score at 6-months compared to those in the control arm controlling for their baseline score, see Table 6. However, when additionally adjusting for district, sex, and smoking/drinking status (which effectively flags smokers/non-smokers who are also drinkers) and HIV status at baseline; the intervention arm had an average increase of 0.02 points on the AUDIT score (95% CI: (-1.55,1.6)) compared to the control arm. It is worth noting that of the variables in the adjusted model; the only statistically significant result is for the district variable. It seems that those in district “S” score on average 5.8 points less than those in “B” (95% CI: (-11.26,-0.35)); similarly those in “L” score 5 points less than those in “B” on AUDIT but we do not have evidence that this difference is statistically significant (95% CI: (-10.35,0.26)), see Table 6 for further details.

At 3-month, the estimates were an average increase of 0.55 (95% CI: (-1.01,2.11)) on the AUDIT score in the intervention arm compared to the control arm when only accounting for the baseline scores; whereas it increased to 0.74 (95% CI: (0.62,2.1)) when adjusting for other covariates in the model, for further details see Table 7.

Louwagie G, et al. BMJ Open 2022; 12:e056496. doi: 10.1136/bmjopen-2021-056496

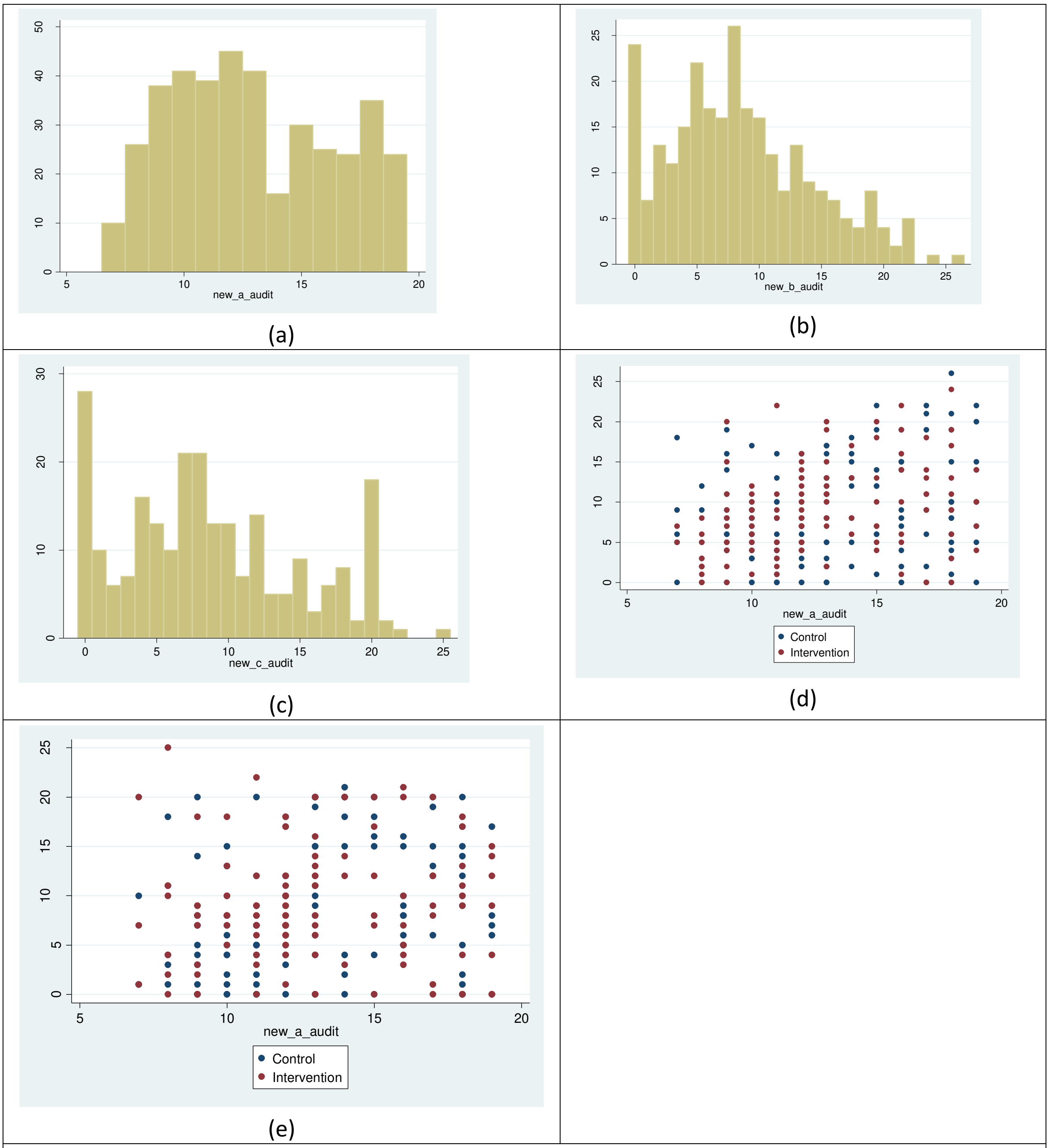

Figure YYY: Graphs (a), (b), and (c) are histograms of the AUDIT score at baseline, 3-months and 6-months, respectively, among those who were considered as hazardous or harmful drinkers. (d) and (e) are scatterplots of the 3-months and 6-months scores versus the baseline scores for this group, respectively.

Louwagie G, *et al. BMJ Open* 2022; 12:e056496. doi: 10.1136/bmjopen-2021-056496

8.4.5 Proportion of ART uptake of HIV-positive participants at month 3 and 6 follow-ups

We will assess whether there has been an increase in proportion of HIV-positive participants on ART at three and six months from baseline using standardised questions on the CRF.

There were 171 HIV-positive participants whose baseline ART medication status was known and whose ART medication status was known as well at 6-months. Of these, 123 remained on their medication, 19 took up medication at 6-months compared to not taking medication at baseline, whereas 29 stopped taking their medication at 6-months but were on medication at baseline, see Table 6 for further details.

There were 10 who had an unknown status in terms of medication at baseline and no information was available about them at 6-months, 12 who were initially of unknown medication status who took up medication at 6-months (these were equally distributed between the two arms). Furthermore, there were 10 who were not taking medication at baseline and 102 who were taking medication at baseline whose 6-months medication status was not recorded.

At 6-months, the odds ratio of taking medication at 6-months was 2.05 (95% CI: (0.80,5.27)) in the intervention arm compared to the control arm, controlling for ART baseline medication status.

There were 188 HIV-positive participants whose baseline ART medication status was known and whose ART medication status was known as well at 3-months. Of these, 122 remained on their medication, 16 took up medication at 3-months compared to not taking medication at baseline, whereas 50 stopped taking their medication at 3-months but were on medication at baseline, see Table 7 for further details.

There were 9 who had an unknown status in terms of medication at baseline and no information was available about them at 3-months, 11 who were initially of unknown

medication status who took up medication at 3-months, and two who were initially of unknown medication status who were not taking medication at 3-months. Furthermore, there were 9 who were not taking medication at baseline and 82 who were taking medication at baseline whose 3-months medication status was not recorded. Furthermore, there were 4 who carried on not taking medication at 3-months.

At 3-months, the odds ratio of taking medication at 6-months was 0.79 (95% CI: (0.38,1.65)) in the intervention arm compared to the control arm, controlling for ART baseline medication status.

Medicine Adherence:

At 3-months 165 (98.8%) of 167 participants had optimal ART medication adherence, whereas 139(97.2%) of 143 had optimal ART medication adherence. These were similar across the two arms.

Similarly, at 3-months 319(91.67%) of 348 participants had optimal TB medication adherence, whereas 120(90.23%) of 133 had optimal TB medication adherence. These were similar across the two arms.

8.5 Subgroup analyses (See above analyses)

We will conduct subgroup analyses to determine whether TB treatment outcomes differ between subgroups, as follows: HIV-positive versus HIV-negative participants; participants with an alcohol problem only versus smokers only versus participants who are conjoint smokers and drinkers; and participants who were GeneXpert positive versus participants who were GeneXpert negative at baseline.

8.5 Sensitivity analyses

In case of missing data, multiple imputations and appropriate sensitivity analyses will be conducted. As it is likely that more than one variable will have missing data we will use multiple imputations using chained equations (**MICE**). A minimum of 10 imputations will be performed; however, the final number of imputations will depend on the missing in the data. We will report the decisions that we make with regard to the number of imputations and the

Supplemental material BMJ Publishing Group Limited (BMJ) disclaims all liability and responsibility arising from any reliance placed on this supplemental material which has been supplied by the author(s) BMJ Open

variables we use in the imputations. We will also conduct a sensitivity analysis to explore the implications of the missing at random assumption [21p,22p].

8.6 Adverse events

Analysis of adverse events and serious AE will explore whether these differ by treatment arm using Chi-square tests.

8.7 Planned interim review and analyses

No interim analysis is planned. The main analyses will be completed after three months of the data closing.

Louwagie G, et al. BMJ Open 2022; 12:e056496. doi: 10.1136/bmjopen-2021-056496

8.8 List of Tables and Graphs

Measures of central tendency and percentages will be reported to two decimal places whereas measures of variability and p-values will be reported to three decimal places.

The following is a list of suggested tables and graphs; the templates are included in Appendix A.

TABLE 1: Numbers in the study at Baseline and follow-ups at month 3, and 6 by centre and study arm.

TABLE 2: Descriptive statistics for socio-demographic and socio-economic characteristics at baseline and as analysed by study arm. Frequencies and (percentages) are presented unless otherwise stated

TABLE 3: Descriptive statistics for smoking history, alcohol history, clinical characteristics and depression score at baseline and as analysed by study arm. Frequencies and (percentages) are presented unless otherwise stated.

TABLE 4: Descriptive statistics for primary and secondary outcomes by study arm at baseline (where available), 3 month (where available) and 6 month. Frequencies and (percentages) are presented unless otherwise stated.

TABLE 5: Number and type of adverse events at month 2, 3, and 6 by centre and study arm.

TABLE 6: Regression analysis results for the primary and secondary outcomes at 6 month. Estimates presented with corresponding 95% CI. Crude and adjusted estimates are provided.

TABLE 7: Regression analysis results for the secondary outcomes that are measured at 3 month. Estimates presented with corresponding 95% CI. Crude and adjusted estimates are provided.

9. SAP amendment log

**Amendment/addition to SAP and reason for change**	**New version number, name and date**
SAP completed and signed-off	*V1.0,*
Updated verion	*V1.1, May 2020*

10. Signatures of approval

Sign-off of the final approved version of the Statistical Analysis Plan by the principle investigator and trial statistician(s) (can also include Trial Manager/Co-ordinator)

**Name**	**Trial Role**	**Signature**	**Date**

11. References

Moriarty, A.S., Louwagie, G.M., Mdege, N.D. *et al*. ImPROving TB outcomes by modifying LIFE-style behaviours through a brief motivational intervention followed by short text messages (ProLife): study protocol for a randomised controlled trial. *Trials* **20**, 457 (2019) doi:10.1186/s13063-019-3551-9

Department of Health, Republic of South Africa. National Tuberculosis Management Guidelines. 2014. http://www.tbonline.info/media/uploads/documents/ntcp_adult_tb-guidelines-27.5.2014.pdf. Accessed 8 Jan 2018

21p. James R Carpenter and Michael G Kenward. Missing data in clinical trials — a practical guide. Birmingham: National Health Service Co-ordinating Centre for Research Methodology, 2008.

22p. James R Carpenter, Michael G Kenward, and Ian R White. Sensitivity analysis after multiple imputation under missing at random — a weighting approach. Statistical Methods in Medical Research, 16:259–275, 2007.

StataCorp. 2019. *Stata Statistical Software: Release 16*. College Station, TX: StataCorp LLC.

R Core Team (201?). R: A language and environment for statistical computing. R Foundation for Statistical Computing, Vienna, Austria. URL http://www.R-project.org/.

Supplemental material BMJ Publishing Group Limited (BMJ) disclaims all liability and responsibility arising from any reliance placed on this supplemental material which has been supplied by the author(s) *BMJ Open*

12. Appendix A (Results tables)

Table 1: Numbers in the study at Baseline and follow-ups at month 3 and 6 by centre and study arm. (SEE FLOWCHART)

TABLE 1: NUMBERS IN THE STUDY AT BASELINE AND FOLLOW-UPS AT MONTH 3 AND 6 BY CENTRE AND STUDY ARM.								
Centre	Baseline		Month 3		Month 6			
	Intervention	Control	Intervention	Control	Intervention	Control		
Total								

Louwagie G, *et al. BMJ Open* 2022; 12:e056496. doi: 10.1136/bmjopen-2021-056496

TABLE 2: Descriptive statistics for socio-demographic, socio-economic, and clinical characteristics at baseline by study arm. Frequencies and (percentages) are presented unless otherwise stated

TABLE 2: DESCRIPTIVE STATISTICS FOR SOCIO-DEMOGRAPHIC, SOCIO-ECONOMIC, AND CLINICAL CHARACTERISTICS AT BASELINE AND AS ANALYSED BY STUDY ARM. FREQUENCIES AND (PERCENTAGES) ARE PRESENTED UNLESS OTHERWISE STATED				
		Baseline		
	N1/N2	Control	Intervention	Total
		Arm 1	Arm 2	
**Age in years: mean (SD)**	291/283	39.37 (12.60)	38.56 (11.15)	
**Age in years: median (IQR)**				
**Gender**				
Female		69 (23.7)	60 (21.2)	129 (22.5)
Male		222 (76.3)	223 (78.8)	445 (77.5)
Do not want to disclose				
Marital status				
Married or living together		102 (35.1)	95 (33.6)	197 (34.3)
Divorced/separated		20 (6.9)	18 (6.4)	38 (6.6)
Widowed		10 (3.4)	7 (2.5)	17 (3.0)
Never married and never lived together		144 (49.5)	150 (53.0)	294 (51.2)
Declined to answer		15 (5.2)	13 (4.6)	28 (4.9)
**Education**				
No education		7 (2.4)	5 (1.8)	12 (2.1)
Grades 1-5		23 (7.9)	20 (7.1)	43 (7.5)
Grades 6-7		32 (11.0)	35 (12.4)	67 (11.7)

Grades 8-11		96 (33.0)	128 (45.2)	224 (39.0)
Grade 12		87 (29.9)	70 (24.7)	157 (27.4)
Higher		24 (8.2)	8 (2.8)	32 (5.6)
Declined to answer		22 (7.6)	17 (6.0)	39 (6.8)
**Employment**				
Self-employed (full-time)		30 (10.3)	36 (12.7)	66 (11.5)
Employed full-time (30 hrs a week or more)		62 (21.3)	54 (19.1)	116 (20.2)
Employed part-time (less than 30 hrs a week)		19 (6.5)	29 (10.2)	48 (8.4)
Retired		17 (5.8)	16 (5.7)	33 (5.7)
Unemployed (but able to work)		125 (43.0)	120 (42.4)	245 (42.7)
Unable to work because of long-term disability or ill health		9 (3.1)	8 (2.8)	17 (3.0)
Full-time student		12 (4.1)	4 (1.4)	16 (2.8)
Caring from my home and family/doing household work/housewife		0 (0.0)	2 (0.7)	2 (0.3)
Occasional work (“piece job”)		17 (5.8)	12 (4.2)	29 (5.1)
Declined to answer		0 (0.0)	2 (0.7)	2 (0.3)
**Ever worked or spent time in mines**				
No		244 (83.8)	237 (83.7)	481 (83.8)
Yes		46 (15.8)	45 (15.9)	91 (15.9)
**Socioeconomic status**				

**Household items (Yes is displayed)**				
A radio		249 (85.6)	234 (82.7)	483 (84.1)
A television		255 (87.6)	255 (90.1)	510 (88.9)
A landline telephone		21 (7.2)	22 (7.8)	43 (7.5)
A desktop or laptop computer		67 (23.0)	48 (17.0)	115 (20.0)
A refrigerator		248 (85.2)	240 (84.8)	488 (85.0)
A vacuum cleaner or floor pol		49 (16.8)	35 (12.4)	84 (14.6)
A microwave oven		198 (68.0)	189 (66.8)	387 (67.4)
An electric or gas stove		254 (87.3)	238 (84.1)	492 (85.7)
A washing machine		153 (52.6)	136 (48.1)	289 (50.3)
Total number of assets: mean (SD)		5.14 (1.96)	4.94 (1.77)	5.04 (1.87)
**In the past month, number of days you or people in the household went to bed hungry because there was no food to eat**				
0 days		244 (83.8)	238 (84.1)	482 (84.0)
1-7 days		45 (15.5)	34 (12.0)	79 (13.8)
More than 7 days		2 (0.7)	9 (3.2)	11 (1.9)
Declined to answer		0 (0.0)	2 (0.7)	2 (0.3)
**TB and medical history**				
**TB history**				
**Patient category**				
New patient		264 (92.3)	249 (90.2)	513 (91.3)
Relapse		10 (3.5)	9 (3.3)	19 (3.4)
Re-treatment after default		9 (3.1)	14 (5.1)	23 (4.1)
Re-treatment after failure		1 (0.3)	2 (0.7)	3 (0.5)
Other		2 (0.7)	2 (0.7)	4 (0.7)
**Site of disease**				
Pulmonary and Extra Pulmonary (ICD-10 A17-A19)		3 (1.1)	3 (1.1)	6 (1.1)

Louwagie G, *et al. BMJ Open* 2022; 12:e056496. doi: 10.1136/bmjopen-2021-056496

Pulmonary only (ICD-10 A15)		281 (98.9)	272 (98.9)	553 (98.9)
**Number of pre-treatment smear results**				
One		197 (92.1)	169 (87.6)	366 (89.9)
Two		17 (7.9)	24 (12.4)	41 (10.1)
**Smear result**	N =	196	180	376
**At least one positive smear result**		111 (56.63)	109 (60.56)	220 (58.51)
**Number of Gene XPert results recorded**				
One		225 (97.8)	210 (97.2)	435 (97.5)
Two		5 (2.2)	6 (2.8)	11 (2.5)
**Gene XPert result**	N =	211	204	415
**At least one positive Gene XPert result**		184 (87.2)	178 (87.25)	362 (87.23)
**Number of culture results recorded on the TB Treatment record**				
One		54 (94.7)	42 (97.7)	96 (96.0)
Two		3 (5.3)	1 (2.3)	4 (4.0)
**Culture result**	N =	41	32	73
**At least one positive culture result**		20 (48.78)	15 (46.88)	35 (47.95)
**Co-morbidities**				
Hypertension		19 (6.93)	11 (4.1)	30 (5.54)
Diabetes		5 (1.84)	4 (1.49)	9 (1.66)
Epilepsy		3 (1.09)	4 (1.49)	7 (1.29)
Mental illness		3 (1.09)	0 (0)	3 (0.55)
Liver disease		1 (0.36)	1 (0.38)	2 (0.37)
Renal insufficiency		1 (0.36)	1 (0.38)	2 (0.37)
Allergies		2 (0.76)	0 (0)	2 (0.38)

Other		1 (0.36)	1 (0.38)	2 (0.37)
**Total Number of comorbidities**				
0		265 (96.0)	260 (96.7)	525 (96.3)
1		10 (3.6)	8 (3.0)	18 (3.3)
2		0 (0.0)	1 (0.4)	1 (0.2)
5		1 (0.4)	0 (0.0)	1 (0.2)
**HIV status**				
Negative		118 (40.7)	125 (44.2)	243 (42.4)
Positive		163 (56.2)	142 (50.2)	305 (53.2)
Unknown		9 (3.1)	16 (5.7)	25 (4.4)
**HIV positive patients**				
**CD4 Count: mean(SD)**				
**Using Cotrimoxazole**		104 (63.8)	100(67.11)	204 (65.38)
**Using anti-retroviral**		139 (85.28)	118 (79.19)	257 (82.37)

TABLE 3: Descriptive statistics for smoking history, alcohol history and depression score at baseline by study arm. Frequencies and (percentages) are presented unless otherwise stated.

TABLE 3: Descriptive statistics for smoking history, alcohol history and depression score at baseline and as analysed by study arm. Frequencies and (percentages) are presented unless otherwise stated.			
	Baseline		
	Control	Intervention	Total
	Arm 1	Arm 2	Total
**In the past month, smoked tobacco**			
Not at all	110 (37.8)	92 (32.5)	202 (35.2)
Daily	149 (51.2)	149 (52.7)	298 (51.9)
Less than Daily	32 (11.0)	42 (14.8)	74 (12.9)
**Had a drink in the past 12-months**	208 (71.5)	223 (78.8)	431 (75.1)
**AUDIT Score (males):** mean (SD) [max :19] [min = 8 if drinkers only]	12.27 (3.98)	13.02 (3.78)	12.66 (3.89)
**AUDIT Score (females):** mean (SD) [max :19] [min = 7 if drinkers only]	11.32 (4.02)	10.98 (4.02)	11.15 (4)
**Drinking and Smoking Combined (Constructed)**			
Drinkers Only	110 (37.8)	92 (32.5)	202 (35.2)
Smokers Only	83 (28.5)	60 (21.2)	143 (24.9)
Smokers and Drinkers	98 (33.7)	131 (46.3)	229 (39.9)

	Arm 1	Arm 2	Total
**Smoking History (current smokers only)**	181	191	372
On the days that you smoke, how soon after you wake up do you have your first cigarette?			
After 60 minutes	30 (16.6)	28 (14.7)	58 (15.6)
31-60 minutes	24 (13.3)	16 (8.4)	40 (10.8)
6- 30 minutes	60 (33.1)	65 (34.0)	125 (33.6)
Within 5 minutes	67 (37.0)	82 (42.9)	149 (40.1)
Duration of smoking in **months**: mean (SD)	212.09 (134.03)	224.93 (127.82)	218.68 (130.86)
Duration of smoking in **months**: median (IQR)	186 (110, 282)	206 (135, 294)	200.5 (123, 287)
Age started smoking in **years**: mean (SD)	19.2 (6.3)	19.3 (6.3)	19.3 (6.3)
Age started smoking in **years**: median (IQR)	18 (15-20)	18 (16-21)	18 (15.5-20.5)
**Form of tobacco used**			
**Manufactured cigarettes (Yes)**	**166 (91.7)**	**179 (93.7)**	**345 (92.7)**
Number of days in the past 7days you smoked: mean (SD)	5.3 (2.65)	5.53 (2.33)	5.42 (2.49)
Average number of cigarettes smoked daily: mean (SD)	6.18 (6.43)	6.48 (8.21)	6.34 (7.39)
**Hand-rolled cigarettes (Yes)**	**14 (7.7)**	**21 (11.0)**	**35 (9.4)**
Number of days in the past 7days you smoked: mean (SD)	4.71 (2.84)	3.71 (2.95)	4.11 (2.91)
Average number of handrolled cigarettes smoked daily: mean (SD)	3.71 (3.97)	3.81 (2.82)	3.77 (3.27)
**Pipe (Yes)**	**4 (2.2)**	**2 (1.0)**	**6 (1.6)**
Number of days in the past 7days you smoked: median (IQR)	1 (0, 2.5)	4.5 (2, 7)	2 (0, 3)
Average number of daily sessions: median (IQR)	1 (0, 3.5)	3 (1, 5)	1.5 (0, 5)
Length of one session (on average) in **minutes**: median (IQR)	90 (60, 107.5)	60 (30, 90)	90 (30, 90)
**Cigars, cheroots or cigarillos (Yes)**	1 (0.55)	0 (0)	1(0.27)

**Water pipe (Yes)**	3 (1.7)	2 (1.0)	5 (1.3)
Other	10 (5.5)	4 (2.1)	14 (3.8)
**Heaviness of smoking index >= 4**	134(74.03)	158(82.72)	292(78.49)
**Smoking inside your home restrictions**			
Total: Not allowed	96 (53.0)	108 (56.5)	204 (54.8)
Some rules: where/when it is allowed	61 (33.7)	58 (30.4)	119 (32.0)
No rules	24 (13.3)	25 (13.1)	49 (13.2)
**Attempts to quit smoking (current smokers only) (Yes)**			
Ever attempted to quit in the past			
Yes	52 (28.7)	64 (33.5)	116 (31.2)
No	129 (71.3)	127 (66.5)	256 (68.8)
Number of attempts to quit: mean (SD)	2.46 (2.98)	2.64 (1.62)	2.56 (2.32)
Time elapsed since attempt to quit last time in months: mean (SD)	36.65 (94.62)	25.13 (37.91)	30.29 (69.22)
Longest duration abstinent in previous quit attempts: mean (SD)	6.15 (13.48)	4.22 (8.9)	5.09 (11.18)
Likelihood to TRY TO QUIT smoking completely and permanently in the next three months			
definitely will not	6 (3.3)	5 (2.6)	11 (3.0)
probably will not	10 (5.5)	12 (6.3)	22 (5.9)
probably will	104 (57.5)	109 (57.1)	213 (57.3)
definitely will	61 (33.7)	65 (34.0)	126 (33.9)
	Control	Intervention	

Louwagie G, *et al. BMJ Open* 2022; 12:e056496. doi: 10.1136/bmjopen-2021-056496

	Arm 1	Arm 2	Total
Likelihood that I WILL QUIT smoking completely and permanently in the next three months			
definitely will not	6 (3.3)	5 (2.6)	11 (3.0)
probably will not	11 (6.1)	13 (6.8)	24 (6.5)
probably will	103 (56.9)	104 (54.5)	207 (55.6)
definitely will	61 (33.7)	69 (36.1)	130 (34.9)
**Ever used any methods to help you stop smoking tobacco in the past 3 months? (**Yes)	23 (12.7)	16 (8.4)	39 (10.5)
**Out of your pocket spend (in Rands) on methods to help you stop smoking in the past 3 months: mean (SD)**	134.87 (237.58)	139.19 (155.07)	136.64 (205.36)
**Average spend per week on cigarettes over the past 3 months: mean (SD)**	168.77 (178.15)	180.29 (185.27)	174.58 (181.61)
**Smokeless tobacco use (all participants)**			
**In the past month, have you used smokeless tobacco (Snuff) on a daily basis**			
Not at all	275 (94.5)	275 (97.2)	550 (95.8)
Daily	12 (4.1)	6 (2.1)	18 (3.1)
Less than Daily	4 (1.4)	2 (0.7)	6 (1.0)
Duration of using ST in months: mean(SD)	113.69 (112.7)	152 (91.16)	126.46 (105.61)
Age started using ST in **years**: mean (SD)	27.56 (10.57)	25.88 (13.43)	27 (11.33)
**Form of ST used (for SLT users)**			
Snuff (by mouth)	2 (12.5)	0 (0.0)	2 (8.3)
Snuff (by nose)	11 (68.8)	5 (62.5)	16 (66.7)
Chewing tobacco leaves	0(0)	0(0)	0(0)
Other	1 (6.3)	0 (0.0)	1 (4.2)
	Arm 1	Arm 2	Total

	Control	Intervention	
**Tobacco smoke exposure:** number of days in the past 30 days, you were in a place where someone smoked close to you (all participants?)			
**Home**			
7 days or less	233 (80.1)	229 (80.9)	462 (80.5)
More than 7 days	58 (19.9)	54 (19.1)	112 (19.5)
**Workplace** (Missing 315; probably Not Applicable)			
7 days or less	84 (65.6)	89 (67.9)	173 (66.8)
More than 7 days	44 (34.4)	42 (32.1)	86 (33.2)
**Cafes/restaurants**			
7 days or less	230 (79.0)	234 (82.7)	464 (80.8)
More than 7 days	61 (21.0)	49 (17.3)	110 (19.2)
**Shebeens, bars or clubs**			
7 days or less	219 (75.3)	213 (75.3)	432 (75.3)
More than 7 days	72 (24.7)	70 (24.7)	142 (24.7)
**Bus/train/taxi/ vehicle**			
7 days or less	229 (78.7)	219 (77.4)	448 (78.0)
More than 7 days	62 (21.3)	64 (22.6)	126 (22.0)
**Shops/shopping mall**			
7 days or less	235 (80.8)	226 (79.9)	461 (80.3)
More than 7 days	56 (19.2)	57 (20.1)	113 (19.7)
	Arm 1	Arm 2	Total

**Had a drink in the past 12-months**	208 (71.5)	223 (78.8)	431 (75.1)
**Help to stop drinking (drinkers only)**			
Ever used any methods to stop drinking alcohol in the past 3 months	21 (11.2)	22 (10.7)	43 (10.9)
Average spend in Rands per week on alcohol over the past 3 months: mean (SD)	363.76 (531.56)	337.03 (387.94)	349.79 (461.66)
**Depression: CESD 10** how often you felt or behaved this way during the past week			
I was bothered by things that usually don't bother me.			
Rarely or None of the Time (Less than 1 day)	180 (61.9)	159 (56.2)	339 (59.1)
Some or a Little of the Time (1-2 days)	71 (24.4)	78 (27.6)	149 (26.0)
Occasionally or a Moderate Amount of the Time (3-4 days)	24 (8.2)	31 (11.0)	55 (9.6)
Most or All of the Time (5-7 days)	16 (5.5)	15 (5.3)	31 (5.4)
I had trouble keeping my mind on what I was doing.			
Rarely or None of the Time (Less than 1 day)	150 (51.5)	136 (48.1)	286 (49.8)
Some or a Little of the Time (1-2 days)	93 (32.0)	85 (30.0)	178 (31.0)
Occasionally or a Moderate Amount of the Time (3-4 days)	31 (10.7)	38 (13.4)	69 (12.0)
Most or All of the Time (5-7 days)	17 (5.8)	24 (8.5)	41 (7.1)
I felt depressed.			
Rarely or None of the Time (Less than 1 day)	152 (52.2)	171 (60.4)	323 (56.3)
Some or a Little of the Time (1-2 days)	94 (32.3)	70 (24.7)	164 (28.6)
Occasionally or a Moderate Amount of the Time (3-4 days)	37 (12.7)	30 (10.6)	67 (11.7)
Most or All of the Time (5-7 days)	8 (2.7)	12 (4.2)	20 (3.5)
I felt that everything I did was an effort.			

Rarely or None of the Time (Less than 1 day)	140 (48.1)	117 (41.3)	257 (44.8)
Some or a Little of the Time (1-2 days)	77 (26.5)	87 (30.7)	164 (28.6)
Occasionally or a Moderate Amount of the Time (3-4 days)	38 (13.1)	43 (15.2)	81 (14.1)
Most or All of the Time (5-7 days)	36 (12.4)	36 (12.7)	72 (12.5)
I felt hopeful about the future.			
Rarely or None of the Time (Less than 1 day)	85 (29.2)	84 (29.7)	169 (29.4)
Some or a Little of the Time (1-2 days)	78 (26.8)	70 (24.7)	148 (25.8)
Occasionally or a Moderate Amount of the Time (3-4 days)	53 (18.2)	56 (19.8)	109 (19.0)
Most or All of the Time (5-7 days)	75 (25.8)	73 (25.8)	148 (25.8)
I felt fearful.			
Rarely or None of the Time (Less than 1 day)	175 (60.1)	159 (56.2)	334 (58.2)
Some or a Little of the Time (1-2 days)	82 (28.2)	79 (27.9)	161 (28.0)
Occasionally or a Moderate Amount of the Time (3-4 days)	25 (8.6)	32 (11.3)	57 (9.9)
Most or All of the Time (5-7 days)	9 (3.1)	13 (4.6)	22 (3.8)
My sleep was restless.			
Rarely or None of the Time (Less than 1 day)	142 (48.8)	135 (47.7)	277 (48.3)
Some or a Little of the Time (1-2 days)	95 (32.6)	89 (31.4)	184 (32.1)
Occasionally or a Moderate Amount of the Time (3-4 days)	31 (10.7)	33 (11.7)	64 (11.1)
Most or All of the Time (5-7 days)	23 (7.9)	26 (9.2)	49 (8.5)
I was happy.			
Rarely or None of the Time (Less than 1 day)	85 (29.2)	74 (26.1)	159 (27.7)
Some or a Little of the Time (1-2 days)	70 (24.1)	65 (23.0)	135 (23.5)
Occasionally or a Moderate Amount of the Time (3-4 days)	73 (25.1)	86 (30.4)	159 (27.7)
Most or All of the Time (5-7 days)	63 (21.6)	58 (20.5)	121 (21.1)
I felt lonely.			

Louwagie G, *et al. BMJ Open* 2022; 12:e056496. doi: 10.1136/bmjopen-2021-056496

Rarely or None of the Time (Less than 1 day)	158 (54.3)	161 (56.9)	319 (55.6)
Some or a Little of the Time (1-2 days)	89 (30.6)	83 (29.3)	172 (30.0)
Occasionally or a Moderate Amount of the Time (3-4 days)	23 (7.9)	26 (9.2)	49 (8.5)
Most or All of the Time (5-7 days)	21 (7.2)	13 (4.6)	34 (5.9)
I could not get “going”.			
Rarely or None of the Time (Less than 1 day)	168 (57.7)	174 (61.5)	342 (59.6)
Some or a Little of the Time (1-2 days)	78 (26.8)	69 (24.4)	147 (25.6)
Occasionally or a Moderate Amount of the Time (3-4 days)	35 (12.0)	29 (10.2)	64 (11.1)
Most or All of the Time (5-7 days)	10 (3.4)	11 (3.9)	21 (3.7)
Total CESD 10: mean (SD)	8.44 (4.38)	8.74 (4.8)	8.59 (4.59)

Table 4: Descriptive statistics for primary and secondary outcomes by study arm at baseline (where available), 3 month (where available) and 6 month. Frequencies and (percentages) are presented unless otherwise stated

TABLE 4: DESCRIPTIVE STATISTICS FOR PRIMARY AND SECONDARY OUTCOMES BY STUDY ARM AT BASELINE (WHERE AVAILABLE), 3 MONTH (WHERE AVAILABLE) AND 6 MONTH. FREQUENCIES AND (PERCENTAGES) ARE PRESENTED UNLESS OTHERWISE STATED.

	Baseline			Follow-up 3-month			Follow-up 6-month		
	Control	Intervention	Total	Control	Intervention	Total	Control	Intervention	Total
	Arm1	Arm2	Total	Arm1	Arm2	Total	Arm1	Arm2	Total
**TB treatment status detailed**									
Cured							108 (37.11)	105 (37.1)	213 (37.11)
Treatment completed							96 (33.0)	87 (30.74)	183 (31.88)
Treatment default							15 (5.2)	29 (10.25)	44 (7.67)
Treatment failure							5 (1.7)	2 (0.7)	7 (1.2)
Acquired drug resistance							1 (0.34)	4 (1.41)	5 (0.87)
Died							11 (3.78)	15 (5.30)	26 (4.53)
Transfer out							8 (2.75)	11 (3.89)	19 (3.31)
Unknown							42 (14.43)	25 (8.83)	67 (11.67)
Missing							5 (1.72)	5 (1.77)	10 (1.74)
**TB treatment status binary (Primary outcome**)**									
Not Successful							87 (29.9)	91 (32.16)	178 (31.01)
Successful							204 (70.10)	192 (67.84)	396 (68.99)
**At least one positive smear result**	85 (53.46)	96 (61.15)	181 (57.28)						
**At least one positive Gene XPert result**	184 (87.20)	178 (87.25)	362 (87.23)						
**At least one positive culture result**	11 (57.89)	10 (66.67)	21 (61.76)						

Louwagie G, et al. BMJ Open 2022; 12:e056496. doi: 10.1136/bmjopen-2021-056496

**Sputum smear, Gene XPert, or culture result**									
Negative	29 (12.45)	32 (14.16)	61 (13.29)						
Positive	204 (87.55)	194 (85.84)	398 (86.71)						
**Conversion from positive to negative*** **									
Yes							83(39.9)	85(43.59)	168(41.69)
No							125(60.1)	110(56.41)	235(58.31)
**Continuous smoking abstinence among cigarette smokers at baseline**									
Yes				27(16.27)	20(11.17)	47(13.62)	12(7.23)	10(5.59)	22(6.38)
No				139(83.73)	159(88.83)	298(86.38)	154(92.77)	169(94.41)	323(93.62)
**Harmful or hazardous drinking[1]**									
**Had a drink in the past 12-months**	208 (71.5)	223 (78.8)	431 (75.1)						
AUDIT score: mean (SD)	12.03 (4)	12.53 (3.93)	12.29 (3.96)						
**Harmful or hazardous drinkers out of those who had a drink in the past 12-months at baseline (%)**	188(90.38)	206(92.38)	394(91.42)	141	130	271	112	127	239
**AUDIT Score (males):** mean (SD) [max :19; min = 8]*!	13.14 (3.31)	13.61 (3.29)	13.39 (3.31)	8.20(6.08)	9.08(4.97)	8.63(5.58)	9.21(6.58)	8.24(5.41)	8.69(5.99)
**AUDIT Score (females):** mean (SD) [max :19; min = 7]*!	11.73 (3.52)	11.55 (3.6)	11.64 (3.54)	8.5(6.52)	8.15(6.44)	8.33(6.44)	7.67(6.84)	9.97(6.79)	8.89(6.86)
AUDIT score: mean (SD)	12.76 (3.42)	13.12 (3.47)	12.94 (3.45)	8.28(6.18)	8.84(5.38)	8.55(5.81)	8.79(6.66)	8.70(5.83)	8.74(6.22)
Difference from baseline				-4.61 (6.26)	-4.07(5.33)	-4.35(5.83)	-4.25(6.56)	-4.17(6.61)	-4.21(6.57)

**Taking ART medication among** HIV Positive patients	[163 (56.2)]	[142 (50.2)]	[305 (53.2)]	[122]	[83]	[205]	[100]	[83]	[183]
Yes	139 (85.28)	115 (80.99)	254 (83.28)	91 (74.6)	58 (69.9)	149 (72.7)	80 (80.0)	74 (89.2)	154 (84.2)
**ART medication adherence**									
Optimal adherence				101(99.02)	64(98.46)	165(98.8)	75(97.4)	64(96.97)	139(97.2)
Suboptimal adherence				1(0.98)	1(1.54)	2(1.2)	2(2.6)	2(3.03)	4(2.8)
**TB medication adherence**									
Optimal adherence				181(92.35)	138(90.79)	319(91.67)	61(89.71)	59(90.77)	120(90.23)
Suboptimal adherence				15(7.65)	14(9.21)	29(8.33)	7(10.29)	6(9.23)	13(9.77)

[1] hazardous/harmful drinkers who are not alcohol dependent= AUDIT score ≥ 8 for men or ≥ 7 for women but < 20

**Primary Outcome: in the published protocol paper "This is a binary variable defined as either successful treatment (cured or treatment completed) or failed treatment, death, acquired drug resistance, loss to follow-up or 'default', or not outcome evaluated.

*** Conversion from positive to negative: this was based on having a cured treatment outcome among those who were positive at baseline.

*! Important distinction at baseline for eligibility purposes.

TABLE 5: Number and type of adverse events at month 2, 3, and 6 by centre and study arm (see other document)

TABLE 5: Number and type of adverse events at month 2, 3, and 6 by centre and study arm							
Centre		Month 2		Month 3		Month 6	
	TYPE	Intervention	Control	Intervention	Control	Intervention	Control

TABLE 6: Regression analysis results for the primary and secondary outcomes at 6 months. Estimates presented with corresponding 95% CI. Crude and adjusted estimates are provided.

TABLE 6: Regression analysis results for the primary and secondary outcomes at 6 months. Estimates presented with corresponding 95% CI. Crude and adjusted estimates are provided.

	Crude Odds Ratio (95% CI)*	P Value*	Adjusted Odds Ratio (95% CI)*	P Value*	
**Primary outcome**					
TB treatment status: Successful (**Ref: Not successful**)	0.9 (0.64,1.27)	0.548	0.86 (0.60,1.24)	0.421	
**Secondary outcomes**					
Sputum smear or culture result: converted from positive to negative (Ref: Not converted)	1.16 (0.83,1.63)	0.374	1.07‡ (0.76,1.51)	0.684	
Six-month continuous smoking abstinence among cigarette smokers at baseline (Ref: No)	0.76 (0.35,1.64)	0.482			
Taking ART medication among HIV positive patients[!!]	2.05 (0.80,5.27)	0.136			
TB medication adherence (Reference: Optimal)	0.89 (0.26,3.07)	0.849			
ART medication adherence (Reference: Optimal)	1.17 (0.14,9.94)	0.884			

Louwagie G, *et al. BMJ Open* 2022; 12:e056496. doi: 10.1136/bmjopen-2021-056496

	Study arm regression coefficient		Study arm regression coefficient		
**AUDIT for those who were harmful or hazardous drinkers at baseline**:**	-0.04 (-2,1.91)	0.966	0.02[!] (-1.55,1.6)	0.976	

* analyses accounted for potential clustering by centre.
† Number of participants whose outcome was treatment successful among the total number in the group.
‡ Adjusted for district, sex, and smoking/drinking status and HIV status at baseline
**Controlling for the AUDIT baseline values.
! Controlling for the AUDIT baseline values and adjusted for district, sex, and smoking/drinking status and HIV status at baseline
!! Adjusting for art status at baseline

BMJ Publishing Group Limited (BMJ) disclaims all liability and responsibility arising from any reliance placed on this supplemental material which has been supplied by the author(s)

TABLE 7: Regression analysis results for secondary outcomes measured at 3-months. Estimates presented with corresponding 95% CI. Crude and adjusted estimates are provided.

TABLE 7: Regression analysis results for secondary outcomes measured at 3-months. Estimates presented with corresponding 95% CI. Crude and adjusted estimates are provided.

	Crude Odds Ratio (95% CI)	P Value	Adjusted Odds Ratio (95% CI)	P Value	
**Secondary outcome**					
3-months continuous smoking abstinence among cigarette smokers at baseline	0.65 (0.37,1.14)	0.135			
Taking ART medication among HIV positive patients!!	0.79 (0.38,1.65)	0.53	0.74‡ (0.35,1.58)	0.443	
TB medication adherence (Reference: Optimal)	1.22 (0.52,2.87)	0.641	[redacted]		
ART medication adherence (Reference: Optimal)	1.58 (0.10,26.12)	0.750	[redacted]		
	Study arm regression coefficient		Adjusted estimates		
AUDIT for those who were harmful or hazardous drinkers at baseline**:	0.55 (-1.01,2.11)	0.474	0.74 (-0.62,2.1)	0.273	

* analyses accounted for clustering.
‡ Adjusted for district, sex, and smoking/drinking status and HIV status at baseline
**Controlling for the AUDIT baseline values.
! Controlling for the AUDIT baseline values and adjusted for district, sex, and smoking/drinking status and HIV status at baseline
!! Adjusting for art status at baseline

Louwagie G, *et al. BMJ Open* 2022; 12:e056496. doi: 10.1136/bmjopen-2021-056496

Additional tables
SMS-fidelity

**Variable**	**Intervention (N= ….) (=no. of participants allocated to intervention group, for example 245)**	**Control (N=…) (=no. participants allocated to control group, for example 248)**
No. of participants who received ALL due IMB messages INDEPENDENT OF WHETHER they completed MI 1 (i.o.w this is ITT analysis)	For example 120/245 (49 %)	For example 2/248 (…%)
Completion of first MI and initiation of SMS-sequence		
Completed first MI	n/N (%) (=No. who completed first MI in intervention arm/ no. participants allocated to intervention arm [%] For example 170/245 [69%])	n/N (%) (=No. who completed first MI in control/ no. participants allocated to control [%] For example 2/248)
No. of participants who received ALL due messages after receipt of MI1 and SMS sequence was generated (this is a type of Per Protocol analysis:	For example 120/170 (71%)	2/2

denominator limited to those who had MI1)				
SMS delivery for participants for whom the SMS-sequence was initiated (after receipt of first MI)				
	Mean (SD)	Median (IQR) Range	Mean (SD)	Median (IQR)
Average no. adherence messages received per participant (n=...) (n = the no. of participants who completed first MI, in this example 170)	For example 8 (3.4)	9 (7-10)<br><br>Range 0-10	Expected to be 0 or close to it	Expected to be 0 or close to it
Average no. tobacco-related messages received (n=...) (n=no of participants who completed first MI AND were current tobacco users at baseline, for example 90)	For example 7 (3.4)	7 (6-7)<br><br>Range 0-7	Expected to be 0 or close to it	Expected to be 0 or close to it
Average no. alcohol related messages received (n=...) (n=no. participants who completed first MI	For example 7 (3.4)	7 (6-7)<br><br>Range 0-7	Expected to be 0 or close to it	Expected to be 0 or close to it

AND were drinkers at baseline=....for example 100)				
Average no. IMB messages received (n= total no who completed first MI)	For example 16 (4.5)	15 (10-20)<br><br>Range 0-24	Expected to be 0 or close to it	Expected to be 0 or close to it

Other variables not yet analysed:

CESD at 3 and 6-month FU
Other smoking related questions at 3 and 6 month (quit intentions etc) but also SLT use at 3 and 6 month. (important because participants may have switched from tobacco smoking to SLT)
Other non-HE questions not yet analysed

13. Additional descriptive Statistics

TABLE 8: Additional Descriptive statistics for characteristics at baseline by study arm. Frequencies and (percentages) are presented unless otherwise stated

		Baseline		
	N1/N2	Intervention	Control	Total
		Arm 1	Arm 2	
TYPE OF MINE WORK	46/45			91
Coal		7 (15.2)	3 (6.7)	10 (11.0)
Diamond		3 (6.5)	1 (2.2)	4 (4.4)
Gold		13 (28.3)	17 (37.8)	30 (33.0)
Platinum and palladium		24 (52.2)	17 (37.8)	41 (45.1)
Chromium		11 (23.9)	12 (26.7)	23 (25.3)
Uranium		1 (2.2)	1 (2.2)	2 (2.2)
Manganese		0(0)	0(0)	0(0)
Other		0(0)	0(0)	0(0)
Total Types of Mines worked in				
1	73			
2	12			
3	3			
4	1			

Louwagie G, et al. BMJ Open 2022; 12:e056496. doi: 10.1136/bmjopen-2021-056496

Exact Distribution for number smoked in the past seven days				
0		24 (14.5)	16 (8.9)	40 (11.6)
1		4 (2.4)	4 (2.2)	8 (2.3)
2		4 (2.4)	3 (1.7)	7 (2.0)
3		5 (3.0)	13 (7.3)	18 (5.2)
4		13 (7.8)	8 (4.5)	21 (6.1)
5		5 (3.0)	17 (9.5)	22 (6.4)
6		1 (0.6)	3 (1.7)	4 (1.2)
7		110 (66.3)	115 (64.2)	225 (65.2)
new_a_positive_TB				
0		29 (11.98)	33 (14.35)	62 (13.14)
1		122 (50.41)	104 (45.22)	226 (47.88)
2		80 (33.06)	81 (35.22)	161 (34.11)
3		11 (4.55)	12 (5.22)	23 (4.87)

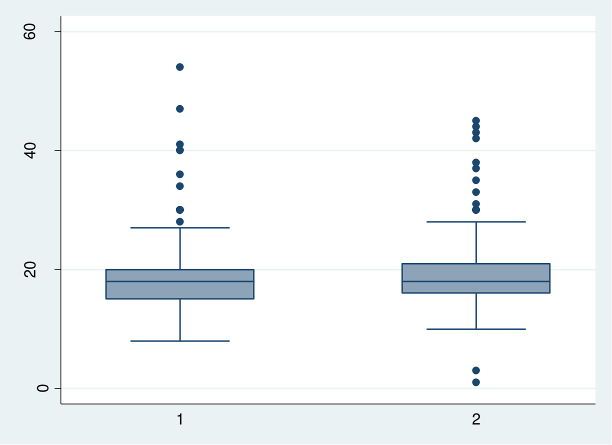

Age started smoking

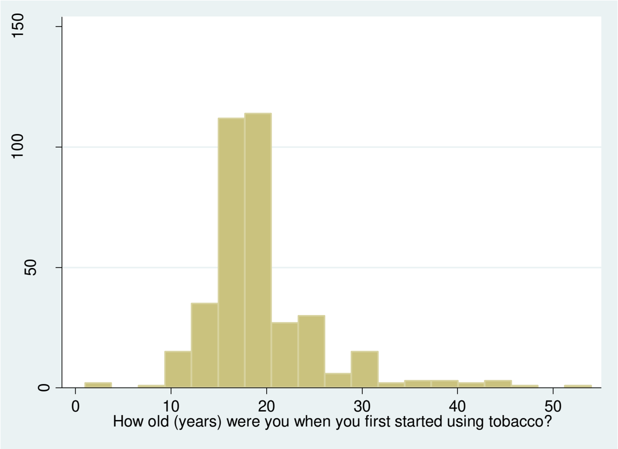

Supplement: Supplementary data [file bmjopen-2021-056496supp002.pdf]
