# Supplementary material for: Effect of a brief motivational interview and text message intervention targeting tobacco smoking, alcohol use and medication adherence to improve tuberculosis treatment outcomes in adult patients with tuberculosis: a multicentre, randomised controlled trial of the ProLife programme in South Africa
Source: BMJ Open. 2022 Feb 14;12(2):e056496. doi: 10.1136/bmjopen-2021-056496 (PMC8845202; doi:10.1136/bmjopen-2021-056496)
Supplement: Author's manuscript [file bmjopen-2021-056496.draft_revisions.pdf]

BMJ Open is committed to open peer review. As part of this commitment we make the peer review history of every article we publish publicly available.

When an article is published we post the peer reviewers' comments and the authors' responses online. We also post the versions of the paper that were used during peer review. These are the versions that the peer review comments apply to.

The versions of the paper that follow are the versions that were submitted during the peer review process. They are not the versions of record or the final published versions. They should not be cited or distributed as the published version of this manuscript.

BMJ Open is an open access journal and the full, final, typeset and author-corrected version of record of the manuscript is available on our site with no access controls, subscription charges or pay-per-view fees (http://bmjopen.bmj.com).

If you have any questions on BMJ Open's open peer review process please email info.bmjopen@bmj.com

BMJ Open

A brief motivational intervention targeting tobacco smoking, alcohol and medication adherence to improve TB treatment outcomes in adult TB patients: A multi-centre, randomised controlled trial of the ProLife programme in South Africa

Journal:	*BMJ Open*
Manuscript ID	bmjopen-2021-056496
Article Type:	Original research
Date Submitted by the Author:	20-Aug-2021
Complete List of Authors:	Louwagie, Goedele; Sefako Makgatho Health Sciences University, Research, Postgraduate Studies and Innovation; University of Pretoria, School of Health Systems and Public Health<br>Kanaan, Mona; University of York, Department of Health Sciences<br>Morojele, Neo; University of Johannesburg, Department of Psychology; Medical Research Council of South Africa, Alcohol, Tobacco and Other Drug Research Unit<br>Van Zyl, Andre; Sefako Makgatho Health Sciences University<br>Moriarty, Andrew; University of York, Hull York Medical School<br>Li, Jinshuo; University of York, Department of Health Sciences<br>Siddiqi, Kamran; University of York, Department of Health Sciences and the Hull York Medical School<br>Turner, Astrid; University of Pretoria, School of Health Systems and Public Health<br>Mdege, Noreen; University of York, Department of Health Sciences<br>Omole, Olufemi; University of the Witwatersrand, Department of Family Medicine<br>Tumbo, John; Sefako Makgatho Health Sciences University, Department of Family Medicine<br>Bachmann, Max; University of East Anglia, Norwich Medical School<br>Parrott, Steve; University of York, Department of Health Sciences<br>Ayo-Yusuf, Olalekan; Sefako Makgatho Health Sciences University, Africa Centre for Tobacco Industry Monitoring & Policy Research; University of Pretoria, School of Health Systems and Public Health
Keywords:	Tuberculosis < INFECTIOUS DISEASES, Public health < INFECTIOUS DISEASES, HIV & AIDS < INFECTIOUS DISEASES, Substance misuse < PSYCHIATRY, Health economics < HEALTH SERVICES ADMINISTRATION & MANAGEMENT, Organisation of health services < HEALTH SERVICES ADMINISTRATION & MANAGEMENT

SCHOLARONE™
Manuscripts

I, the Submitting Author has the right to grant and does grant on behalf of all authors of the Work (as defined in the below author licence), an exclusive licence and/or a non-exclusive licence for contributions from authors who are: i) UK Crown employees; ii) where BMJ has agreed a CC-BY licence shall apply, and/or iii) in accordance with the terms applicable for US Federal Government officers or employees acting as part of their official duties; on a worldwide, perpetual, irrevocable, royalty-free basis to BMJ Publishing Group Ltd ("BMJ") its licensees and where the relevant Journal is co-owned by BMJ to the co-owners of the Journal, to publish the Work in this journal and any other BMJ products and to exploit all rights, as set out in our licence.

The Submitting Author accepts and understands that any supply made under these terms is made by BMJ to the Submitting Author unless you are acting as an employee on behalf of your employer or a postgraduate student of an affiliated institution which is paying any applicable article publishing charge ("APC") for Open Access articles. Where the Submitting Author wishes to make the Work available on an Open Access basis (and intends to pay the relevant APC), the terms of reuse of such Open Access shall be governed by a Creative Commons licence – details of these licences and which Creative Commons licence will apply to this Work are set out in our licence referred to above.

Other than as permitted in any relevant BMJ Author's Self Archiving Policies, I confirm this Work has not been accepted for publication elsewhere, is not being considered for publication elsewhere and does not duplicate material already published. I confirm all authors consent to publication of this Work and authorise the granting of this licence.

For peer review only

A brief motivational intervention targeting tobacco smoking, alcohol and medication adherence to improve TB treatment outcomes in adult TB patients: A multi-centre, randomised controlled trial of the ProLife programme in South Africa

Goedele Maria Louwagie[1, 2], Mona Kanaan[3], Neo Keitumetse Morojele[4, 5], André van Zyl[1], Andrew Stephen Moriarty[3], Jinshuo Li[3], Kamran Siddiqi[3], Astrid Turner[2], Noreen Dadirai Mdege[3], Olufemi Babatunde Omole[6], John Tumbo[1], Max Oscar Bachmann[7], Steve Parrott[3], Olalekan Ayo-Yusuf[1, 2]

Correspondence to Goedele Maria Louwagie; goedele.louwagie@up.ac.za

[1]Goedele Maria Louwagie, Sefako Makgatho Health Sciences University and [2]School of Health Systems and Public Health, University of Pretoria , RSA; goedele.louwagie@up.ac.za

[3]Mona Kanaan, Department of Health Sciences, University of York, UK; mona.kanaan@york.ac.uk

[4]Neo Keitumetse Morojele, Department of Psychology, University of Johannesburg and [5]Alcohol, Tobacco and Other Drug Research Unit, South African Medical Research Council, RSA; nmorojele@uj.ac.za

[1]André van Zyl, Sefako Makgatho Health Sciences University, RSA; drevanzyl@gmail.com

[3]Andrew Stephen Moriarty, Department of Health Sciences and the Hull York Medical School, University of York, UK; andrew.moriarty@york.ac.uk

[3]Jinshuo Li, Department of Health Sciences, University of York, UK; jinshuo.li@york.ac.uk

[3]Kamran Siddiqi, Department of Health Sciences and the Hull York Medical School, University of York, UK; kamran.siddiqi@york.ac.uk

[2]Astrid Turner, School of Health Systems and Public Health, University of Pretoria, RSA; astrid.turner@up.ac.za

[3]Noreen Dadirai Mdege, Department of Health Sciences, University of York, UK; noreen.mdege@york.ac.uk

[6]Olufemi Babatunde Omole, Department of Family Medicine, University of Witwatersrand, RSA; Olufemi.Omole@wits.ac.za

[1]John Tumbo, Department of Family Medicine, Sefako Makgatho Health Sciences University, RSA; tumbo@lantic.net

[7]Max Oscar Bachmann, Norwich Medical School, University of East Anglia, UK; m.bachmann@uea.ac.uk

[3]Steve Parrott, Department of Health Sciences, University of York, UK; steve.parrott@york.ac.uk

For peer review only - http://bmjopen.bmj.com/site/about/guidelines.xhtml

[1]Olalekan Ayo-Yusuf, Africa Centre for Tobacco Industry Monitoring & Policy Research, Sefako Makgatho Health Sciences University, and [2]School of Health Systems and Public Health, University of Pretoria, RSA; lekan.ayo-yusuf@smu.ac.za

ABSTRACT

Introduction South Africa has one of the highest TB burdens in the world. Smoking, harmful alcohol use and poor medication adherence may adversely affect TB treatment outcomes. We aimed to investigate the effectiveness of a complex behavioural intervention, ProLife, on TB treatment success (primary outcome) and medication adherence, alcohol use and tobacco smoking.

Methods The intervention, delivered by lay health workers, consisted of motivational interviewing (MI), augmented with subsequent Short Message Service (SMS), targeting medication adherence, alcohol use and tobacco smoking. This was a multi-centre, individual, randomised controlled trial where participants were assigned (1:1) to the ProLife intervention or usual care. Participants were adults with drug-sensitive pulmonary TB from 27 clinics who smoked or reported harmful/hazardous alcohol use. Questionnaires were administered at baseline, 3- and 6-months. Clinical information and TB treatment outcomes were obtained from patients' records.

Results Between 15 November 2018 and 31 August 2019, 574 participants were randomised to receive either the ProLife intervention (n=283) or usual care (n=291). TB treatment success rates did not differ significantly between intervention (67.8%) and control (70.1%) (OR=0.9 (95% CI: 0.64,1.27)). There was also no evidence of an effect on continuous smoking abstinence (OR=0.65 (95% CI: 0.37,1.14) at 3-months; OR=0.76 (95% CI: 0.35,1.63) at 6-months) or TB medication adherence (OR=1.22 (95%CI: 0.52,2.87) at 3-months; OR=0.89 (95%CI: 0.26,3.07) at 6-months). The mean Alcohol Use Disorder Identification Test score was higher by 0.55 (95% CI: -1.01,2.11) in the intervention arm at 3-months and lower by 0.04 points (95% CI: -2,1.91) at 6-months; and adjusting for baseline values. Adherence to MI counselling principles was fair. The mean cost of the intervention was $180.02 ($0.42) per participant.

Conclusion The ProLife intervention was not effective. Simultaneous targeting of multiple health risk behaviours with MI and SMS may not be an effective approach to improve TB outcomes.

Trial registration: ISRCTN62728852, Registered on 13th April 2018.

For peer review only - http://bmjopen.bmj.com/site/about/guidelines.xhtml

Key words: Tuberculosis, smoking, alcohol, motivational interviewing, mHealth, anti-retroviral therapy, adherence

STRENGTHS AND LIMITATIONS OF THIS STUDY

- The use of motivational interviewing combined with Short Text Messaging to address the effect of multiple risk behaviours (smoking, drinking and poor adherence) on TB treatment outcomes is a novel and much needed intervention.
- Our study design was strong: this was a multi-site individually randomised controlled trial with a large sample size and a high follow up rate for the primary outcome.
- We used validated measurement tools; furthermore data analysis and primary outcome assessment were blinded, thereby limiting measurement bias.
- The intervention uptake was quite low in particular for the second and third motivational interviewing counselling session.
- The intervention aimed at tobacco smoking, hazardous/harmful alcohol use and medication adherence may have been too complex for both interventionists and participants.

For peer review only - http://bmjopen.bmj.com/site/about/guidelines.xhtml

INTRODUCTION

Tuberculosis (TB) is among the most common chronic infectious diseases in the world today. In 2019, 1.4 million deaths worldwide were attributed to TB, and the majority of these occurred in low-income and middle-income countries (LMICs).(1) South Africa has one of the highest TB burdens of the 30 high TB burden countries. It also has a high prevalence of human immunodeficiency virus (HIV) co-infection in patients with TB and a relatively high mortality in these co-infected patients.(1) Studies of interventions to advance the goal of ending the TB epidemic and improving treatment outcomes are research priorities in South Africa and other LMICs.(2)

Mortality and morbidity from TB is strongly associated with health risk behaviours, particularly smoking and hazardous or harmful alcohol use, both of which are prevalent and often co-occur in TB patients.(3–10) Strategies are also required to improve TB medication adherence in patients with TB and adherence to TB medication and anti-retroviral therapy (ART) in TB-HIV co-infected patients, both of which may be negatively influenced by excessive alcohol use.(1) There is very limited research on how to concurrently tackle these three risk behaviours - namely smoking, harmful alcohol use and poor medication adherence - in patients with TB, particularly in LMICs.

Motivational Interviewing (MI) is a counselling technique known to facilitate behaviour change,(11) and has been shown to effectively support reduced drinking, tobacco cessation and TB treatment and/or antiretroviral therapy (ART) medication adherence.(7,12,13) MI interventions can be effectively delivered by lay health workers (LHWs).(14) The more widespread use of LHWs and the increased use of mobile health (mHealth) digital technologies represent promising ways to increase the scalability of MI interventions. Indeed, the World Health Organisation (WHO) has called for researchers to capitalise on advances in mobile phone technology, network coverage and the increased use of common and widely available digital technologies (including the mobile phone short message service (SMS)) to improve TB care.(15) There is robust evidence that mHealth technologies can have modest beneficial effects on a range of health outcomes, including medication adherence.(16) An SMS intervention in co-infected TB/HIV patients demonstrated effectiveness in increasing adherence to ART,(17) although the existing evidence is not of sufficient quality to know if this consistently applies to medication adherence in TB patients.(18)

A limitation of existing MI and mHealth interventions is that they have been studied in the context of modifying a single lifestyle factor. Integrated interventions are likely to be better accepted and more effective than multiple interventions targeting different health risk factors.(19,20) In the case of TB, there is a need for an intervention that has the flexibility to target multiple lifestyle factors as

For peer review only - http://bmjopen.bmj.com/site/about/guidelines.xhtml

appropriate and in line with patient preferences. This could be achieved through increased integration of TB and non-communicable disease services.(21)

Recent re-engineering of primary health care in South Africa has seen the introduction of municipal ward-based primary healthcare outreach teams of community health workers (CHWS). CHWs work in an integrated, team-based manner, supported by nurses, and take responsibility for health education and promotion, counselling and support for a range of health conditions.(22,23) Task shifting in this context has been shown to improve population health in LMICs (24) and these teams can be trained and supported to take responsibility for TB/HIV care.(25) Integrated interventions could be implemented within this framework in a feasible and scalable way to improve outcomes for patients with TB across South Africa and beyond.

Building on previous successes with MI and mHealth interventions, we developed a complex behavioural intervention (ProLife) comprising MI-based counselling and SMS, targeting three lifestyle risk behaviours for poor TB outcomes (smoking, hazardous/harmful alcohol consumption and poor medication adherence), and delivered by LHWs. We then conducted a randomised controlled trial (RCT) to assess the effectiveness of the ProLife intervention on improving TB treatment outcomes, smoking abstinence, reducing alcohol consumption and improving adherence to TB and ART medication compared to usual care. The cost-effectiveness of the intervention was also assessed but only the costing results will be presented in this paper.

METHODS

Study design and participants

This was a prospective, two-arm, multi-centre, individually randomised controlled trial, which took place across 27 primary care clinics in 3 districts in South Africa (Lejweleputswa in the Free State; Bojanala in the North West province; and Sedibeng in Gauteng province). Adult patients (18 years or older) were eligible for the study if they had drug-sensitive pulmonary TB (PTB), and were initiating TB treatment or had been on TB treatment for less than a month for this treatment episode (both "new" and "retreatment patients"). They had to be tobacco smokers (defined as smoking daily or non-daily in the last four weeks on the Global Adult Tobacco Survey questionnaire)(26) and/or hazardous/harmful drinkers who were not alcohol dependent (Alcohol Use Disorders Identification Test (AUDIT) score ≥ 8 for men or ≥ 7 for women but <20).(27) They also had to have access to a mobile phone and understand one of the 4 languages used for the trial (English, isiZulu, SeSotho and

For peer review only - http://bmjopen.bmj.com/site/about/guidelines.xhtml

Setswana). Potential participants were recruited consecutively at the participating clinics between 15 November 2018 and 31 August 2019. Trained field workers identified those interested in the study and screened them for eligibility. If eligible and willing to be enrolled into the trial, written informed consent was obtained. The RCT received ethics approval from the Research Ethics Committees of the five participating research institutions.(28)

Randomisation and blinding

Patients were centrally randomised (1:1) to the ProLife intervention or control group using a randomised sequence generator by the trial statistician (MK) who was blind to the arm allocation. We used block randomisation with varying block sizes stratified by clinic so as to achieve equal numbers in intervention and control groups within each clinic. Fieldworkers used sequentially numbered, sealed, opaque envelopes to allocate participants to intervention or control. ProLife involved a complex behavioural intervention therefore LHWs and participants could not be blinded to the intervention. However, the determination of the primary outcome was done by the TB nurses who were blinded to the intervention status of the participants, based on routinely collected data. The statistician (MK) was blinded to the intervention or control arm allocation of participants during the analysis.

Intervention and procedures

The ProLife intervention was developed based on a conceptual framework, following a review of pre-existing evidence.(29) This framework assumed that smoking cessation, reducing harmful alcohol use and improved adherence to TB and HIV treatment would result in improved TB treatment outcomes.(28) The intervention consisted of 3 brief MI counselling sessions, lasting 15-20 minutes, one month apart, delivered by trained LHWs at their TB clinic. The first MI session took place immediately or shortly after the randomisation and involved prioritisation and agenda setting, wherein the participant determined which factor should be prioritised (either a plan to quit tobacco smoking, or reduce or quit drinking, or deal with barriers relating to ART or TB medication adherence). The second and third sessions built on the previous one until all relevant behavioural problems had been addressed. These sessions were reinforced with follow-up SMS text messages, twice a week over 12 week.(28) Study patients received 10 TB-related messages followed by seven alcohol reduction- and/or seven smoking cessation-related messages, as appropriate. Messages were aimed at giving information, and augmenting motivation or behavioural skills (we refer to the feasibility paper for more details)(29).

For peer review only - http://bmjopen.bmj.com/site/about/guidelines.xhtml

Participants randomised to the ProLife intervention also received the same "usual care" as those in the control group. The control group received the usual care and routine treatment and support offered to TB patients in South Africa, which varies by district but includes: health education; dietetic input; social support; point of care biochemical testing; and HIV testing with pre-and post-test HIV test counselling.

Data were collected at baseline, 3 and 6-months and recorded by fieldworkers equipped with mobile phones with the ProLife mobile data collection application (built with CommCare)(30) installed. They used a standardised electronic case report form (CRF) and followed standard operating procedures (SOPs) to ensure quality. Details of data collection, protection and storage procedures have been reported elsewhere.(28)

Patient and public involvement

Patients or the public were not involved in the design, or conduct, or reporting, or dissemination plans of our research.

Outcomes

Primary Outcome

The primary outcome of TB treatment success at 6 to 9 months follow-up (depending on when it was recorded) was as per the WHO definitions adopted in South Africa,(8) that is, either successful treatment (cured or treatment completed) or failed treatment, death, acquired drug resistance, loss to follow-up (defined as treatment interruption of more than 2 months), or outcome not evaluated. It was measured using the routinely collected TB treatment outcomes in patients' individual files.

Secondary Outcomes

For those participants with bacteriologically confirmed PTB at baseline (either sputum acid fast bacilli (AFB) positive, culture positive or GeneXpert positive PTB), sputum conversion at the end of treatment ("cure rate") was measured as a secondary outcome.(8) Continuous smoking abstinence was assessed at 3 and 6-months follow-up in those participants who were current cigarette smokers at baseline. It was defined as having quit smoking completely and a self-report of not smoking more than 5 cigarettes from the start of the study, in addition to a negative biochemical test (exhaled Carbon Monoxide (CO) < 7 ppm).(31,32) Changes in alcohol consumption were computed using the AUDIT questionnaire scores measured at 3 and 6-months follow-up in those participants who were hazardous/harmful drinkers at baseline.

For peer review only - http://bmjopen.bmj.com/site/about/guidelines.xhtml

HIV positive participants were asked about ART status at baseline, 3 and 6-months using standardised questions on the CRF and change in ART status as measured at the 2 follow-up times.

TB and ART medication adherence was measured using modified versions of the AIDS Clinical Trials Group Adherence Questionnaire, a validated tool for measuring adherence specifically to ART.(33) Adherence was measured using an adherence index calculated by the formula (using the 4-day recall): [Total number of doses taken/Total number of doses prescribed] x 100. Patients with at least 95% adherence were classed as having optimal adherence and those with less than 95% were classed as having low (or suboptimal) adherence. This was assessed at 3 and 6-months.

Training and Intervention Fidelity Monitoring

The training and intervention fidelity monitoring is described in more detail in previous papers.(28,29) In brief, 18 LHWs, 3 district coordinators and 1 research assistant who focused on counselling supervision underwent MI training over 5 days. LHWs completed a post-session semi-structured form onto which they indicated the extent to which they implemented each element of MI, as well as their general qualitative impressions of that particular session. In addition, we assessed MI intervention fidelity based on ratings of the counsellors' recorded MI sessions, as described below and in footnotes to Supplementary table 4. SMS-delivery was also monitored. SMS-message delivery was also assessed.

Economic evaluation

The ProLife intervention costs consisted of the costs of training and the delivery of the ProLife intervention, including relevant personnel involvement (trainers and LHWs), materials used, travel, accommodation and refreshments, and digital infrastructure for the intervention. These were estimated based on research team records. Usual care costs consisted of TB medication costs, biochemical investigations, and ART costs if applicable. These were estimated based on information obtained through routine records. The country specific version of Euroqol (EQ-5D-3L) for South Africa was administered to participants at baseline, 3 and 6-months follow-up to measure health-related quality of life.(34,35)

Statistical analysis

The sample size was estimated at 696 in total (348 participants per arm), to detect a 10% difference in TB treatment success rates (0.86 vs. 0.76) in the ProLife arm (intervention) vs. the control arm with 80% power, a significance level of 0.05 and 25% attrition rate. The assumed success rates in the

For peer review only - http://bmjopen.bmj.com/site/about/guidelines.xhtml

control group were based on actual success rates in TB patients in the studied provinces obtained from TB managers at the time of the grant application for this study.

We summarised baseline data descriptively by trial arm. For the primary outcome, we conducted statistical analysis on an intention-to-treat basis. We used binary logistic regression to compare the main outcome (TB treatment success rate) between the intervention and the usual care arm. Where treatment outcome data were missing, the outcome was coded as unsuccessful. We carried out similar analyses for the secondary outcomes with appropriate regression techniques. For the reduction in harmful or hazardous drinking, we used linear regression to estimate the difference in total AUDIT score between control and intervention groups accounting for the baseline AUDIT score as covariate. Separate analyses at 3 and 6-months were performed.

For our main analyses we adjusted for baseline characteristics if these differed between trial arms at baseline. The covariates that we controlled for in each model are specified when a model is presented. The statistical packages STATA (36) and R (37) were used to carry out the analyses, with a P-value < 0.05 considered statistically significant.

The validated Motivational Interviewing Treatment Integrity (MITI) coding tool (version 4.2.1) was used to assess MI intervention fidelity.(38) The coding entailed making 'Global Ratings' (on 4 dimensions: Cultivating change talk, Softening sustain talk, Partnership and Empathy) and 'Behaviour' counts (with respect to the items: giving information, persuade, persuade with permission, question, simple reflection, complex reflection, affirm, seeking collaboration, emphasising autonomy and confront). A score was assigned to each of these items and the scores compared against the competency and proficiency thresholds that are specified in the MITI manual.

For the analysis of the costs, all costs were collected in South Africa Rand (ZAR) except for the data management system subscription. Results are presented in both ZAR and US dollar ($) using the 2019 OECD exchange rate (1 USD = 14.448 Rand).(34) No South African specific valuation set was available. The valuation set of Argentina, based on a Visual Analogue Scale for EQ-5D-3L, was used to derive utility values, because the GDP per capita in international dollars was the closest between the two countries at the time of analysis.(35,39) Quality-adjusted life years (QALYs) were derived from the utility values at the time points by calculating the area under the curve.(40)

RESULTS

For peer review only - http://bmjopen.bmj.com/site/about/guidelines.xhtml

Participant enrolment and follow-up

A total of 2099 TB patients were screened for eligibility out of which 574 consenting and eligible participants were randomised: 291 to control and 283 to intervention. Trial recruitment was terminated on 31 August 2019 before the planned sample size was reached because of budget and time constraints. In the intervention arm, 227 (80.2%) participants completed the first MI (MI 1) session, 199 (70.3%) MI 2 and 150 (53.0%) MI 3. In the intervention arm, at least one message was delivered to 208 (73.5%) participants, while 99 (35.0%) received all messages. Of those randomised to the control and intervention groups, the primary outcome was recorded in 244 (83.8%) and 253 (89.4%) participants, respectively.(Figure 1)

Figure 1 Consort flow diagram

Baseline participant characteristics of the intervention and control arm

Socio-demographic; socio-economic and clinical characteristics

Baseline characteristics were distributed similarly in the intervention and control arms for most variables but with some imbalances in educational level. A total of 513 (91.3%) participants were new TB patients, 129 (22.5%) women, and nearly all had PTB (ICD-10 A15) without extrapulmonary TB manifestations (553 (98.9%)). About half of the participants were HIV positive (305 (53.2%)), of whom 204 (65.4%) were on cotrimoxazole and 257 (82.4%) were on anti-retroviral therapy. (Table 1) Details of marital status, employment, wealth, depression status and co-morbidities are presented in Supplementary table 1.

For peer review only

For peer review only - http://bmjopen.bmj.com/site/about/guidelines.xhtml

Table 1 Baseline descriptive socio-economic statistics and clinical characteristics by study arm

	Control (N = 291)<br>n (%)*	Intervention (N = 283)<br>n (%)*	Total (N=574)<br>n (%)*
**Age in years:** mean (SD)	39.37 (12.60)	38.56 (11.15)	
**Female Sex**	69 (23.7)	60 (21.2)	129 (22.5)
**Education**			
No education	7 (2.4)	5 (1.8)	12 (2.1)
Grades 1-5	23 (7.9)	20 (7.1)	43 (7.5)
Grades 6-7	32 (11.0)	35 (12.4)	67 (11.7)
Grades 8-11	96 (33.0)	128 (45.2)	224 (39.0)
Grade 12	87 (29.9)	70 (24.7)	157 (27.4)
Higher	24 (8.2)	8 (2.8)	32 (5.6)
Declined to answer**	22 (7.6)	17 (6.0)	39 (6.8)
**TB Patient category**			
New patient	264 (92.3)	249 (90.2)	513 (91.3)
Relapse	10 (3.5)	9 (3.3)	19 (3.4)
Re-treatment after default	9 (3.1)	14 (5.1)	23 (4.1)
Re-treatment after failure	1 (0.3)	2 (0.7)	3 (0.5)
Other	2 (0.7)	2 (0.7)	4 (0.7)
**TB Site of disease** Pulmonary only (ICD-10 A15)	281 (98.9)	272 (98.9)	553 (98.9)
**TB Sputum smear, Gene XPert, or culture result available** N=	236	227	463
**At least one Sputum smear, Gene XPert, or culture result positive**	208 (88.1)	195 (85.9)	403 (87.0)
**HIV status**			
Negative	118 (40.7)	125 (44.2)	243 (42.4)
Positive	163 (56.2)	142 (50.2)	305 (53.2)
Unknown	9 (3.1)	16 (5.7)	25 (4.4)
**HIV positive patients**			
**Using Cotrimoxazole**	104 (63.8)	100 (67.1)	204 (65.4)
**Using anti-retroviral**	139 (85.3)	118 (79.2)	257 (82.4)

For peer review only - http://bmjopen.bmj.com/site/about/guidelines.xhtml

* Frequencies and (percentages) are presented unless otherwise stated
**More variables with the option "declined to answer" are listed in the Supplementary table 1

Table 2 Baseline descriptive alcohol and smoking characteristics by study arm

	Control (N=291) n (%)*	Intervention (N=283) n (%)*	Total (N=574) n (%)*
**In the past month, smoked tobacco**			
Not at all**	110 (37.8)	92 (32.5)	202 (35.2)
Daily	149 (51.2)	149 (52.7)	298 (51.9)
Less than Daily	32 (11.0)	42 (14.8)	74 (12.9)
**Had a drink in the past 12-months**	208 (71.5)	223 (78.8)	431 (75.1)
**AUDIT Score (males) : mean (SD) [max: 19]†**	12.27 (3.98)	13.02 (3.78)	12.66 (3.89)
**AUDIT Score (females): mean (SD) [max: 19]†**	11.32 (4.02)	10.98 (4.02)	11.15 (4.0)
**Hazardous/harmful drinking and Smoking Combined (Constructed)**			
Hazardous/harmful drinking Only‡	110 (37.8)	92 (32.5)	202 (35.2)
Smoking Only	103 (35.4)	77 (27.2)	180 (31.4)
Smoking and hazardous/harmful drinking‡	78 (26.8)	114 (40.3)	192 (33.4)

* Frequencies and (percentages) are presented unless otherwise stated
**Non-smokers were only included if they were harmful or hazardous drinkers
†Only hazardous/harmful drinkers and/or current smokers were included in the study. Therefore TB patients were excluded if they were non-current smokers and had an AUDIT score <7 (females) or <8 (males) or >19; however, they were included if they were smokers independent of whether they had a drink in the past year and therefore independent of the AUDIT score. These AUDIT scores are thus representative of the mean AUDIT scores in the entire study sample and differ from the AUDIT score in the harmful/hazardous drinkers whose change in AUDIT score was measured at 3 and 6-months follow-up (see Table 3)
‡Harmful/hazardous drinking is defined as Alcohol Use Disorders Identification Test (AUDIT) score ≥ 8 for men or ≥ 7 for women but <20

For peer review only

For peer review only - http://bmjopen.bmj.com/site/about/guidelines.xhtml

There were 372 current smokers (298 daily, 74 less than daily). Seventy eight participants (26.8 %) in the control arm were dual smokers *and* drinkers compared to 114 (40.3 %) in the intervention arm. In the control arm, 110 (37.8%) were hazardous/harmful drinkers only and 103 (35.4%) were smokers only, compared to 92 (32.5%) and 77 (27.2) respectively, in the intervention arm. (Table 2) More details of smoking and drinking history, forms of tobacco use, addiction and quit attempts are presented in Supplementary Table 2.

Primary outcome

Overall, 396 (70%) of participants were classified as treated successfully (treatment completed or cured). The remainder either interrupted treatment, failed treatment, developed drug resistance, was transferred out or had an unknown the treatment outcome.(Supplementary table 3) The percentage of successful TB treatment did not differ significantly between the control and intervention arm (70.1% vs. 67.8%). The odds ratio (OR) for successful TB treatment was 0.90 (95%CI: (0.64,1.27)) comparing the intervention arm to the control arm; and was similar to adjusted ORs. (Tables 3 and 4)

Secondary outcomes

Cure rates

Among the 403 participants who had at least one positive bacteriological result at baseline, 168 (41.7%) were recorded as cured; of these, 83/205 (39.9%) were in the control arm compared to 85/195 (43.6%) in the intervention arm. The OR of being cured was 1.16 (95% CI: 0.83,1.63) in the intervention versus the control arm; and was similar to the adjusted OR. (Tables 3 and 4)

Continuous smoking abstinence

Among those who identified as cigarette smokers at baseline (345 (60.1%)), 27 had information (self-report plus biochemical verification) to enable the identification of continuous abstinence at 6-months of which 22 had continuously abstained from smoking. These were similarly distributed across the two study arms: 10 (5.59%) participants in the intervention arm compared to 12 (7.23%) in the control arm, OR 0.76 (95% CI: 0.35,1.63). (Tables 3 and 4) At the 3-month follow-up 20 (11.2%) participants in the intervention arm compared to 27 (16.3%) in the control arm continuously abstained from smoking, OR 0.65 (95% CI: 0.37,1.14). (Tables 3 and 5)

For peer review only - http://bmjopen.bmj.com/site/about/guidelines.xhtml

TABLE 3 Descriptive statistics for primary and secondary outcomes by study arm at baseline (where available), 3-months (where available) and 6-months

	Baseline n (%)*			3-month follow-up n (%)*			6-month follow-up n (%)*		
	**Control**	**Intervention**	**Total**	**Control**	**Intervention**	**Total**	**Control**	**Intervention**	**Total**
**TB treatment status[a]**									
Successful**							204 (70.1)	192 (67.8)	396 (69.0)
Not Successful							87 (29.9)	91 (32.16)	178 (31.01)
**Cured [a], *****									
Yes							83 (39.9)	85 (43.6)	168 (41.7)
No							125 (60.1)	110 (56.4)	235 (58.3)
**Continuous smoking abstinence[b]**									
Yes				27 (16.3)	20 (11.2)	47 (13.6)	12 (7.2)	10 (5.6)	22 (6.4)
No				139 (83.7)	159 (88.8)	298 (86.4)	154 (92.8)	169 (94.4)	323 (93.6)
**Harmful/hazardous drinkers[!]: N =**	188†	206†	394†	141†	130†	271†	112†	127†	239†
AUDIT score: mean (SD)	12.76 (3.42)	13.12 (3.47)	12.94 (3.45)	8.28 (6.18)	8.84 (5.38)	8.55 (5.81)	8.79 (6.66)	8.70 (5.83)	8.74 (6.22)
Difference from baseline: mean (SD)				-4.61 (6.26)	-4.07 (5.33)	-4.35 (5.83)	-4.25 (6.56)	-4.17 (6.61)	-4.21 (6.57)
**HIV positive patients**:	163† (56.2)	142† (50.2)	305† (53.2)	122†	83†	205†	100†	83†	183†
**Taking ART medication if HIV positive‡**	139 (85.3)	115 (81.0)	254 (83.3)	91 (74.6)	58 (69.9)	149 (72.7)	80 (80.0)	74 (89.2)	154 (84.2)

For peer review only - http://bmjopen.bmj.com/site/about/guidelines.xhtml

**ART medication adherence**[b]						
Optimal adherence	101 (99.0)	64 (98.5)	165 (98.8)	75 (97.4)	64 (97.0)	139 (97.2)
Suboptimal adherence	1 (1.0)	1 (1.54)	2 (1.2)	2 (2.6)	2 (3.0)	4 (2.8)
**TB medication adherence**						
Optimal adherence	181 (92.3)	138 (90.8)	319 (91.7)	61 (89.7)	59 (90.8)	120 (90.2)
Suboptimal adherence	15 (7.6)	14 (9.2)	29 (8.3)	7 (10.3)	6 (9.2)	13 (9.8)

a: only assessed at 6-months

b: assessed at 3 and 6-months; this table refers to *cigarette* smokers only (other forms of tobacco smoking are excluded)

* Frequencies and (percentages) are presented unless otherwise stated

**Primary Outcome: This is a binary variable defined as either successful treatment (cured or treatment completed) or failed treatment, death, acquired drug resistance, loss to follow-up or treatment interrupted for more than 2 months, or outcome not evaluated/unknown.

***Based on having a cured treatment outcome among those who were bacteriologically positive at baseline.

! hazardous/harmful drinkers who are not alcohol dependent= AUDIT score ≥ 8 for men or ≥ 7 for women but < 20; *! Important distinction at baseline for eligibility purposes.

†Denominator for the mean (SD) or denominator for %

‡ Information on HIV-positivity was obtained from information from TB records combined with patient self-report at baseline. True HIV-positivity rates may have been higher.

For peer review only

TABLE 4 Regression analysis results for the primary and secondary outcomes at 6-months

	Crude Odds Ratio (95% CI)*	P Value*	Adjusted Odds Ratio (95% CI)*	P Value*
**Primary outcome**				
TB treatment status: Successful (Ref: Not successful)	0.90 (0.64,1.27)	0.548	0.86‡ (0.60,1.24)	0.421
**Secondary outcomes**				
Cured (Ref: Not Cured)	1.16 (0.83,1.63)	0.374	1.07‡ (0.76,1.51)	0.684
Continuous smoking abstinence (Ref: No)***	0.76 (0.35,1.63)	0.482		
TB medication adherence (Ref: Optimal)	0.89 (0.26,3.07)	0.849		
ART medication adherence (Ref: Optimal)	1.17 (0.14,9.94)	0.884		
Taking ART medication (Ref: No)	2.05[!] (0.80,5.27)	0.136		
AUDIT	-0.04[**] (-2,1.91)	0.966	0.02[!!] (-1.55,1.6)	0.976

* Analyses accounted for potential clustering by centre.
‡ Adjusted for district, sex, and smoking/drinking status and HIV status at baseline. It is worth noting that of the variables in the adjusted model; the only statistically significant result is for the district variable.
! Adjusting for art status at baseline
**Controlling for the AUDIT baseline values. The values represent the study arm regression coefficient.
!! Controlling for the AUDIT baseline values and adjusted for district, sex, and smoking/drinking status and HIV status at baseline. The values represent the study arm regression coefficient.
***Given the limited number of those who were identified as continually abstained, we were only able to adjust for one additional variable at a time. Adding one of the following variables: heaviness of smoking, type of drinker at baseline, age when started smoking, and the duration of smoking at baseline; the adjusted odds ratio of continuous abstinence comparing the intervention to the control arm ranged between 0.73 and 0.76 with similar confidence limits as for the crude estimate.

For peer review only - http://bmjopen.bmj.com/site/about/guidelines.xhtml

TABLE 5 Regression analysis results for secondary outcomes measured at 3-months				
Secondary outcome	Crude Odds Ratio (95% CI)*	P Value*	Adjusted Odds Ratio (95% CI)*	P Value*
Continuous smoking abstinence (Ref: No)**	0.65 (0.37,1.14)	0.135		
TB medication adherence (Ref: Optimal)	1.22 (0.52,2.87)	0.641		
ART medication adherence (Ref: Optimal)	1.58 (0.10,26.12)	0.750		
Taking ART medication (Ref: No)	0.79[!] (0.38,1.65)	0.53	0.74‡ (0.35,1.58)	0.443
AUDIT	0.55† (-1.01,2.11)	0.474	0.74[!!] (-0.62,2.1)	0.273

*Analyses accounted for clustering.
! Adjusting for art status at baseline
‡ Adjusted for art status at baseline, district, sex, and smoking/drinking status and HIV status at baseline.
†Controlling for the AUDIT baseline values. The values represent the study arm regression coefficient.
!! Controlling for the AUDIT baseline values and adjusted for district, sex, and smoking/drinking status and HIV status at baseline. The values represent the study arm regression coefficient.
** Given the limited number of those who were identified as continually abstained, we were only able to adjust for one additional variable at a time. Adding one of the following variables: heaviness of smoking, type of drinker at baseline, age when started smoking, and the duration of smoking at baseline; the adjusted odds ratio of continuous abstinence comparing the intervention to the control arm ranged between 0.63 and 0.66 with similar confidence limits as for the crude estimate.

For peer review only - http://bmjopen.bmj.com/site/about/guidelines.xhtml

Change in harmful/hazardous drinking

AUDIT scores were about 4 points lower at both follow-up times than at baseline independent of the intervention.(Table 3) In the intervention arm, participants had, on average, a reduction of 0.04 points (95% CI: -2,1.91) on the AUDIT score at 6-months, compared to those in the control arm controlling for baseline scores; whereas an average increase of 0.55 (95% CI: -1.01,2.11) was observed at 3-months. (Tables 4 and 5)

Medication adherence and ART uptake

At 6-months, the OR of taking ART medication was 2.05 (95% CI: 0.80,5.27) comparing the intervention arm to the control arm and controlling for ART baseline medication status; whereas it was 0.79 (95% CI: 0.38,1.65) at 3-months. The proportion of participants who had optimal TB medication adherence was 90.2% (120/133) at 6-months and 91.7% (319/348) at 3-months. Suboptimal TB medication adherence ORs were 0.89 (95%CI: 0.26,3.07) and 1.22 (95%CI: 0.52,2.87) comparing intervention arm to the control arm at 6-months and 3-months, respectively. The proportions of participants on ART who had optimal ART medication adherence were high at both 3-months (165/167, 98.8%) and 6-months follow-up (139/143, 97.2%). Suboptimal ART medication adherence ORs were 1.17 (95%CI: 0.14,9.94) and 1.58 (95%CI: 0.10,26.12) comparing the intervention arm to the control arm at 6-months and 3-months, respectively. (Tables 3, 4 and 5)

Intervention fidelity

Motivational interviewing fidelity

The recordings of 17 counsellors (one each) were transcribed verbatim and then assessed. In terms of the global ratings, the LHWs' counselling sessions were above proficiency levels on all items, namely, cultivating change talk, softening sustain talk, partnership, and empathy (as the mean scores were all above 2). In terms of the summary measures, the LHWs' counselling sessions did not achieve the basic proficiency threshold of 3.5 for the Relational component (Partnership + Empathy) as their mean score was 3.1 (SD=1.19). However, their mean score on the Technical component (Cultivating Change Talk + Softening Sustain Talk) of 3.3 (SD=0.97) was above the threshold of 3. For behavioural counts, "Asking Questions" had the highest mean score (24.2; SD=10.42), followed by "Affirm" with a mean score of 5.5 (SD=3.7). The counsellors were least likely to engage in the following: Persuade with permission and emphasising autonomy. The mean Reflections to Questions Ratio was 0.23 (SD=0.24). The LHWs made on average 9.3 (SD=4.74) MI adherent (Affirm, Emphasize Autonomy and Seek Collaboration) and 1.2 (SD=2.28) MI non-adherent (Confront and Persuade) statements per session. (Supplementary table 4)

For peer review only - http://bmjopen.bmj.com/site/about/guidelines.xhtml

SMS-delivery

Of the total number of Information-Motivation-Behaviour messages triggered, 3583 (80.4%) were delivered. All due SMS messages were delivered to 95 (41.9%) of the participants who completed the first MI, See Supplementary Table 5 for more details.

Costs and health related quality of life

Unit costs used to estimate the mean costs are presented in the Supplementary Table 6. Cost-utility ratios are not presented since the intervention was not effective. The mean cost of the ProLife intervention was ZAR 2601 (SD 6) ($180.02 (SD $0.42)) per participant in the intervention arm (n=283). The mean cost of usual care was ZAR 681 (SD 357) ($47.13 (SD $24.71)) in the intervention arm (n=122) versus ZAR 706 (SD 302) ($48.86 (SD $20.90)) in the control arm (n=131). The total mean cost of care including the intervention was ZAR 3285 (SD R357) ($227.37 (24.71)) in the intervention arm (n=122). EQ-5D-3L data were available at the 3 time points for 137 intervention and 159 control arm participants. The mean QALYs estimated over six months were 0.442 (SD 0.061) in the intervention arm versus 0.430 (SD 0.074) in the control arm (adjusted mean difference 0.006 (95% CI -0.001 to 0.013)).

DISCUSSION

This RCT did not find evidence for improved TB treatment success rates in those receiving the ProLife intervention compared to those receiving usual care. There was also no significant beneficial effect on any of the secondary outcomes, i.e., smoking, alcohol consumption, medication adherence and ART initiation. To our knowledge there are no other published studies of similar complex interventions that aim to improve TB treatment outcomes in patients who smoke or drink to harmful or hazardous extent.

The lack of effectiveness of our intervention consisting of three sessions of MI combined with SMS can have a number of possible explanations. Although intervention uptake was high (80.2%) for the first counselling session, many participants did not return for the second (29.7%) and third (47%) sessions. As a result of this only about half of the intervention arm participants received all three MI sessions. Furthermore, about one quarter of all participants did not receive any SMS-messages. There were a number of possible technical barriers to SMS delivery: invalid phone numbers, phones that are off for a long duration of time, numbers that are deactivated, and phones that are

For peer review only - http://bmjopen.bmj.com/site/about/guidelines.xhtml

disconnected from the network leading.(41) The proportion of participants with TB treatment outcomes recorded was high, but follow-up rates at 3- and 6-months were low. Due to COVID-19 lockdown, we had to switch to telephonic follow-up of participants using a shortened questionnaire (22 participants) and could not access clinics to retrieve outstanding TB treatment outcomes.

The lack of effectiveness on TB treatment success may also be explained by the complexity of the ProLife intervention itself: counsellors had to address multiple behaviours, namely medication adherence, tobacco smoking and hazardous/harmful drinking. This integrated approach was nevertheless adopted to avoid the need for multiple vertical counselling services (in addition to TB treatment and HIV treatment), to allow the different elements of the programme to reinforce one another, and to improve the affordability, feasibility and acceptability for a future roll-out of the programme. Despite having established the feasibility and acceptability of this approach,(29) it is possible that MI for multiple behaviour change in the ProLife study was counterproductive as counsellors may have ended up not focusing on any of the behaviours at optimal levels. Similarly, patients might have found it difficult to change multiple behaviours simultaneously, especially because smoking and drinking are mutually reinforcing. There is some indirect evidence that sequential interventions may be better, at least for smoking cessation.(42) More intensive counselling (more sessions) or a modified counselling method with or without MI may have been more appropriate.(43) For example, more emphasis on increasing patient knowledge in addition to increasing self-efficacy may have been more effective.(44) The cause of the mobile health message delivery problems (such as poor network coverage, no electricity to charge phones) would need to be investigated in order to increase the effectiveness of future mHealth interventions. Messages may also have to be intensified or modified to be more interactive and/or tailored to specific circumstances of each individual. This would improve the personal value of the intervention to the individual, which is likely to increase the chances of their participation in the intervention.(45) There are also many obstacles to behavioural change that were not modifiable by our intervention (for example poverty, low health literacy, cultural beliefs and migrant labour), which may need to be addressed more explicitly in future studies.

Interventions evaluated by other studies were either complex interventions aimed at improving TB outcomes but without an alcohol or smoking intervention component (46,47) or focused on a single behaviour, namely smoking or drinking.(48,49) Of the latter studies, a brief smoking cessation intervention was effective in inducing smoking cessation in TB patients but did not improve TB outcomes.(48) Conversely, in another study in India, intensive counselling for alcohol disorders led

For peer review only - http://bmjopen.bmj.com/site/about/guidelines.xhtml

to significantly better TB treatment outcomes in the intervention group compared to the control group.(49) Smoking cessation also led to better TB treatment outcomes in a secondary analysis of a large tobacco cessation trial in TB patients in Bangladesh and Pakistan.(50)

The non-significant result for smoking-related outcomes does not align with findings from our previous TB study, which used a single MI session and found that the chance of sustained smoking cessation was twice as high in the MI intervention group compared to the control group,(14) albeit with a less stringent exhaled CO cut-off point. Evidence on the effectiveness of MI for smoking abstinence in non-TB settings has however been more equivocal.(13)

Self-reported alcohol consumption decreased with about 4 points in both intervention and control arms at both follow-up times. Answering questions on drinking in brief intervention trials may alter subsequent self-reported behaviour.(51) Exposing non-intervention control groups to an integral component of the intervention may therefore underestimate the effect of the intervention.(51) There have been few previous studies looking at MI and SMS interventions for the modification of hazardous/harmful drinking in the context of TB. A previous trial of a brief counselling intervention to reduce alcohol consumption in patients with TB did not find a significant effect on alcohol reduction.(52) Outside a TB setting, results have been mixed. A meta-analysis showed a small but significant improvement in outcomes when MI was used in conjunction with cognitive behavioural therapy for co-morbid alcohol use and depression.(53)

Self-reported TB and ART medication adherence was high overall in this population, which is consistent with other studies conducted in South Africa (54,55) and Sub-Saharan Africa in general.(56) It is possible that we did not find a difference in treatment adherence due to a ceiling effect.

There were several key strengths in this RCT. This was an individually randomised controlled trial with a relatively large sample size and a high follow up rate (87%) for the primary outcome. Primary outcome assessment was blinded. This was a novel intervention, which built on previous successes with both MI and mHealth interventions and was aligned with the WHO's call to increase the use of digital technologies to improve TB care.(15) Overall, the quality of the counselling was acceptable and the results of these preliminary MI analyses suggest that the LHWs trained as counsellors were proficient in some areas of MI, as observed by their global rating scores on cultivating change talk, softening sustain talk, partnership and empathy. (Supplementary Table 4) Patients and counsellors

For peer review only - http://bmjopen.bmj.com/site/about/guidelines.xhtml

also reported very positive experiences with the ProLife intervention in the feasibility stage of the study.(29)

There were some limitations associated with this RCT. Trial recruitment had to be terminated before the planned sample size because of constrained time to complete the study occasioned by delays in disbursement of funding, having to pilot the scaled ProLife RCT process and the new data application system, and obtaining approvals from five different institutional review boards. The intervention uptake was generally low for the second and third MI session. Loss to follow-up (LTFU) was high for the 3- and 6-month questionnaires. LTFU was particularly high in the intervention arm at 3-month follow-up. A possible explanation for the latter is that intervention participants were not motivated to return for the 3 month follow-up because – unlike the control participants - they also had to return for 3 MI counselling sessions. Consistent with the normalisation process theory,(57) cognitive participation in the intervention might have been higher had we been deliberate in the implementation to ensure the TB nurse who would have routinely seen the participants, provided additional support and motivated participants to attend MI sessions with the counsellor. In this way, the intervention would have gained 'legitimacy'. Other possible explanations for high LTFU might include those outside the research team's control, for example inability to attend services because of work or migration, as listed in the Figure 1. Self-reports tend to underreport drinking while overestimating adherence behaviour compared with other assessment methods.(58) To enhance the quality of self-reported behaviour, we utilized validated alcohol consumption questionnaire (AUDIT) (27) and a four-day timeline follow-back for medication adherence to reduce recall bias.(33) Smoking cessation was confirmed with exhaled CO using strict cut-off points.

Conclusion and recommendations

This RCT provided evidence that the ProLife intervention was not effective in modifying the primary or secondary outcomes. Valuable lessons were learnt on challenges relating to training LHWs in MI counselling and delivery, the SMS-delivery in a challenging socio-economic context and the reasons for loss to follow-up of TB participants with multiple health problems. Further research is needed to provide answers on how to increase intervention uptake in poor resource settings and whether our complex intervention should have been more intensive (with several sessions for each behavioural problem). Other important questions are whether another counselling method would have been more effective and whether sequential interventions are superior to integrated counselling whereby several behaviours are addressed at the same time. In conclusion, TB multimorbidity is a global

For peer review only

health problem that warrants the urgent design and implementation of appropriate models of care.(21)

For peer review only

For peer review only - http://bmjopen.bmj.com/site/about/guidelines.xhtml

Acknowledgements

We are grateful to the following people: Ms M Malefo for her work as project manager, Ms D Bell for conducting the MI training, Ms N Kitleli for supervising the lay counsellors, the district and clinic TB managers and TB nurses, the fieldworkers and the lay counsellors. The authors are also grateful to the National Department of Health and the three Provincial Departments of Health for granting permission for this study.

Contributors

GML has led on study design and manuscript preparation and contributed to the monitoring of the study implementation. MK contributed to study design and the manuscript, led on the sample size determination and did the statistical analysis. NKM contributed to study design, particularly with respect to development of the MI intervention, did the analysis of the MI data and contributed to the manuscript writing. AVZ led the data management and SMS-system, monitored data quality and contributed to data analysis and manuscript writing. ASM contributed to study design and led on the preparation of the manuscript. NDM contributed to study design, intervention development and manuscript preparation. JL and SP led on the economic evaluation. AT contributed to the economic evaluation. JT was site lead for the Bojanala sub-district. OBO was site lead for the Sedibeng sub-district. MOB contributed to study design, data interpretation and the manuscript. KS and OAA are co-principal investigators. Both contributed to study design, data interpretation and manuscript writing. All authors read and approved the final draft of the manuscript.

Funding

This project was funded by the SA-Medical Research/Newton Foundation Grant on TB control implementation science. (UK/South Africa Newton Fund RFA: TB control implementation science (MRC-RFA-02: TB -05-2015)). This UK funded award is part of the EDCTP2 programme supported by the European Union.

The funder had no role in study design; collection, management, analysis, and interpretation of data; writing of the report or the decision to submit the report for publication.

Trial sponsor: Sefako Makgatho Health Sciences University, RSA. Contact: lekan.ayo-yusuf@smu.ac.za

For peer review only

Competing interests

OA Ayo-Yusuf, the principal investigator, received a research grant (MRC-RFA-02: TB -05-2015) which was allocated to Sefako Makgatho Health Sciences University to pay for the expenses related to the research project. As co-prinicipal investigator K Siddiqi received part of the MRC-Newton research grant (MRC-RFA-02: TB -05-2015) which was allocated to the University of York to support the research by the authors affiliated to the University of York (K Siddiqi, ND Mdege, M Kanaan, S Parrot, J Lee). GL received a small monthly remuneration per month from 1 October 2018 until 30 June 2020 as academic research coordinator. André van Zyl was employed as a research data manager on a part-time basis from 1 September 2017 and 30 June 2020. OA Ayo-Yusuf received support for attending meetings and for travel by Tax Justice Network Africa. He is the Vice Chair of the Standing Committee on Health, Academy of Science, SA (not remunerated).

Ethical considerations

This study received ethics approval from the Research Ethics Committees of: Sefako Makgatho Health Sciences University (SMU; Ref: SMUREC/D/234/2017: IR); the South African Medical Research Council (with the SMU Ethics Committee serving as the Research Ethics Committee Record); the University of Pretoria (Ref: 434/2017); the University of Witwatersrand (M160455); and the University of York (no reference number, approval date 15 January 2017 (29).

Patient Consent for publication

Not applicable

Availability of data and material

Data are stored in the institutional data repository at Sefako Makgatho Health Sciences University called Discover research (https://smu-za.figshare.com/). Data access will be embargoed until 30 June 2023.

Open access

This is an open access article distributed in accordance with a Creative Commons

For peer review only - http://bmjopen.bmj.com/site/about/guidelines.xhtml

Attribution (CC BY 4.0) license, which permits others to copy, redistribute, remix, transform and build upon this work for any purpose, provided the original work is properly cited, a link to the licence is given and indication of whether changes were made. See: https://creativecommons.org/licenses/by/4.0/.

References

1. World Health Organisation. Global tuberculosis report 2020. Available: https://apps.who.int/iris/bitstream/handle/10665/336069/9789240013131-eng.pdf [Accessed 4 Aug 2021].

2. Fitchett JR, MacPherson P. Implementing the End TB Strategy and the intersection with the Sustainable Development Goals. *Trans R Soc Trop Med Hyg* 2016;110:145–7.

3. Naidoo P, Peltzer K, Louw J, et al. Predictors of TB and ART medication non-adherence in Public primary care patients in South Africa: a cross-sectional study. *BMC Public Health* 2013;13(396).

4. Finlay A, Lancaster J, Holtz TH, et al. Patient- and provider-level risk factors associated with default from tuberculosis treatment, South Africa, 2002: A case-control study. *BMC Public Health* 2012;12:56.

5. Waitt CJ, Squire SB. A systematic review of risk factors for death in adults during and after tuberculosis treatment. *Int J Tuberc lung Dis* 2011;15:871–85.

6. Lin HH, Ezzati M, Murray M. Tobacco smoke, indoor air pollution and tuberculosis: A systematic review and meta-analysis. *PLoS Med* 2007;4:0173–89.

7. Rehm J, Samokhvalov A V., Neuman MG, et al. The association between alcohol use, alcohol use disorders and tuberculosis (TB). A systematic review. *BMC Public Health* 2009;9:1–12.

8. Department of Health of South Africa. National Tuberculosis Management Guidelines. Pretoria: 2014. Available from: http://www.tbonline.info/media/uploads/documents/ntcp_adult_tb-guidelines-27.5.2014.pdf [Accessed 4 Aug 2021].

9. Peltzer K. Conjoint alcohol and tobacco use among tuberculosis patients in public primary healthcare in South Africa. *South African J Psychiatry* 2014;20:21–26.

10. Ragan EJ, Kleinman MB, Sweigart B, et al. The impact of alcohol use on tuberculosis treatment outcomes: a systematic review and meta-analysis. *Int J Tuberc Lung Dis* 2020;24:73–82.

11. Rubak S, Sandbæk A, Lauritzen T, et al. Motivational interviewing: A systematic review and

For peer review only - http://bmjopen.bmj.com/site/about/guidelines.xhtml

meta-analysis. *Br J Gen Pract* 2005;55:305–12.

12. Golin CE, Earp JA, Tien HC, et al. A 2-arm, randomized, controlled trial of a motivational interviewing-based intervention to improve adherence to antiretroviral therapy (ART) among patients failing or initiating ART. *J Acquir Immune Defic Syndr* 2006;42:42–51.
13. Lindson N, Thompson TP, Ferrey A, et al. Motivational interviewing for smoking cessation. *Cochrane Database Syst Rev* 2019;2019(7).
14. Louwagie GMC, Okuyemi KS, Ayo-Yusuf OA. Efficacy of brief motivational interviewing on smoking cessation at tuberculosis clinics in Tshwane, South Africa: a randomized controlled trial. *Addiction* 2014;109:1942–52
15. World Health Organization. Handbook for the use of digital technologies to support tuberculosis medication adherence, 2017. Available: https://apps.who.int/iris/handle/10665/259832 [Accessed 4 Aug 2021].
16. Free C, Phillips G, Watson L, et al. The Effectiveness of Mobile-Health Technologies to Improve Health Care Service Delivery Processes: A Systematic Review and Meta-Analysis. *PLoS Med* 2013;10(1).
17. Hirsch-Moverman Y, Daftary A, Yuengling KA, et al. Using mhealth for HIV/TB treatment support in lesotho: Enhancing patient-provider communication in the START study. *J Acquir Immune Defic Syndr* 2017;74:S37–43.
18. Nglazi MD, Bekker LG, Wood R, et al. Mobile phone text messaging for promoting adherence to anti-tuberculosis treatment: A systematic review. *BMC Infect Dis* 2013;13:1–16.
19. Oosterveen E, Tzelepis F, Ashton L, et al. A systematic review of eHealth behavioral interventions targeting smoking, nutrition, alcohol, physical activity and/or obesity for young adults. *Prev Med*. 2017;99:197–206.
20. Haug S, Paz Castro R, Kowatsch T, et al. Efficacy of a technology-based, integrated smoking cessation and alcohol intervention for smoking cessation in adolescents: Results of a cluster-randomised controlled trial. *J Subst Abuse Treat* 2017;82:55–66.
21. Siddiqi K, Stubbs B, Lin Y, et al. TB multimorbidity: a global health challenge demanding urgent attention. *Int J Tuberc Lung Dis* 2021;25:87–90.
22. Moosa S, Derese A, Peersman W. Insights of health district managers on the implementation of primary health care outreach teams in Johannesburg, South Africa: A descriptive study with focus group discussions. *Hum Resour Health* 2017;15:1–9.
23. Marcus TS, Hugo J, Jinabhai CC. Which primary care model? A qualitative analysis of ward-based outreach teams in South Africa. *African J Prim Heal Care Fam Med* 2017;9:1–8.
24. Seidman G, Atun R. Does task shifting yield cost savings and improve efficiency for health

For peer review only

For peer review only - http://bmjopen.bmj.com/site/about/guidelines.xhtml

systems? A systematic review of evidence from low-income and middle-income countries. *Hum Resour Health* 2017;15:1–13.

25. Heunis C, Wouters E, Kigozi G, et al. TB/HIV-related training, knowledge and attitudes of community health workers in the Free State province, South Africa. *African J AIDS Res* 2013;12:113–9.

26. Global Adult Tobacco Survey Collaborative Group. Altanta GC for DC and P. Tobacco Questions for Surveys: A Subset of key questions from the Global Adult Tobacco Survey (GATS). 2011. 2nd Edition.

27. Bohn MJ, Babor TF, Kranzler HR. The Alcohol Use Disorders Identification Test (AUDIT): validation of a screening instrument for use in medical settings. *J Stud Alcohol* 1995;56:423–32.

28. Moriarty AS, Louwagie GM, Mdege ND, et al. ImPROving TB outcomes by modifying LIFE-style behaviours through a brief motivational intervention followed by short text messages (ProLife): Study protocol for a randomised controlled trial. *Trials* 2019;20:1–12.

29. Louwagie GM, Morojele N, Siddiqi K, et al. Addressing tobacco smoking and drinking to improve TB treatment outcomes, in South Africa: A feasibility study of the ProLife program. *Transl Behav Med* 2020; 10: 1491–1503.

30. CommCare. Cambridge, MA: Dimagi; 2017. Available from: https://play.google.com/store/apps/details?id=org.commcare.dalvik&hl=en_US&gl=US. [Accessed 15 June 2021].

31. Siddiqi K, Khan A, Ahmad M, et al. Action to stop smoking in suspected tuberculosis (ASSIST) in Pakistan: a cluster randomized, controlled trial. *Ann Intern Med* 2013;158:667–75.

32. West R, Hajek P, Stead L, et al. Outcome criteria in smoking cessation trials: proposal for a common standard. *Addiction* 2005;100:299–303.

33. van den Boogaard J, Lyimo RA, Boeree MJ, et al. Electronic monitoring of treatment adherence and validation of alternative adherence measures in tuberculosis patients: A pilot study. *Bull World Health Organ* 2011;89:632–9.

34. OECD [Internet]. Available from: https://data.oecd.org/conversion/exchange-rates.htm

35. Augustovski FA, Irazola VE, Velazquez AP, et al. Argentine valuation of the EQ-5D health states. *Value Heal* 2009;12:587–96.

36. StataCorp. Stata Statistical Software: Release 15. College Station, TX: StataCorp LLC. 2017.

37. Team RC. R: A language and environment for statistical computing. Vienna, Austria: R Foundation for Statistical Computing; 2017. Available from: https://www.r-project.org/ [Accessed 04 Aug 2021]

For peer review only - http://bmjopen.bmj.com/site/about/guidelines.xhtml

38. Moyers TB, Rowell LN, Manuel JK, Ernst D, Houck JM. The Motivational Interviewing Treatment Integrity Code (MITI 4): Rationale, Preliminary Reliability and Validity. *J Subst Abuse Treat* 2016;65:36-42. doi:10.1016/j.jsat.2016.01.001
39. International Monetary Fund. IMF DataMapper - GDP per capita, current prices (Purchasing power parity; international dollars per capita), 2018. Available from: https://www.imf.org/external/datamapper/datasets [Accessed 04 Aug 2021]
40. Richardson GMA. Calculation of quality adjusted life years in the published literature: a review of methodology and transparency. *Health Econ* 2004;13:1203–10.
41. Telerivet. Message Statuses . User Guide, 2021. Available from: https://guide.telerivet.com/hc/en-us/articles/360038959611-Message-Statuses. [Accessed 21 June 2021]
42. Meader N, King K, Wright K et al. Multiple risk behavior interventions: Meta-analyses of RCTs. *Am J Prev Med* 2017;53:e19–e30.
43. Abraham C, Michie S. A taxonomy of behavior change techniques used in interventions. *Health Psychol* 2008;27:379-87.
44. Kahwati L, Viswanathan M, Golin CE, et al. Identifying configurations of behavior change techniques in effective medication adherence interventions: a qualitative comparative analysis. *Syst Rev* 2016;5(83).
45. Lustria MLA, Noar SM, Cortese J, et al. A meta-analysis of web-delivered tailored health behavior change interventions. *Journal of Health Communication* 2013;18: 1039–69.
46. Khachadourian V, Truzyan N, Harutyunyan A, et al. People-centred care versus clinic-based DOT for continuation phase TB treatment in Armenia: a cluster randomized trial. *BMC Pulm Med* 2020;20(1):105.
47. Thiam S, LeFevre AM, Hane F, et al. Effectiveness of a strategy to improve adherence to tuberculosis treatment in a resource-poor setting: a cluster randomized controlled trial. *JAMA* 2007;297:380–6.
48. Goel S, Kathiresan J, Singh P SR. Effect of a brief smoking cessation intervention on adult tobacco smokers with pulmonary tuberculosis: A cluster randomized controlled trial from North India. *Indian J Public Heal* 2017;61(Suppl 1):S47–53
49. Manogaran C, Nagarajan K, Ovung S, et al. Alcohol intervention strategy among tuberculosis patients: a pilot study from South India. *Int J Tuberc Lung Dis* 2017;21:947–52
50. Siddiqi K, Keding A, Marshall AM, et al. Effect of quitting smoking on health outcomes during treatment for tuberculosis: secondary analysis of the TB & Tobacco Trial. *Thorax* 2021 Published Online First Jul 16:thoraxjnl-2020-215926. doi: 10.1136/thoraxjnl-2020-215926.

For peer review only

For peer review only - http://bmjopen.bmj.com/site/about/guidelines.xhtml

51. McCambridge J, Kypri K. Can simply answering research questions change behaviour? Systematic review and meta analyses of brief alcohol intervention trials. *PLoS One* 2011;6(10):e23748.

52. Peltzer K, Naidoo P, Louw J, et al. Screening and brief interventions for hazardous and harmful alcohol use among patients with active tuberculosis attending primary public care clinics in South Africa: results from a cluster randomized controlled trial. *BMC Public Health* 2013;13:699.

53. Riper H, Andersson G, Hunter SB, et al. Treatment of comorbid alcohol use disorders and depression with cognitive-behavioural therapy and motivational interviewing: a meta-analysis. *Addiction* 2014;109:394–406.

54. Webb Mazinyo E, Kim L, Masuku S et al. Adherence to Concurrent Tuberculosis Treatment and Antiretroviral Treatment among Co-Infected Persons in South Africa. *PLoS One* 2008;11:e0159317-e.

55. McInerney PA, Nicholas PK, Wantland D et al. Characteristics of anti-tuberculosis medication adherence in South Africa. *Appl Nurs Res* 2007;20:164–70.

56. Mills EJ, Nachega JB, Buchan I et al. Adherence to Antiretroviral Therapy in Sub-Saharan Africa and North America: A Meta-analysis. *JAMA* 2006;296:679–90.

57. Gallacher K, May CR, Montori V, et al. Understanding Patients' Experiences of Treatment Burden in Chronic Heart Failure Using Normalization Process Theory. *Ann Fam Med* 2011;9:235-243.

58. Stirratt MJ, Dunbar-Jacob J, Crane HM et al. Self-report measures of medication adherence behavior: recommendations on optimal use. *Transl Behav Med* 2015;5:470–82.

For peer review only

For peer review only - http://bmjopen.bmj.com/site/about/guidelines.xhtml

For peer review only

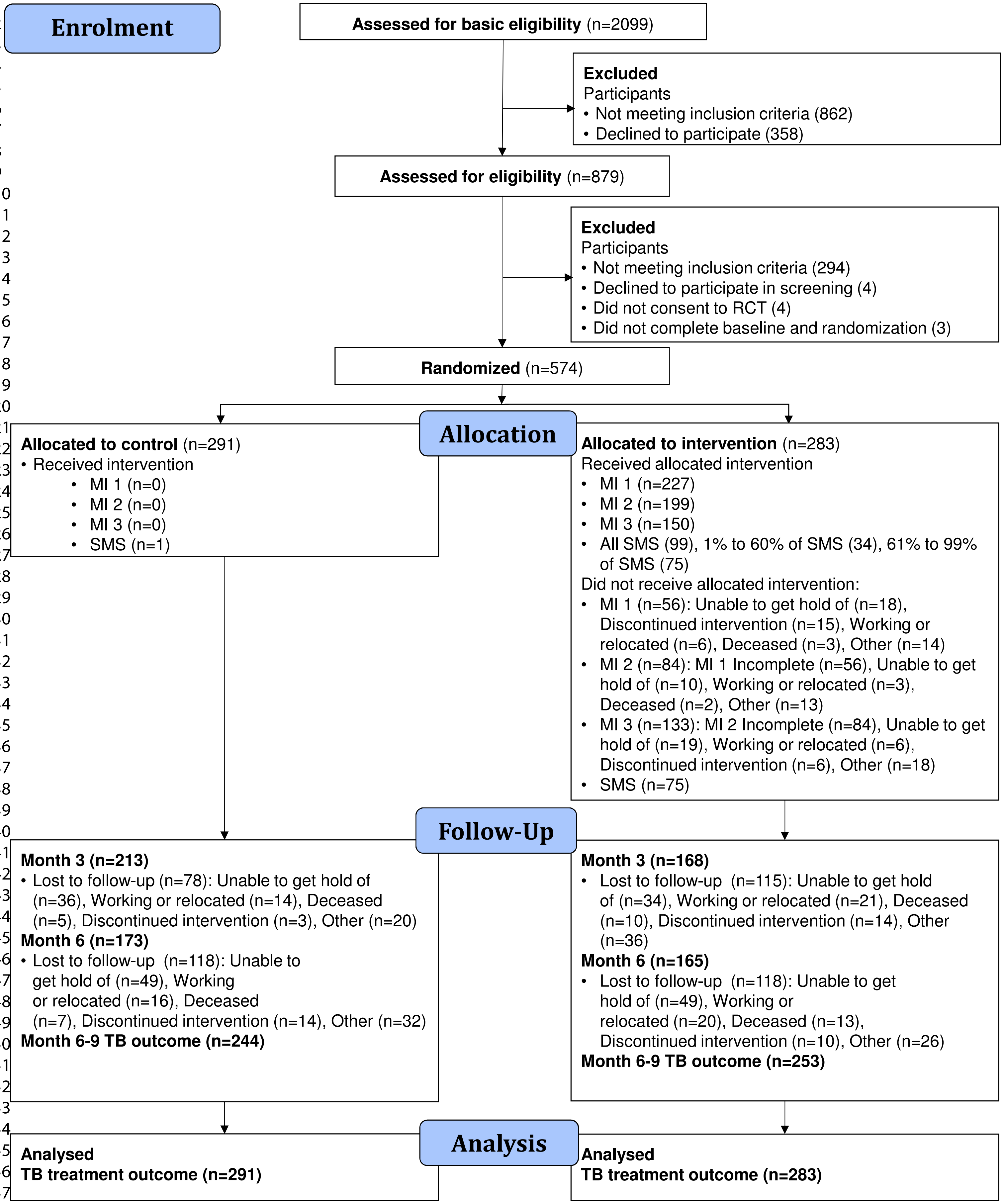
Enrolment
Assessed for basic eligibility (n=2099)
Excluded
Participants
• Not meeting inclusion criteria (862)
• Declined to participate (358)
Assessed for eligibility (n=879)
Excluded
Participants
• Not meeting inclusion criteria (294)
• Declined to participate in screening (4)
• Did not consent to RCT (4)
• Did not complete baseline and randomization (3)
Randomized (n=574)
Allocation
Allocated to control (n=291)
• Received intervention
• MI 1 (n=0)
• MI 2 (n=0)
• MI 3 (n=0)
• SMS (n=1)
Allocated to intervention (n=283)
Received allocated intervention
• MI 1 (n=227)
• MI 2 (n=199)
• MI 3 (n=150)
• All SMS (99), 1% to 60% of SMS (34), 61% to 99% of SMS (75)
Did not receive allocated intervention:
• MI 1 (n=56): Unable to get hold of (n=18), Discontinued intervention (n=15), Working or relocated (n=6), Deceased (n=3), Other (n=14)
• MI 2 (n=84): MI 1 Incomplete (n=56), Unable to get hold of (n=10), Working or relocated (n=3), Deceased (n=2), Other (n=13)
• MI 3 (n=133): MI 2 Incomplete (n=84), Unable to get hold of (n=19), Working or relocated (n=6), Discontinued intervention (n=6), Other (n=18)
• SMS (n=75)
Follow-Up
Month 3 (n=213)
• Lost to follow-up (n=78): Unable to get hold of (n=36), Working or relocated (n=14), Deceased (n=5), Discontinued intervention (n=3), Other (n=20)
Month 6 (n=173)
• Lost to follow-up (n=118): Unable to get hold of (n=49), Working or relocated (n=16), Deceased (n=7), Discontinued intervention (n=14), Other (n=32)
Month 6-9 TB outcome (n=244)
Month 3 (n=168)
• Lost to follow-up (n=115): Unable to get hold of (n=34), Working or relocated (n=21), Deceased (n=10), Discontinued intervention (n=14), Other (n=36)
Month 6 (n=165)
• Lost to follow-up (n=118): Unable to get hold of (n=49), Working or relocated (n=20), Deceased (n=13), Discontinued intervention (n=10), Other (n=26)
Month 6-9 TB outcome (n=253)
Analysis
Analysed
TB treatment outcome (n=291)
Analysed
TB treatment outcome (n=283)

SUPPLEMENTARY TABLES

Supplementary table 1 Detailed baseline descriptive statistics for socio-demographic, socio-economic, and clinical characteristics by study arm

	Control (N = 291) n (%)*	Intervention (N = 283) n (%)*	Total (N=574) n (%)*
**Marital status**			
Married or living together	102 (35.1)	95 (33.6)	197 (34.3)
Divorced/separated	20 (6.9)	18 (6.4)	38 (6.6)
Widowed	10 (3.4)	7 (2.5)	17 (3.0)
Never married and never lived together	144 (49.5)	150 (53.0)	294 (51.2)
Declined to answer	15 (5.2)	13 (4.6)	28 (4.9)
**Employment**			
Self-employed (full-time)	30 (10.3)	36 (12.7)	66 (11.5)
Employed full-time (30 hrs a week or more)	62 (21.3)	54 (19.1)	116 (20.2)
Employed part-time (less than 30 hrs a week)	19 (6.5)	29 (10.2)	48 (8.4)
Retired	17 (5.8)	16 (5.7)	33 (5.7)
Unemployed (but able to work)	125 (43.0)	120 (42.4)	245 (42.7)
Unable to work because of long-term disability or ill health	9 (3.1)	8 (2.8)	17 (3.0)
Full-time student	12 (4.1)	4 (1.4)	16 (2.8)
Caring from my home and family/doing household work/housewife	0 (0.0)	2 (0.7)	2 (0.3)
Occasional work (“piece job”)	17 (5.8)	12 (4.2)	29 (5.1)
Declined to answer	0 (0.0)	2 (0.7)	2 (0.3)
**Ever worked or spent time in mines**			
No	244 (83.8)	237 (83.7)	481 (83.8)
Yes	46 (15.8)	45 (15.9)	91 (15.9)
**Household items (Yes is displayed)**			
A radio	249 (85.6)	234 (82.7)	483 (84.1)
A television	255 (87.6)	255 (90.1)	510 (88.9)
A landline telephone	21 (7.2)	22 (7.8)	43 (7.5)
A desktop or laptop computer	67 (23.0)	48 (17.0)	115 (20.0)
A refrigerator	248 (85.2)	240 (84.8)	488 (85.0)
A vacuum cleaner or floor pol	49 (16.8)	35 (12.4)	84 (14.6)
A microwave oven	198 (68.0)	189 (66.8)	387 (67.4)
An electric or gas stove	254 (87.3)	238 (84.1)	492 (85.7)
A washing machine	153 (52.6)	136 (48.1)	289 (50.3)
Total number of assets: mean (SD)	5.14 (1.96)	4.94 (1.77)	5.04 (1.87)
**In the past month, number of days you or people in the household went to bed hungry because there was no food to eat**			
0 days	244 (83.8)	238 (84.1)	482 (84.0)
1-7 days	45 (15.5)	34 (12.0)	79 (13.8)
More than 7 days	2 (0.7)	9 (3.2)	11 (1.9)
Declined to answer	0 (0.0)	2 (0.7)	2 (0.3)
**Number of pre-treatment smear tests recorded?**			

One	197 (92.1)	169 (87.6)	366 (89.9)
Two	17 (7.9)	24 (12.4)	41 (10.1)
**Number of Gene XPert results recorded**			
One	225 (97.8)	210 (97.2)	435 (97.5)
Two	5 (2.2)	6 (2.8)	11 (2.5)
**Number of culture results recorded on the TB Treatment record**			
One	54 (94.7)	42 (97.7)	96 (96.0)
Two	3 (5.3)	1 (2.3)	4 (4.0)
**Co-morbidities**			
Hypertension	19 (6.93)	11 (4.1)	30 (5.54)
Diabetes	5 (1.84)	4 (1.49)	9 (1.66)
Epilepsy	3 (1.09)	4 (1.49)	7 (1.29)
Mental illness	3 (1.09)	0 (0)	3 (0.55)
Liver disease	1 (0.36)	1 (0.38)	2 (0.37)
Renal insufficiency	1 (0.36)	1 (0.38)	2 (0.37)
Allergies	2 (0.76)	0 (0)	2 (0.38)
Other	1 (0.36)	1 (0.38)	2 (0.37)
**Depression score (CESD 10): mean (SD)**	8.44 (4.38)	8.74 (4.8)	8.59 (4.59)

*Counts and percentages unless otherwise indicated

For peer review only

Supplementary table 2 Detailed descriptive statistics for smoking history, alcohol history at baseline by study arm

	Control n (%)*	Intervention n (%)*	Total n (%)*
Smoking History (current smokers only) N=	181	191	372
**On the days that you smoke, how soon after you wake up do you have your first cigarette?**			
After 60 minutes	30 (16.6)	28 (14.7)	58 (15.6)
31-60 minutes	24 (13.3)	16 (8.4)	40 (10.8)
6- 30 minutes	60 (33.1)	65 (34.0)	125 (33.6)
Within 5 minutes	67 (37.0)	82 (42.9)	149 (40.1)
**Duration of smoking in months: mean (SD)**	212.09 (134.03)	224.93 (127.82)	218.68 (130.86)
**Duration of smoking in months: median (IQR)**	186 (110, 282)	206 (135, 294)	200.5 (123, 287)
**Age started smoking in years: mean (SD)**	19.2 (6.3)	19.3 (6.3)	19.3 (6.3)
**Age started smoking in years: median (IQR)**	18 (15-20)	18 (16-21)	18 (15.5-20.5)
**Form of tobacco used**			
**Manufactured cigarettes (Yes)**	166 (91.7)	179 (93.7)	345 (92.7)
Number of days you smoked in the past 7days: mean (SD)	5.3 (2.65)	5.53 (2.33)	5.42 (2.49)
Average number of cigarettes smoked daily: mean (SD)	6.18 (6.43)	6.48 (8.21)	6.34 (7.39)
**Hand-rolled cigarettes (Yes)**	14 (7.7)	21 (11.0)	35 (9.4)
Number of days you smoked in the past 7days: mean (SD)	4.71 (2.84)	3.71 (2.95)	4.11 (2.91)
Average number of handrolled cigarettes smoked daily: mean (SD)	3.71 (3.97)	3.81 (2.82)	3.77 (3.27)
**Pipe (Yes)**	4 (2.2)	2 (1.0)	6 (1.6)
Number of days in the past 7days you smoked: median (IQR)	1 (0, 2.5)	4.5 (2, 7)	2 (0, 3)
Average number of daily sessions: median (IQR)	1 (0, 3.5)	3 (1, 5)	1.5 (0, 5)
Length of one session (on average) in **minutes**: median (IQR)	90 (60, 107.5)	60 (30, 90)	90 (30, 90)
**Cigars, cheroots or cigarillos (Yes)**	1 (0.55)	0 (0)	1(0.27)
**Water pipe (Yes)**	3 (1.7)	2 (1.0)	5 (1.3)
**Other**	10 (5.5)	4 (2.1)	14 (3.8)
**Heaviness of smoking index >= 4**	134(74.03)	158(82.72)	292(78.49)
Smoking inside your home restrictions			
Total: Not allowed	96 (53.0)	108 (56.5)	204 (54.8)
Some rules: where/when it is allowed	61 (33.7)	58 (30.4)	119 (32.0)
No rules	24 (13.3)	25 (13.1)	49 (13.2)

For peer review only

Attempts to quit smoking (current smokers only)			
**Ever attempted to quit in the past (Yes)**	52 (28.7)	64 (33.5)	116 (31.2)
Number of attempts to quit: mean (SD)	2.46 (2.98)	2.64 (1.62)	2.56 (2.32)
Time elapsed since attempt to quit last time, in months: mean (SD)	36.65 (94.62)	25.13 (37.91)	30.29 (69.22)
Longest duration abstinent in previous quit attempts: mean (SD)	6.15 (13.48)	4.22 (8.9)	5.09 (11.18)
**Likelihood to TRY TO QUIT smoking completely and permanently in the next three months**			
Definitely will not	6 (3.3)	5 (2.6)	11 (3.0)
Probably will not	10 (5.5)	12 (6.3)	22 (5.9)
Probably will	104 (57.5)	109 (57.1)	213 (57.3)
Definitely will	61 (33.7)	65 (34.0)	126 (33.9)
**Likelihood that I WILL QUIT smoking completely and permanently in the next three months**			
Definitely will not	6 (3.3)	5 (2.6)	11 (3.0)
Probably will not	11 (6.1)	13 (6.8)	24 (6.5)
Probably will	103 (56.9)	104 (54.5)	207 (55.6)
Definitely will	61 (33.7)	69 (36.1)	130 (34.9)
**Ever used any methods to help you stop smoking tobacco in the past 3-months?** (Yes)	23 (12.7)	16 (8.4)	39 (10.5)
Smokeless tobacco use (all participants)			
**In the past month, have you used smokeless tobacco (Snuff) on a daily basis**			
Not at all	275 (94.5)	275 (97.2)	550 (95.8)
Daily	12 (4.1)	6 (2.1)	18 (3.1)
Less than Daily	4 (1.4)	2 (0.7)	6 (1.0)
**Duration of using ST in months: mean(SD)**	113.69 (112.7)	152 (91.16)	126.46 (105.61)
**Age started using ST in years: mean (SD)**	27.56 (10.57)	25.88 (13.43)	27 (11.33)
**Form of ST used (for SLT users)**			
Snuff (by mouth)	2 (12.5)	0 (0.0)	2 (8.3)
Snuff (by nose)	11 (68.8)	5 (62.5)	16 (66.7)
Chewing tobacco leaves	0(0)	0(0)	0(0)
Other	1 (6.3)	0 (0.0)	1 (4.2)
**Help to stop drinking (N= , had a drink in past 12 months)**	208	223	431
**Ever used any methods to stop drinking alcohol in the past 3-months**	21 (11.2)	22 (10.7)	43 (10.9)

* Frequencies and (percentages) are presented unless otherwise stated

Supplementary table 3 Detailed descriptive statistics for primary outcome by study arm at 6-months

TB treatment status detailed	Control n (%)	Intervention n (%)	Total n (%)
Cured	108 (37.1)	105 (37.1)	213 (37.1)
Treatment completed	96 (33.0)	87 (30.7)	183 (31.9)
Treatment interrupted > 2 months	15 (5.2)	29 (10.2)	44 (7.7)
Treatment failure	5 (1.7)	2 (0.7)	7 (1.2)
Acquired drug resistance	1 (0.3)	4 (1.4)	5 (0.87)
Died	11 (3.8)	15 (5.3)	26 (4.5)
Transfer out	8 (2.75)	11 (3.9)	19 (3.31)
Unknown	42 (14.4)	25 (8.8)	67 (11.7)

For peer review only

Supplementary table 4 MI treatment fidelity scores of counselling sessions delivered by 17 lay health workers*

Ratings	Mean (SD)	Range
**Global ratings****		
**Cultivating change talk**	3.2 (1.17)	1-5
**Softening sustain talk**	3.4 (0.96)	1-4
**Partnership**	3.4 (1.12)	2-5
**Empathy**	2.9 (1.34)	1-5
**Behaviour counts[&]**		
**Giving information**	4.8 (8.13)	0-5
**Persuade**	0.8 (1.48)	0-5
**Persuade with permission**	0.1 (0.24)	0-1
**Question**	24.2 (10.42)	12-51
**Simple reflection**	4.1 (3.21)	0-10
**Complex reflection**	1.6 (2.69)	0-9
**Affirm**	5.5 (3.47)	1-12
**Seeking collaboration**	2.4 (1.46)	0-6
**Emphasising autonomy**	1.4 (1.37)	0-4
**Confront**	0.4 (0.74)	0-3
**Summary measures**		
**Total MI non-adherent †**	1.2 (2.28)	0-7
**Total MI adherent†**	9.3 (4.74)	1-16
**Technical global ratings****	3.3 (0.97)	1.5-4.5
**Relational global ratings****	3.1 (1.19)	1.5-5
**Reflection to question ratio††**	0.23 (0.24)	0-0.83
**Percentage of complex reflections*****	20.4 (21.9)	0-67

*The recordings of 17 counsellors (one each) were transcribed verbatim and then assessed. In order to assess the fidelity of the counsellors' delivery of motivational interviewing during the trial, we are used the validated Motivational Interviewing Treatment Integrity Coding Manual 4.2.1 (MITI) tool. The table shows the results of the fidelity assessment that was conducted by one rater who is proficient in English, seSotho and seTswana and in motivational interviewing. The rater listened to the recordings and coded a randomly selected 20-min portion of the written transcript. In the case of shorter counselling sessions, the entire recording was assessed.

**The "global ratings" involve assessing, on a 5-point Likert scale (1 for low and 5 for high), how well or poorly the counsellor adheres to the MI practice. Ratings are conducted on four items, two each making up Technical Components (Cultivating change talk and Softening sustain talk) and Relational Components (Partnership and Empathy).

**Scores on Cultivating Change Talk and Softening Sustain Talk are averaged to obtain the Technical global scores.

***Scores on Partnership and Empathy are averaged to obtain Relational global scores. The basic competency threshold scores for fair and good proficiency are 3 and 4, respectively for Technical scores and 3.5 and 4, respectively for Relational scores.

[&]The "behaviour counts" involve counting 10 verbal behaviours of the counsellor during the intervention.

† "MI adherence" is determined by adding up the following verbal behaviours: Seeking Collaboration, Affirm and Emphasising autonomy. "MI non-adherence" is determined by summing instances of Confront and Persuade. No thresholds for MI adherence or non-adherence are specified in the MITI 4.2.1.

††The Reflection-to-Question (R:Q) ratio is the total reflections divided by the total questions asked. One reflection to each question is considered a "fair" practice level while two reflections to each question is considered a "good" practice level.

*** The Percentage of Complex Reflections (% CRs) is calculated by dividing the number of complex reflections by the sum of complex reflections and simple reflections. A fair and a good % CRs are 40% and 50%, respectively.

For peer review only

Supplementary table 5 SMS delivery

		Intervention (N=283)		Control (N=291)
No. of participants who received ALL due IMB messages INDEPENDENT OF WHETHER they completed MI 1		99/283 (35%)		1/291 (0.3%)
**Completion of first MI and initiation of SMS-sequence**				
Completed first MI		227/283 (80.2%)		0/291 (0%)
No. of participants who received ALL due messages after receipt of MI1		95/227 (41.9%)		0/0 (0%)
**SMS delivery for participants for whom the SMS-sequence was initiated (after receipt of first MI)**				
	Mean (SD)	Median (IQR) Range	Mean (SD)	Median (IQR) Range
Average no. adherence messages received per participant (n=227)	7.9 (3.5)	10 (8-10) 0-11	0	0
Average no. tobacco-related messages received (n=153)	5.4 (2.5)	7 (5-7) 0-7	0	0
Average no. alcohol related messages received (n=171)	5.5 (2.5)	7 (5-7) 0-7	0	0
Average no. IMB messages received (n= 227)	15.7 (7.3)	17 (13-22) 0-25		

For peer review only - http://bmjopen.bmj.com/site/about/guidelines.xhtml

**Supplementary table 6** Unit costs used in the analysis (Presented in Rand as sources, $1=R14.448 in 2019)	
**Item**	Unit costs
**Personnel**	LHWs: R3000/month, 160 hours/month, R18.75/hour; District coordinators: R7500/month, 160 hours/month, R46.88/hour; MRC appointed supervisor: R205.79/hour; Trainers: R373.17/hour; Administrative staff: R333.24/hour
**Materials**	Training manual: R3250; Printing: R3000; Additional printing & stationary: R500
**Accommodation / Travel / Refreshments**	LHWs accommodation: R540 000; Trainers accommodation/travel: R36 000; Supervision accommodation/travel: R30 000; Refreshment: R12 500
**SMS system**	Monthly subscription at R433.44 ($30) for 3-months: R1300
**MI sessions**	Session 1 (17 minutes): R5.31; Session 2 (16 minutes): R5.00; Session 3 (17 minutes): R5.31
**Biochemical investigations**	Cost of TB smear microscopy: R28.37; culture: R79.22 and GeneXpert: R201.56 (Source: 2019/20 National Health Laboratory Service pricing schedule)*
**ART**	Atroiza: R3.78/dose; Dumiva: R5.55/dose; Tenemine: R2.34/dose; Zovilam: R1.72/dose; Kavimun: R1.89/dose; Ricovir: R1.36/dose; Zidomat: R1.38/dose; Lazena: R0.55/dose; Efrin:R0.63/dose; Efamat: R0.63/dose; Acriptaz: R0.61/dose (Source: Western Cape Department of Health. Antiretroviral and TB Stockmaster Worksheet. 2019)
**TB medication**	Month's supply of RHZE (intensive) @ R65.80; RH (continuation) @ R55.56 (Source: Western Cape Department of Health. Antiretroviral and TB Stockmaster Worksheet. 2019)

For peer review only

CONSORT 2010 checklist of information to include when reporting a randomised trial*

Section/Topic	Item No	Checklist item	Reported on page No
**Title and abstract**			
	1a	Identification as a randomised trial in the title	1
	1b	Structured summary of trial design, methods, results, and conclusions (for specific guidance see CONSORT for abstracts)	2
**Introduction**			
Background and objectives	2a	Scientific background and explanation of rationale	4-5
	2b	Specific objectives or hypotheses	5-7
**Methods**			
Trial design	3a	Description of trial design (such as parallel, factorial) including allocation ratio	5-6
	3b	Important changes to methods after trial commencement (such as eligibility criteria), with reasons	NA
Participants	4a	Eligibility criteria for participants	5
	4b	Settings and locations where the data were collected	5
Interventions	5	The interventions for each group with sufficient details to allow replication, including how and when they were actually administered	6
Outcomes	6a	Completely defined pre-specified primary and secondary outcome measures, including how and when they were assessed	7-8
	6b	Any changes to trial outcomes after the trial commenced, with reasons	NA
Sample size	7a	How sample size was determined	8
	7b	When applicable, explanation of any interim analyses and stopping guidelines	NA
Randomisation:			
Sequence generation	8a	Method used to generate the random allocation sequence	6
	8b	Type of randomisation; details of any restriction (such as blocking and block size)	6
Allocation concealment mechanism	9	Mechanism used to implement the random allocation sequence (such as sequentially numbered containers), describing any steps taken to conceal the sequence until interventions were assigned	6
Implementation	10	Who generated the random allocation sequence, who enrolled participants, and who assigned participants to interventions	6
Blinding	11a	If done, who was blinded after assignment to interventions (for example, participants, care providers, those	6

For peer review only - http://bmjopen.bmj.com/site/about/guidelines.xhtml

		assessing outcomes) and how	
	11b	If relevant, description of the similarity of interventions	NA
Statistical methods	12a	Statistical methods used to compare groups for primary and secondary outcomes	9
	12b	Methods for additional analyses, such as subgroup analyses and adjusted analyses	9
**Results**			
Participant flow (a diagram is strongly recommended)	13a	For each group, the numbers of participants who were randomly assigned, received intended treatment, and were analysed for the primary outcome	10, Figure 1
	13b	For each group, losses and exclusions after randomisation, together with reasons	10, Fig 1
Recruitment	14a	Dates defining the periods of recruitment and follow-up	5-6
	14b	Why the trial ended or was stopped	10,21
Baseline data	15	A table showing baseline demographic and clinical characteristics for each group	Table 1 and 2, Supp Table 1
Numbers analysed	16	For each group, number of participants (denominator) included in each analysis and whether the analysis was by original assigned groups	Fig 1, Table 3
Outcomes and estimation	17a	For each primary and secondary outcome, results for each group, and the estimated effect size and its precision (such as 95% confidence interval)	Table 3,4,5
	17b	For binary outcomes, presentation of both absolute and relative effect sizes is recommended	Only relative effect
Ancillary analyses	18	Results of any other analyses performed, including subgroup analyses and adjusted analyses, distinguishing pre-specified from exploratory	Table 4, 5
Harms	19	All important harms or unintended effects in each group (for specific guidance see CONSORT for harms)	No harms reported
**Discussion**			
Limitations	20	Trial limitations, addressing sources of potential bias, imprecision, and, if relevant, multiplicity of analyses	21-22
Generalisability	21	Generalisability (external validity, applicability) of the trial findings	NA, no effect
Interpretation	22	Interpretation consistent with results, balancing benefits and harms, and considering other relevant evidence	21
**Other information**			
Registration	23	Registration number and name of trial registry	2
Protocol	24	Where the full trial protocol can be accessed, if available	Published paper referenced

For peer review only

Funding	25	Sources of funding and other support (such as supply of drugs), role of funders	23

*We strongly recommend reading this statement in conjunction with the CONSORT 2010 Explanation and Elaboration for important clarifications on all the items. If relevant, we also recommend reading CONSORT extensions for cluster randomised trials, non-inferiority and equivalence trials, non-pharmacological treatments, herbal interventions, and pragmatic trials. Additional extensions are forthcoming: for those and for up to date references relevant to this checklist, see www.consort-statement.org.

For peer review only

BMJ Open

Effect of a brief motivational interview and text message intervention targeting tobacco smoking, alcohol use, and medication adherence to improve tuberculosis treatment outcomes in adult patients with tuberculosis: a multicentre, randomised controlled trial of the ProLife programme in South Africa

Journal:	*BMJ Open*
Manuscript ID	bmjopen-2021-056496.R1
Article Type:	Original research
Date Submitted by the Author:	25-Nov-2021
Complete List of Authors:	Louwagie, Goedele; Sefako Makgatho Health Sciences University, Research, Postgraduate Studies and Innovation; University of Pretoria, School of Health Systems and Public Health<br>Kanaan, Mona; University of York, Department of Health Sciences<br>Morojele, Neo; University of Johannesburg, Department of Psychology; Medical Research Council of South Africa, Alcohol, Tobacco and Other Drug Research Unit<br>Van Zyl, Andre; Sefako Makgatho Health Sciences University<br>Moriarty, Andrew; University of York, Hull York Medical School<br>Li, Jinshuo; University of York, Department of Health Sciences<br>Siddiqi, Kamran; University of York, Department of Health Sciences and the Hull York Medical School<br>Turner, Astrid; University of Pretoria, School of Health Systems and Public Health<br>Mdege, Noreen; University of York, Department of Health Sciences<br>Omole, Olufemi; University of the Witwatersrand, Department of Family Medicine<br>Tumbo, John; Sefako Makgatho Health Sciences University, Department of Family Medicine<br>Bachmann, Max; University of East Anglia, Norwich Medical School<br>Parrott, Steve; University of York, Department of Health Sciences<br>Ayo-Yusuf, Olalekan; Sefako Makgatho Health Sciences University, Africa Centre for Tobacco Industry Monitoring & Policy Research; University of Pretoria, School of Health Systems and Public Health
**Primary Subject Heading**:	Public health
Secondary Subject Heading:	Health economics, Infectious diseases, HIV/AIDS, Smoking and tobacco, Health services research
Keywords:	Tuberculosis < INFECTIOUS DISEASES, Public health < INFECTIOUS DISEASES, HIV & AIDS < INFECTIOUS DISEASES, Substance misuse < PSYCHIATRY, Health economics < HEALTH SERVICES ADMINISTRATION & MANAGEMENT, Organisation of health services < HEALTH SERVICES ADMINISTRATION & MANAGEMENT

SCHOLARONE™
Manuscripts

I, the Submitting Author has the right to grant and does grant on behalf of all authors of the Work (as defined in the below author licence), an exclusive licence and/or a non-exclusive licence for contributions from authors who are: i) UK Crown employees; ii) where BMJ has agreed a CC-BY licence shall apply, and/or iii) in accordance with the terms applicable for US Federal Government officers or employees acting as part of their official duties; on a worldwide, perpetual, irrevocable, royalty-free basis to BMJ Publishing Group Ltd ("BMJ") its licensees and where the relevant Journal is co-owned by BMJ to the co-owners of the Journal, to publish the Work in this journal and any other BMJ products and to exploit all rights, as set out in our licence.

The Submitting Author accepts and understands that any supply made under these terms is made by BMJ to the Submitting Author unless you are acting as an employee on behalf of your employer or a postgraduate student of an affiliated institution which is paying any applicable article publishing charge ("APC") for Open Access articles. Where the Submitting Author wishes to make the Work available on an Open Access basis (and intends to pay the relevant APC), the terms of reuse of such Open Access shall be governed by a Creative Commons licence – details of these licences and which Creative Commons licence will apply to this Work are set out in our licence referred to above.

Other than as permitted in any relevant BMJ Author's Self Archiving Policies, I confirm this Work has not been accepted for publication elsewhere, is not being considered for publication elsewhere and does not duplicate material already published. I confirm all authors consent to publication of this Work and authorise the granting of this licence.

For peer review only

Effect of a brief motivational interview and text message intervention targeting tobacco smoking, alcohol use, and medication adherence to improve tuberculosis treatment outcomes in adult patients with tuberculosis: a multicentre, randomised controlled trial of the ProLife programme in South Africa

Goedele Maria Louwagie[1, 2], Mona Kanaan[3], Neo Keitumetse Morojele[4, 5], André van Zyl[1], Andrew Stephen Moriarty[3], Jinshuo Li[3], Kamran Siddiqi[3], Astrid Turner[2], Noreen Dadirai Mdege[3], Olufemi Babatunde Omole[6], John Tumbo[1], Max Oscar Bachmann[7], Steve Parrott[3], Olalekan Ayo-Yusuf[1, 2]

Correspondence to Goedele Maria Louwagie; goedele.louwagie@up.ac.za

[1]Goedele Maria Louwagie, Sefako Makgatho Health Sciences University and [2]School of Health Systems and Public Health, University of Pretoria , RSA; goedele.louwagie@up.ac.za

[3]Mona Kanaan, Department of Health Sciences, University of York, UK; mona.kanaan@york.ac.uk

[4]Neo Keitumetse Morojele, Department of Psychology, University of Johannesburg and [5]Alcohol, Tobacco and Other Drug Research Unit, South African Medical Research Council, RSA; nmorojele@uj.ac.za

[1]André van Zyl, Sefako Makgatho Health Sciences University, RSA; drevanzyl@gmail.com

[3]Andrew Stephen Moriarty, Department of Health Sciences and the Hull York Medical School, University of York, UK; andrew.moriarty@york.ac.uk

[3]Jinshuo Li, Department of Health Sciences, University of York, UK; jinshuo.li@york.ac.uk

[3]Kamran Siddiqi, Department of Health Sciences and the Hull York Medical School, University of York, UK; kamran.siddiqi@york.ac.uk

[2]Astrid Turner, School of Health Systems and Public Health, University of Pretoria, RSA; astrid.turner@up.ac.za

[3]Noreen Dadirai Mdege, Department of Health Sciences, University of York, UK; noreen.mdege@york.ac.uk

[6]Olufemi Babatunde Omole, Department of Family Medicine, University of Witwatersrand, RSA; Olufemi.Omole@wits.ac.za

[1]John Tumbo, Department of Family Medicine, Sefako Makgatho Health Sciences University, RSA; tumbo@lantic.net

[7]Max Oscar Bachmann, Norwich Medical School, University of East Anglia, UK; m.bachmann@uea.ac.uk

For peer review only

[3]Steve Parrott, Department of Health Sciences, University of York, UK; steve.parrott@york.ac.uk

[1]Olalekan Ayo-Yusuf, Africa Centre for Tobacco Industry Monitoring & Policy Research, Sefako Makgatho Health Sciences University, and [2]School of Health Systems and Public Health, University of Pretoria, RSA; lekan.ayo-yusuf@smu.ac.za

ABSTRACT

Objectives: to investigate the effectiveness of a complex behavioural intervention, ProLife, on tuberculosis (TB) treatment success, medication adherence, alcohol use and tobacco smoking.

Design: multi-centre, individual, randomised controlled trial where participants were assigned (1:1) to the ProLife intervention or usual care.

Setting: 27 primary care clinics in South Africa.

Participants: 574 adults starting treatment for drug-sensitive pulmonary TB who smoked tobacco or reported harmful/hazardous alcohol use.

Interventions: The intervention, delivered by lay health workers (LHWs), consisted of 3 brief motivational interviewing (MI) sessions, augmented with Short Message Service (SMS) messages, targeting medication adherence, alcohol use and tobacco smoking.

Outcome measures: the primary outcome was successful versus unsuccessful TB treatment at 6 to 9 months, from TB records; secondary outcomes: biochemically confirmed sustained smoking cessation, reduction in the Alcohol Use Disorder Identification Test (AUDIT) score, improved TB and antiretroviral treatment (ART) adherence and ART initiation, each measured at 3 and 6 months by questionnaires; cure rates in patients who had bacteriology-confirmed TB at baseline, from TB records.

Results: Between 15 November 2018 and 31 August 2019, 574 participants were randomised to receive either the intervention (n=283) or usual care (n=291). TB treatment success rates did not differ significantly between intervention (67.8%) and control (70.1%) (OR=0.9 (95% CI: 0.64,1.27)). There was no evidence of an effect at 3- and 6-months respectively on continuous smoking abstinence (OR=0.65 (95% CI: 0.37,1.14); OR=0.76 (95% CI: 0.35,1.63)), TB medication adherence (OR=1.22 (95%CI: 0.52,2.87); OR=0.89 (95%CI: 0.26,3.07)), taking ART (OR=0.79 (0.38,1.65), OR=2.05 (0.80,5.27)) or AUDIT scores (mean score difference 0.55 (95% CI: -1.01,2.11); -0.04 (95% CI: -2,1.91); and adjusting for baseline values. Cure rates were not significantly higher (OR=1.16 (0.83,1.63)).

Conclusions: Simultaneous targeting of multiple health risk behaviours with MI and SMS using LHWs may not be an effective approach to improve TB outcomes.

For peer review only

For peer review only - http://bmjopen.bmj.com/site/about/guidelines.xhtml

Trial registration: ISRCTN62728852, Registered on 13th April 2018.

Key words: Tuberculosis, smoking, alcohol, motivational interviewing, mHealth, anti-retroviral therapy, adherence

STRENGTHS AND LIMITATIONS OF THIS STUDY

- The use of motivational interviewing combined with Short Text Messaging to address the effect of multiple risk behaviours (smoking, drinking and poor adherence) on tuberculosis treatment outcomes is a novel and much needed intervention.
- Our study design was strong: this was a multi-site individually randomised controlled trial with a large sample size and a high follow up rate for the primary outcome.
- We used validated measurement tools; furthermore data analysis and primary outcome assessment were blinded, thereby limiting measurement bias.
- The study was underpowered for secondary outcomes. Low intervention uptake may have diluted any potential intervention effects.

For peer review only

For peer review only - http://bmjopen.bmj.com/site/about/guidelines.xhtml

INTRODUCTION

Tuberculosis (TB) is among the most common chronic infectious diseases in the world today. In 2019, 1.4 million deaths worldwide were attributed to TB, and the majority of these occurred in low-income and middle-income countries (LMICs).(1) South Africa not only has one of the highest TB burdens in the world; it is faced with high TB treatment interruption and loss to follow-up rates. It also has a high prevalence of human immunodeficiency virus (HIV) co-infection in patients with TB and a relatively high mortality in these co-infected patients.(1) Studies of interventions to advance the goal of ending the TB epidemic and improving treatment outcomes are therefore research priorities in South Africa and other LMICs.(2)

Mortality and morbidity from TB is strongly associated with health risk behaviours, particularly smoking and hazardous or harmful alcohol use, both of which are prevalent and often co-occur in patients with TB.(3–10) Strategies are also required to improve TB medication adherence in patients with TB and adherence to TB medication and anti-retroviral therapy (ART) in patients co-infected with TB and HIV, both of which may be negatively influenced by excessive alcohol use.(1) There is very limited research on how to concurrently tackle these three risk behaviours - namely smoking, harmful alcohol use and poor medication adherence - in patients with TB, particularly in LMICs.

Motivational Interviewing (MI) has been shown to support reduced drinking, smoking cessation in TB patients and TB treatment and/or antiretroviral therapy (ART) medication adherence.(11–13) MI interventions can be effectively delivered by lay health workers (LHWs).(14) The more widespread use of LHWs and the increased use of mobile health (mHealth) digital technologies represent promising ways to increase the scalability of MI interventions. Indeed, the World Health Organisation (WHO) has called for researchers to capitalise on advances in mobile phone technology, network coverage and the increased use of common and widely available digital technologies (including the mobile phone short message service (SMS)) to improve TB care.(15) There is evidence that mHealth technologies can have modest beneficial effects on a range of health outcomes, including medication adherence.(16,17) Mobile phone messaging also shows a modest effect in improving TB treatment success rates.(18,19) The evidence is however stronger for two-way messaging and interactive systems for which smart phones are required.(18) These are often not available to patients with TB in Africa.(20)

A limitation of existing MI and mHealth interventions is that they have been studied in the context of modifying a single lifestyle factor. Integrated interventions are likely to be better accepted and more effective than multiple interventions targeting different health risk factors.(21,22) In the case of TB,

For peer review only - http://bmjopen.bmj.com/site/about/guidelines.xhtml

there is a need for an intervention that has the flexibility to target multiple lifestyle factors as appropriate and in line with patient preferences. This could be achieved through increased integration of TB and non-communicable disease services.(23)

Recent re-engineering of primary health care in South Africa has seen the introduction of municipal ward-based primary healthcare outreach teams of community health workers (CHWS). CHWs work in an integrated, team-based manner, supported by nurses, and take responsibility for health education and promotion, counselling and support for a range of health conditions.(24,25) Task shifting in this context has been shown to improve population health in LMICs (26) and these teams can be trained and supported to take responsibility for TB/HIV care.(27) Integrated interventions could be implemented within this framework in a feasible and scalable way to improve outcomes for patients with TB across South Africa and beyond.

Building on previous successes with MI and mHealth interventions, we developed a complex behavioural intervention (ProLife) comprising MI-based counselling and SMS, targeting three lifestyle risk behaviours for poor TB outcomes (smoking, hazardous/harmful alcohol consumption and poor medication adherence), and delivered by LHWs. We then conducted a randomised controlled trial (RCT) to assess the effectiveness of the ProLife intervention on improving TB treatment outcomes, smoking abstinence, reducing alcohol consumption and improving adherence to TB and ART medication compared to usual care. The cost-effectiveness of the intervention was also assessed but only the costing results will be presented in this paper.

METHODS

Study design and participants

This was a prospective, two-arm, multi-centre, individually RCT, which took place across 27 primary care clinics in 3 districts in South Africa (Lejweleputswa in the Free State province; Bojanala in the North West province; and Sedibeng in Gauteng province). Adult patients (18 years or older) were eligible for the study if they had drug-sensitive Pulmonary TB (PTB), and were initiating TB treatment or had been on TB treatment for less than a month for this treatment episode (both “new” and “retreatment patients”). They had to be tobacco smokers (defined as smoking daily or non-daily in the last four weeks on the Global Adult Tobacco Survey questionnaire)(28) and/or hazardous/harmful drinkers who were not alcohol dependent (Alcohol Use Disorders Identification Test (AUDIT) score ≥ 8 for men or ≥ 7 for women but <20).(29) They also had to have access to a

For peer review only

For peer review only - http://bmjopen.bmj.com/site/about/guidelines.xhtml

mobile phone and understand one of the 4 languages used for the trial (English, IsiZulu, SeSotho and Setswana). Potential participants were recruited consecutively at the participating clinics between 15 November 2018 and 31 August 2019. Trained field workers identified those interested in the study and screened them for eligibility. If eligible and willing to be enrolled into the trial, written informed consent was obtained. The RCT received ethics approval from the Research Ethics Committees of the five participating research institutions.(30)

Randomisation and blinding

Patients were centrally randomised (1:1) to the ProLife intervention or control group using a randomised sequence generator by the trial statistician (MK) who was blind to the arm allocation. We used block randomisation with varying block sizes stratified by clinic so as to achieve equal numbers in intervention and control groups within each clinic. Fieldworkers used sequentially numbered, sealed, opaque envelopes to allocate participants to intervention or control. ProLife involved a complex behavioural intervention therefore LHWs and participants could not be blinded to the intervention. However, the determination of the primary outcome was done by the TB nurses who were blinded to the intervention status of the participants, based on routinely collected data. The statistician (MK) was blinded to the intervention or control arm allocation of participants during the analysis.

Intervention and procedures

The ProLife intervention was developed based on a conceptual framework, following a review of pre-existing evidence.(31) This framework assumed that smoking cessation, reducing harmful alcohol use and improved adherence to TB and HIV treatment would result in improved TB treatment outcomes.(30) The intervention consisted of 3 brief MI counselling sessions, lasting 15-20 minutes, one month apart, delivered by trained LHWs at their TB clinic. The first MI session took place immediately or shortly after the randomisation and involved prioritisation and agenda setting, wherein the participant determined which factor should be prioritised (either a plan to quit tobacco smoking, or reduce or quit drinking, or deal with barriers relating to ART or TB medication adherence). The second and third sessions built on the previous one until all relevant behavioural problems had been addressed. These sessions were reinforced with follow-up SMS text messages, twice a week over 12 weeks.(30) Study patients received 10 TB-related messages followed by seven alcohol reduction- and/or seven smoking cessation-related messages, as appropriate. Messages were aimed at giving information, and augmenting motivation or behavioural skills (we refer to the feasibility paper for more details).(31)

For peer review only - http://bmjopen.bmj.com/site/about/guidelines.xhtml

Participants randomised to the ProLife intervention also received the same "usual care" as those in the control group. The control group received the usual care and routine treatment and support offered to patients with TB in South Africa, which varies by district but includes: health education; dietetic input; social support; point of care biochemical testing; and HIV testing with pre- and post-test HIV test counselling.

Data were collected at baseline, 3- and 6-months and recorded by fieldworkers equipped with mobile phones with the ProLife mobile data collection application (built with CommCare)(32) installed. They used a standardised electronic case report form (CRF) and followed standard operating procedures (SOPs) to ensure quality. Details of data collection, protection and storage procedures have been reported elsewhere.(30)

Patient and public involvement

Patients or the public were not involved in the design, or conduct, or reporting, or dissemination plans of our research.

Outcomes

Primary Outcome

The primary outcome of TB treatment success at 6 to 9 months follow-up (depending on when it was recorded) was as per the WHO definitions adopted in South Africa,(10) that is, either successful treatment (cured or treatment completed) or failed treatment, death, acquired drug resistance, loss to follow-up (defined as treatment interruption of more than 2 months), or outcome not evaluated. It was measured using the routinely collected TB treatment outcomes in patients' individual files.

Secondary Outcomes

For those participants with bacteriologically confirmed PTB at baseline (either sputum acid fast bacilli (AFB) positive, culture positive or GeneXpert positive PTB), sputum conversion at the end of treatment ("cure rate") was measured as a secondary outcome.(10) Continuous smoking abstinence was assessed at 3- and 6-months follow-up in those participants who were current cigarette smokers at baseline. It was defined as having quit smoking completely and a self-report of not smoking more than 5 cigarettes from the start of the study, in addition to a negative biochemical test (exhaled Carbon Monoxide (CO) < 7 ppm).(33,34) Changes in alcohol consumption were computed using the

For peer review only

For peer review only - http://bmjopen.bmj.com/site/about/guidelines.xhtml

AUDIT questionnaire scores measured at 3- and 6-months follow-up in those participants who were hazardous/harmful drinkers at baseline.

HIV positive participants were asked about ART status at baseline, 3- and 6-months using standardised questions on the CRF and change in ART status as measured at the 2 follow-up times.

TB and ART medication adherence was measured using modified versions of the AIDS Clinical Trials Group Adherence Questionnaire, a validated tool for measuring adherence specifically to ART.(35) Adherence was measured using an adherence index calculated by the formula (using the 4-day recall): [Total number of doses taken/Total number of doses prescribed] x 100. Patients with at least 95% adherence were classed as having optimal adherence and those with less than 95% were classed as having low (or suboptimal) adherence. This was assessed at 3- and 6-months.

During COVID-19 lockdown (in the second term of 2020) we switched to telephonic follow-up of participants using a shortened questionnaire whereby only strictly needed information for the measurement of outcomes was inquired about.

Training and Intervention Fidelity Monitoring

The training and intervention fidelity monitoring is described in more detail in previous papers.(30,31) In brief, 18 LHWs, 3 district coordinators and 1 research assistant who focused on counselling supervision underwent MI training over 5 days. LHWs completed a post-session semi-structured form onto which they indicated the extent to which they implemented each element of MI, as well as their general qualitative impressions of that particular session. In addition, we assessed MI intervention fidelity based on ratings of the counsellors' recorded MI sessions, as described below and in footnotes to the relevant supplementary table. SMS-message delivery was also assessed.

Economic evaluation

The ProLife intervention costs consisted of the costs of training and the delivery of the ProLife intervention, including relevant personnel involvement (trainers and LHWs), materials used, travel, accommodation and refreshments, and digital infrastructure for the intervention. These were estimated based on research team records. Usual care costs consisted of TB medication costs, biochemical investigations, and ART costs if applicable. These were estimated based on information obtained through routine records. The country specific version of Euroqol with 5 dimensions and 3 levels of response categories (EQ-5D-3L) for South Africa was administered to participants at baseline, 3- and 6-months follow-up to measure health-related quality of life.(36,37)

For peer review only - http://bmjopen.bmj.com/site/about/guidelines.xhtml

Statistical analysis

The sample size was estimated at 696 in total (348 participants per arm), to detect a 10% difference in TB treatment success rates (0.86 vs. 0.76) in the ProLife arm (intervention) vs. the control arm with 80% power, a significance level of 0.05 and 25% attrition rate. The assumed success rates in the control group were based on actual success rates in patients with TB in the studied provinces obtained from TB managers at the time of the grant application for this study.

We summarised baseline data descriptively by trial arm. For the primary outcome, we conducted statistical analysis on an intention-to-treat basis. We used binary logistic regression to compare the main outcome (TB treatment success rate) between the intervention and the usual care arm. Where treatment outcome data were missing, the outcome was coded as unsuccessful. TB treatment outcomes recorded by the TB nurse were taken on face value as inconsistencies in the dates of bacteriological results did not permit us to verify the correctness of the nurse assessment. We carried out similar statistical analyses for the secondary outcomes with appropriate regression techniques. For the reduction in harmful or hazardous drinking, we used linear regression to estimate the difference in total AUDIT score between control and intervention groups accounting for the baseline AUDIT score as covariate. Separate analyses at 3- and 6-months were performed.

For our main analyses we adjusted for baseline characteristics if these differed between trial arms at baseline. The covariates that we controlled for in each model are specified when a model is presented. The statistical packages STATA (38) and R (39) were used to carry out the analyses, with a P-value < 0.05 considered statistically significant.

The validated Motivational Interviewing Treatment Integrity (MITI) coding tool (version 4.2.1) was used to assess MI intervention fidelity.(40) The coding entailed making 'Global Ratings' (on 4 dimensions: Cultivating change talk, Softening sustain talk, Partnership and Empathy) and 'Behaviour' counts (with respect to the items: giving information, persuade, persuade with permission, question, simple reflection, complex reflection, affirm, seeking collaboration, emphasising autonomy and confront). A score was assigned to each of these items and the scores compared against the competency and proficiency thresholds that are specified in the MITI manual.

For the analysis of the costs, all costs were collected in South Africa Rand (ZAR) except for the data management system subscription. Results are presented in both ZAR and US dollar ($) using the 2019 OECD exchange rate (1 USD = 14.448 Rand).(36) No South African specific valuation set was

For peer review only - http://bmjopen.bmj.com/site/about/guidelines.xhtml

available for EQ-5D-3L. The valuation set of Argentina, based on a Visual Analogue Scale, was used to derive utility values, because the GDP per capita in international dollars was the closest between the two countries at the time of analysis.(37,41) Quality-adjusted life years (QALYs) were derived from the utility values at the time points by calculating the area under the curve.(42) No missing data imputation was performed.

RESULTS

Participant enrolment and follow-up

A total of 2099 patients with TB were screened for eligibility out of which 574 consenting and eligible participants were randomised: 291 to control and 283 to intervention. Trial recruitment was terminated on 31 August 2019 before the planned sample size was reached because of budget and time constraints. In the intervention arm, 227 (80.2%) participants completed the first MI (MI 1) session, 199 (70.3%) MI 2 and 150 (53.0%) MI 3. In the intervention arm, at least one message was delivered to 208 (73.5%) participants, while 99 (35.0%) received all messages. Of those randomised to the control and intervention groups, the primary outcome was recorded in 244 (83.8%) and 253 (89.4%) participants, respectively. (Figure 1)

Figure 1 Consort flow diagram

Baseline participant characteristics of the intervention and control arm

Socio-demographic; socio-economic and clinical characteristics

Baseline characteristics were distributed similarly in the intervention and control arms for most variables but with some imbalances in educational level. A total of 513 (91.3%) participants were new patients with TB, 129 (22.5%) women, and nearly all had PTB (International Classification of Diseases-10 A15) without extrapulmonary TB manifestations (553 (98.9%)). About half of the participants were HIV positive (305 (53.2%)), of whom 204 (65.4%) were on cotrimoxazole and 257 (82.4%) were on ART. (Table 1) Details of marital status, employment, wealth, depression status and co-morbidities are presented in Supplementary table 1.

For peer review only - http://bmjopen.bmj.com/site/about/guidelines.xhtml

Table 1 Baseline descriptive socio-economic statistics and clinical characteristics by study arm

	Control (N = 291) n (%)*	Intervention (N = 283) n (%)*	Total (N=574) n (%)*
**Age in years:** mean (SD)	39.37 (12.60)	38.56 (11.15)	
**Female Sex**	69 (23.7)	60 (21.2)	129 (22.5)
**Education**			
No education	7 (2.4)	5 (1.8)	12 (2.1)
Grades 1-5	23 (7.9)	20 (7.1)	43 (7.5)
Grades 6-7	32 (11.0)	35 (12.4)	67 (11.7)
Grades 8-11	96 (33.0)	128 (45.2)	224 (39.0)
Grade 12	87 (29.9)	70 (24.7)	157 (27.4)
Higher	24 (8.2)	8 (2.8)	32 (5.6)
Declined to answer**	22 (7.6)	17 (6.0)	39 (6.8)
**TB Patient category**			
New patient	264 (92.3)	249 (90.2)	513 (91.3)
Relapse	10 (3.5)	9 (3.3)	19 (3.4)
Re-treatment after default	9 (3.1)	14 (5.1)	23 (4.1)
Re-treatment after failure	1 (0.3)	2 (0.7)	3 (0.5)
Other	2 (0.7)	2 (0.7)	4 (0.7)
**TB Site of disease** Pulmonary only (ICD-10 A15)	281 (98.9)	272 (98.9)	553 (98.9)
**TB Sputum smear, Gene XPert, or culture result available** N=	236	227	463
**At least one Sputum smear, Gene XPert, or culture result positive**	208 (88.1)	195 (85.9)	403 (87.0)
**HIV status**			
Negative	118 (40.7)	125 (44.2)	243 (42.4)
Positive	163 (56.2)	142 (50.2)	305 (53.2)
Unknown	9 (3.1)	16 (5.7)	25 (4.4)
**HIV positive patients**			
**Using Cotrimoxazole**	104 (63.8)	100 (67.1)	204 (65.4)
**Using anti-retroviral**	139 (85.3)	118 (79.2)	257 (82.4)

For peer review only - http://bmjopen.bmj.com/site/about/guidelines.xhtml

* Frequencies and (percentages) are presented unless otherwise stated
**More variables with the option "declined to answer" are listed in the Supplementary table 1

Table 2 Baseline descriptive alcohol and smoking characteristics by study arm

	Control (N=291) n (%)*	Intervention (N=283) n (%)*	Total (N=574) n (%)*
**In the past month, smoked tobacco**			
Not at all**	110 (37.8)	92 (32.5)	202 (35.2)
Daily	149 (51.2)	149 (52.7)	298 (51.9)
Less than Daily	32 (11.0)	42 (14.8)	74 (12.9)
**Had a drink in the past 12-months**	208 (71.5)	223 (78.8)	431 (75.1)
**AUDIT Score (males) : mean (SD) [max: 19]†**	12.27 (3.98)	13.02 (3.78)	12.66 (3.89)
**AUDIT Score (females): mean (SD) [max: 19]†**	11.32 (4.02)	10.98 (4.02)	11.15 (4.0)
**Hazardous/harmful drinking and Smoking Combined (Constructed)**			
Hazardous/harmful drinking Only‡	110 (37.8)	92 (32.5)	202 (35.2)
Smoking Only	103 (35.4)	77 (27.2)	180 (31.4)
Smoking and hazardous/harmful drinking‡	78 (26.8)	114 (40.3)	192 (33.4)

* Frequencies and (percentages) are presented unless otherwise stated
**Non-smokers were only included if they were harmful or hazardous drinkers
†Only hazardous/harmful drinkers and/or current smokers were included in the study. Therefore patients with TB were excluded if they were non-current smokers and had an AUDIT score <7 (females) or <8 (males) or >19; however, they were included if they were smokers independent of whether they had a drink in the past year and therefore independent of the AUDIT score. These AUDIT scores are thus representative of the mean AUDIT scores in the entire study sample and differ from the AUDIT score in the harmful/hazardous drinkers whose change in AUDIT score was measured at 3 and 6-months follow-up
‡Harmful/hazardous drinking is defined as Alcohol Use Disorders Identification Test (AUDIT) score ≥ 8 for men or ≥ 7 for women but <20

For peer review only - http://bmjopen.bmj.com/site/about/guidelines.xhtml

There were 372 current smokers (298 daily, 74 less than daily). Seventy eight participants (26.8 %) in the control arm were dual smokers *and* drinkers compared to 114 (40.3 %) in the intervention arm. In the control arm, 110 (37.8%) were hazardous/harmful drinkers only and 103 (35.4%) were smokers only, compared to 92 (32.5%) and 77 (27.2) respectively, in the intervention arm. (Table 2) More details of smoking and drinking history, forms of tobacco use, addiction and quit attempts are presented in Supplementary Table 2.

Primary outcome

Overall, 396 (70%) of participants were classified as treated successfully (treatment completed or cured). The remainder either interrupted treatment, failed treatment, developed drug resistance, were transferred out or had an unknown treatment outcome.(Supplementary table 3) The percentage of successful TB treatment did not differ significantly between the control and intervention arm (70.1% vs. 67.8%), odds ratio (OR) for successful TB treatment 0.90 (95%CI: (0.64,1.27)) comparing the intervention arm to the control arm; and was similar to adjusted ORs. (Tables 3 and 4)

Secondary outcomes

Cure rates

Among the 403 participants who had at least one positive bacteriological result at baseline, 168 (41.7%) were recorded as cured; of these, 83/205 (39.9%) were in the control arm compared to 85/195 (43.6%) in the intervention arm. The OR of being cured was 1.16 (95% CI: 0.83,1.63) in the intervention versus the control arm; and was similar to the adjusted OR. (Tables 3 and 4)

Continuous smoking abstinence

Among those who identified as cigarette smokers at baseline (345 (60.1%)), 27 had information (self-report plus biochemical verification) to enable the identification of continuous abstinence at 6-months of which 22 had continuously abstained from smoking. These were similarly distributed across the two study arms: 10 (5.59%) participants in the intervention arm compared to 12 (7.23%) in the control arm, OR 0.76 (95% CI: 0.35,1.63). (Tables 3 and 4) At the 3-month follow-up 20 (11.2%) participants in the intervention arm compared to 27 (16.3%) in the control arm continuously abstained from smoking, OR 0.65 (95% CI: 0.37,1.14). (Tables 3 and 5)

For peer review only - http://bmjopen.bmj.com/site/about/guidelines.xhtml

TABLE 3 Descriptive statistics for primary and secondary outcomes by study arm at baseline (where available), 3-months (where available) and 6-months									
	Baseline n (%)*			3-months follow-up n (%)*			6-months follow-up n (%)*		
	**Control**	**Intervention**	**Total**	**Control**	**Intervention**	**Total**	**Control**	**Intervention**	**Total**
**TB treatment status[a]**									
Successful**							204 (70.1)	192 (67.8)	396 (69.0)
Not Successful							87 (29.9)	91 (32.16)	178 (31.01)
**Cured [a], *** **									
Yes							83 (39.9)	85 (43.6)	168 (41.7)
No							125 (60.1)	110 (56.4)	235 (58.3)
**Continuous smoking abstinence[b]**									
Yes				27 (16.3)	20 (11.2)	47 (13.6)	12 (7.2)	10 (5.6)	22 (6.4)
No				139 (83.7)	159 (88.8)	298 (86.4)	154 (92.8)	169 (94.4)	323 (93.6)
**Harmful/hazardous drinkers[!]: N =**	188†	206†	394†	141†	130†	271†	112†	127†	239†
AUDIT score: mean (SD)	12.76 (3.42)	13.12 (3.47)	12.94 (3.45)	8.28 (6.18)	8.84 (5.38)	8.55 (5.81)	8.79 (6.66)	8.70 (5.83)	8.74 (6.22)
Difference from baseline: mean (SD)				-4.61 (6.26)	-4.07 (5.33)	-4.35 (5.83)	-4.25 (6.56)	-4.17 (6.61)	-4.21 (6.57)
**HIV positive patients**:	163† (56.2)	142† (50.2)	305† (53.2)	122†	83†	205†	100†	83†	183†
**Taking ART medication if HIV positive‡**	139 (85.3)	115 (81.0)	254 (83.3)	91 (74.6)	58 (69.9)	149 (72.7)	80 (80.0)	74 (89.2)	154 (84.2)

For peer review only - http://bmjopen.bmj.com/site/about/guidelines.xhtml

**ART medication adherence[b]**						
Optimal adherence	101 (99.0)	64 (98.5)	165 (98.8)	75 (97.4)	64 (97.0)	139 (97.2)
Suboptimal adherence	1 (1.0)	1 (1.54)	2 (1.2)	2 (2.6)	2 (3.0)	4 (2.8)
**TB medication adherence**						
Optimal adherence	181 (92.3)	138 (90.8)	319 (91.7)	61 (89.7)	59 (90.8)	120 (90.2)
Suboptimal adherence	15 (7.6)	14 (9.2)	29 (8.3)	7 (10.3)	6 (9.2)	13 (9.8)

a: only assessed at 6-months

b: assessed at 3 and 6-months; this table refers to *cigarette* smokers only (other forms of tobacco smoking are excluded)

* Frequencies and (percentages) are presented unless otherwise stated

**Primary Outcome: This is a binary variable defined as either successful treatment (cured or treatment completed) or failed treatment, death, acquired drug resistance, loss to follow-up or treatment interrupted for more than 2 months, or outcome not evaluated/unknown.

***Based on having a cured treatment outcome among those who were bacteriologically positive at baseline.

! hazardous/harmful drinkers who are not alcohol dependent= AUDIT score ≥ 8 for men or ≥ 7 for women but < 20; *! Important distinction at baseline for eligibility purposes.

†Denominator for the mean (SD) or denominator for %

‡ Information on HIV-positivity was obtained from information from TB records combined with patient self-report at baseline. True HIV-positivity rates may have been higher.

For peer review only

For peer review only - http://bmjopen.bmj.com/site/about/guidelines.xhtml

**TABLE 4** Regression analysis results for the primary and secondary outcomes at 6-months				
	Crude Odds Ratio (95% CI)*	P Value*	Adjusted Odds Ratio (95% CI)*	P Value*
**Primary outcome**				
TB treatment status: Successful (Ref: Not successful)	0.90 (0.64,1.27)	0.548	0.86‡ (0.60,1.24)	0.421
**Secondary outcomes**				
Cured (Ref: Not Cured)	1.16 (0.83,1.63)	0.374	1.07‡ (0.76,1.51)	0.684
Continuous smoking abstinence (Ref: No)***	0.76 (0.35,1.63)	0.482		
TB medication adherence (Ref: Optimal)	0.89 (0.26,3.07)	0.849		
ART medication adherence (Ref: Optimal)	1.17 (0.14,9.94)	0.884		
Taking ART medication (Ref: No)	2.05[!] (0.80,5.27)	0.136		
AUDIT	-0.04[**] (-2,1.91)	0.966	0.02[!!] (-1.55,1.6)	0.976

* Analyses accounted for potential clustering by centre.
‡ Adjusted for district, sex, and smoking/drinking status and HIV status at baseline. It is worth noting that of the variables in the adjusted model; the only statistically significant result is for the district variable.
! Adjusting for art status at baseline
**Controlling for the AUDIT baseline values. The values represent the study arm regression coefficient.
!! Controlling for the AUDIT baseline values and adjusted for district, sex, and smoking/drinking status and HIV status at baseline. The values represent the study arm regression coefficient.
***Given the limited number of those who were identified as continually abstained, we were only able to adjust for one additional variable at a time. Adding one of the following variables: heaviness of smoking, type of drinker at baseline, age when started smoking, and the duration of smoking at baseline; the adjusted odds ratio of continuous abstinence comparing the intervention to the control arm ranged between 0.73 and 0.76 with similar confidence limits as for the crude estimate.

For peer review only

TABLE 5 Regression analysis results for secondary outcomes measured at 3-months

Secondary outcome	Crude Odds Ratio (95% CI)*	P Value*	Adjusted Odds Ratio (95% CI)*	P Value*
Continuous smoking abstinence (Ref: No)**	0.65 (0.37,1.14)	0.135		
TB medication adherence (Ref: Optimal)	1.22 (0.52,2.87)	0.641		
ART medication adherence (Ref: Optimal)	1.58 (0.10,26.12)	0.750		
Taking ART medication (Ref: No)	0.79! (0.38,1.65)	0.53	0.74‡ (0.35,1.58)	0.443
AUDIT	0.55† (-1.01,2.11)	0.474	0.74!! (-0.62,2.1)	0.273

*Analyses accounted for clustering.
! Adjusting for art status at baseline
‡ Adjusted for art status at baseline, district, sex, and smoking/drinking status and HIV status at baseline.
†Controlling for the AUDIT baseline values. The values represent the study arm regression coefficient.
!! Controlling for the AUDIT baseline values and adjusted for district, sex, and smoking/drinking status and HIV status at baseline. The values represent the study arm regression coefficient.
** Given the limited number of those who were identified as continually abstained, we were only able to adjust for one additional variable at a time. Adding one of the following variables: heaviness of smoking, type of drinker at baseline, age when started smoking, and the duration of smoking at baseline; the adjusted odds ratio of continuous abstinence comparing the intervention to the control arm ranged between 0.63 and 0.66 with similar confidence limits as for the crude estimate.

For peer review only

For peer review only - http://bmjopen.bmj.com/site/about/guidelines.xhtml

Change in harmful/hazardous drinking

AUDIT scores were about 4 points lower at both follow-up times than at baseline, independent of the intervention.(Table 3) In the intervention arm, participants had, on average, a reduction of 0.04 points (95% CI: -2,1.91) on the AUDIT score at 6-months, compared to those in the control arm controlling for baseline scores; whereas an average increase of 0.55 (95% CI: -1.01,2.11) was observed at 3-months. (Tables 4 and 5)

Medication adherence and ART uptake

At 6-months, the OR of taking ART medication was 2.05 (95% CI: 0.80,5.27) comparing the intervention arm to the control arm and controlling for ART baseline medication status; whereas it was 0.79 (95% CI: 0.38,1.65) at 3-months. The proportion of participants who had optimal TB medication adherence was 90.2% (120/133) at 6-months and 91.7% (319/348) at 3-months. Suboptimal TB medication adherence ORs were 0.89 (95%CI: 0.26,3.07) and 1.22 (95%CI: 0.52,2.87) comparing intervention arm to the control arm at 6-months and 3-months, respectively. The proportions of participants on ART who had optimal ART medication adherence were high at both 3-months (165/167, 98.8%) and 6-months follow-up (139/143, 97.2%). Suboptimal ART medication adherence ORs were 1.17 (95%CI: 0.14,9.94) and 1.58 (95%CI: 0.10,26.12) comparing the intervention arm to the control arm at 6-months and 3-months, respectively. (Tables 3, 4 and 5)

Intervention fidelity

Motivational interviewing fidelity

The recordings of 17 counsellors (one each) were transcribed verbatim and then assessed. In terms of the global ratings, the LHWs' counselling sessions were above proficiency levels on all items, namely, cultivating change talk, softening sustain talk, partnership, and empathy (as the mean scores were all above 2). In terms of the summary measures, the LHWs' counselling sessions did not achieve the basic proficiency threshold of 3.5 for the Relational component (Partnership + Empathy) as their mean score was 3.1 (SD=1.19). However, their mean score on the Technical component (Cultivating Change Talk + Softening Sustain Talk) of 3.3 (SD=0.97) was above the threshold of 3. For behavioural counts, "Asking Questions" had the highest mean score (24.2; SD=10.42), followed by "Affirm" with a mean score of 5.5 (SD=3.7). The counsellors were least likely to engage in the following: Persuade with permission and emphasising autonomy. The mean Reflections to Questions Ratio was 0.23 (SD=0.24). The LHWs made on average 9.3 (SD=4.74) MI adherent (Affirm, Emphasize Autonomy and Seek Collaboration) and 1.2 (SD=2.28) MI non-adherent (Confront and Persuade) statements per session. (Supplementary Table 4)

For peer review only - http://bmjopen.bmj.com/site/about/guidelines.xhtml

SMS-delivery

Of the total number of Information-Motivation-Behaviour messages triggered, 3583 (80.4%) were delivered. All due SMS messages were delivered to 95 (41.9%) of the participants who completed the first MI, See Supplementary Table 5 for more details.

Costs and health related quality of life

Unit costs used to estimate the mean costs are presented in the Supplementary Table 6. Incremental cost-utility ratios are not presented since the intervention was not clinically effective. The mean cost of the ProLife intervention was ZAR 2601 (SD 6) ($180.02 (SD $0.42)) per participant in the intervention arm (n=283). The mean cost of usual care was ZAR 681 (SD 357) ($47.13 (SD $24.71)) in the intervention arm (n=122) versus ZAR 706 (SD 302) ($48.86 (SD $20.90)) in the control arm (n=131). The total mean cost of care including the intervention was ZAR 3285 (SD R357) ($227.37 (SD 24.71)) in the intervention arm (n=122). EQ-5D-3L data were available at the 3 time points for 137 intervention and 159 control arm participants. The mean QALYs estimated over six months were 0.442 (SD 0.061) in the intervention arm versus 0.430 (SD 0.074) in the control arm (adjusted mean difference 0.006 (95% CI -0.001 to 0.013)).

DISCUSSION

This RCT did not provide evidence for improved TB treatment success rates in those receiving the ProLife intervention compared to those receiving usual care. We could also not demonstrate significant beneficial effects on any of the secondary outcomes, i.e., smoking, alcohol consumption, medication adherence and ART initiation. To our knowledge there are no other published studies of similar complex interventions that aim to improve TB treatment outcomes in patients who smoke or drink to harmful or hazardous extent. Interventions evaluated by other studies were either complex interventions or SMS-based interventions aimed at improving TB outcomes through the pathway of increasing adherence, but without an alcohol or smoking intervention component (43,44) or focused on a single behaviour, namely smoking or drinking.(45,46). Of the latter studies, a brief smoking cessation intervention was effective in inducing smoking cessation in patients with TB but did not improve TB outcomes.(45) Conversely, in another study in India, intensive counselling for alcohol disorders led to significantly better TB treatment outcomes in the intervention group compared to the control group.(46) Smoking cessation also led to better TB treatment outcomes in a secondary analysis of a large tobacco cessation trial in patients with TB in Bangladesh and Pakistan.(47) Our

For peer review only - http://bmjopen.bmj.com/site/about/guidelines.xhtml

non-significant result for smoking-related outcomes is not consistent with findings from our previous TB study, which used a single MI session and found that the chance of sustained smoking cessation was twice as high in the MI intervention group compared to the control group,(14) albeit with a less stringent exhaled CO cut-off point. Evidence on the effectiveness of MI for smoking abstinence in non-TB settings has been equivocal.(48) Self-reported alcohol consumption decreased with about 4 points in both intervention and control arms in our study at both follow-up times. Answering questions on drinking in brief intervention trials may alter subsequent self-reported behaviour: exposing non-intervention control groups to an integral component of the intervention may therefore underestimate the effect of the intervention.(49) There have been few previous studies looking at MI and SMS interventions for the modification of hazardous/harmful drinking in the context of TB. A previous trial of a brief counselling intervention to reduce alcohol consumption in patients with TB did not find a significant effect on alcohol reduction.(50) Outside a TB setting, results have been mixed. A meta-analysis showed a small but significant improvement in outcomes when MI was used in conjunction with cognitive behavioural therapy for co-morbid alcohol use and depression.(51) Self-reported TB and ART medication adherence was high overall in our study population, which is consistent with other studies conducted in South Africa.(52,53) It is possible that we did not find a difference in treatment adherence due to a ceiling effect.

There were several key strengths in this RCT. This was an individually RCT with a relatively large sample size and a high follow up rate (87%) for the primary outcome. Primary outcome assessment was blinded. This was a novel intervention, which built on previous successes with both MI and mHealth interventions and was aligned with the WHO's call to increase the use of digital technologies to improve TB care.(15) We utilized a validated alcohol consumption questionnaire (AUDIT) (29) and a four-day timeline follow-back for medication adherence to reduce recall bias as self-reports tend to underreport drinking while overestimating adherence behaviour compared with.(54) (35) Smoking cessation was confirmed with exhaled CO using strict cut-off points. Overall, the quality of the counselling was acceptable. The results of our MI analyses suggest that the LHWs trained as counsellors were more proficient in MI than during the feasibility stage, as observed by their global rating scores on cultivating change talk, softening sustain talk, partnership and empathy. (Supplementary Table 4) These results were achieved by ongoing monitoring and training of LHWs during the trial and adapting the training based on feedback from the feasibility stage. Extra counsellors were also appointed to minimise travel distances to clinics. There were some limitations associated with this RCT. Trial recruitment had to be terminated before the planned sample size because of funding and time constraints. Nevertheless the calculation of sample size was based on

For peer review only - http://bmjopen.bmj.com/site/about/guidelines.xhtml

an anticipated 25% LTFU for the primary outcome, while in reality only 13.4 %, of the TB outcomes were not available. As a result, we achieved a slightly higher power to detect the a 10% difference in primary outcomes, than we had aimed for (83% vs. 80%). The smaller sample size did however reduce the power to detect a difference for secondary outcomes for which the LTFU was much higher than 25%. Also, the calculated sample size was not powered for subgroup analysis which was the case for outcomes relating to smoking, drinking, ART and cure rates. In addition, due to COVID-19 lockdown in March 2020, we had to switch to telephonic follow-up of participants using a shortened questionnaire (22 participants) and could not access clinics to retrieve outstanding TB treatment outcomes. The low intervention uptake meant that half of the participants received only one or two MI sessions combined with SMS messages. SMS messages were only used for the first half of the study period, and one quarter of participants did not receive their messages, a commonly occurring problem in developing countries.(20,55) It could be argued that in the absence of ongoing text messages, the MI and associated text messages were not enough to keep participants focussed for the second three months of the trial. The 2-arm study design did not permit the untangling of the individual effects of SMS and MI. Understanding their separate effects could have important cost implications as SMS-communication would be cheaper and easier to organise than individual counselling.

The lack of effectiveness of our intervention on the primary outcome (TB treatment success) can have a number of possible explanations. Although intervention uptake was high (80.2%) for the first counselling session, many participants did not return for the second (29.7%) and third (47%) sessions. As a result of this only about half of the intervention arm participants received all three MI sessions. Furthermore, about one quarter of all participants did not receive any SMS-messages. Low intervention uptake leads to a dilution of any potential effects. The lack of effectiveness on TB treatment success could perhaps also be explained by the complexity of the ProLife intervention itself: counsellors had to address multiple behaviours, namely medication adherence, tobacco smoking and hazardous/harmful drinking. Despite having established the feasibility and acceptability of this approach (31) and ongoing on site performance monitoring and feedback of counsellors, it is possible that MI for multiple behaviour change in the ProLife study was counterproductive as counsellors may have ended up not focusing on any of the behaviours at optimal levels. Similarly, patients might have found it difficult to change multiple behaviours simultaneously, especially because smoking and drinking are mutually reinforcing. This integrated approach was nevertheless adopted to avoid the need for multiple vertical counselling services (in addition to TB treatment and HIV treatment), to allow the different elements of the programme to reinforce one another, and to improve the affordability, feasibility and acceptability for a future roll-out of the programme. It is

For peer review only - http://bmjopen.bmj.com/site/about/guidelines.xhtml

also possible that sequential interventions may be better, at least for smoking cessation.(56) More intensive counselling (more sessions) or a modified counselling method may have been more appropriate, even more so since a recent review of reviews of MI casts doubt on its efficacy.(48,57,58) For example, more emphasis on increasing patient knowledge in addition to increasing self-efficacy may have been more effective.(59) The cause of the mobile health message delivery problems (such as poor network coverage, no electricity to charge phones) (60) would need to be investigated in order to increase the effectiveness of future mHealth interventions. Messages may also have to be intensified or modified to be more interactive and/or tailored to specific circumstances of each individual. This would improve the personal value of the intervention to the individual, which is likely to increase the chances of their participation in the intervention.(61) Consistent with the normalisation process theory,(62) cognitive participation in the intervention might have been higher had we been deliberate in the implementation to ensure the TB nurse who would have routinely seen the participants, provided additional support and motivated participants to attend MI sessions with the counsellor. In this way, the intervention would have gained 'legitimacy' but this would have led to unblinding of the nurses to the intervention arm.

In conclusion, we could not demonstrate that the ProLife intervention was effective in improving TB treatment outcomes. This may be due to the lack of effect of the intervention, but the study may also have been underpowered for the intermediary secondary outcomes. Valuable lessons were learnt on challenges relating to training LHWs in MI counselling and delivery, SMS-delivery in a challenging socio-economic context and the reasons for loss to follow-up of TB participants with multiple health problems. Further research is needed to provide answers on how to increase intervention uptake in poor resource settings and whether our complex intervention should have been more intensive. Other important questions are whether another counselling method would have been more effective. Lastly, in the light of the already existing evidence of SMS and the costs and implementation challenges relating to MI, intervention studies limited to an mHealth intervention but using different intensities, duration and type of interventions (one-way, two-way, interactive) are needed.

For peer review only - http://bmjopen.bmj.com/site/about/guidelines.xhtml

For peer review only

Acknowledgements

We are grateful to the following people: Ms M Malefo for her work as project manager, Ms D Bell for conducting the MI training, Ms N Kitleli for supervising the lay counsellors, Mesadie Machaea-Malinga for conducting the fidelity assessments, the district and clinic TB managers and TB nurses, the fieldworkers and the lay counsellors. The authors are also grateful to the National Department of Health and the three Provincial Departments of Health for granting permission for this study.

Contributors

MK contributed to study design and the manuscript, led on the sample size determination and did the statistical analysis. NKM contributed to study design, particularly with respect to development of the MI intervention, did the analysis of the MI data and contributed to the manuscript writing. AVZ led the data management and SMS-system, monitored data quality and contributed to data analysis and manuscript writing. ASM contributed to study design and led on the preparation of the manuscript. NDM contributed to study design, intervention development and manuscript preparation. JL and SP led on the economic evaluation. AT contributed to the economic evaluation. JT was site lead for the Bojanala sub-district. OBO was site lead for the Sedibeng sub-district. MOB contributed to study design, data interpretation and the manuscript. KS and OAA are co-principal investigators. Both contributed to study design, data interpretation and manuscript writing. All authors read and approved the final draft of the manuscript.

Funding

This project was funded by the SA-Medical Research/Newton Foundation Grant on TB control implementation science. (UK/South Africa Newton Fund RFA: TB control implementation science (MRC-RFA-02: TB -05-2015)). This UK funded award is part of the EDCTP2 programme supported by the European Union.

The funder had no role in study design; collection, management, analysis, and interpretation of data; writing of the report or the decision to submit the report for publication.

Trial sponsor: Sefako Makgatho Health Sciences University, RSA. Contact: lekan.ayo-yusuf@smu.ac.za

For peer review only

For peer review only - http://bmjopen.bmj.com/site/about/guidelines.xhtml

Competing interests

OA Ayo-Yusuf, the principal investigator, received a research grant (MRC-RFA-02: TB - 05-2015) which was allocated to Sefako Makgatho Health Sciences University to pay for the expenses related to the research project. As co-prinicipal investigator K Siddiqi received part of the MRC-Newton research grant (MRC-RFA-02: TB -05-2015) which was allocated to the University of York to support the research by the authors affiliated to the University of York (K Siddiqi, ND Mdege, M Kanaan, S Parrot, J Lee). Goedele Louwagie received a small monthly remuneration per month from 1 October 2018 until 30 June 2020 as academic research coordinator. André van Zyl was employed as a research data manager on a part-time basis from 1 September 2017 and 30 June 2020. OA Ayo-Yusuf received support for attending meetings and for travel by Tax Justice Network Africa. He is the Vice Chair of the Standing Committee on Health, Academy of Science, SA (not remunerated). The remaining authors declare no competing interests.

Ethical considerations

This study received ethics approval from the Research Ethics Committees of: Sefako Makgatho Health Sciences University (SMU; Ref: SMUREC/D/234/2017: IR); the South African Medical Research Council (with the SMU Ethics Committee serving as the Research Ethics Committee Record); the University of Pretoria (Ref: 434/2017); the University of Witwatersrand (M160455); and the University of York (no reference number, approval date 15 January 2017 (29).

Patient Consent for publication

Not applicable

Data availability statement

The study protocol was previously published (ImPROving TB outcomes by modifying LIFE-style behaviours through a brief motivational intervention followed by short text messages (ProLife): study protocol for a randomised controlled trial). The de-identified participant and SMS data sets are stored in labelled Stata files and are accompanied by a statistical analysis plan and metadata explaining each variable.

For peer review only

Although data can be viewed immediately, data will be embargoed for data analysis until 30 June 2023. Thereafter, permission must be obtained from the principal investigators (OA and KS) for any data analysis not yet performed by the primary research group. Data are stored in the institutional data repository at Sefako Makgatho Health Sciences University called Discover research (https://smu-za.figshare.com/) with a CC-BY 4.0 (Attribution) license (Creative Commons — Attribution 4.0 International — CC BY 4.0). The study protocol and statistical analysis plan are available as supplementary material.

Open access

This is an open access article distributed in accordance with a Creative Commons Attribution (CC BY 4.0) license, which permits others to copy, redistribute, remix, transform and build upon this work for any purpose, provided the original work is properly cited, a link to the licence is given and indication of whether changes were made. See: https://creativecommons.org/licenses/by/4.0/.

References

1. World Health Organisation. Global tuberculosis report 2020. Available: https://apps.who.int/iris/bitstream/handle/10665/336069/9789240013131-eng.pdf [Accessed 4 Aug 2021].
2. Fitchett JR, MacPherson P. Implementing the End TB Strategy and the intersection with the Sustainable Development Goals. *Trans R Soc Trop Med Hyg* 2016;110:145–7.
3. Thomas BE, Thiruvengadam K, Rani S, et al. Smoking, alcohol use disorder and tuberculosis treatment outcomes: A dual co-morbidity burdenthat cannot be ignored. *PLoS ONE* 14(7):e0220507.
4. Imtiaz S, Shield KD, Roerecke M, et al. Alcohol consumption as a risk factor for tuberculosis: meta-analyses and burden of disease. *Eur Respir J* 2017;50: 1700216.
5. Ogbo FA, Ogeleka P, Okoro A, et al. Tuberculosis disease burden and attributable risk factors in Nigeria, 1990–2016. *Tropical Medicine and Health* 2018;46:34.
6. Azeez A, Mutambayi R, Odeyemi A, et al. Survival model analysis of tuberculosis treatment among patients with human immunodeficiency virus coinfection. *Int J Mycobacteriol* 2019;8:244-51.
7. Mollel EW, Chilongola JO. Predictors for Mortality among Multidrug-Resistant Tuberculosis

For peer review only - http://bmjopen.bmj.com/site/about/guidelines.xhtml

Patients in Tanzania. J Trop Med 2017;2017:9241238. doi: 10.1155/2017/9241238.

8. Peltzer K. Conjoint alcohol and tobacco use among tuberculosis patients in public primary healthcare in South Africa. *S Afr J Psychiatr* 2014;20(1):21–6.

9. Ragan EJ, Kleinman MB, Sweigart B, et al. The impact of alcohol use on tuberculosis treatment outcomes: a systematic review and meta-analysis. *Int J Tuberc Lung Dis* 2020;24(1):73–82.

10. Department of Health of South Africa. National Tuberculosis Management Guidelines. Pretoria: 2014. Available from: http://www.tbonline.info/media/uploads/documents/ntcp_adult_tb-guidelines-27.5.2014.pdf [Accessed 4 Aug 2021].

11. Harder VS, Musau AM, Musyimi CW, et al. A randomized clinical trial of mobile phone motivational interviewing for alcohol use problems in Kenya. *Addiction* 2020 ;115:1050-1060.

12. Palacio A, Garay D, Langer B, et al. Motivational Interviewing Improves Medication Adherence: a Systematic Review and Meta-analysis. *Gen Intern Med.* 31:929–40. DOI: 10.1007/s11606-016-3685-3.

13. Madhombiro M, Kidd M, Dube B, et al. Effectiveness of a psychological intervention delivered by general nurses for alcohol use disorders in people living with HIV in Zimbabwe: a cluster randomized controlled trial. *J Int AIDS Soc* 2020 Dec;23:e25641.

14. Louwagie GMC, Okuyemi KS, Ayo-Yusuf OA. Efficacy of brief motivational interviewing on smoking cessation at tuberculosis clinics in Tshwane, South Africa: a randomized controlled trial. *Addiction* 2014;109:1942–52

15. World Health Organization. Handbook for the use of digital technologies to support tuberculosis medication adherence, 2017. Available: https://apps.who.int/iris/handle/10665/259832 [Accessed 4 Aug 2021].

16. Hirsch-Moverman Y, Daftary A, Yuengling KA, et al. Using mhealth for HIV/TB treatment support in lesotho: Enhancing patient-provider communication in the start study. *J Acquir Immune Defic Syndr* 2017;74:S37–43.

17. Shah R, Watson J, Free C. A systematic review and meta-analysis in the effectiveness of mobile phone interventions used to improve adherence to antiretroviral therapy in HIV infection. *BMC Public Health* 2019;19:915.

18. Gashu KD, Gelaye KA, Mekonnen ZA, et al. Does phone messaging improves tuberculosis treatment success? A systematic review and meta-analysis. *BMC Infect Dis* 2020;20:42.

19. Ngwatu BK, Nsengiyumva NP, Oxlade O, et al. The impact of digital health technologies on tuberculosis treatment: a systematic review. Eur Respir J. 2018 11;51(1):1701596. doi:

For peer review only

For peer review only - http://bmjopen.bmj.com/site/about/guidelines.xhtml

10.1183/13993003.01596-2017

20. Ggita JM, Ojok C, Meyer AJ, et al. Patterns of usage and preferences of users for tuberculosis-related text messages and voice calls in Uganda. *Int J Tuberc Lung Dis* 2018;22:530-536.

21. Oosterveen E, Tzelepis F, Ashton L, et al. A systematic review of eHealth behavioral interventions targeting smoking, nutrition, alcohol, physical activity and/or obesity for young adults. *Prev Med* 2017;99:197–206.

22. Haug S, Paz Castro R, Kowatsch T, et al. Efficacy of a technology-based, integrated smoking cessation and alcohol intervention for smoking cessation in adolescents: Results of a cluster-randomised controlled trial. *J Subst Abuse Treat* 2017;82:55–66.

23. Siddiqi K, Stubbs B, Lin Y, et al. TB multimorbidity: a global health challenge demanding urgent attention. *Int J Tuberc Lung Dis* 2021;25:87–90.

24. Moosa S, Derese A, Peersman W. Insights of health district managers on the implementation of primary health care outreach teams in Johannesburg, South Africa: A descriptive study with focus group discussions. *Hum Resour Health* 2017;15:1–9.

25. Marcus TS, Hugo J, Jinabhai CC. Which primary care model? A qualitative analysis of ward-based outreach teams in South Africa. *African J Prim Heal Care Fam Med* 2017;9:1–8.

26. Seidman G, Atun R. Does task shifting yield cost savings and improve efficiency for health systems? A systematic review of evidence from low-income and middle-income countries. *Hum Resour Health* 2017;15:1–13.

27. Heunis C, Wouters E, Kigozi G, et al. TB/HIV-related training, knowledge and attitudes of community health workers in the Free State province, South Africa. *African J AIDS Res* 2013;12:113–9.

28. World Health Organization & Centers for Disease Control (U.S.). Tobacco questions for surveys: a subset of key questions from the Global Adult Tobacco Survey (GATS): global tobacco surveillance system. *World Health Organization* 2011. Available from: https://apps.who.int/iris/handle/10665/87331 [Accessed 16 Nov 2021]

29. Bohn MJ, Babor TF, Kranzler HR. The Alcohol Use Disorders Identification Test (AUDIT): validation of a screening instrument for use in medical settings. *J Stud Alcohol* 1995;56:423–32.

30. Moriarty AS, Louwagie GM, Mdege ND, et al. ImPROving TB outcomes by modifying LIFE-style behaviours through a brief motivational intervention followed by short text messages (ProLife): Study protocol for a randomised controlled trial. *Trials* 2019;20:1–12.

31. Louwagie GM, Morojele N, Siddiqi K, et al. Addressing tobacco smoking and drinking to improve TB treatment outcomes, in South Africa: A feasibility study of the ProLife program. *Transl Behav Med* 2020; 10: 1491–1503.

For peer review only - http://bmjopen.bmj.com/site/about/guidelines.xhtml

32. CommCare. Cambridge, MA: Dimagi; 2017. Available from: https://play.google.com/store/apps/details?id=org.commcare.dalvik&hl=en_US&gl=US. [Accessed 15 June 2021].

33. Siddiqi K, Khan A, Ahmad M, et al. Action to stop smoking in suspected tuberculosis (ASSIST) in Pakistan: a cluster randomized, controlled trial. *Ann Intern Med* 2013;158:667–75.

34. West R, Hajek P, Stead L, et al. Outcome criteria in smoking cessation trials: proposal for a common standard. *Addiction* 2005;100:299–303.

35. van den Boogaard J, Lyimo RA, Boeree MJ, et al. Electronic monitoring of treatment adherence and validation of alternative adherence measures in tuberculosis patients: A pilot study. *Bull World Health Organ* 2011;89:632–9.

36. OECD [Internet]. Available from: https://data.oecd.org/conversion/exchange-rates.htm [Accessed 26 Oct 2021]

37. Augustovski FA, Irazola VE, Velazquez AP, et al. Argentine valuation of the EQ-5D health states. *Value Heal* 2009;12:587–96.

38. StataCorp. Stata Statistical Software: Release 15. College Station, TX: StataCorp LLC. 2017.

39. Team RC. R: A language and environment for statistical computing. Vienna, Austria: R Foundation for Statistical Computing; 2017. Available from: https://www.r-project.org/ [Accessed 04 Aug 2021]

40. Moyers TB, Rowell LN, Manuel JK, et al. The Motivational Interviewing Treatment Integrity Code (MITI 4): Rationale, Preliminary Reliability and Validity. *J Subst Abuse Treat* 2016;65:36-42. doi:10.1016/j.jsat.2016.01.001

41. International Monetary Fund. IMF DataMapper - GDP per capita, current prices (Purchasing power parity; international dollars per capita), 2018. Available from: https://www.imf.org/external/datamapper/datasets [Accessed 04 Aug 2021]

42. Richardson GMA. Calculation of quality adjusted life years in the published literature: a review of methodology and transparency. *Health Econ* 2004;13:1203–10.

43. Khachadourian V, Truzyan N, Harutyunyan A, et al. People-centred care versus clinic-based DOT for continuation phase TB treatment in Armenia: a cluster randomized trial. *BMC Pulm Med* 2020;20:105.

44. Thiam S, LeFevre AM, Hane F, et al. Effectiveness of a strategy to improve adherence to tuberculosis treatment in a resource-poor setting: a cluster randomized controlled trial. *JAMA* 2007;297:380–6.

45. Goel S, Kathiresan J, Singh P SR. Effect of a brief smoking cessation intervention on adult tobacco smokers with pulmonary tuberculosis: A cluster randomized controlled trial from

For peer review only - http://bmjopen.bmj.com/site/about/guidelines.xhtml

North India. *Indian J Public Heal* 2017;61(Suppl 1):S47–53

46. Manogaran C, Nagarajan K, Ovung S, et al. Alcohol intervention strategy among tuberculosis patients: a pilot study from South India. *Int J Tuberc Lung Dis* 2017;21:947–52

47. Siddiqi K, Keding A, Marshall AM, et al. Effect of quitting smoking on health outcomes during treatment for tuberculosis: secondary analysis of the TB & Tobacco Trial. *Thorax* 2021 Published Online First Jul 16:thoraxjnl-2020-215926. doi: 10.1136/thoraxjnl-2020-215926.

48. Lindson N, Thompson TP, Ferrey A, et al. Motivational interviewing for smoking cessation. *Cochrane Database Syst Rev*. 2019;2019(7).

49. McCambridge J, Kypri K. Can simply answering research questions change behaviour? Systematic review and meta analyses of brief alcohol intervention trials. *PLoS One* 2011;6(10):e23748.

50. Peltzer K, Naidoo P, Louw J, et al. Screening and brief interventions for hazardous and harmful alcohol use among patients with active tuberculosis attending primary public care clinics in South Africa: results from a cluster randomized controlled trial. *BMC Public Health* 2013;13:699.

51. Riper H, Andersson G, Hunter SB, et al. Treatment of comorbid alcohol use disorders and depression with cognitive-behavioural therapy and motivational interviewing: a meta-analysis. *Addiction* 2014;109:394–406.

52. Moosa A, Gengiah TN, Lewis L, et al. Long-term adherence to antiretroviral therapy in a South African adult patient cohort: a retrospective study. *BMC Infect Dis* 2019;19(1):775. doi: 10.1186/s12879-019-4410-8.

53. Webb Mazinyo E, Kim L, Masuku S, et al. Adherence to Concurrent Tuberculosis Treatment and Antiretroviral Treatment among Co-Infected Persons in South Africa, 2008–2010. *PLoS One* 2016;11(7): e0159317. https://doi.org/10.1371/journal.pone.0159317.

54. Stirratt MJ, Dunbar-Jacob J, Crane HM, et al. Self-report measures of medication adherence behavior: recommendations on optimal use. *Transl Behav Med* 2015;5:470–82.

55. Wanyama JN, Nabaggala SM, Kiragga A, et al. High mobile phone ownership but low internet access and use among young adults attending an urban HIV clinic in Uganda. *Vulnerable Child Youth Stud* 2018;13:3,207-20.

56. Meader N, King K, Wright K et al. Multiple risk behavior interventions: Meta-analyses of RCTs. *Am J Prev Med* 2017;53:e19–e30.

57. Abraham C, Michie S. A taxonomy of behavior change techniques used in interventions. *Health Psychol* 2008;27:379-87.

58. Frost H, Campbell P, Maxwell M, et al. Effectiveness of Motivational Interviewing on adult

For peer review only

For peer review only - http://bmjopen.bmj.com/site/about/guidelines.xhtml

behaviour change in health and social care settings: A systematic review of reviews. *PLoS One* 2018;18:13(10):e0204890

59. Kahwati L, Viswanathan M, Golin CE, et al. Identifying configurations of behavior change techniques in effective medication adherence interventions: a qualitative comparative analysis. *Syst Rev* 2016;5(83).

60. Telerivet. Message Statuses . User Guide, 2021. Available from: https://guide.telerivet.com/hc/en-us/articles/360038959611-Message-Statuses. [Accessed 21 June 2021]

61. Lustria MLA, Noar SM, Cortese J, et al. A meta-analysis of web-delivered tailored health behavior change interventions. *Journal of Health Communication* 2013;18:1039–69.

62. Gallacher K, May CR, Montori V, et al. Understanding Patients' Experiences of Treatment Burden in Chronic Heart Failure Using Normalization Process Theory. *Ann Fam Med* 2011;9:235-243.

For peer review only

For peer review only

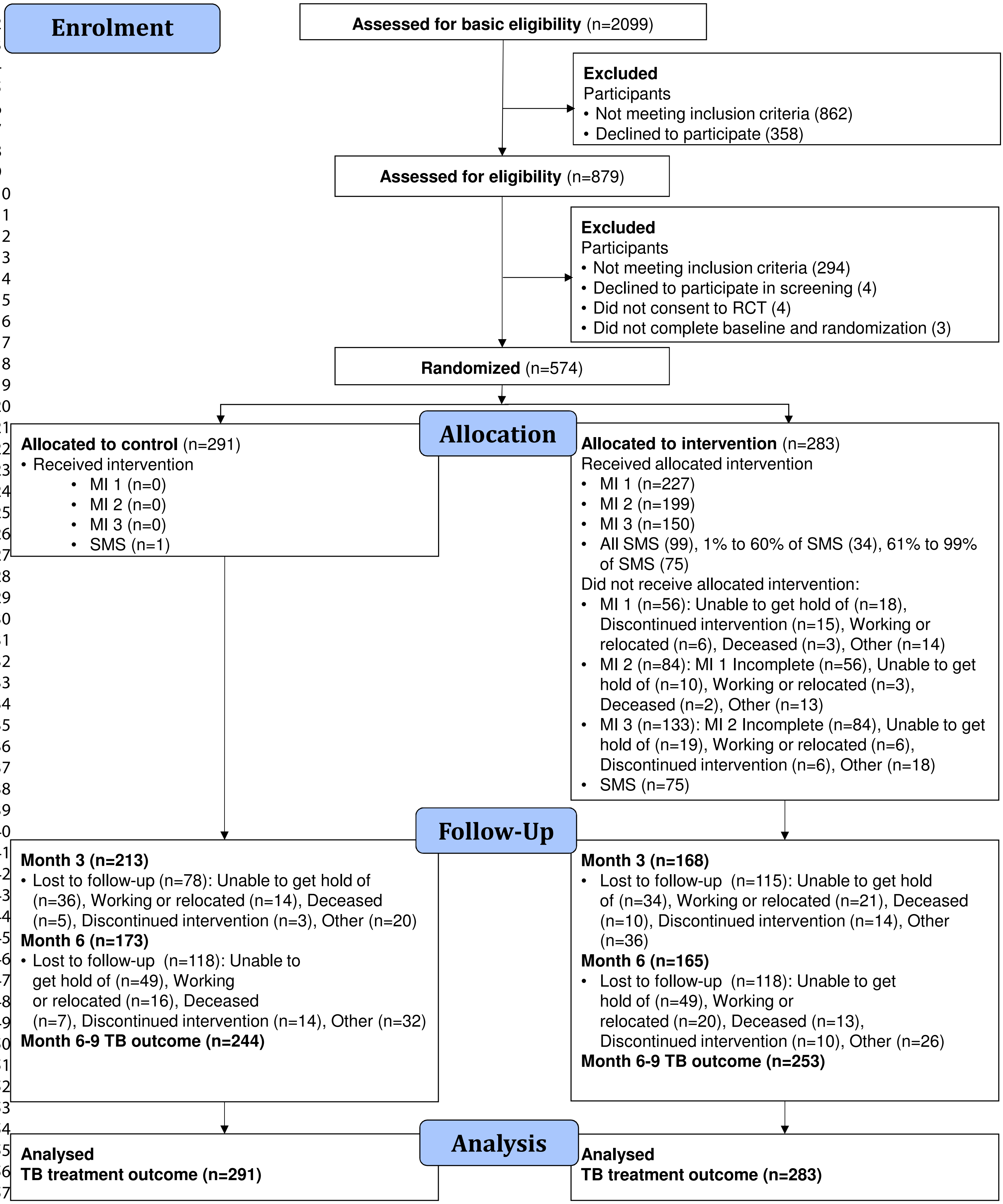
Enrolment
Assessed for basic eligibility (n=2099)
Excluded
Participants
• Not meeting inclusion criteria (862)
• Declined to participate (358)
Assessed for eligibility (n=879)
Excluded
Participants
• Not meeting inclusion criteria (294)
• Declined to participate in screening (4)
• Did not consent to RCT (4)
• Did not complete baseline and randomization (3)
Randomized (n=574)
Allocation
Allocated to control (n=291)
• Received intervention
• MI 1 (n=0)
• MI 2 (n=0)
• MI 3 (n=0)
• SMS (n=1)
Allocated to intervention (n=283)
Received allocated intervention
• MI 1 (n=227)
• MI 2 (n=199)
• MI 3 (n=150)
• All SMS (99), 1% to 60% of SMS (34), 61% to 99% of SMS (75)
Did not receive allocated intervention:
• MI 1 (n=56): Unable to get hold of (n=18), Discontinued intervention (n=15), Working or relocated (n=6), Deceased (n=3), Other (n=14)
• MI 2 (n=84): MI 1 Incomplete (n=56), Unable to get hold of (n=10), Working or relocated (n=3), Deceased (n=2), Other (n=13)
• MI 3 (n=133): MI 2 Incomplete (n=84), Unable to get hold of (n=19), Working or relocated (n=6), Discontinued intervention (n=6), Other (n=18)
• SMS (n=75)
Follow-Up
Month 3 (n=213)
• Lost to follow-up (n=78): Unable to get hold of (n=36), Working or relocated (n=14), Deceased (n=5), Discontinued intervention (n=3), Other (n=20)
Month 6 (n=173)
• Lost to follow-up (n=118): Unable to get hold of (n=49), Working or relocated (n=16), Deceased (n=7), Discontinued intervention (n=14), Other (n=32)
Month 6-9 TB outcome (n=244)
Month 3 (n=168)
• Lost to follow-up (n=115): Unable to get hold of (n=34), Working or relocated (n=21), Deceased (n=10), Discontinued intervention (n=14), Other (n=36)
Month 6 (n=165)
• Lost to follow-up (n=118): Unable to get hold of (n=49), Working or relocated (n=20), Deceased (n=13), Discontinued intervention (n=10), Other (n=26)
Month 6-9 TB outcome (n=253)
Analysis
Analysed
TB treatment outcome (n=291)
Analysed
TB treatment outcome (n=283)

SUPPLEMENTARY TABLES

Supplementary table 1 Detailed baseline descriptive statistics for socio-demographic, socio-economic, and clinical characteristics by study arm

	Control (N = 291) n (%)*	Intervention (N = 283) n (%)*	Total (N=574) n (%)*
**Marital status**			
Married or living together	102 (35.1)	95 (33.6)	197 (34.3)
Divorced/separated	20 (6.9)	18 (6.4)	38 (6.6)
Widowed	10 (3.4)	7 (2.5)	17 (3.0)
Never married and never lived together	144 (49.5)	150 (53.0)	294 (51.2)
Declined to answer	15 (5.2)	13 (4.6)	28 (4.9)
**Employment**			
Self-employed (full-time)	30 (10.3)	36 (12.7)	66 (11.5)
Employed full-time (30 hrs a week or more)	62 (21.3)	54 (19.1)	116 (20.2)
Employed part-time (less than 30 hrs a week)	19 (6.5)	29 (10.2)	48 (8.4)
Retired	17 (5.8)	16 (5.7)	33 (5.7)
Unemployed (but able to work)	125 (43.0)	120 (42.4)	245 (42.7)
Unable to work because of long-term disability or ill health	9 (3.1)	8 (2.8)	17 (3.0)
Full-time student	12 (4.1)	4 (1.4)	16 (2.8)
Caring from my home and family/doing household work/housewife	0 (0.0)	2 (0.7)	2 (0.3)
Occasional work (“piece job”)	17 (5.8)	12 (4.2)	29 (5.1)
Declined to answer	0 (0.0)	2 (0.7)	2 (0.3)
**Ever worked or spent time in mines**			
No	244 (83.8)	237 (83.7)	481 (83.8)
Yes	46 (15.8)	45 (15.9)	91 (15.9)
**Household items (Yes is displayed)**			
A radio	249 (85.6)	234 (82.7)	483 (84.1)
A television	255 (87.6)	255 (90.1)	510 (88.9)
A landline telephone	21 (7.2)	22 (7.8)	43 (7.5)
A desktop or laptop computer	67 (23.0)	48 (17.0)	115 (20.0)
A refrigerator	248 (85.2)	240 (84.8)	488 (85.0)
A vacuum cleaner or floor pol	49 (16.8)	35 (12.4)	84 (14.6)
A microwave oven	198 (68.0)	189 (66.8)	387 (67.4)
An electric or gas stove	254 (87.3)	238 (84.1)	492 (85.7)
A washing machine	153 (52.6)	136 (48.1)	289 (50.3)
Total number of assets: mean (SD)	5.14 (1.96)	4.94 (1.77)	5.04 (1.87)
**In the past month, number of days you or people in the household went to bed hungry because there was no food to eat**			
0 days	244 (83.8)	238 (84.1)	482 (84.0)
1-7 days	45 (15.5)	34 (12.0)	79 (13.8)
More than 7 days	2 (0.7)	9 (3.2)	11 (1.9)
Declined to answer	0 (0.0)	2 (0.7)	2 (0.3)
**Number of pre-treatment smear tests recorded?**			

For peer review only

One	197 (92.1)	169 (87.6)	366 (89.9)
Two	17 (7.9)	24 (12.4)	41 (10.1)
**Number of Gene XPert results recorded**			
One	225 (97.8)	210 (97.2)	435 (97.5)
Two	5 (2.2)	6 (2.8)	11 (2.5)
**Number of culture results recorded on the TB Treatment record**			
One	54 (94.7)	42 (97.7)	96 (96.0)
Two	3 (5.3)	1 (2.3)	4 (4.0)
**Co-morbidities**			
Hypertension	19 (6.93)	11 (4.1)	30 (5.54)
Diabetes	5 (1.84)	4 (1.49)	9 (1.66)
Epilepsy	3 (1.09)	4 (1.49)	7 (1.29)
Mental illness	3 (1.09)	0 (0)	3 (0.55)
Liver disease	1 (0.36)	1 (0.38)	2 (0.37)
Renal insufficiency	1 (0.36)	1 (0.38)	2 (0.37)
Allergies	2 (0.76)	0 (0)	2 (0.38)
Other	1 (0.36)	1 (0.38)	2 (0.37)
**Depression score (CESD 10): mean (SD)**	8.44 (4.38)	8.74 (4.8)	8.59 (4.59)

*Counts and percentages unless otherwise indicated

For peer review only

**Supplementary table 2** Detailed descriptive statistics for smoking history, alcohol history at baseline by study arm			
	Control<br>n (%)*	Intervention<br>n (%)*	Total<br>n (%)*
Smoking History (current smokers only) N=	181	191	372
**On the days that you smoke, how soon after you wake up do you have your first cigarette?**			
After 60 minutes	30 (16.6)	28 (14.7)	58 (15.6)
31-60 minutes	24 (13.3)	16 (8.4)	40 (10.8)
6- 30 minutes	60 (33.1)	65 (34.0)	125 (33.6)
Within 5 minutes	67 (37.0)	82 (42.9)	149 (40.1)
**Duration of smoking in months: mean (SD)**	212.09 (134.03)	224.93 (127.82)	218.68 (130.86)
**Duration of smoking in months: median (IQR)**	186 (110, 282)	206 (135, 294)	200.5 (123, 287)
**Age started smoking in years: mean (SD)**	19.2 (6.3)	19.3 (6.3)	19.3 (6.3)
**Age started smoking in years: median (IQR)**	18 (15-20)	18 (16-21)	18 (15.5-20.5)
**Form of tobacco used**			
**Manufactured cigarettes (Yes)**	166 (91.7)	179 (93.7)	345 (92.7)
Number of days you smoked in the past 7days: mean (SD)	5.3 (2.65)	5.53 (2.33)	5.42 (2.49)
Average number of cigarettes smoked daily: mean (SD)	6.18 (6.43)	6.48 (8.21)	6.34 (7.39)
**Hand-rolled cigarettes (Yes)**	14 (7.7)	21 (11.0)	35 (9.4)
Number of days you smoked in the past 7days: mean (SD)	4.71 (2.84)	3.71 (2.95)	4.11 (2.91)
Average number of handrolled cigarettes smoked daily: mean (SD)	3.71 (3.97)	3.81 (2.82)	3.77 (3.27)
**Pipe (Yes)**	4 (2.2)	2 (1.0)	6 (1.6)
Number of days in the past 7days you smoked: median (IQR)	1 (0, 2.5)	4.5 (2, 7)	2 (0, 3)
Average number of daily sessions: median (IQR)	1 (0, 3.5)	3 (1, 5)	1.5 (0, 5)
Length of one session (on average) in **minutes**: median (IQR)	90 (60, 107.5)	60 (30, 90)	90 (30, 90)
**Cigars, cheroots or cigarillos (Yes)**	1 (0.55)	0 (0)	1(0.27)
**Water pipe (Yes)**	3 (1.7)	2 (1.0)	5 (1.3)
**Other**	10 (5.5)	4 (2.1)	14 (3.8)
**Heaviness of smoking index >= 4**	134(74.03)	158(82.72)	292(78.49)
Smoking inside your home restrictions			
Total: Not allowed	96 (53.0)	108 (56.5)	204 (54.8)
Some rules: where/when it is allowed	61 (33.7)	58 (30.4)	119 (32.0)
No rules	24 (13.3)	25 (13.1)	49 (13.2)

For peer review only

Attempts to quit smoking (current smokers only)			
**Ever attempted to quit in the past (Yes)**	52 (28.7)	64 (33.5)	116 (31.2)
Number of attempts to quit: mean (SD)	2.46 (2.98)	2.64 (1.62)	2.56 (2.32)
Time elapsed since attempt to quit last time, in months: mean (SD)	36.65 (94.62)	25.13 (37.91)	30.29 (69.22)
Longest duration abstinent in previous quit attempts: mean (SD)	6.15 (13.48)	4.22 (8.9)	5.09 (11.18)
**Likelihood to TRY TO QUIT smoking completely and permanently in the next three months**			
Definitely will not	6 (3.3)	5 (2.6)	11 (3.0)
Probably will not	10 (5.5)	12 (6.3)	22 (5.9)
Probably will	104 (57.5)	109 (57.1)	213 (57.3)
Definitely will	61 (33.7)	65 (34.0)	126 (33.9)
**Likelihood that I WILL QUIT smoking completely and permanently in the next three months**			
Definitely will not	6 (3.3)	5 (2.6)	11 (3.0)
Probably will not	11 (6.1)	13 (6.8)	24 (6.5)
Probably will	103 (56.9)	104 (54.5)	207 (55.6)
Definitely will	61 (33.7)	69 (36.1)	130 (34.9)
**Ever used any methods to help you stop smoking tobacco in the past 3-months?** (Yes)	23 (12.7)	16 (8.4)	39 (10.5)
Smokeless tobacco use (all participants)			
**In the past month, have you used smokeless tobacco (Snuff) on a daily basis**			
Not at all	275 (94.5)	275 (97.2)	550 (95.8)
Daily	12 (4.1)	6 (2.1)	18 (3.1)
Less than Daily	4 (1.4)	2 (0.7)	6 (1.0)
**Duration of using ST in months: mean(SD)**	113.69 (112.7)	152 (91.16)	126.46 (105.61)
**Age started using ST in years: mean (SD)**	27.56 (10.57)	25.88 (13.43)	27 (11.33)
**Form of ST used (for SLT users)**			
Snuff (by mouth)	2 (12.5)	0 (0.0)	2 (8.3)
Snuff (by nose)	11 (68.8)	5 (62.5)	16 (66.7)
Chewing tobacco leaves	0(0)	0(0)	0(0)
Other	1 (6.3)	0 (0.0)	1 (4.2)
**Help to stop drinking (N= , had a drink in past 12 months)**	208	223	431
**Ever used any methods to stop drinking alcohol in the past 3-months**	21 (11.2)	22 (10.7)	43 (10.9)

* Frequencies and (percentages) are presented unless otherwise stated

For peer review only - http://bmjopen.bmj.com/site/about/guidelines.xhtml

Supplementary table 3 Detailed descriptive statistics for primary outcome by study arm at 6-months

TB treatment status detailed	Control n (%)	Intervention n (%)	Total n (%)
Cured	108 (37.1)	105 (37.1)	213 (37.1)
Treatment completed	96 (33.0)	87 (30.7)	183 (31.9)
Treatment interrupted > 2 months	15 (5.2)	29 (10.2)	44 (7.7)
Treatment failure	5 (1.7)	2 (0.7)	7 (1.2)
Acquired drug resistance	1 (0.3)	4 (1.4)	5 (0.87)
Died	11 (3.8)	15 (5.3)	26 (4.5)
Transfer out	8 (2.75)	11 (3.9)	19 (3.31)
Unknown	42 (14.4)	25 (8.8)	67 (11.7)

For peer review only

**Supplementary table 4** MI treatment fidelity scores of counselling sessions delivered by 17 lay health workers*		
**Ratings**	Mean (SD)	Range
**Global ratings****		
**Cultivating change talk**	3.2 (1.17)	1-5
**Softening sustain talk**	3.4 (0.96)	1-4
**Partnership**	3.4 (1.12)	2-5
**Empathy**	2.9 (1.34)	1-5
**Behaviour counts**[&]		
**Giving information**	4.8 (8.13)	0-5
**Persuade**	0.8 (1.48)	0-5
**Persuade with permission**	0.1 (0.24)	0-1
**Question**	24.2 (10.42)	12-51
**Simple reflection**	4.1 (3.21)	0-10
**Complex reflection**	1.6 (2.69)	0-9
**Affirm**	5.5 (3.47)	1-12
**Seeking collaboration**	2.4 (1.46)	0-6
**Emphasising autonomy**	1.4 (1.37)	0-4
**Confront**	0.4 (0.74)	0-3
**Summary measures**		
**Total MI non-adherent †**	1.2 (2.28)	0-7
**Total MI adherent†**	9.3 (4.74)	1-16
**Technical global ratings****	3.3 (0.97)	1.5-4.5
**Relational global ratings****	3.1 (1.19)	1.5-5
**Reflection to question ratio††**	0.23 (0.24)	0-0.83
**Percentage of complex reflections*****	20.4 (21.9)	0-67

*The recordings of 17 counsellors (one each) were transcribed verbatim and then assessed. In order to assess the fidelity of the counsellors' delivery of motivational interviewing during the trial, we are used the validated Motivational Interviewing Treatment Integrity Coding Manual 4.2.1 (MITI) tool. The table shows the results of the fidelity assessment that was conducted by one rater who is proficient in English, seSotho and seTswana and in motivational interviewing. The rater listened to the recordings and coded a randomly selected 20-min portion of the written transcript. In the case of shorter counselling sessions, the entire recording was assessed.

**The "global ratings" involve assessing, on a 5-point Likert scale (1 for low and 5 for high), how well or poorly the counsellor adheres to the MI practice. Ratings are conducted on four items, two each making up Technical Components (Cultivating change talk and Softening sustain talk) and Relational Components (Partnership and Empathy).

**Scores on Cultivating Change Talk and Softening Sustain Talk are averaged to obtain the Technical global scores.

***Scores on Partnership and Empathy are averaged to obtain Relational global scores. The basic competency threshold scores for fair and good proficiency are 3 and 4, respectively for Technical scores and 3.5 and 4, respectively for Relational scores.

[&]The "behaviour counts" involve counting 10 verbal behaviours of the counsellor during the intervention.

† "MI adherence" is determined by adding up the following verbal behaviours: Seeking Collaboration, Affirm and Emphasising autonomy. "MI non-adherence" is determined by summing instances of Confront and Persuade. No thresholds for MI adherence or non-adherence are specified in the MITI 4.2.1.

††The Reflection-to-Question (R:Q) ratio is the total reflections divided by the total questions asked. One reflection to each question is considered a "fair" practice level while two reflections to each question is considered a "good" practice level.

*** The Percentage of Complex Reflections (% CRs) is calculated by dividing the number of complex reflections by the sum of complex reflections and simple reflections. A fair and a good % CRs are 40% and 50%, respectively.

For peer review only

Supplementary table 5 SMS delivery

	Intervention (N=283)		Control (N=291)	
No. of participants who received ALL due IMB messages INDEPENDENT OF WHETHER they completed MI 1	99/283 (35%)		1/291 (0.3%)	
**Completion of first MI and initiation of SMS-sequence**				
Completed first MI	227/283 (80.2%)		0/291 (0%)	
No. of participants who received ALL due messages after receipt of MI1	95/227 (41.9%)		0/0 (0%)	
**SMS delivery for participants for whom the SMS-sequence was initiated (after receipt of first MI)**				
	Mean (SD)	Median (IQR) Range	Mean (SD)	Median (IQR) Range
Average no. adherence messages received per participant (n=227)	7.9 (3.5)	10 (8-10) 0-11	0	0
Average no. tobacco-related messages received (n=153)	5.4 (2.5)	7 (5-7) 0-7	0	0
Average no. alcohol related messages received (n=171)	5.5 (2.5)	7 (5-7) 0-7	0	0
Average no. IMB messages received (n= 227)	15.7 (7.3)	17 (13-22) 0-25		

For peer review only

For peer review only - http://bmjopen.bmj.com/site/about/guidelines.xhtml

Supplementary table 6 Unit costs used in the analysis (Presented in Rand as sources, $1=R14.448 in 2019)

Item	Unit costs
**Personnel**	LHWs: R3000/month, 160 hours/month, R18.75/hour; District coordinators: R7500/month, 160 hours/month, R46.88/hour; MRC appointed supervisor: R205.79/hour; Trainers: R373.17/hour; Administrative staff: R333.24/hour
**Materials**	Training manual: R3250; Printing: R3000; Additional printing & stationary: R500
**Accommodation / Travel / Refreshments**	LHWs accommodation: R540 000; Trainers accommodation/travel: R36 000; Supervision accommodation/travel: R30 000; Refreshment: R12 500
**SMS system**	Monthly subscription at R433.44 ($30) for 3-months: R1300
**MI sessions**	Session 1 (17 minutes): R5.31; Session 2 (16 minutes): R5.00; Session 3 (17 minutes): R5.31
**Biochemical investigations**	Cost of TB smear microscopy: R28.37; culture: R79.22 and GeneXpert: R201.56 (Source: 2019/20 National Health Laboratory Service pricing schedule)*
**ART**	Atroiza: R3.78/dose; Dumiva: R5.55/dose; Tenemine: R2.34/dose; Zovilam: R1.72/dose; Kavimun: R1.89/dose; Ricovir: R1.36/dose; Zidomat: R1.38/dose; Lazena: R0.55/dose; Efrin:R0.63/dose; Efamat: R0.63/dose; Acriptaz: R0.61/dose (Source: Western Cape Department of Health. Antiretroviral and TB Stockmaster Worksheet. 2019)
**TB medication**	Month's supply of RHZE (intensive) @ R65.80; RH (continuation) @ R55.56 (Source: Western Cape Department of Health. Antiretroviral and TB Stockmaster Worksheet. 2019)

For peer review only

PROLIFE

Im*pro*ving TB outcomes by modifying *life*-style behaviours through a brief motivational intervention followed by short text messages (Phase II)

A multi-centre randomised controlled trial looking at the effect of a complex behavioural intervention on TB and lifestyle-related outcomes in South Africa

Version 1.2

Trial Registration number/registry: ISRCTN62728852/ISRCTN registry http://www.isrctn.com/ISRCTN62728852

Version date: ~~**14 Feb 2020**~~ 25 Oct 2020
Author(s): Mona Kanaan

Department of Health Sciences
University of York
York, YO10 5DD

Principal Investigator: Olalekan Ayo-Yusuf and Kamran Siddiqi
Trial Coordinator: Goedele M Louwagie

**Sponsor**	**Olalekan Ayo-Yusuf, Sefako Makgatho University/ University of Pretoria**
**Funder**	**Newton Fund/MRC**
**Funder Reference Number**	**MRC-RFA-02: TB -05-2015**

For peer review only

STATISTICAL ANALYSIS PLAN
V1.2

Contents

1. Definition of terms 4
2. Trial Objectives 5
3. Design 6
4. Sample Size 7
5. Randomisation 7
6. Outcomes 8
   6.1 Primary outcome 8
   6.2 Secondary outcomes 9
      6.2.1 Monitoring adverse events 9
   6.3 Trial assessment schedule 11
   6.4 Other important information 12
   6.5 Fidelity of the intervention 12
7. Data 13
   7.1 Data collection methods 13
   7.2 Data management 16
8. Analysis 17
   8.1 Participants Flow 18
   8.2 Baseline data 19
   8.3 Primary analysis 24
      8.3.1 Primary outcome definition: 24
      8.3.2 Primary outcome Analysis 24
      8.3.3 Primary outcome Analysis Results 25
   8.4 Secondary analyses 26
      8.4.1 Secondary outcome definitions that involve defining a positive baseline test: 27
      8.4.2 Continuous Abstinence 29
      8.4.4 Change in harmful or hazardous drinking at month 3 and 6 follow-ups 32
      8.4.5 Proportion of ART uptake of HIV-positive participants at month 3 and 6 follow-ups . 34
   8.5 Subgroup analyses (See above analyses) 35
   8.5 Sensitivity analyses 35
   8.6 Adverse events 36
   8.7 Planned interim review and analyses 36

For peer review only - http://bmjopen.bmj.com/site/about/guidelines.xhtml

8.8 List of Tables and Graphs 37
9. SAP amendment log 38
10. Signatures of approval 38
11. References 39
12. Appendix A (Results tables) 40
Table 1: 40
13. Appendix B (Scoring notes +) .... **Error! Bookmark not defined.**
13. Additional descriptive Statistics 65

This analysis plan deals only with the statistical analysis of the trial.

For peer review only

1. Definition of terms

**Abbreviation**	**Expansion**
ACTG	AIDS Clinical Trials Group
AE	Adverse Event
ART	Anti-retroviral therapy
AUDIT	Alcohol Use Disorders Identification Test
CI	Confidence Interval
CO	Carbon monoxide
CONSORT	Consolidated Standards of Reporting Trials
CRF	Case Report Form
EPTB	Extra-pulmonary TB
HIV	Human immunodeficiency virus
LHW	Lay health worker
MI	Motivational interviewing
PTB	Pulmonary TB
SMS	Short Message Service
SA	South Africa
SADHS	South Africa Demographic and Health Survey
SAE	Serious Adverse Event
SD	Standard deviation
SOPs	Standard Operating Procedures
TB	Tuberculosis

For peer review only - http://bmjopen.bmj.com/site/about/guidelines.xhtml

2. Trial Objectives

The PROLIFE model is a complex behavioural intervention comprised of a brief motivational interviewing (MI) counselling strategy augmented with subsequent SMS messaging. To be delivered in three brief sessions, the MI intervention will target three main areas, as appropriate:

- Tobacco smoking
- Alcohol drinking
- Tuberculosis (TB) and Anti-retroviral therapy (ART) adherence or ART initiation

Primary objective:

- To assess the effectiveness of the PROLIFE model delivered by lay health workers (LWH) compared to usual care in improving Pulmonary TB (PTB) treatment outcomes

Secondary objective: (**this element will not be addressed in this SAP**)

- To estimate the cost-effectiveness of the PROLIFE model

For peer review only - http://bmjopen.bmj.com/site/about/guidelines.xhtml

3. Design

This is a pragmatic, prospective, multicentre, two-arm, parallel, individual RCT taking place in 27 purposively selected primary care clinics with the highest TB case-load in three districts in South Africa: Welkom in the Free State; Bojanala in the North West province; and Sedibeng in Gauteng province. The intervention will be delivered by LHWs and three district coordinators who will each cover 1–2 clinics.

This is a pragmatic parallel superiority individually randomised controlled trial. There are two treatment arms:

The control arm (Arm1): Intervention arm – participants will receive the PROLIFE programme;

The intervention arm (Arm2): Control arm – participants will receive usual treatment and support provided to TB patients in TB treatment clinics in South Africa ('usual care').

Full details of the background and design of the trial are presented in the protocol (version 1.2 Prolife Protocol_15 Dec 17_with markup) and the published protocol in Moriarty et al (2019) https://doi.org/10.1186/s13063-019-3551-9.

Participants

The inclusion criteria for participants are:

- adult patients (aged ≥ 18 years)
    - with drug-sensitive (bacteriologically or clinically confirmed) PTB;
- initiating TB treatment or on TB treatment for < 1 month (these include both 'new' and 'retreatment' patients);
- current smokers and/or
- hazardous/harmful drinkers who are not alcohol dependent (Alcohol Use Disorders Identification Test [AUDIT] score ≥ 8 for men or ≥ 7 for women but < 20);
- access to a functional mobile phone; and
- understand one of the four languages used for the trial (Sesotho, Setswana, Isizulu or English).

Exclusion criteria:

- alcohol-dependent participants (AUDIT score ≥ 20);
- Extrapulmonary TB without PTB; or
- Resistance to one or more TB drugs at baseline

For peer review only - http://bmjopen.bmj.com/site/about/guidelines.xhtml

4. Sample Size

We will recruit 696 participants (348 per study arm). The sample size calculations were based on the following assumptions:

- Detection a 10% difference in TB treatment success rates (0.86 vs 0.76) in the ProLife group versus the control group
- 80% power,
- a significance level of 0.05, and
- 25% attrition.

The sample size per clinic was in the range of 14–74 participants per clinic with a median of 24. The assumed success rates in the control group are based on actual success rates in TB patients in the studied provinces that were available at the time of sample size calculations in 2015.

5. Randomisation

Patients will be randomised using a randomised sequence generator performed by the trial statistician (MK) who will remain blind to the arm allocation. We will use block randomisation with varying block sizes stratified by the clinic to achieve equal numbers in intervention and control groups within each clinic. Allocation concealment will be done with consecutively numbered, sealed, opaque envelopes.

Lay health workers delivering the intervention, field researchers, and participants cannot be blinded to the intervention. However, the determination of the primary outcome will be completed by TB nurses who are blinded to the intervention status of the participants based on routinely collected data.

The statistician will be blinded to the intervention or control arm allocation of participants during the analysis stage.

For peer review only

6. Outcomes

6.1 Primary outcome

The primary outcome is TB treatment success at six to nine months of follow-up. This is a binary variable defined as

- Success: cured or treatment completed
- Failure: failed treatment, death, acquired drug resistance, loss to follow-up or 'default', or not outcome evaluated.

The different mutually exclusive treatment outcomes are summarised here

Treatment outcome	Definition
Cure	Patient in whom baseline smear or culture was positive at beginning of treatment AND is smear/culture negative in the last month of treatment and on at least one previous occasion at least 30 days prior<br>According to local protocol, a patient who is diagnosed using Gene Xpert and is sputum negative for TB at 11 and 23 weeks is considered 'Cured'.
Treatment completed	Patient whose baseline smear or culture was positive at the beginning and has completed treatment but does not have a negative smear/culture in the last month of treatment and on at least one previous occasion > 30 days prior. Patients diagnosed with PTB whose baseline smear (or culture) result was negative and who started treatment based on clinical and radiological findings who have shown clinical improvement and completed the prescribed course of treatment.<br>*N.B. The smear examination may not have been done or the results may not be available at the end of treatment.*
Treatment failure	Patient whose baseline smear or culture was positive and remains or becomes positive again at 5 months or later during treatment.<br>Patients who were negative at baseline but were later found to be positive.<br>*N.B. This definition excludes those patients who are diagnosed with RR-TB or MDR-TB during treatment.*
Died	Patient who dies for any reason during the course of TB treatment.
Treatment default	Patient whose treatment was interrupted for two consecutive months or more during the treatment period.
Transfer out	Patient who was referred to a facility in another district to continue treatment and for whom the treatment outcome is not known.
Acquired resistance	Participants who are subsequently referred for MDR treatment.

For peer review only - http://bmjopen.bmj.com/site/about/guidelines.xhtml

6.2 Secondary outcomes

The following outcome measures will be recorded at the six-month follow-up:

- sputum conversion at the end of treatment in the group of participants who had bacteriology confirmed PTB at baseline [1]
- continuous smoking abstinence for identified smokers at baseline[2]

Whereas, the following will be assessed at three and six months follow-up:

- reduction in harmful or hazardous drinking[3]
- TB and ART medication adherence will be measured using a modified version of the AIDS Clinical Trials Group (ACTG) Adherence Questionnaire[4]; using an adherence index calculated by the formula (using the four-day recall table):

  [Total number of doses taken/Total number of doses prescribed] x 100

  Patients with at least 95% of adherence will be considered as having optimal adherence otherwise will be considered as having low (or suboptimal) adherence.
- increase in proportion of HIV-positive participants on ART at three and six months from baseline using standardised questions on the CRF.

6.2.1 Monitoring adverse events

Adverse events (AE) and serious adverse events (SAE) will be defined *apriori* and relevant information will be collected.

A **Serious Adverse Event (SAE)** is actually a special case of an adverse event where adverse outcomes are severe. It includes following events: Death of any of the participants associated with a clinical trial. **Examples of events**: Death, a life-threatening adverse event, inpatient hospitalization or prolongation of existing hospitalization, a persistent or significant.

An **adverse event (AE)** is any untoward medical occurrence in a patient or clinical investigation subject administered a pharmaceutical product and which does not necessarily have a causal relationship with this treatment.

The events are reported to ethics committee within 72 hours

[1] i.e. cure rates in intervention group versus control group for participants who initially had sputum AFB-positive, culture-positive or GeneXpert-positive PTB
[2] defined as a self-report of not smoking > 5 cigarettes six months from the start of the abstinence period, supported by a negative biochemical test CO < 7 ppm
[3] alcohol use will be measured using the AUDIT questionnaire. The questionnaire will be administered at screening (which will take place on the same day or shortly after the baseline assessment) and again at three months and six months.
[4] The questionnaire is a validated tool for measuring adherence specifically to ART and we will use an adapted version to also measure TB medication adherence [40].

For peer review only - http://bmjopen.bmj.com/site/about/guidelines.xhtml

The following information were collected to report the events:

Participants identification number, Gender, Age, Date of Enrollment, Arm (Control or intervention), Date of death notification to staff, Date of death , If death is related or not related to study.

For peer review only

For peer review only - http://bmjopen.bmj.com/site/about/guidelines.xhtml

6.3 Trial assessment schedule

Table 2 details the trial assessment schedule

Table 2: *Trial assessment schedule*

Assessment	*Items*	Pre randomisation	Timeline (post randomisation)				
		Eligibility (Day 0)	*Baseline (Day 0)*	*2 months*	*3 months*	*6-9 months*	*Trial end*
**ELIGIBILITY**							
Smoking status		X					
Smoking profile			X				
Alcohol profile		X					
Medical eligibility		X					
Eligible, consenting		X					
**MEASURES**							
Trial ID, visit date			X				
Socio-demographic history			X				
Depression screen			X		X	X	
Clinical review of TB Treatment record for disease information			X				X
Smoking history			X				
Smoking abstinence (self-report)					X	X	
Exhaled CO					X	X	
Record sputum culture or smear or Gene Expert result			X	X	X	X	
HIV Status			X				
ART Status (if HIV positive)			X		X	X	
AUDIT		X			X	X	
Modified ACTG (Follow up)					X	X	
Economic evaluation			X		X	X	

For peer review only - http://bmjopen.bmj.com/site/about/guidelines.xhtml

6.4 Other important information

In addition to the above, the following information was collected at baseline. Socio-demographic history included age, gender, marital status, education, employment status, and comorbidities. For details about these variables, see Table 3.

6.5 Fidelity of the intervention

The main fidelity analysis will be published somewhere else. Some descriptive statistics regarding fidelity will be added here once the main analysis is completed and the statistician is unblinded to the treatment arm.

For peer review only

7. Data

7.1 Data collection methods

The fieldworkers will screen all TB patients for eligibility immediately after the TB nurse at the clinic has initiated TB treatment and opened the TB "blue card". Consent will be obtained for this screening phase as the alcohol related questions are sensitive and the fieldworkers must gain insight in the patient files.

Eligible and consenting patients will be enrolled in the trial and the baseline questionnaire and record review completed. Patients will be given a unique Trial Number that will be used on all research documents. Data will be collected and recorded by field workers equipped with Android phones with a mobile data collection application installed.

Participants in the control arm will continue with the routine TB care. Intervention arm study participants will be referred by the fieldworker to the lay counsellor for motivational interviewing. The first MI session will be on the same day of the completion of the screening questionnaire, where possible (with a 2-week window period). The second and third MI session will be scheduled 4 weeks and 8 weeks from the first counselling session respectively each time with a 2-week window period.

MI counselling and data collection will take place in a well-ventilated private area inside or outside the clinic, and audio-recorded after consent obtained. Fieldworkers and LHWs will be provided with high particulate respirator masks to minimise the risk of infection.

Fieldworkers will follow-up all participants in both arms at 3 and 6 months within a window period of 2-weeks before and 2-weeks after the ideal 3 and 6-month visit. Participants will receive SMS reminders 3 days before each planned visit. Participants will also be in a position to send "please call me "messages to the fieldworkers or district coordinators, who will then call the participant to solve problems that may have arisen with the appointment.

Patients who did not return for the planned 3 and 6 months visit will be contacted by telephone up to 3-times, as needed. Home visits will also be undertaken by existing clinic "tracer teams" or Community Based Outreach teams -where feasible - for participants who cannot be traced by telephone. The data collection process is illustrated in Table 3.

For peer review only - http://bmjopen.bmj.com/site/about/guidelines.xhtml

Table 3: Data Collection Process by time-point and details of data collection method

**Time-point**	**Information required at time-point**	**Data collection method**
**Baseline interview**	**1. <u>Socioeconomic and demographic status (to include history of mine work)</u>**<br>• Age<br>• Gender<br>• Marital status<br>• Educational level achieved<br>• Employment<br>• Mine work/type of mine work<br><br>2. Clinical information:<br>• Patient category (First episode vs recurrence)<br>• Site of disease<br>• Results of sputum smear, culture and Gene Xpert<br>• HIV status<br>• ART information<br>• Co-morbidities<br><br>3. Current smoking status and quit history, second hand smoke exposure<br><br>4. Alcohol history<br>5. Depression	TB Treatment Record<br><br>CRF – Questions to participant – demographic details<br><br>CRF – Questions to participant and information from the TB Treatment Record (as indicated in CRF)<br><br>Questions adapted from Global Adult Tobacco Survey Questionnaire<br><br>Baseline AUDIT score<br>CES-D
**3 months**	1. ART information<br><br>2. TB and ART medication adherence (modified ACTCG)<br><br>3. Alcohol history (repeat AUDIT)<br><br>4. Smoking history<br>5. SLT use	CRF – Question to patient<br><br>ACTG questionnaire for both TB medication and ART<br><br>AUDIT score at 3 month

For peer review only - http://bmjopen.bmj.com/site/about/guidelines.xhtml

	6. Depression	Follow-up questions as per Russell's Standard and exhaled CO<br><br>CES-D
**6 months**	1. TB treatment status <u>(Primary outcome)</u><br><br>2. Sputum smear or culture result<br><br>3. ART information<br><br>4. TB and ART medication adherence<br><br>5. Alcohol history (repeat AUDIT)<br><br>6. Smoking history and exhaled CO<br><br>7. Depression	TB treatment outcome from TB Treatment Record combined with information from TB record on cultures and smear results.<br><br>CRF- Question to patient<br><br>Follow-up ACTG questionnaire at 6 months<br><br>AUDIT at 6 months<br><br>Questions as per Russell's Standard and exhaled CO<br><br>CES-D

For peer review only - http://bmjopen.bmj.com/site/about/guidelines.xhtml

7.2 Data management

Sefako Makgatho University (SMU) appointed a data manager who will utilise an electronic platform for data collection, ensuring data quality, and facilitating the SMS messages.

Fieldworkers collecting research data will be equipped with Android mobile phones, which will have a mobile application installed on them to allow for data collection in areas with poor internet connectivity. The electronic data captured will be stored on secure and password protected storage servers and mobile phones, which ensure data privacy through only allowing authorised research staff access to the data.

The electronic data collection system used for the study requires an SMS gateway to send and receive messages to the research participants. Consenting participants' phone numbers, participant IDs, and associated SMS messages will be stored on the SMS gateway's secured and password-protected server.

Data quality will be ensured by providing fieldworkers with standard operating procedures (SOPs), training, and ongoing support on the importance of data quality, data collection, and data collection problem-solving. The data manager will continuously monitor the captured data for missing variables and inconsistencies in order to resolve any data problems.

The data manager will export the data from the secured server, conceal the participants study arm allocation, and de-identify the data before sharing the data in STATA and R compatible formats. The exported de-identified data will be stored in Dropbox, a secure cloud storage platform, for sharing with the lead trial statistician at the University of York for analysis.

All research data and documents referring to the PROLIFE trial will be stored and maintained in a secured storage space at SMU for a minimum of 15 years from the end of the PROLIFE trial. Study materials will be destroyed 15 years after the study.

For peer review only - http://bmjopen.bmj.com/site/about/guidelines.xhtml

8. Analysis

The computer packages STATA 16 (StataCorp. 2019) and R 3.5.3 (ref) will be used. Significance tests will be two-sided and the significance level is set at 0.05. The statistician will remain blind to allocation until results are finalised. We will follow the CONSORT statement guidelines in reporting.

Below, we detail the analyses that we will carry out for the data collected at baseline, the primary outcome, the secondary outcomes, and adverse events. We also list the sensitivity analyses that we might perform and subgroup analyses.

For peer review only

8.1 Participants Flow

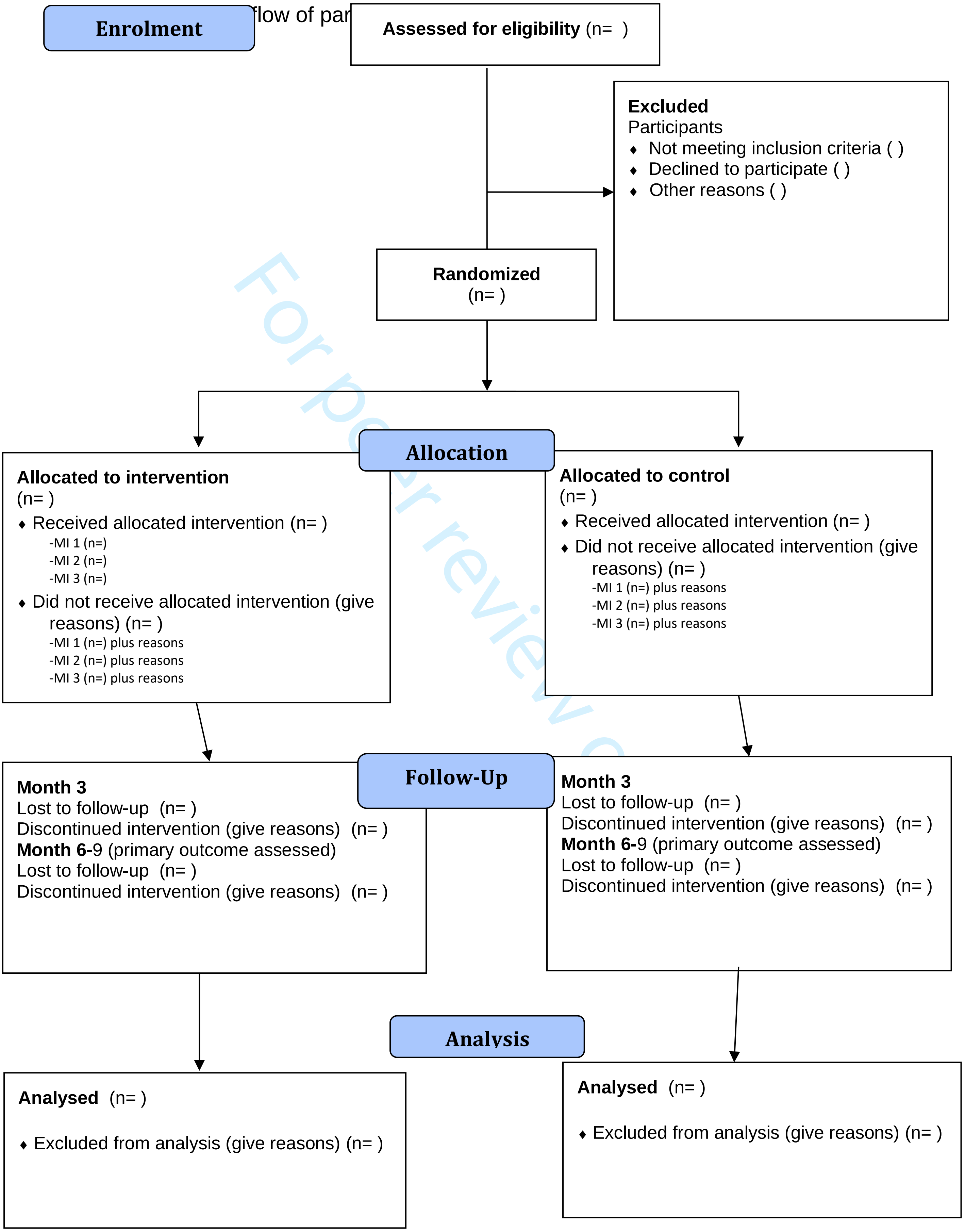

For peer review only - http://bmjopen.bmj.com/site/about/guidelines.xhtml

Figure 1 CONSORT DIAGRAM SHOWING FLOW OF PARTICIPANTS

8.2 Baseline data

Baseline data Analysis Plan:

Baseline data including demographic variables will be summarised descriptively by trial arm, but no formal statistical comparisons will be undertaken. Continuous measures will be reported as means and standard deviations (SD) while categorical data will be reported as counts and percentages, see Tables 1 to 4 in Appendix A. For skewed continuous measures, we will also provide medians and interquartile ranges.

For peer review only

Baseline data results:

Consent:

In the control arm (Arm1) 286 gave written consent and 5 verbal consent for participation in the study and for access to their medical records

In the intervention arm (Arm2) 279 gave written consent and 4 verbal consent for participation in the study and for access to their medical records

Baseline Imbalances

Education:

It seems that for education there is imbalance between the two arms for Grades 8-11/Grade 12/Higher; the control arm (Arm1) had a higher educated group the difference in percentage points 8.2% as opposed to 2.8% in the intervention arm, see Table 2 for further details. There are 9 participants who are not literate in the study (7 in the control arm (Arm1) and 5 in the intervention arm (Arm2)).

Drinking

In the intervention arm, 223 (78.8%) had a drink in the past 12-months compared to 208 (71.5%) in the control arm, see Table 3.

In the control arm (Arm1), 110 (37.8%) were drinkers only, 83 (28.5%) were smokers only, and 98 (33.7%) were smokers and drinkers compared to 92 (32.5%), 60 (21.2%), and 131 (46.3%) in the intervention arm (Arm2), respectively.

For peer review only - http://bmjopen.bmj.com/site/about/guidelines.xhtml

Assets:

The vast majority had a radio (84%), a television (89%), a refrigerator (85%), an electric or gas stove (86%), and a microwave (67%), however, only 50% had a washing machine. Furthermore, a minority had a landline telephone (7.5%), a desktop or laptop computer (20%:), and a vacuum cleaner or floor polisher (15%). Figure 2 is a boxplot of the total number of assets by study arm. The spread in the control arm (Arm1) is greater than that in the intervention arm (Arm2). However, the mean number of assets is similar across the two groups of 5 (SD: 1.87).

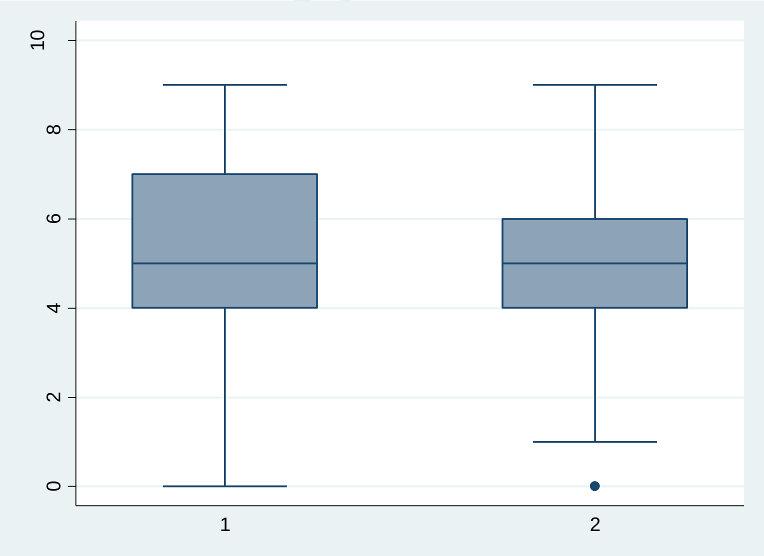

Figure 2: A boxplot of the total number of assets by study arm

For peer review only - http://bmjopen.bmj.com/site/about/guidelines.xhtml

Medical History:

There were 305 (53.2%) **HIV positive** participants out of which 204 (65.38%) were using Cotrimoxazole and 257 (82.37%) were undergoing anti-retroviral therapy, see Table 2.

TB History: The vast majority of participants were New TB patients 513(91.3%), with Pulmonary only (ICD-10 A15) being the site of disease for the vast majority 553 (98.9%). Among those with results known, the majority had one pre-treatment smear result 366 (89.9%), one Gene XPert recorded 435 (97.5%), and one culture result recorded 96 (96%).

Among those with results available, 220 (58.51%) had at least **one positive smear** result,362 (87.23%) had at least **one positive Gene XPert result**, and 35 (47.95%) at least **one positive culture result**.

However, 85 (53.46%) in the control arm had at least one positive smear result within 60 days of the TB treatment start date compared to 96 (61.15%) in the intervention arm.

In addition, only 34 had their culture results within 60 days from the TB start dates of which 21 had positive results; 11 in the control arm compared to 10 in the intervention arm.

The vast majority of participants did not have any **co-morbidities** 525 (96.3%) and 18 (3.3%) had one.

For peer review only - http://bmjopen.bmj.com/site/about/guidelines.xhtml

Baseline Smoking Related variables:

Thirty five percent (202) did not smoke in the past month, whereas 52% smoked daily and the remaining 13% smoked in the past month but less frequently than on a daily basis, see Table 3.

In the past 30 days, the vast majority were exposed to smoke for seven days or less at: home 462 (80.5%); cafes/restaurants 464 (80.8%); Shebeens, bars or clubs 432 (75.3%); Bus/train/taxi/ vehicle 448 (78.0%); and Shops/shopping mall 461 (80.3%) and where applicable at the workplace 173 (66.8%).

Among those who smoked in the past month 372: 181 in the control arm (Arm1) vs 191 in the intervention arm (Arm2),

345 (92.7%) **smoked manufactured cigarettes** of which 225(65%) did so on a daily basis in the past seven days whereas 40 (11.6%) did not smoke in the past week. The mean number of days smoked was 5.42 days in the past week (SD: 2.49) with 6.34 cigarettes (SD: 7.39) smoked daily on average.

Hand-Rolled cigarettes were used by 35 participants of which 16 (45.7%) smoked daily in the past week. They smoked on average for 4.11 (SD:2.91) days in the past week and on average smoked 3.77 (SD: 3.27) hand-rolled cigarettes per day.

There was only one person who exclusively smoked waterpipe, two who exclusively smoked pipe, and seven who exclusively used other formats of tobacco other than the ones that are listed here.

For the vast majority the total number of cigarettes smoked is based on their answer to manufactured cigarettes. In 27 cases, they supplemented this with other sources and 8 used only other forms to report the number smoked on average per day.

Over the past 3 months, they **spent** on average 174.58 (SD: 181.61) Rands per week on tobacco products.

For peer review only - http://bmjopen.bmj.com/site/about/guidelines.xhtml

Smoking restrictions

No smoking was allowed **inside home** for 204(54.8%) participants, whereas 119(32.0%) had some rule where/when it is allowed and 49(13.2%) had no rules in place.

Quit Attempts:

Among smokers, 116 (31.2%) made an **attempt to quit**; the mean number of attempts to quit was 2.56 (SD: 2.32).

Furthermore, 213 (57.3%) said they will probably **try to quit** smoking completely and permanently in the next three months and 126 (33.9%) said that they definitely will.

Whereas, 207 (55.6%) said they will probably **quit** smoking completely and permanently in the next three months and 130 (34.9%) said that they definitely will.

Only 39 (10.5%) have ever used any methods in the past 3 months to help them stop smoking tobacco. These spent on average 136.64 (SD: 205.36) Rands on methods to help you stop smoking in the past 3 months.

Smokeless tobacco was used by only 24 participants (4.2%). They have been using ST for an average of 10.5 years (SD: 8.8) and have started using it at the age of 27 (SD: 11.33) years on average.

Heaviness of smoking:

In the control arm (Arm1), 67(37.0%) of smokers reported smoking within 5 minutes of waking up whereas 82(42.9%) did so in the intervention arm (Arm2).

Among those who smoked and who reported the number of cigarettes/pipes/cigars they used on average per day, 134(74.03%) in the control arm (Arm1) and 158(82.72%) in the intervention arm (Arm2) were considered as heavy smokers.

For peer review only - http://bmjopen.bmj.com/site/about/guidelines.xhtml

8.3 Primary analysis

8.3.1 Primary outcome definition:

As per section 6.1, we would define the primary outcome as those who had a successful treatment versus not. Where successful treatment is considered if the patient is considered to have been cured or treatment completed; all other categories will be deemed as not successful. The successful treatment[5] categories are defined as follows:

Cure	Patient in whom baseline smear or culture was positive at beginning of treatment AND is smear/culture negative in the last month of treatment and on at least one previous occasion at least 30 days prior According to local protocol, a patient who is diagnosed using Gene Xpert and is sputum negative for TB at 11 and 23 weeks is considered 'Cured'.
Treatment completed	Patient whose baseline smear or culture was positive at the beginning and has completed treatment but does not have a negative smear/culture in the last month of treatment and on at least one previous occasion > 30 days prior. Patients diagnosed with PTB whose baseline smear (or culture) result was negative and who started treatment based on clinical and radiological findings who have shown clinical improvement and completed the prescribed course of treatment. *N.B. The smear examination may not have been done or the results may not be available at the end of treatment.*

8.3.2 Primary outcome Analysis

For the primary outcome, we will conduct analysis on an intention-to-treat basis. We will use binary logistic regression to compare the main outcome between the

[5] Following discussion with the team, we will take this at face value as it is not possible to query some of the anomalies found in the recording of dates of tests.

intervention and the usual care arm. We will also investigate any potential clustering at the centre level and account for it. We will present the results for this analysis in Table 6.

We will also adjust for HIV status, sex, alcohol versus tobacco versus both, and district; if these differ between trial arms at baseline.

8.3.3 Primary outcome Analysis Results

Table 4 gives the descriptive statistics for the initial outcome and the derived - dichotomised outcome; these indicate that overall, 69% were classified as successful treatment based on the medical professional assessment (cured/treatment completed). This percentage was similar for the two arms with the control arm (Arm1) having a slightly higher percentage of success of 70.1% compared to 67.8% in the intervention arm (Arm2)[6]. Table 9 gives the distribution per centre by study arm. Generally, these are fairly balanced any imbalance observed is most probably due to the early termination of the study.

For 203 participants, the TB treatment outcome date was not available, with those participants more likely to not have been cured (59% not cured), however, the percentage was similar across the two study arms for those with missing TB treatment outcome date.

The odds of successful treatment is 0.9 (95% CI: (0.64,1.27)) in the intervention arm (Arm2) compared to the control arm (Arm1). This estimate is very similar to the estimate adjusting for district and drinking/smoking status. This is also the case if you further adjust for sex and HIV status at baseline where the OR is 0.86 (95% CI: (0.60,1.24)); see Table 6 for further details.

[6] Primary outcome Control: 204/291, 95% CI proportion 0.70 (0.64,0.75)
Primary outcome Intervention:192/283, 95% CI proportion 0.68 (0.62,0.73)

For peer review only - http://bmjopen.bmj.com/site/about/guidelines.xhtml

8.4 Secondary analyses

Secondary analyses plan

In the group of participants who had bacteriology confirmed at baseline, we will use logistic regression to compare cured versus not cured, as indicated by the outcome at the end of treatment, between the two study arms. We will also control for baseline characteristics and other covariates such as sex, alcohol use, HIV-status, district, and account for any potential clustering by centre.

We will use a similar approach for the six-months continuous smoking abstinence outcome. This analysis will be performed on the group of participants who were current tobacco smokers at baseline. We will also control for baseline characteristics and other covariates such as age, duration of smoking, alcohol problem (hazardous, harmful, non-drinker/light drinker), heaviness of smoking index[7], depression, and potentially HIV-status.

For the reduction in harmful or hazardous drinking, we will use linear regression to measure difference in total AUDIT score between control and intervention groups accounting for the baseline AUDIT scores. Separate analyses for the AUDIT at 3 and 6 months will be performed.

The AUDIT is a 10-items questionnaire with a range between 0 and 40 where higher values indicate higher dependency.[8] It is worth mentioning that a score of 8 or more

For peer review only

[7] [Goedele's Comment on an earlier Version]Definitely a measure of severity of smoking and duration of smoking. For example Heaviness of Smoking Index which can be derived as follows:

HSI=Heaviness of smoking index ≥ 4, calculated based on sum of time to first cigarette (0: 61+min, 1:31-60 min, 2: 6-30 min, 3: ≤5 min) and number of cigarettes smoked per day (0: 0-10 cigarettes per day [CPD]

Also: age, duration of smoking, alcohol problem (hazardous, harmful, non-drinker/light drinker), maybe HIV-status, (adding this may reduce your sample size too much, because of missing HIV-status, unless you include HIVstatus unknown as a category of HIV-status). Depression

[8] Scoring the audit
Scores for each question range from 0 to 4, with the first response for each question (eg never) scoring 0, the second (eg less than monthly) scoring 1, the third (eg monthly) scoring 2, the fourth (eg weekly) scoring 3, and the last response (eg. daily or almost daily) scoring 4. For questions 9 and 10, which only have three responses, the scoring is 0, 2 and 4 (from left to right).
A score of 8 or more is associated with harmful or hazardous drinking, a score of 13 or more in women, and 15 or more in men, is likely to indicate alcohol dependence.

For peer review only - http://bmjopen.bmj.com/site/about/guidelines.xhtml

is associated with harmful or hazardous drinking, a score of 13 or more in women, and 15 or more in men, is likely to indicate alcohol dependence. Eligibility criteria for our study is an AUDIT score ≥ 8 for men or ≥ 7 for women but <20. However, if assumptions of linear regression are not met we will either transform the data or use alternative regression analyses such as ordinal logistic regression.

Adherence to TB and ART medication will be measured using an adherence index based on a modified version of the AIDS Clinical Trials Group (ACTG) Adherence Questionnaire; where patients with at least 95% of adherence will be considered as having optimal adherence otherwise will be considered as having low (or suboptimal) adherence. We will use logistic regression to model patient's characteristics (age, sex, alcohol, smoking status, depression) that might influence adherence at 6-month; we will also compare adherence between study arms. Similar to the previous outcomes, we will account for any potential clustering by centre.

We will also report the proportion of HIV-positive participants on ART at six months and compare these to the baseline using standardised questions on the CRF.

8.4.1 Secondary outcome definitions that involve defining a positive baseline test:

To operationalise the above we need to define how we determine if someone has a positive baseline smear or culture. Each participant might have up to two tests of the following: smear test, GeneExpert test, and culture test.

If a test was administered two times then to be considered negative both tests should be negative, otherwise it is considered positive. If a test was administered only once, then the result of that instance is taken as is.

1Saunders JB, Aasland OG, Babor TF et al. Development of the alcohol use disorders identification test (AUDIT): WHO collaborative project on early detection of persons with harmful alcohol consumption — II. Addiction 1993, 88: 791–803

For peer review only - http://bmjopen.bmj.com/site/about/guidelines.xhtml

If for a patient more than one test was administered, then to be considered negative the result should be negative under both tests.

This is illustrated in the following table.

Baseline	P=+, N = -		
		XXXX Test at run 2 (Result 2)	
XXXX Test run 1 (Result 1)		N	P
	N	NN	NP
	P	PN	PP
XXXX=Smear or Culture or GeneXpert			
Smear test at baseline	N1	NN	
	P1	NP, PN, PP	
Genexpert	N2	NN	
	P2	NP, PN, PP	
culture	N3	NN	
	P3	NP, PN, PP	
Positive at baseline	PB	P1 or P2 or P3	
Negative at baseline	NB	N1 and N2 and N3	

To define a conversion among those who were positive at baseline, we used the primary outcome response category cured to indicate a negative result at month 6 per the nurse's assessment.

Among the 403 participants who were positive at baseline 168(41.69%) were recorded as cured by 6-month of these 83 (39.9%) in the control arm compared to 85 (43.59%) see Table 4. The odds ratio of conversion is 1.16 (95% CI: (0.83,1.63)) comparing the intervention arm to the control arm. When adjusting for district, sex, and smoking/drinking status and HIV status at baseline, the OR reduces to 1.07 (95% CI: (0.76,1.51))

For peer review only

For peer review only - http://bmjopen.bmj.com/site/about/guidelines.xhtml

8.4.2 6-months Continuous Abstinence

For this outcome, those who smoked cigarettes at baseline were considered as the analytic sample. In addition, those where the following could not be ascertained: self-report of not smoking > 5 cigarettes six months from the start of the abstinence period and supported by a negative biochemical test CO < 7 ppm were considered as smokers for the analysis of this variable. The number of participants who identified as cigarette smokers at baseline were 345 (60.1%)[9].

23 (85.19%) out of 27 with three measurements available[10] managed to abstain continuously for six months. These were similarly distributed across the two study arms, see Table 4. Among those who identified as cigarette smokers, 10 (5.59%) participants in the intervention arm continuously abstained. In the control arm there were 12 (7.23%) who continuously abstained for 6-months.

The crude odds of 6-months continuous abstinence is 0.76 (95% CI: (0.35,1.63)) in the intervention arm compared to the control arm among baseline cigarette smokers. Given the limited number of those who were identified as continually abstained, we were only able to adjust for one additional variable at a time. Adding one of the following variables: heaviness of smoking, type of drinker at baseline, age when started smoking, and the duration of smoking at baseline, the adjusted odds ratio of continuous abstinence comparing the intervention to the control arm did not differ much from the crude estimate of 0.76. The adjusted estimate for the various models ranged between 0.73 and 0.76 with similar confidence limits as for the crude estimate. Furthermore, we did not have evidence that any of the adjusting variables were statistically significantly correlated to continuous abstinence in these models.

We carried an additional analysis where those who died and were smokers at baseline, 22 in total, 20 were cigarette smokers and were removed from the analytical sample for the continuous abstinence outcome. This resulted in a crude

[9] Any type of tobacco smoking at baseline 372 (64.8%). The numbers reflect those who used manufactured cigarettes which were the vast majority.

[10] We had only 27 participants who had self-report of not smoking at 3 months, 6 months and a carbon monoxide reading at 6 months. Continuous abstinence was defined as a self-report of not smoking > 5 cigarettes six months from the start of the abstinence period and supported by a negative biochemical test CO < 7 ppm

For peer review only - http://bmjopen.bmj.com/site/about/guidelines.xhtml

OR of 0.78 which is similar to the OR when the larger analytic sample was considered; this was also the case for the associated 95% CI. Similar estimates were observed when adjusting for the aforementioned variables.

We carried an additional analysis where the analytic sample was all those who were smokers regardless of the type used. There were a total of 372 that identified as smokers. The crude odds ratio in this case changes to 0.86 (95% CI: (0.38,1.95)). The 95% CI is similar to that of the smaller analytic sample. When adjusting for the heaviness of smoking, type of drinker at baseline, age when started smoking, and the duration of smoking at baseline, the adjusted odds ratio of continuous abstinence comparing the intervention to the control arm did not differ much from the crude estimate.

3-months Continuous Abstinence

Among those who identified as cigarette smokers, 20 (11.17%) participants in the intervention arm continuously abstained for 3-months while in the control arm there were 27 (16.27%) who continuously abstained for 3-months.

The crude odds of 3-months continuous abstinence is 0.65 (95% CI: (0.37,1.14)) in the intervention arm compared to the control arm among baseline cigarette smokers. Given the limited number of those who were identified as continually abstained, we were only able to adjust for one additional variable at a time. Adding one of the following variables: heaviness of smoking, type of drinker at baseline, age when started smoking, and the duration of smoking at baseline, the adjusted odds ratio of continuous abstinence comparing the intervention to the control arm did not differ much from the crude estimate of 0.65. The adjusted estimate for the various models ranged between 0.63 and 0.66 with similar confidence limits as for the crude estimate.

For peer review only - http://bmjopen.bmj.com/site/about/guidelines.xhtml

Point Abstinence

There were 57 participants who indicated that they stopped smoking tobacco completely at the 3-month follow-up, of which one had more than 5 cigarettes in the past 3 months. There were 81 participants who indicated that they stopped smoking tobacco completely at the 6-month follow-up, of which three had more than 5 cigarettes in the past 3 months. Only 30 participants had information for the entirety of the 6-month period, of which none consumed more than 5 cigarettes over the past 6-month period. Of these, 23 had a confirmed CO < 7 ppm, 4 had these levels >= 7, and 3 were missing.

182 responded that they continued smoking either as usual or at a reduced rate but regularly at month 3; whereas 133 done so at month 6 of these we had 97 that had measurements at both time points thus resulting in a total of 218 where they have smoked on a regular basis over the past 6 month period. 11 had responded as not smoking in the past three months at month 6 but had missing information for the first three months; 9 of these had carbon monoxide readings available at month 6. Of these nine, two had their CO >= 7 (in fact these were 10 & 10.1).

For peer review only - http://bmjopen.bmj.com/site/about/guidelines.xhtml

8.4.4 **Change in harmful or hazardous drinking at month 3 and 6 follow-ups**

Alcohol use was measured using the AUDIT questionnaire. We will assess whether there has been a reduction in alcohol consumption three months and six months following recruitment. This analysis will be performed on the group of participants who were harmful or hazardous drinkers at baseline. Figure YYY provides histograms of the AUDIT score at baseline, 3-months and 6-months, respectively, among those who were considered as hazardous or harmful drinkers. It also presents scatterplots of the 3-months and 6-months scores versus the baseline scores for this group by trial arm, respectively.

Among those who were harmful or hazardous drinkers at baseline those in the intervention arm had on average a reduction of 0.04 points (95% CI: (-2,1.91)) on the AUDIT score at 6-months compared to those in the control arm controlling for their baseline score, see Table 6. However, when additionally adjusting for district, sex, and smoking/drinking status (which effectively flags smokers/non-smokers who are also drinkers) and HIV status at baseline; the intervention arm had an average increase of 0.02 points on the AUDIT score (95% CI: (-1.55,1.6)) compared to the control arm. It is worth noting that of the variables in the adjusted model; the only statistically significant result is for the district variable. It seems that those in district "S" score on average 5.8 points less than those in "B" (95% CI: (-11.26,-0.35)); similarly those in "L" score 5 points less than those in "B" on AUDIT but we do not have evidence that this difference is statistically significant (95% CI: (-10.35,0.26)), see Table 6 for further details.

At 3-month, the estimates were an average increase of 0.55 (95% CI: (-1.01,2.11)) on the AUDIT score in the intervention arm compared to the control arm when only accounting for the baseline scores; whereas it increased to 0.74 (95% CI: (0.62,2.1)) when adjusting for other covariates in the model, for further details see Table 7.

For peer review only - http://bmjopen.bmj.com/site/about/guidelines.xhtml

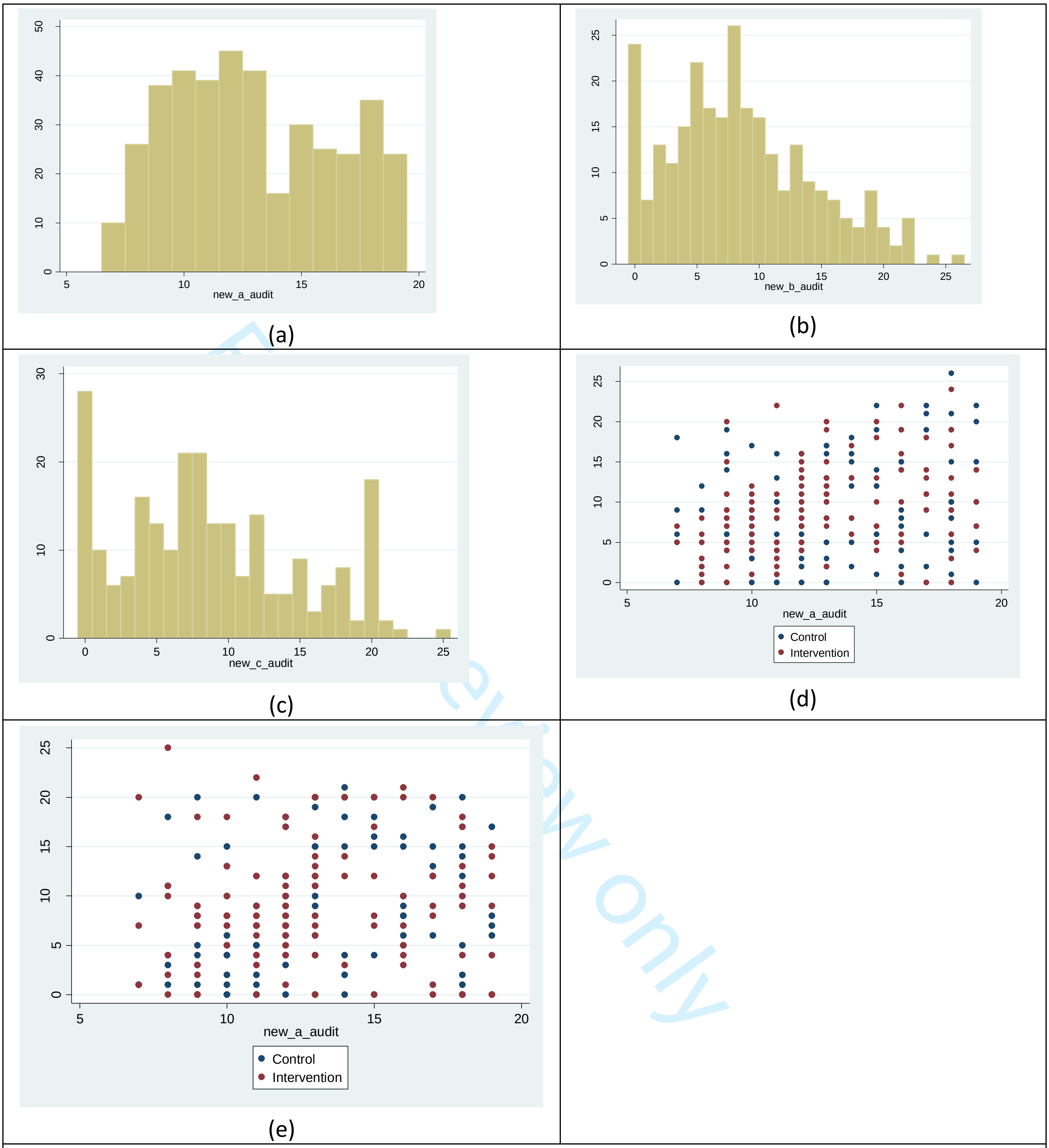

Figure YYY: Graphs (a), (b), and (c) are histograms of the AUDIT score at baseline, 3-months and 6-months, respectively, among those who were considered as hazardous or harmful drinkers. (d) and (e) are scatterplots of the 3-months and 6-months scores versus the baseline scores for this group, respectively.

For peer review only - http://bmjopen.bmj.com/site/about/guidelines.xhtml

8.4.5 Proportion of ART uptake of HIV-positive participants at month 3 and 6 follow-ups

We will assess whether there has been an increase in proportion of HIV-positive participants on ART at three and six months from baseline using standardised questions on the CRF.

There were 171 HIV-positive participants whose baseline ART medication status was known and whose ART medication status was known as well at 6-months. Of these, 123 remained on their medication, 19 took up medication at 6-months compared to not taking medication at baseline, whereas 29 stopped taking their medication at 6-months but were on medication at baseline, see Table 6 for further details.

There were 10 who had an unknown status in terms of medication at baseline and no information was available about them at 6-months, 12 who were initially of unknown medication status who took up medication at 6-months (these were equally distributed between the two arms). Furthermore, there were 10 who were not taking medication at baseline and 102 who were taking medication at baseline whose 6-months medication status was not recorded.

At 6-months, the odds ratio of taking medication at 6-months was 2.05 (95% CI: (0.80,5.27)) in the intervention arm compared to the control arm, controlling for ART baseline medication status.

There were 188 HIV-positive participants whose baseline ART medication status was known and whose ART medication status was known as well at 3-months. Of these, 122 remained on their medication, 16 took up medication at 3-months compared to not taking medication at baseline, whereas 50 stopped taking their medication at 3-months but were on medication at baseline, see Table 7 for further details.

There were 9 who had an unknown status in terms of medication at baseline and no information was available about them at 3-months, 11 who were initially of unknown

For peer review only - http://bmjopen.bmj.com/site/about/guidelines.xhtml

medication status who took up medication at 3-months, and two who were initially of unknown medication status who were not taking medication at 3-months. Furthermore, there were 9 who were not taking medication at baseline and 82 who were taking medication at baseline whose 3-months medication status was not recorded. Furthermore, there were 4 who carried on not taking medication at 3-months.

At 3-months, the odds ratio of taking medication at 6-months was 0.79 (95% CI: (0.38,1.65)) in the intervention arm compared to the control arm, controlling for ART baseline medication status.

Medicine Adherence:

At 3-months 165 (98.8%) of 167 participants had optimal ART medication adherence, whereas 139(97.2%) of 143 had optimal ART medication adherence. These were similar across the two arms.

Similarly, at 3-months 319(91.67%) of 348 participants had optimal TB medication adherence, whereas 120(90.23%) of 133 had optimal TB medication adherence. These were similar across the two arms.

8.5 Subgroup analyses (See above analyses)

We will conduct subgroup analyses to determine whether TB treatment outcomes differ between subgroups, as follows: HIV-positive versus HIV-negative participants; participants with an alcohol problem only versus smokers only versus participants who are conjoint smokers and drinkers; and participants who were GeneXpert positive versus participants who were GeneXpert negative at baseline.

8.5 Sensitivity analyses

In case of missing data, multiple imputations and appropriate sensitivity analyses will be conducted. As it is likely that more than one variable will have missing data we will use multiple imputations using chained equations (**MICE**). A minimum of 10 imputations will be performed; however, the final number of imputations will depend on the missing in the data. We will report the decisions that we make with regard to the number of imputations and the

For peer review only - http://bmjopen.bmj.com/site/about/guidelines.xhtml

variables we use in the imputations. We will also conduct a sensitivity analysis to explore the implications of the missing at random assumption [21p,22p].

8.6 Adverse events

Analysis of adverse events and serious AE will explore whether these differ by treatment arm using Chi-square tests.

8.7 Planned interim review and analyses

No interim analysis is planned. The main analyses will be completed after three months of the data closing.

For peer review only

For peer review only - http://bmjopen.bmj.com/site/about/guidelines.xhtml

8.8 List of Tables and Graphs

Measures of central tendency and percentages will be reported to two decimal places whereas measures of variability and p-values will be reported to three decimal places.

The following is a list of suggested tables and graphs; the templates are included in Appendix A.

TABLE 1: Numbers in the study at Baseline and follow-ups at month 3, and 6 by centre and study arm.

TABLE 2: Descriptive statistics for socio-demographic and socio-economic characteristics at baseline and as analysed by study arm. Frequencies and (percentages) are presented unless otherwise stated

TABLE 3: Descriptive statistics for smoking history, alcohol history, clinical characteristics and depression score at baseline and as analysed by study arm. Frequencies and (percentages) are presented unless otherwise stated.

TABLE 4: Descriptive statistics for primary and secondary outcomes by study arm at baseline (where available), 3 month (where available) and 6 month. Frequencies and (percentages) are presented unless otherwise stated.

TABLE 5: Number and type of adverse events at month 2, 3, and 6 by centre and study arm.

TABLE 6: Regression analysis results for the primary and secondary outcomes at 6 month. Estimates presented with corresponding 95% CI. Crude and adjusted estimates are provided.

TABLE 7: Regression analysis results for the secondary outcomes that are measured at 3 month. Estimates presented with corresponding 95% CI. Crude and adjusted estimates are provided.

For peer review only

For peer review only - http://bmjopen.bmj.com/site/about/guidelines.xhtml

9. SAP amendment log

Amendment/addition to SAP and reason for change	New version number, name and date
SAP completed and signed-off	*V1.0,*
Updated verion	*V1.1, May 2020*

10. Signatures of approval

Sign-off of the final approved version of the Statistical Analysis Plan by the principle investigator and trial statistician(s) (can also include Trial Manager/Co-ordinator)

**Name**	**Trial Role**	**Signature**	**Date**

For peer review only - http://bmjopen.bmj.com/site/about/guidelines.xhtml

11. References

Moriarty, A.S., Louwagie, G.M., Mdege, N.D. *et al*. ImPROving TB outcomes by modifying LIFE-style behaviours through a brief motivational intervention followed by short text messages (ProLife): study protocol for a randomised controlled trial. *Trials* **20**, 457 (2019) doi:10.1186/s13063-019-3551-9

Department of Health, Republic of South Africa. National Tuberculosis Management Guidelines. 2014. http://www.tbonline.info/media/uploads/documents/ntcp_adult_tb-guidelines-27.5.2014.pdf. Accessed 8 Jan 2018

21p. James R Carpenter and Michael G Kenward. Missing data in clinical trials — a practical guide. Birmingham: National Health Service Co-ordinating Centre for Research Methodology, 2008.

22p. James R Carpenter, Michael G Kenward, and Ian R White. Sensitivity analysis after multiple imputation under missing at random — a weighting approach. Statistical Methods in Medical Research, 16:259–275, 2007.

StataCorp. 2019. *Stata Statistical Software: Release 16*. College Station, TX: StataCorp LLC.

R Core Team (201?). R: A language and environment for statistical computing. R Foundation for Statistical Computing, Vienna, Austria. URL http://www.R-project.org/.

For peer review only - http://bmjopen.bmj.com/site/about/guidelines.xhtml

12. Appendix A (Results tables)

Table 1: Numbers in the study at Baseline and follow-ups at month 3 and 6 by centre and study arm. (SEE FLOWCHART)

TABLE 1: NUMBERS IN THE STUDY AT BASELINE AND FOLLOW-UPS AT MONTH 3 AND 6 BY CENTRE AND STUDY ARM.						
Centre	Baseline		Month 3		Month 6	
	Intervention	Control	Intervention	Control	Intervention	Control
Total						

For peer review only

TABLE 2: Descriptive statistics for socio-demographic, socio-economic, and clinical characteristics at baseline by study arm. Frequencies and (percentages) are presented unless otherwise stated

TABLE 2: DESCRIPTIVE STATISTICS FOR SOCIO-DEMOGRAPHIC, SOCIO-ECONOMIC, AND CLINICAL CHARACTERISTICS AT BASELINE AND AS ANALYSED BY STUDY ARM. FREQUENCIES AND (PERCENTAGES) ARE PRESENTED UNLESS OTHERWISE STATED				
		Baseline		
	N1/N2	Control	Intervention	Total
		Arm 1	Arm 2	
**Age in years: mean (SD)**	291/283	39.37 (12.60)	38.56 (11.15)	
**Age in years: median (IQR)**				
**Gender**				
Female		69 (23.7)	60 (21.2)	129 (22.5)
Male		222 (76.3)	223 (78.8)	445 (77.5)
Do not want to disclose				
Marital status				
Married or living together		102 (35.1)	95 (33.6)	197 (34.3)
Divorced/separated		20 (6.9)	18 (6.4)	38 (6.6)
Widowed		10 (3.4)	7 (2.5)	17 (3.0)
Never married and never lived together		144 (49.5)	150 (53.0)	294 (51.2)
Declined to answer		15 (5.2)	13 (4.6)	28 (4.9)
**Education**				
No education		7 (2.4)	5 (1.8)	12 (2.1)
Grades 1-5		23 (7.9)	20 (7.1)	43 (7.5)
Grades 6-7		32 (11.0)	35 (12.4)	67 (11.7)

For peer review only - http://bmjopen.bmj.com/site/about/guidelines.xhtml

For peer review only

Grades 8-11		96 (33.0)	128 (45.2)	224 (39.0)
Grade 12		87 (29.9)	70 (24.7)	157 (27.4)
Higher		24 (8.2)	8 (2.8)	32 (5.6)
Declined to answer		22 (7.6)	17 (6.0)	39 (6.8)
**Employment**				
Self-employed (full-time)		30 (10.3)	36 (12.7)	66 (11.5)
Employed full-time (30 hrs a week or more)		62 (21.3)	54 (19.1)	116 (20.2)
Employed part-time (less than 30 hrs a week)		19 (6.5)	29 (10.2)	48 (8.4)
Retired		17 (5.8)	16 (5.7)	33 (5.7)
Unemployed (but able to work)		125 (43.0)	120 (42.4)	245 (42.7)
Unable to work because of long-term disability or ill health		9 (3.1)	8 (2.8)	17 (3.0)
Full-time student		12 (4.1)	4 (1.4)	16 (2.8)
Caring from my home and family/doing household work/housewife		0 (0.0)	2 (0.7)	2 (0.3)
Occasional work ("piece job")		17 (5.8)	12 (4.2)	29 (5.1)
Declined to answer		0 (0.0)	2 (0.7)	2 (0.3)
**Ever worked or spent time in mines**				
No		244 (83.8)	237 (83.7)	481 (83.8)
Yes		46 (15.8)	45 (15.9)	91 (15.9)
**Socioeconomic status**				

For peer review only - http://bmjopen.bmj.com/site/about/guidelines.xhtml

**Household items (Yes is displayed)**				
A radio		249 (85.6)	234 (82.7)	483 (84.1)
A television		255 (87.6)	255 (90.1)	510 (88.9)
A landline telephone		21 (7.2)	22 (7.8)	43 (7.5)
A desktop or laptop computer		67 (23.0)	48 (17.0)	115 (20.0)
A refrigerator		248 (85.2)	240 (84.8)	488 (85.0)
A vacuum cleaner or floor pol		49 (16.8)	35 (12.4)	84 (14.6)
A microwave oven		198 (68.0)	189 (66.8)	387 (67.4)
An electric or gas stove		254 (87.3)	238 (84.1)	492 (85.7)
A washing machine		153 (52.6)	136 (48.1)	289 (50.3)
Total number of assets: mean (SD)		5.14 (1.96)	4.94 (1.77)	5.04 (1.87)
**In the past month, number of days you or people in the household went to bed hungry because there was no food to eat**				
0 days		244 (83.8)	238 (84.1)	482 (84.0)
1-7 days		45 (15.5)	34 (12.0)	79 (13.8)
More than 7 days		2 (0.7)	9 (3.2)	11 (1.9)
Declined to answer		0 (0.0)	2 (0.7)	2 (0.3)
**TB and medical history**				
**TB history**				
**Patient category**				
New patient		264 (92.3)	249 (90.2)	513 (91.3)
Relapse		10 (3.5)	9 (3.3)	19 (3.4)
Re-treatment after default		9 (3.1)	14 (5.1)	23 (4.1)
Re-treatment after failure		1 (0.3)	2 (0.7)	3 (0.5)
Other		2 (0.7)	2 (0.7)	4 (0.7)
**Site of disease**				
Pulmonary and Extra Pulmonary (ICD-10 A17-A19)		3 (1.1)	3 (1.1)	6 (1.1)

For peer review only - http://bmjopen.bmj.com/site/about/guidelines.xhtml

Pulmonary only (ICD-10 A15)		281 (98.9)	272 (98.9)	553 (98.9)
**Number of pre-treatment smear results**				
One		197 (92.1)	169 (87.6)	366 (89.9)
Two		17 (7.9)	24 (12.4)	41 (10.1)
**Smear result**	N =	196	180	376
**At least one positive smear result**		111 (56.63)	109 (60.56)	220 (58.51)
**Number of Gene XPert results recorded**				
One		225 (97.8)	210 (97.2)	435 (97.5)
Two		5 (2.2)	6 (2.8)	11 (2.5)
**Gene XPert result**	N =	211	204	415
**At least one positive Gene XPert result**		184 (87.2)	178 (87.25)	362 (87.23)
**Number of culture results recorded on the TB Treatment record**				
One		54 (94.7)	42 (97.7)	96 (96.0)
Two		3 (5.3)	1 (2.3)	4 (4.0)
**Culture result**	N =	41	32	73
**At least one positive culture result**		20 (48.78)	15 (46.88)	35 (47.95)
**Co-morbidities**				
Hypertension		19 (6.93)	11 (4.1)	30 (5.54)
Diabetes		5 (1.84)	4 (1.49)	9 (1.66)
Epilepsy		3 (1.09)	4 (1.49)	7 (1.29)
Mental illness		3 (1.09)	0 (0)	3 (0.55)
Liver disease		1 (0.36)	1 (0.38)	2 (0.37)
Renal insufficiency		1 (0.36)	1 (0.38)	2 (0.37)
Allergies		2 (0.76)	0 (0)	2 (0.38)

For peer review only - http://bmjopen.bmj.com/site/about/guidelines.xhtml

Other		1 (0.36)	1 (0.38)	2 (0.37)
**Total Number of comorbidities**				
0		265 (96.0)	260 (96.7)	525 (96.3)
1		10 (3.6)	8 (3.0)	18 (3.3)
2		0 (0.0)	1 (0.4)	1 (0.2)
5		1 (0.4)	0 (0.0)	1 (0.2)
**HIV status**				
Negative		118 (40.7)	125 (44.2)	243 (42.4)
Positive		163 (56.2)	142 (50.2)	305 (53.2)
Unknown		9 (3.1)	16 (5.7)	25 (4.4)
**HIV positive patients**				
**CD4 Count: mean(SD)**				
**Using Cotrimoxazole**		104 (63.8)	100(67.11)	204 (65.38)
**Using anti-retroviral**		139 (85.28)	118 (79.19)	257 (82.37)

For peer review only

For peer review only - http://bmjopen.bmj.com/site/about/guidelines.xhtml

TABLE 3: Descriptive statistics for smoking history, alcohol history and depression score at baseline by study arm. Frequencies and (percentages) are presented unless otherwise stated.

TABLE 3: Descriptive statistics for smoking history, alcohol history and depression score at baseline and as analysed by study arm. Frequencies and (percentages) are presented unless otherwise stated.			
	Baseline		
	Control	Intervention	Total
	Arm 1	Arm 2	Total
**In the past month, smoked tobacco**			
Not at all	110 (37.8)	92 (32.5)	202 (35.2)
Daily	149 (51.2)	149 (52.7)	298 (51.9)
Less than Daily	32 (11.0)	42 (14.8)	74 (12.9)
**Had a drink in the past 12-months**	208 (71.5)	223 (78.8)	431 (75.1)
**AUDIT Score (males):** mean (SD) [max :19] [min = 8 if drinkers only]	12.27 (3.98)	13.02 (3.78)	12.66 (3.89)
**AUDIT Score (females):** mean (SD) [max :19] [min = 7 if drinkers only]	11.32 (4.02)	10.98 (4.02)	11.15 (4)
**Drinking and Smoking Combined (Constructed)**			
Drinkers Only	110 (37.8)	92 (32.5)	202 (35.2)
Smokers Only	83 (28.5)	60 (21.2)	143 (24.9)
Smokers and Drinkers	98 (33.7)	131 (46.3)	229 (39.9)

For peer review only - http://bmjopen.bmj.com/site/about/guidelines.xhtml

	Arm 1	Arm 2	Total
**Smoking History (current smokers only)**	181	191	372
On the days that you smoke, how soon after you wake up do you have your first cigarette?			
After 60 minutes	30 (16.6)	28 (14.7)	58 (15.6)
31-60 minutes	24 (13.3)	16 (8.4)	40 (10.8)
6- 30 minutes	60 (33.1)	65 (34.0)	125 (33.6)
Within 5 minutes	67 (37.0)	82 (42.9)	149 (40.1)
Duration of smoking in **months**: mean (SD)	212.09 (134.03)	224.93 (127.82)	218.68 (130.86)
Duration of smoking in **months**: median (IQR)	186 (110, 282)	206 (135, 294)	200.5 (123, 287)
Age started smoking in **years**: mean (SD)	19.2 (6.3)	19.3 (6.3)	19.3 (6.3)
Age started smoking in **years**: median (IQR)	18 (15-20)	18 (16-21)	18 (15.5-20.5)
**Form of tobacco used**			
**Manufactured cigarettes (Yes)**	**166 (91.7)**	**179 (93.7)**	**345 (92.7)**
Number of days in the past 7days you smoked: mean (SD)	5.3 (2.65)	5.53 (2.33)	5.42 (2.49)
Average number of cigarettes smoked daily: mean (SD)	6.18 (6.43)	6.48 (8.21)	6.34 (7.39)
**Hand-rolled cigarettes (Yes)**	**14 (7.7)**	**21 (11.0)**	**35 (9.4)**
Number of days in the past 7days you smoked: mean (SD)	4.71 (2.84)	3.71 (2.95)	4.11 (2.91)
Average number of handrolled cigarettes smoked daily: mean (SD)	3.71 (3.97)	3.81 (2.82)	3.77 (3.27)
**Pipe (Yes)**	**4 (2.2)**	**2 (1.0)**	**6 (1.6)**
Number of days in the past 7days you smoked: median (IQR)	1 (0, 2.5)	4.5 (2, 7)	2 (0, 3)
Average number of daily sessions: median (IQR)	1 (0, 3.5)	3 (1, 5)	1.5 (0, 5)
Length of one session (on average) in **minutes**: median (IQR)	90 (60, 107.5)	60 (30, 90)	90 (30, 90)
**Cigars, cheroots or cigarillos (Yes)**	1 (0.55)	0 (0)	1(0.27)

For peer review only - http://bmjopen.bmj.com/site/about/guidelines.xhtml

**Water pipe (Yes)**	3 (1.7)	2 (1.0)	5 (1.3)
Other	10 (5.5)	4 (2.1)	14 (3.8)
**Heaviness of smoking index >= 4**	134(74.03)	158(82.72)	292(78.49)
**Smoking inside your home restrictions**			
Total: Not allowed	96 (53.0)	108 (56.5)	204 (54.8)
Some rules: where/when it is allowed	61 (33.7)	58 (30.4)	119 (32.0)
No rules	24 (13.3)	25 (13.1)	49 (13.2)
**Attempts to quit smoking (current smokers only) (Yes)**			
Ever attempted to quit in the past			
Yes	52 (28.7)	64 (33.5)	116 (31.2)
No	129 (71.3)	127 (66.5)	256 (68.8)
Number of attempts to quit: mean (SD)	2.46 (2.98)	2.64 (1.62)	2.56 (2.32)
Time elapsed since attempt to quit last time in months: mean (SD)	36.65 (94.62)	25.13 (37.91)	30.29 (69.22)
Longest duration abstinent in previous quit attempts: mean (SD)	6.15 (13.48)	4.22 (8.9)	5.09 (11.18)
Likelihood to TRY TO QUIT smoking completely and permanently in the next three months			
definitely will not	6 (3.3)	5 (2.6)	11 (3.0)
probably will not	10 (5.5)	12 (6.3)	22 (5.9)
probably will	104 (57.5)	109 (57.1)	213 (57.3)
definitely will	61 (33.7)	65 (34.0)	126 (33.9)
	Control	Intervention	

For peer review only - http://bmjopen.bmj.com/site/about/guidelines.xhtml

	Arm 1	Arm 2	Total
Likelihood that I WILL QUIT smoking completely and permanently in the next three months			
definitely will not	6 (3.3)	5 (2.6)	11 (3.0)
probably will not	11 (6.1)	13 (6.8)	24 (6.5)
probably will	103 (56.9)	104 (54.5)	207 (55.6)
definitely will	61 (33.7)	69 (36.1)	130 (34.9)
**Ever used any methods to help you stop smoking tobacco in the past 3 months?** (Yes)	23 (12.7)	16 (8.4)	39 (10.5)
**Out of your pocket spend (in Rands) on methods to help you stop smoking in the past 3 months: mean (SD)**	134.87 (237.58)	139.19 (155.07)	136.64 (205.36)
**Average spend per week on cigarettes over the past 3 months: mean (SD)**	168.77 (178.15)	180.29 (185.27)	174.58 (181.61)
**Smokeless tobacco use (all participants)**			
**In the past month, have you used smokeless tobacco (Snuff) on a daily basis**			
Not at all	275 (94.5)	275 (97.2)	550 (95.8)
Daily	12 (4.1)	6 (2.1)	18 (3.1)
Less than Daily	4 (1.4)	2 (0.7)	6 (1.0)
Duration of using ST in months: mean(SD)	113.69 (112.7)	152 (91.16)	126.46 (105.61)
Age started using ST in **years**: mean (SD)	27.56 (10.57)	25.88 (13.43)	27 (11.33)
**Form of ST used (for SLT users)**			
Snuff (by mouth)	2 (12.5)	0 (0.0)	2 (8.3)
Snuff (by nose)	11 (68.8)	5 (62.5)	16 (66.7)
Chewing tobacco leaves	0(0)	0(0)	0(0)
Other	1 (6.3)	0 (0.0)	1 (4.2)
	Arm 1	Arm 2	Total

For peer review only - http://bmjopen.bmj.com/site/about/guidelines.xhtml

	Control	Intervention	
**Tobacco smoke exposure:** number of days in the past 30 days, you were in a place where someone smoked close to you (all participants?)			
**Home**			
7 days or less	233 (80.1)	229 (80.9)	462 (80.5)
More than 7 days	58 (19.9)	54 (19.1)	112 (19.5)
**Workplace** (Missing 315; probably Not Applicable)			
7 days or less	84 (65.6)	89 (67.9)	173 (66.8)
More than 7 days	44 (34.4)	42 (32.1)	86 (33.2)
**Cafes/restaurants**			
7 days or less	230 (79.0)	234 (82.7)	464 (80.8)
More than 7 days	61 (21.0)	49 (17.3)	110 (19.2)
**Shebeens, bars or clubs**			
7 days or less	219 (75.3)	213 (75.3)	432 (75.3)
More than 7 days	72 (24.7)	70 (24.7)	142 (24.7)
**Bus/train/taxi/ vehicle**			
7 days or less	229 (78.7)	219 (77.4)	448 (78.0)
More than 7 days	62 (21.3)	64 (22.6)	126 (22.0)
**Shops/shopping mall**			
7 days or less	235 (80.8)	226 (79.9)	461 (80.3)
More than 7 days	56 (19.2)	57 (20.1)	113 (19.7)
	Arm 1	Arm 2	Total

For peer review only - http://bmjopen.bmj.com/site/about/guidelines.xhtml

**Had a drink in the past 12-months**	208 (71.5)	223 (78.8)	431 (75.1)
**Help to stop drinking (drinkers only)**			
Ever used any methods to stop drinking alcohol in the past 3 months	21 (11.2)	22 (10.7)	43 (10.9)
Average spend in Rands per week on alcohol over the past 3 months: mean (SD)	363.76 (531.56)	337.03 (387.94)	349.79 (461.66)
**Depression: CESD 10** how often you felt or behaved this way during the past week			
I was bothered by things that usually don't bother me.			
Rarely or None of the Time (Less than 1 day)	180 (61.9)	159 (56.2)	339 (59.1)
Some or a Little of the Time (1-2 days)	71 (24.4)	78 (27.6)	149 (26.0)
Occasionally or a Moderate Amount of the Time (3-4 days)	24 (8.2)	31 (11.0)	55 (9.6)
Most or All of the Time (5-7 days)	16 (5.5)	15 (5.3)	31 (5.4)
I had trouble keeping my mind on what I was doing.			
Rarely or None of the Time (Less than 1 day)	150 (51.5)	136 (48.1)	286 (49.8)
Some or a Little of the Time (1-2 days)	93 (32.0)	85 (30.0)	178 (31.0)
Occasionally or a Moderate Amount of the Time (3-4 days)	31 (10.7)	38 (13.4)	69 (12.0)
Most or All of the Time (5-7 days)	17 (5.8)	24 (8.5)	41 (7.1)
I felt depressed.			
Rarely or None of the Time (Less than 1 day)	152 (52.2)	171 (60.4)	323 (56.3)
Some or a Little of the Time (1-2 days)	94 (32.3)	70 (24.7)	164 (28.6)
Occasionally or a Moderate Amount of the Time (3-4 days)	37 (12.7)	30 (10.6)	67 (11.7)
Most or All of the Time (5-7 days)	8 (2.7)	12 (4.2)	20 (3.5)
I felt that everything I did was an effort.			

For peer review only - http://bmjopen.bmj.com/site/about/guidelines.xhtml

Rarely or None of the Time (Less than 1 day)	140 (48.1)	117 (41.3)	257 (44.8)
Some or a Little of the Time (1-2 days)	77 (26.5)	87 (30.7)	164 (28.6)
Occasionally or a Moderate Amount of the Time (3-4 days)	38 (13.1)	43 (15.2)	81 (14.1)
Most or All of the Time (5-7 days)	36 (12.4)	36 (12.7)	72 (12.5)
I felt hopeful about the future.			
Rarely or None of the Time (Less than 1 day)	85 (29.2)	84 (29.7)	169 (29.4)
Some or a Little of the Time (1-2 days)	78 (26.8)	70 (24.7)	148 (25.8)
Occasionally or a Moderate Amount of the Time (3-4 days)	53 (18.2)	56 (19.8)	109 (19.0)
Most or All of the Time (5-7 days)	75 (25.8)	73 (25.8)	148 (25.8)
I felt fearful.			
Rarely or None of the Time (Less than 1 day)	175 (60.1)	159 (56.2)	334 (58.2)
Some or a Little of the Time (1-2 days)	82 (28.2)	79 (27.9)	161 (28.0)
Occasionally or a Moderate Amount of the Time (3-4 days)	25 (8.6)	32 (11.3)	57 (9.9)
Most or All of the Time (5-7 days)	9 (3.1)	13 (4.6)	22 (3.8)
My sleep was restless.			
Rarely or None of the Time (Less than 1 day)	142 (48.8)	135 (47.7)	277 (48.3)
Some or a Little of the Time (1-2 days)	95 (32.6)	89 (31.4)	184 (32.1)
Occasionally or a Moderate Amount of the Time (3-4 days)	31 (10.7)	33 (11.7)	64 (11.1)
Most or All of the Time (5-7 days)	23 (7.9)	26 (9.2)	49 (8.5)
I was happy.			
Rarely or None of the Time (Less than 1 day)	85 (29.2)	74 (26.1)	159 (27.7)
Some or a Little of the Time (1-2 days)	70 (24.1)	65 (23.0)	135 (23.5)
Occasionally or a Moderate Amount of the Time (3-4 days)	73 (25.1)	86 (30.4)	159 (27.7)
Most or All of the Time (5-7 days)	63 (21.6)	58 (20.5)	121 (21.1)
I felt lonely.			

For peer review only - http://bmjopen.bmj.com/site/about/guidelines.xhtml

Rarely or None of the Time (Less than 1 day)	158 (54.3)	161 (56.9)	319 (55.6)
Some or a Little of the Time (1-2 days)	89 (30.6)	83 (29.3)	172 (30.0)
Occasionally or a Moderate Amount of the Time (3-4 days)	23 (7.9)	26 (9.2)	49 (8.5)
Most or All of the Time (5-7 days)	21 (7.2)	13 (4.6)	34 (5.9)
I could not get “going”.			
Rarely or None of the Time (Less than 1 day)	168 (57.7)	174 (61.5)	342 (59.6)
Some or a Little of the Time (1-2 days)	78 (26.8)	69 (24.4)	147 (25.6)
Occasionally or a Moderate Amount of the Time (3-4 days)	35 (12.0)	29 (10.2)	64 (11.1)
Most or All of the Time (5-7 days)	10 (3.4)	11 (3.9)	21 (3.7)
Total CESD 10: mean (SD)	8.44 (4.38)	8.74 (4.8)	8.59 (4.59)

For peer review only - http://bmjopen.bmj.com/site/about/guidelines.xhtml

Table 4: Descriptive statistics for primary and secondary outcomes by study arm at baseline (where available), 3 month (where available) and 6 month. Frequencies and (percentages) are presented unless otherwise stated

For peer review only

TABLE 4: DESCRIPTIVE STATISTICS FOR PRIMARY AND SECONDARY OUTCOMES BY STUDY ARM AT BASELINE (WHERE AVAILABLE), 3 MONTH (WHERE AVAILABLE) AND 6 MONTH. FREQUENCIES AND (PERCENTAGES) ARE PRESENTED UNLESS OTHERWISE STATED.

	Baseline			Follow-up 3-month			Follow-up 6-month		
	Control	Intervention	Total	Control	Interventio n	Total	Control	Intervention	Total
	Arm1	Arm2	Total	Arm1	Arm2	Total	Arm1	Arm2	Total
**TB treatment status detailed**									
Cured							108 (37.11)	105 (37.1)	213 (37.11)
Treatment completed							96 (33.0)	87 (30.74)	183 (31.88)
Treatment default							15 (5.2)	29 (10.25)	44 (7.67)
Treatment failure							5 (1.7)	2 (0.7)	7 (1.2)
Acquired drug resistance							1 (0.34)	4 (1.41)	5 (0.87)
Died							11 (3.78)	15 (5.30)	26 (4.53)
Transfer out							8 (2.75)	11 (3.89)	19 (3.31)
Unknown							42 (14.43)	25 (8.83)	67 (11.67)
Missing							5 (1.72)	5 (1.77)	10 (1.74)
**TB treatment status binary** (Primary outcome**)									
Not Successful							87 (29.9)	91 (32.16)	178 (31.01)
Successful							204 (70.10)	192 (67.84)	396 (68.99)
**At least one positive smear result**	85 (53.46)	96 (61.15)	181 (57.28)						
**At least one positive Gene XPert result**	184 (87.20)	178 (87.25)	362 (87.23)						
**At least one positive culture result**	11 (57.89)	10 (66.67)	21 (61.76)						

For peer review only - http://bmjopen.bmj.com/site/about/guidelines.xhtml

**Sputum smear, Gene XPert, or culture result**									
Negative	29 (12.45)	32 (14.16)	61 (13.29)						
Positive	204 (87.55)	194 (85.84)	398 (86.71)						
**Conversion from positive to negative*** **									
Yes							83(39.9)	85(43.59)	168(41.69)
No							125(60.1)	110(56.41)	235(58.31)
**Continuous smoking abstinence among cigarette smokers at baseline**									
Yes				27(16.27)	20(11.17)	47(13.62)	12(7.23)	10(5.59)	22(6.38)
No				139(83.73)	159(88.83)	298(86.38)	154(92.77)	169(94.41)	323(93.62)
**Harmful or hazardous drinking[1]**									
**Had a drink in the past 12-months**	208 (71.5)	223 (78.8)	431 (75.1)						
AUDIT score: mean (SD)	12.03 (4)	12.53 (3.93)	12.29 (3.96)						
**Harmful or hazardous drinkers out of those who had a drink in the past 12-months at baseline (%)**	188(90.38)	206(92.38)	394(91.42)	141	130	271	112	127	239
**AUDIT Score (males):** mean (SD) [max :19; min = 8]*!	13.14 (3.31)	13.61 (3.29)	13.39 (3.31)	8.20(6.08)	9.08(4.97)	8.63(5.58)	9.21(6.58)	8.24(5.41)	8.69(5.99)
**AUDIT Score (females):** mean (SD) [max :19; min = 7]*!	11.73 (3.52)	11.55 (3.6)	11.64 (3.54)	8.5(6.52)	8.15(6.44)	8.33(6.44)	7.67(6.84)	9.97(6.79)	8.89(6.86)
AUDIT score: mean (SD)	12.76 (3.42)	13.12 (3.47)	12.94 (3.45)	8.28(6.18)	8.84(5.38)	8.55(5.81)	8.79(6.66)	8.70(5.83)	8.74(6.22)
Difference from baseline				-4.61 (6.26)	-4.07(5.33)	-4.35(5.83)	-4.25(6.56)	-4.17(6.61)	-4.21(6.57)

For peer review only - http://bmjopen.bmj.com/site/about/guidelines.xhtml

**Taking ART medication among** HIV Positive patients	[163 (56.2)]	[142 (50.2)]	[305 (53.2)]	[122]	[83]	[205]	[100]	[83]	[183]
Yes	139 (85.28)	115 (80.99)	254 (83.28)	91 (74.6)	58 (69.9)	149 (72.7)	80 (80.0)	74 (89.2)	154 (84.2)
**ART medication adherence**									
Optimal adherence				101(99.02)	64(98.46)	165(98.8)	75(97.4)	64(96.97)	139(97.2)
Suboptimal adherence				1(0.98)	1(1.54)	2(1.2)	2(2.6)	2(3.03)	4(2.8)
**TB medication adherence**									
Optimal adherence				181(92.35)	138(90.79)	319(91.67)	61(89.71)	59(90.77)	120(90.23)
Suboptimal adherence				15(7.65)	14(9.21)	29(8.33)	7(10.29)	6(9.23)	13(9.77)

[1] hazardous/harmful drinkers who are not alcohol dependent= AUDIT score ≥ 8 for men or ≥ 7 for women but < 20

**Primary Outcome: in the published protocol paper "This is a binary variable defined as either successful treatment (cured or treatment completed) or failed treatment, death, acquired drug resistance, loss to follow-up or ‘default’, or not outcome evaluated.

*** Conversion from positive to negative: this was based on having a cured treatment outcome among those who were positive at baseline.

*! Important distinction at baseline for eligibility purposes.

For peer review only

TABLE 5: Number and type of adverse events at month 2, 3, and 6 by centre and study arm (see other document)

TABLE 5: Number and type of adverse events at month 2, 3, and 6 by centre and study arm							
Centre		Month 2		Month 3		Month 6	
	TYPE	Intervention	Control	Intervention	Control	Intervention	Control

For peer review only

For peer review only - http://bmjopen.bmj.com/site/about/guidelines.xhtml

TABLE 6: Regression analysis results for the primary and secondary outcomes at 6 months. Estimates presented with corresponding 95% CI. Crude and adjusted estimates are provided.

TABLE 6: Regression analysis results for the primary and secondary outcomes at 6 months. Estimates presented with corresponding 95% CI. Crude and adjusted estimates are provided.

	Crude Odds Ratio (95% CI)*	P Value*	Adjusted Odds Ratio (95% CI)*	P Value*	
**Primary outcome**					
TB treatment status: Successful (**Ref: Not successful**)	0.9 (0.64,1.27)	0.548	0.86 (0.60,1.24)	0.421	
**Secondary outcomes**					
Sputum smear or culture result: converted from positive to negative (Ref: Not converted)	1.16 (0.83,1.63)	0.374	1.07‡ (0.76,1.51)	0.684	
Six-month continuous smoking abstinence among cigarette smokers at baseline (Ref: No)	0.76 (0.35,1.64)	0.482			
Taking ART medication among HIV positive patients[!!]	2.05 (0.80,5.27)	0.136			
TB medication adherence (Reference: Optimal)	0.89 (0.26,3.07)	0.849			
ART medication adherence (Reference: Optimal)	1.17 (0.14,9.94)	0.884			

For peer review only

	Study arm regression coefficient		Study arm regression coefficient		
**AUDIT for those who were harmful or hazardous drinkers at baseline**:**	-0.04 (-2,1.91)	0.966	0.02[!] (-1.55,1.6)	0.976	

* analyses accounted for potential clustering by centre.
† Number of participants whose outcome was treatment successful among the total number in the group.
‡ Adjusted for district, sex, and smoking/drinking status and HIV status at baseline
**Controlling for the AUDIT baseline values.
! Controlling for the AUDIT baseline values and adjusted for district, sex, and smoking/drinking status and HIV status at baseline
!! Adjusting for art status at baseline

For peer review only

For peer review only - http://bmjopen.bmj.com/site/about/guidelines.xhtml

TABLE 7: Regression analysis results for secondary outcomes measured at 3-months. Estimates presented with corresponding 95% CI. Crude and adjusted estimates are provided.

TABLE 7: Regression analysis results for secondary outcomes measured at 3-months. Estimates presented with corresponding 95% CI. Crude and adjusted estimates are provided.

	Crude Odds Ratio (95% CI)	P Value	Adjusted Odds Ratio (95% CI)	P Value	
**Secondary outcome**					
3-months continuous smoking abstinence among cigarette smokers at baseline	0.65 (0.37,1.14)	0.135			
Taking ART medication among HIV positive patients[!!]	0.79 (0.38,1.65)	0.53	0.74‡ (0.35,1.58)	0.443	
TB medication adherence (Reference: Optimal)	1.22 (0.52,2.87)	0.641	[redacted]		
ART medication adherence (Reference: Optimal)	1.58 (0.10,26.12)	0.750	[redacted]		
	Study arm regression coefficient		Adjusted estimates		
AUDIT for those who were harmful or hazardous drinkers at baseline**:	0.55 (-1.01,2.11)	0.474	0.74 (-0.62,2.1)	0.273	

* analyses accounted for clustering.
‡ Adjusted for district, sex, and smoking/drinking status and HIV status at baseline
**Controlling for the AUDIT baseline values.
! Controlling for the AUDIT baseline values and adjusted for district, sex, and smoking/drinking status and HIV status at baseline
!! Adjusting for art status at baseline

For peer review only

Additional tables
SMS-fidelity

**Variable**	**Intervention (N= ….) (=no. of participants allocated to intervention group, for example 245)**	**Control (N=…) (=no. participants allocated to control group, for example 248)**
No. of participants who received ALL due IMB messages INDEPENDENT OF WHETHER they completed MI 1 (i.o.w this is ITT analysis)	For example 120/245 (49 %)	For example 2/248 (…%)
Completion of first MI and initiation of SMS-sequence		
Completed first MI	n/N (%) (=No. who completed first MI in intervention arm/ no. participants allocated to intervention arm [%] For example 170/245 [69%])	n/N (%) (=No. who completed first MI in control/ no. participants allocated to control [%] For example 2/248)
No. of participants who received ALL due messages after receipt of MI1 and SMS sequence was generated (this is a type of Per Protocol analysis:	For example 120/170 (71%)	2/2

For peer review only - http://bmjopen.bmj.com/site/about/guidelines.xhtml

denominator limited to those who had MI1)				
SMS delivery for participants for whom the SMS-sequence was initiated (after receipt of first MI)				
	Mean (SD)	Median (IQR) Range	Mean (SD)	Median (IQR)
Average no. adherence messages received per participant (n=...) (n = the no. of participants who completed first MI, in this example 170)	For example 8 (3.4)	9 (7-10) Range 0-10	Expected to be 0 or close to it	Expected to be 0 or close to it
Average no. tobacco-related messages received (n=...) (n=no of participants who completed first MI AND were current tobacco users at baseline, for example 90)	For example 7 (3.4)	7 (6-7) Range 0-7	Expected to be 0 or close to it	Expected to be 0 or close to it
Average no. alcohol related messages received (n=...) (n=no. participants who completed first MI	For example 7 (3.4)	7 (6-7) Range 0-7	Expected to be 0 or close to it	Expected to be 0 or close to it

For peer review only - http://bmjopen.bmj.com/site/about/guidelines.xhtml

AND were drinkers at baseline=....for example 100)				
Average no. IMB messages received (n= total no who completed first MI)	For example 16 (4.5)	15 (10-20)<br><br>Range 0-24	Expected to be 0 or close to it	Expected to be 0 or close to it

Other variables not yet analysed:

CESD at 3 and 6-month FU
Other smoking related questions at 3 and 6 month (quit intentions etc) but also SLT use at 3 and 6 month. (important because participants may have switched from tobacco smoking to SLT)
Other non-HE questions not yet analysed

For peer review only - http://bmjopen.bmj.com/site/about/guidelines.xhtml

13. Additional descriptive Statistics

TABLE 8: Additional Descriptive statistics for characteristics at baseline by study arm. Frequencies and (percentages) are presented unless otherwise stated

		Baseline		
	N1/N2	Intervention	Control	Total
		Arm 1	Arm 2	
TYPE OF MINE WORK	46/45			91
Coal		7 (15.2)	3 (6.7)	10 (11.0)
Diamond		3 (6.5)	1 (2.2)	4 (4.4)
Gold		13 (28.3)	17 (37.8)	30 (33.0)
Platinum and palladium		24 (52.2)	17 (37.8)	41 (45.1)
Chromium		11 (23.9)	12 (26.7)	23 (25.3)
Uranium		1 (2.2)	1 (2.2)	2 (2.2)
Manganese		0(0)	0(0)	0(0)
Other		0(0)	0(0)	0(0)
Total Types of Mines worked in				
1	73			
2	12			
3	3			
4	1			

For peer review only - http://bmjopen.bmj.com/site/about/guidelines.xhtml

Exact Distribution for number smoked in the past seven days				
0		24 (14.5)	16 (8.9)	40 (11.6)
1		4 (2.4)	4 (2.2)	8 (2.3)
2		4 (2.4)	3 (1.7)	7 (2.0)
3		5 (3.0)	13 (7.3)	18 (5.2)
4		13 (7.8)	8 (4.5)	21 (6.1)
5		5 (3.0)	17 (9.5)	22 (6.4)
6		1 (0.6)	3 (1.7)	4 (1.2)
7		110 (66.3)	115 (64.2)	225 (65.2)
new_a_positive_TB				
0		29 (11.98)	33 (14.35)	62 (13.14)
1		122 (50.41)	104 (45.22)	226 (47.88)
2		80 (33.06)	81 (35.22)	161 (34.11)
3		11 (4.55)	12 (5.22)	23 (4.87)

For peer review only - http://bmjopen.bmj.com/site/about/guidelines.xhtml

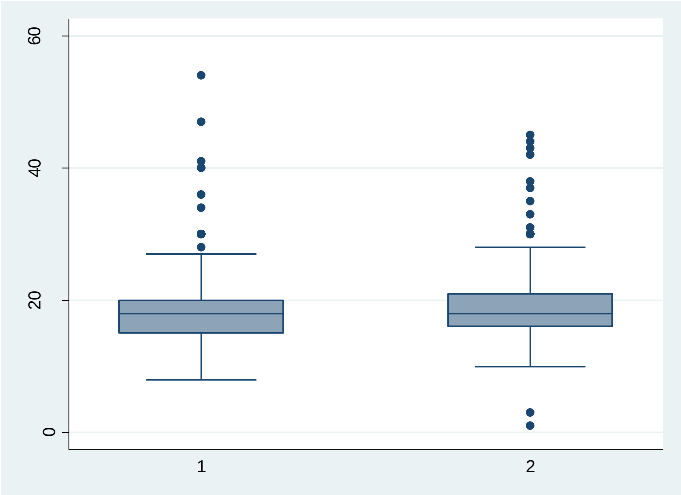

Age started smoking

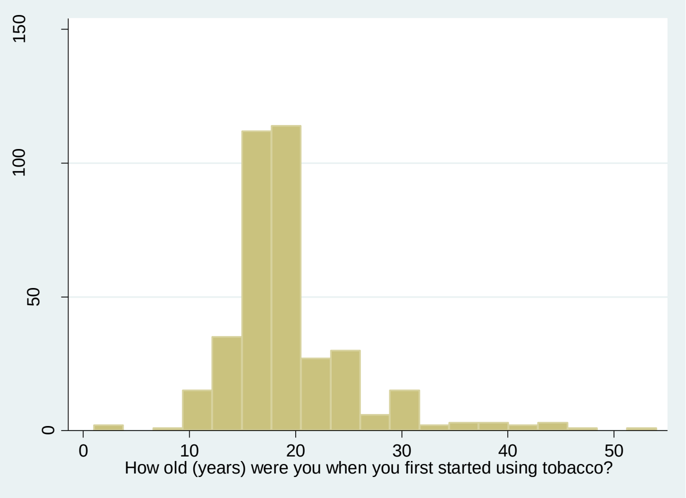

For peer review only - http://bmjopen.bmj.com/site/about/guidelines.xhtml

CONSORT 2010 checklist of information to include when reporting a randomised trial*

Section/Topic	Item No	Checklist item	Reported on page No
**Title and abstract**			
	1a	Identification as a randomised trial in the title	1
	1b	Structured summary of trial design, methods, results, and conclusions (for specific guidance see CONSORT for abstracts)	2
**Introduction**			
Background and objectives	2a	Scientific background and explanation of rationale	4-5
	2b	Specific objectives or hypotheses	5-7
**Methods**			
Trial design	3a	Description of trial design (such as parallel, factorial) including allocation ratio	5-6
	3b	Important changes to methods after trial commencement (such as eligibility criteria), with reasons	NA
Participants	4a	Eligibility criteria for participants	5
	4b	Settings and locations where the data were collected	5
Interventions	5	The interventions for each group with sufficient details to allow replication, including how and when they were actually administered	6
Outcomes	6a	Completely defined pre-specified primary and secondary outcome measures, including how and when they were assessed	7-8
	6b	Any changes to trial outcomes after the trial commenced, with reasons	NA
Sample size	7a	How sample size was determined	8
	7b	When applicable, explanation of any interim analyses and stopping guidelines	NA
Randomisation:			
Sequence generation	8a	Method used to generate the random allocation sequence	6
	8b	Type of randomisation; details of any restriction (such as blocking and block size)	6
Allocation concealment mechanism	9	Mechanism used to implement the random allocation sequence (such as sequentially numbered containers), describing any steps taken to conceal the sequence until interventions were assigned	6
Implementation	10	Who generated the random allocation sequence, who enrolled participants, and who assigned participants to interventions	6
Blinding	11a	If done, who was blinded after assignment to interventions (for example, participants, care providers, those	6

For peer review only - http://bmjopen.bmj.com/site/about/guidelines.xhtml

		assessing outcomes) and how	
	11b	If relevant, description of the similarity of interventions	NA
Statistical methods	12a	Statistical methods used to compare groups for primary and secondary outcomes	9
	12b	Methods for additional analyses, such as subgroup analyses and adjusted analyses	9
**Results**			
Participant flow (a diagram is strongly recommended)	13a	For each group, the numbers of participants who were randomly assigned, received intended treatment, and were analysed for the primary outcome	10, Figure 1
	13b	For each group, losses and exclusions after randomisation, together with reasons	10, Fig 1
Recruitment	14a	Dates defining the periods of recruitment and follow-up	5-6
	14b	Why the trial ended or was stopped	10,21
Baseline data	15	A table showing baseline demographic and clinical characteristics for each group	Table 1 and 2, Supp Table 1
Numbers analysed	16	For each group, number of participants (denominator) included in each analysis and whether the analysis was by original assigned groups	Fig 1, Table 3
Outcomes and estimation	17a	For each primary and secondary outcome, results for each group, and the estimated effect size and its precision (such as 95% confidence interval)	Table 3,4,5
	17b	For binary outcomes, presentation of both absolute and relative effect sizes is recommended	Only relative effect
Ancillary analyses	18	Results of any other analyses performed, including subgroup analyses and adjusted analyses, distinguishing pre-specified from exploratory	Table 4, 5
Harms	19	All important harms or unintended effects in each group (for specific guidance see CONSORT for harms)	No harms reported
**Discussion**			
Limitations	20	Trial limitations, addressing sources of potential bias, imprecision, and, if relevant, multiplicity of analyses	21-22
Generalisability	21	Generalisability (external validity, applicability) of the trial findings	NA, no effect
Interpretation	22	Interpretation consistent with results, balancing benefits and harms, and considering other relevant evidence	21
**Other information**			
Registration	23	Registration number and name of trial registry	2
Protocol	24	Where the full trial protocol can be accessed, if available	Published paper referenced

For peer review only

For peer review only - http://bmjopen.bmj.com/site/about/guidelines.xhtml

Funding	25	Sources of funding and other support (such as supply of drugs), role of funders	23

*We strongly recommend reading this statement in conjunction with the CONSORT 2010 Explanation and Elaboration for important clarifications on all the items. If relevant, we also recommend reading CONSORT extensions for cluster randomised trials, non-inferiority and equivalence trials, non-pharmacological treatments, herbal interventions, and pragmatic trials. Additional extensions are forthcoming: for those and for up to date references relevant to this checklist, see www.consort-statement.org.

For peer review only

BMJ Open

Effect of a brief motivational interview and text message intervention targeting tobacco smoking, alcohol use, and medication adherence to improve tuberculosis treatment outcomes in adult patients with tuberculosis: a multicentre, randomised controlled trial of the ProLife programme in South Africa

Journal:	*BMJ Open*
Manuscript ID	bmjopen-2021-056496.R2
Article Type:	Original research
Date Submitted by the Author:	17-Dec-2021
Complete List of Authors:	Louwagie, Goedele; Sefako Makgatho Health Sciences University, Research, Postgraduate Studies and Innovation; University of Pretoria, School of Health Systems and Public Health<br>Kanaan, Mona; University of York, Department of Health Sciences<br>Morojele, Neo; University of Johannesburg, Department of Psychology; Medical Research Council of South Africa, Alcohol, Tobacco and Other Drug Research Unit<br>Van Zyl, Andre; Sefako Makgatho Health Sciences University<br>Moriarty, Andrew; University of York, Hull York Medical School<br>Li, Jinshuo; University of York, Department of Health Sciences<br>Siddiqi, Kamran; University of York, Department of Health Sciences and the Hull York Medical School<br>Turner, Astrid; University of Pretoria, School of Health Systems and Public Health<br>Mdege, Noreen; University of York, Department of Health Sciences<br>Omole, Olufemi; University of the Witwatersrand, Department of Family Medicine<br>Tumbo, John; Sefako Makgatho Health Sciences University, Department of Family Medicine<br>Bachmann, Max; University of East Anglia, Norwich Medical School<br>Parrott, Steve; University of York, Department of Health Sciences<br>Ayo-Yusuf, Olalekan; Sefako Makgatho Health Sciences University, Africa Centre for Tobacco Industry Monitoring & Policy Research; University of Pretoria, School of Health Systems and Public Health
**Primary Subject Heading**:	Public health
Secondary Subject Heading:	Health economics, Infectious diseases, HIV/AIDS, Smoking and tobacco, Health services research
Keywords:	Tuberculosis < INFECTIOUS DISEASES, Public health < INFECTIOUS DISEASES, HIV & AIDS < INFECTIOUS DISEASES, Substance misuse < PSYCHIATRY, Health economics < HEALTH SERVICES ADMINISTRATION & MANAGEMENT, Organisation of health services < HEALTH SERVICES ADMINISTRATION & MANAGEMENT

SCHOLARONE™
Manuscripts

I, the Submitting Author has the right to grant and does grant on behalf of all authors of the Work (as defined in the below author licence), an exclusive licence and/or a non-exclusive licence for contributions from authors who are: i) UK Crown employees; ii) where BMJ has agreed a CC-BY licence shall apply, and/or iii) in accordance with the terms applicable for US Federal Government officers or employees acting as part of their official duties; on a worldwide, perpetual, irrevocable, royalty-free basis to BMJ Publishing Group Ltd ("BMJ") its licensees and where the relevant Journal is co-owned by BMJ to the co-owners of the Journal, to publish the Work in this journal and any other BMJ products and to exploit all rights, as set out in our licence.

The Submitting Author accepts and understands that any supply made under these terms is made by BMJ to the Submitting Author unless you are acting as an employee on behalf of your employer or a postgraduate student of an affiliated institution which is paying any applicable article publishing charge ("APC") for Open Access articles. Where the Submitting Author wishes to make the Work available on an Open Access basis (and intends to pay the relevant APC), the terms of reuse of such Open Access shall be governed by a Creative Commons licence – details of these licences and which Creative Commons licence will apply to this Work are set out in our licence referred to above.

Other than as permitted in any relevant BMJ Author's Self Archiving Policies, I confirm this Work has not been accepted for publication elsewhere, is not being considered for publication elsewhere and does not duplicate material already published. I confirm all authors consent to publication of this Work and authorise the granting of this licence.

For peer review only

Effect of a brief motivational interview and text message intervention targeting tobacco smoking, alcohol use, and medication adherence to improve tuberculosis treatment outcomes in adult patients with tuberculosis: a multicentre, randomised controlled trial of the ProLife programme in South Africa

Goedele Maria Louwagie[1, 2], Mona Kanaan[3], Neo Keitumetse Morojele[4, 5], André van Zyl[1], Andrew Stephen Moriarty[3], Jinshuo Li[3], Kamran Siddiqi[3], Astrid Turner[2], Noreen Dadirai Mdege[3], Olufemi Babatunde Omole[6], John Tumbo[1], Max Oscar Bachmann[7], Steve Parrott[3], Olalekan Ayo-Yusuf[1, 2]

Correspondence to Goedele Maria Louwagie; goedele.louwagie@up.ac.za

[1]Goedele Maria Louwagie, Sefako Makgatho Health Sciences University and [2]School of Health Systems and Public Health, University of Pretoria , RSA; goedele.louwagie@up.ac.za

[3]Mona Kanaan, Department of Health Sciences, University of York, UK; mona.kanaan@york.ac.uk

[4]Neo Keitumetse Morojele, Department of Psychology, University of Johannesburg and [5]Alcohol, Tobacco and Other Drug Research Unit, South African Medical Research Council, RSA; nmorojele@uj.ac.za

[1]André van Zyl, Sefako Makgatho Health Sciences University, RSA; drevanzyl@gmail.com

[3]Andrew Stephen Moriarty, Department of Health Sciences and the Hull York Medical School, University of York, UK; andrew.moriarty@york.ac.uk

[3]Jinshuo Li, Department of Health Sciences, University of York, UK; jinshuo.li@york.ac.uk

[3]Kamran Siddiqi, Department of Health Sciences and the Hull York Medical School, University of York, UK; kamran.siddiqi@york.ac.uk

[2]Astrid Turner, School of Health Systems and Public Health, University of Pretoria, RSA; astrid.turner@up.ac.za

[3]Noreen Dadirai Mdege, Department of Health Sciences, University of York, UK; noreen.mdege@york.ac.uk

[6]Olufemi Babatunde Omole, Department of Family Medicine, University of Witwatersrand, RSA; Olufemi.Omole@wits.ac.za

[1]John Tumbo, Department of Family Medicine, Sefako Makgatho Health Sciences University, RSA; tumbo@lantic.net

[7]Max Oscar Bachmann, Norwich Medical School, University of East Anglia, UK; m.bachmann@uea.ac.uk

For peer review only

For peer review only - http://bmjopen.bmj.com/site/about/guidelines.xhtml

[3]Steve Parrott, Department of Health Sciences, University of York, UK; steve.parrott@york.ac.uk

[1]Olalekan Ayo-Yusuf, Africa Centre for Tobacco Industry Monitoring & Policy Research, Sefako Makgatho Health Sciences University, and [2]School of Health Systems and Public Health, University of Pretoria, RSA; lekan.ayo-yusuf@smu.ac.za

ABSTRACT

Objectives: To investigate the effectiveness of a complex behavioural intervention, ProLife, on tuberculosis (TB) treatment success, medication adherence, alcohol use and tobacco smoking.

Design: Multicentre, individual, randomised controlled trial where participants were assigned (1:1) to the ProLife intervention or usual care.

Setting: 27 primary care clinics in South Africa.

Participants: 574 adults starting treatment for drug-sensitive pulmonary TB who smoked tobacco or reported harmful/hazardous alcohol use.

Interventions: The intervention, delivered by lay health workers (LHWs), consisted of 3 brief motivational interviewing (MI) sessions, augmented with Short Message Service (SMS) messages, targeting medication adherence, alcohol use and tobacco smoking.

Outcome measures: The primary outcome was successful versus unsuccessful TB treatment at 6 to 9 months, from TB records. Secondary outcomes were biochemically confirmed sustained smoking cessation, reduction in the Alcohol Use Disorder Identification Test (AUDIT) score, improved TB and antiretroviral treatment (ART) adherence and ART initiation, each measured at 3 and 6 months by questionnaires; and cure rates in patients who had bacteriology-confirmed TB at baseline, from TB records.

Results: Between 15 November 2018 and 31 August 2019, 574 participants were randomised to receive either the intervention (n=283) or usual care (n=291). TB treatment success rates did not differ significantly between intervention (67.8%) and control (70.1%; OR=0.9 (95% CI: 0.64,1.27)). There was no evidence of an effect at 3- and 6-months respectively on continuous smoking abstinence (OR=0.65 (95% CI: 0.37,1.14); OR=0.76 (95% CI: 0.35,1.63)), TB medication adherence (OR=1.22 (95%CI: 0.52,2.87); OR=0.89 (95%CI: 0.26,3.07)), taking ART (OR=0.79 (0.38,1.65), OR=2.05 (0.80,5.27)) or AUDIT scores (mean score difference 0.55 (95% CI: -1.01,2.11); -0.04 (95% CI: -2,1.91); and adjusting for baseline values. Cure rates were not significantly higher (OR=1.16 (0.83,1.63)).

Conclusions: Simultaneous targeting of multiple health risk behaviours with MI and SMS using LHWs may not be an effective approach to improve TB outcomes.

For peer review only

For peer review only - http://bmjopen.bmj.com/site/about/guidelines.xhtml

Trial registration: ISRCTN62728852, Registered on 13th April 2018.

Key words: Tuberculosis, smoking, alcohol, motivational interviewing, mHealth, anti-retroviral therapy, adherence

STRENGTHS AND LIMITATIONS OF THIS STUDY

- The use of motivational interviewing combined with Short Text Messaging to address the effect of multiple risk behaviours (smoking, drinking and poor adherence) on tuberculosis treatment outcomes is a novel and much needed intervention.
- Our study design was strong: this was a multi-site individually randomised controlled trial with a large sample size and a high follow up rate for the primary outcome.
- We used validated measurement tools; furthermore, data analysis and primary outcome assessment were blinded, thereby limiting measurement bias.
- However, the study was underpowered for secondary outcomes, and low intervention uptake may have diluted any potential intervention effects.

For peer review only

For peer review only - http://bmjopen.bmj.com/site/about/guidelines.xhtml

INTRODUCTION

Tuberculosis (TB) is among the most common chronic infectious diseases in the world today. In 2019, 1.4 million deaths worldwide were attributed to TB, and the majority of these occurred in low-income and middle-income countries (LMICs).[1] South Africa not only has one of the highest TB burdens in the world; it is faced with high TB treatment interruption and loss to follow-up rates. It also has a high prevalence of human immunodeficiency virus (HIV) co-infection in patients with TB and a relatively high mortality in these co-infected patients.[1] Studies of interventions to advance the goal of ending the TB epidemic and improving treatment outcomes are therefore research priorities in South Africa and other LMICs.[2]

Mortality and morbidity from TB is strongly associated with health risk behaviours, particularly smoking and hazardous or harmful alcohol use, both of which are prevalent and often co-occur in patients with TB.[3]–[10] Strategies are also required to improve TB medication adherence in patients with TB and adherence to TB medication and anti-retroviral therapy (ART) in patients co-infected with TB and HIV, both of which may be negatively influenced by excessive alcohol use.[1] There is very limited research on how to concurrently tackle these three risk behaviours - namely smoking, harmful alcohol use and poor medication adherence - in patients with TB, particularly in LMICs.

Motivational Interviewing (MI) has been shown to support reduced drinking, smoking cessation in TB patients and TB treatment and/or antiretroviral therapy (ART) medication adherence.[11]–[13] MI interventions can be effectively delivered by lay health workers (LHWs).[14] The more widespread use of LHWs and the increased use of mobile health (mHealth) digital technologies represent promising ways to increase the scalability of MI interventions. Indeed, the World Health Organisation (WHO) has called for researchers to capitalise on advances in mobile phone technology, network coverage and the increased use of common and widely available digital technologies (including the mobile phone short message service (SMS)) to improve TB care.[15] There is evidence that mHealth technologies can have modest beneficial effects on a range of health outcomes, including medication adherence.[16], [17] Mobile phone messaging also shows a modest effect in improving TB treatment success rates.[18], [19] The evidence is however stronger for two-way messaging and interactive systems for which smart phones are required.[18] These are often not available to patients with TB in Africa.[20]

A limitation of existing MI and mHealth interventions is that they have been studied in the context of modifying a single lifestyle factor. Integrated interventions are likely to be better accepted and more

For peer review only - http://bmjopen.bmj.com/site/about/guidelines.xhtml

effective than multiple interventions targeting different health risk factors.[21], [22] In the case of TB, there is a need for an intervention that has the flexibility to target multiple lifestyle factors as appropriate and in line with patient preferences. This could be achieved through increased integration of TB and non-communicable disease services.[23]

Recent re-engineering of primary health care in South Africa has seen the introduction of municipal ward-based primary healthcare outreach teams of community health workers (CHWS). CHWs work in an integrated, team-based manner, supported by nurses, and take responsibility for health education and promotion, counselling and support for a range of health conditions.[24], [25] Task shifting in this context has been shown to improve population health in LMICs [26] and these teams can be trained and supported to take responsibility for TB/HIV care.[27] Integrated interventions could be implemented within this framework in a feasible and scalable way to improve outcomes for patients with TB across South Africa and beyond.

Building on previous successes with MI and mHealth interventions, we developed a complex behavioural intervention (ProLife) comprising MI-based counselling and SMS, targeting three lifestyle risk behaviours for poor TB outcomes (smoking, hazardous/harmful alcohol consumption and poor medication adherence), and delivered by LHWs. We then conducted a randomised controlled trial (RCT) to assess the effectiveness of the ProLife intervention on improving TB treatment outcomes, smoking abstinence, reducing alcohol consumption and improving adherence to TB and ART medication compared to usual care. The cost-effectiveness of the intervention was also assessed but only the costing results will be presented in this paper.

METHODS

Study design and participants

This was a prospective, two-arm, multicentre, individual RCT, which took place across 27 primary care clinics in 3 districts in South Africa (Lejweleputswa in the Free State province; Bojanala in the North West province; and Sedibeng in Gauteng province). Adult patients (18 years or older) were eligible for the study if they had drug-sensitive Pulmonary TB (PTB), and were initiating TB treatment or had been on TB treatment for less than a month for this treatment episode (both “new” and “retreatment patients”). They had to be tobacco smokers (defined as smoking daily or non-daily in the last four weeks on the Global Adult Tobacco Survey questionnaire)[28] and/or hazardous/harmful drinkers who were not alcohol dependent (Alcohol Use Disorders Identification Test (AUDIT) score ≥ 8 for men or ≥

For peer review only - http://bmjopen.bmj.com/site/about/guidelines.xhtml

7 for women but <20).[29] They also had to have access to a mobile phone and understand one of the 4 languages used for the trial (English, IsiZulu, SeSotho and Setswana). Potential participants were recruited consecutively at the participating clinics between 15 November 2018 and 31 August 2019. Trained field workers identified those interested in the study and screened them for eligibility. If eligible and willing to be enrolled into the trial, written informed consent was obtained. The RCT received ethics approval from the Research Ethics Committees of the five participating research institutions and the trial protocol was previously published.[30]

Randomisation and blinding

Patients were centrally randomised (1:1) to the ProLife intervention or control group using a randomised sequence generator by the trial statistician (MK) who was blind to the arm allocation. We used block randomisation with varying block sizes stratified by clinic so as to achieve equal numbers in intervention and control groups within each clinic. Fieldworkers used sequentially numbered, sealed, opaque envelopes to allocate participants to intervention or control. ProLife involved a complex behavioural intervention therefore LHWs and participants could not be blinded to the intervention. However, the determination of the primary outcome was done by the TB nurses who were blinded to the intervention status of the participants, based on routinely collected data. The statistician (MK) was blinded to the intervention or control arm allocation of participants during the analysis.

Intervention and procedures

The ProLife intervention was developed based on a conceptual framework, following a review of pre-existing evidence.[31] This framework assumed that smoking cessation, reducing harmful alcohol use and improved adherence to TB and HIV treatment would result in improved TB treatment outcomes.[30] The intervention consisted of 3 brief MI counselling sessions, lasting 15-20 minutes, one month apart, delivered by trained LHWs at their TB clinic. The first MI session took place immediately or shortly after the randomisation and involved prioritisation and agenda setting, wherein the participant determined which factor should be prioritised (either a plan to quit tobacco smoking, or reduce or quit drinking, or deal with barriers relating to ART or TB medication adherence). The second and third sessions built on the previous one until all relevant behavioural problems had been addressed. These sessions were reinforced with follow-up SMS text messages, twice a week over 12 weeks.[30] Study patients received 10 TB-related messages followed by seven alcohol reduction- and/or seven smoking cessation-related messages, as appropriate. Messages were aimed at giving information, and augmenting motivation or behavioural skills (we refer to the

For peer review only - http://bmjopen.bmj.com/site/about/guidelines.xhtml

feasibility paper for more details).[31] Applicable SMS-messages were automatically activated after the first MI had taken place. Thereafter remaining messages were delivered even if the participant did not attend the second or third MI session.

Participants randomised to the ProLife intervention also received the same "usual care" as those in the control group. The control group received the usual care and routine treatment and support offered to patients with TB in South Africa, which varies by district but includes: health education; dietetic input; social support; point of care biochemical testing; and HIV testing with pre- and post-test HIV test counselling.

Data were collected at baseline, 3- and 6-months and recorded by fieldworkers equipped with mobile phones with the ProLife mobile data collection application (built with CommCare)[32] installed. They used a standardised electronic case report form (CRF) and followed standard operating procedures (SOPs) to ensure quality. Details of data collection, protection and storage procedures have been reported elsewhere.[30]

Patient and public involvement

Patients or the public were not involved in the design, or conduct, or reporting, or dissemination plans of our research.

Outcomes

Primary Outcome

The primary outcome of TB treatment success at 6 to 9 months follow-up (depending on when it was recorded) was as per the WHO definitions adopted in South Africa,[10] that is, either successful treatment (cured or treatment completed) or failed treatment, death, acquired drug resistance, loss to follow-up (defined as treatment interruption of more than 2 months), or outcome not evaluated. It was measured using the routinely collected TB treatment outcomes in patients' individual files.

Secondary Outcomes

For those participants with bacteriologically confirmed PTB at baseline (either sputum acid fast bacilli (AFB) positive, culture positive or GeneXpert positive PTB), sputum conversion at the end of treatment ("cure rate") was measured as a secondary outcome.[10] Continuous smoking abstinence was assessed at 3- and 6-months follow-up in those participants who were current cigarette smokers at

For peer review only - http://bmjopen.bmj.com/site/about/guidelines.xhtml

baseline. It was defined as having quit smoking completely and a self-report of not smoking more than 5 cigarettes from the start of the study, in addition to a negative biochemical test (exhaled Carbon Monoxide (CO) < 7 ppm).[33], [34] Changes in alcohol consumption were computed using the AUDIT questionnaire scores measured at 3- and 6-months follow-up in those participants who were hazardous/harmful drinkers at baseline.

HIV positive participants were asked about ART status at baseline, 3- and 6-months using standardised questions on the CRF and change in ART status as measured at the 2 follow-up times.

TB and ART medication adherence was measured using modified versions of the AIDS Clinical Trials Group Adherence Questionnaire, a validated tool for measuring adherence specifically to ART.[35] Adherence was measured using an adherence index calculated by the formula (using the 4-day recall): [Total number of doses taken/Total number of doses prescribed] x 100. Patients with at least 95% adherence were classed as having optimal adherence and those with less than 95% were classed as having low (or suboptimal) adherence. This was assessed at 3- and 6-months.

During COVID-19 lockdown (in the second term of 2020) we switched to telephonic follow-up of participants using a shortened questionnaire whereby only strictly needed information for the measurement of outcomes was inquired about.

Training and Intervention Fidelity Monitoring

The training and intervention fidelity monitoring is described in more detail in previous papers.[30], [31] In brief, 18 LHWs, 3 district coordinators and 1 research assistant who focused on counselling supervision underwent MI training over 5 days. LHWs completed a post-session semi-structured form onto which they indicated the extent to which they implemented each element of MI, as well as their general qualitative impressions of that particular session. In addition, we assessed MI intervention fidelity based on ratings of the counsellors' recorded MI sessions, as described below and in footnotes to the supplementary table 4. SMS-message delivery was also assessed.

Economic evaluation

The ProLife intervention costs consisted of the costs of training and the delivery of the ProLife intervention, including relevant personnel involvement (trainers and LHWs), materials used, travel, accommodation and refreshments, and digital infrastructure for the intervention. These were estimated based on research team records. Usual care costs consisted of TB medication costs, biochemical investigations, and ART costs if applicable. These were estimated based on information obtained through routine records. The country specific version of Euroqol with 5 dimensions and 3

For peer review only - http://bmjopen.bmj.com/site/about/guidelines.xhtml

levels of response categories (EQ-5D-3L) for South Africa was administered to participants at baseline, 3- and 6-months follow-up to measure health-related quality of life.[36], [37]

Statistical analysis

The sample size was estimated at 696 in total (348 participants per arm), to detect a 10% difference in TB treatment success rates (0.86 vs. 0.76) in the ProLife arm (intervention) vs. the control arm with 80% power, a significance level of 0.05 and 25% attrition rate. The assumed success rates in the control group were based on actual success rates in patients with TB in the studied provinces obtained from TB managers at the time of the grant application for this study.

We summarised baseline data descriptively by trial arm. For the primary outcome, we conducted statistical analysis on an intention-to-treat basis. We used binary logistic regression to compare the main outcome (TB treatment success rate) between the intervention and the usual care arm. Where treatment outcome data were missing, the outcome was coded as unsuccessful. TB treatment outcomes recorded by the TB nurse were taken on face value as inconsistencies in the dates of bacteriological results did not permit us to verify the correctness of the nurse assessment. We carried out similar statistical analyses for the secondary outcomes with appropriate regression techniques. For the reduction in harmful or hazardous drinking, we used linear regression to estimate the difference in total AUDIT score between control and intervention groups accounting for the baseline AUDIT score as covariate. Separate analyses at 3- and 6-months were performed.

For our main analyses we adjusted for baseline characteristics if these differed between trial arms at baseline. The covariates that we controlled for in each model are specified when a model is presented. The statistical packages STATA [38] and R [39] were used to carry out the analyses, with a P-value < 0.05 considered statistically significant.

The validated Motivational Interviewing Treatment Integrity (MITI) coding tool (version 4.2.1) was used to assess MI intervention fidelity.[40] The coding entailed making 'Global Ratings' (on 4 dimensions: Cultivating change talk, Softening sustain talk, Partnership and Empathy) and 'Behaviour' counts (with respect to the items: giving information, persuade, persuade with permission, question, simple reflection, complex reflection, affirm, seeking collaboration, emphasising autonomy and confront). A score was assigned to each of these items and the scores compared against the competency and proficiency thresholds that are specified in the MITI manual.

For peer review only - http://bmjopen.bmj.com/site/about/guidelines.xhtml

For the analysis of the costs, all costs were collected in South Africa Rand (ZAR) except for the data management system subscription. Results are presented in both ZAR and US dollar ($) using the 2019 OECD exchange rate (1 USD = 14.448 Rand).[36] No South African specific valuation set was available for EQ-5D-3L. The valuation set of Argentina, based on a Visual Analogue Scale, was used to derive utility values, because the GDP per capita in international dollars was the closest between the two countries at the time of analysis.[37], [41] Quality-adjusted life years (QALYs) were derived from the utility values at the time points by calculating the area under the curve.[42] No missing data imputation was performed.

Data are stored in the institutional data repository at Sefako Makgatho Health Sciences University. Data will be embargoed until 30 June 2023 after which they will be freely accessible. [43]

RESULTS

Participant enrolment and follow-up

A total of 2099 patients with TB were screened for eligibility out of which 574 consenting and eligible participants were randomised: 291 to control and 283 to intervention. Trial recruitment was terminated on 31 August 2019 before the planned sample size was reached because of budget and time constraints. In the intervention arm, 227 (80.2%) participants completed the first MI (MI 1) session, 199 (70.3%) MI 2 and 150 (53.0%) MI 3. In the intervention arm, at least one message was delivered to 208 (73.5%) participants, while 99 (35.0%) received all messages. Of those randomised to the control and intervention groups, the primary outcome was recorded in 244 (83.8%) and 253 (89.4%) participants, respectively. (Figure 1)

Figure 1 Consort flow diagram

Baseline participant characteristics of the intervention and control arm

Socio-demographic; socio-economic and clinical characteristics

Baseline characteristics were distributed similarly in the intervention and control arms for most variables but with some imbalances in educational level. A total of 513 (91.3%) participants were new patients with TB, 129 (22.5%) women, and nearly all had PTB (International Classification of Diseases-10 A15) without extrapulmonary TB manifestations (553 (98.9%)). About half of the participants were HIV positive (305 (53.2%)), of whom 204 (65.4%) were on cotrimoxazole and 257 (82.4%) were on ART. (Table 1) Details of marital status, employment, wealth, depression status and co-morbidities are presented in Supplementary table 1.

For peer review only - http://bmjopen.bmj.com/site/about/guidelines.xhtml

Table 1 Baseline descriptive socio-economic statistics and clinical characteristics by study arm

	Control (N = 291) n (%)*	Intervention (N = 283) n (%)*	Total (N=574) n (%)*
**Age in years:** mean (SD)	39.37 (12.60)	38.56 (11.15)	
**Female Sex**	69 (23.7)	60 (21.2)	129 (22.5)
**Education**			
No education	7 (2.4)	5 (1.8)	12 (2.1)
Grades 1-5	23 (7.9)	20 (7.1)	43 (7.5)
Grades 6-7	32 (11.0)	35 (12.4)	67 (11.7)
Grades 8-11	96 (33.0)	128 (45.2)	224 (39.0)
Grade 12	87 (29.9)	70 (24.7)	157 (27.4)
Higher	24 (8.2)	8 (2.8)	32 (5.6)
Declined to answer**	22 (7.6)	17 (6.0)	39 (6.8)
**TB Patient category**			
New patient	264 (92.3)	249 (90.2)	513 (91.3)
Relapse	10 (3.5)	9 (3.3)	19 (3.4)
Re-treatment after default	9 (3.1)	14 (5.1)	23 (4.1)
Re-treatment after failure	1 (0.3)	2 (0.7)	3 (0.5)
Other	2 (0.7)	2 (0.7)	4 (0.7)
**TB Site of disease** Pulmonary only (ICD-10 A15)	281 (98.9)	272 (98.9)	553 (98.9)
**TB Sputum smear, Gene XPert, or culture result available** N=	236	227	463
**At least one Sputum smear, Gene XPert, or culture result positive**	208 (88.1)	195 (85.9)	403 (87.0)
**HIV status**			
Negative	118 (40.7)	125 (44.2)	243 (42.4)
Positive	163 (56.2)	142 (50.2)	305 (53.2)
Unknown	9 (3.1)	16 (5.7)	25 (4.4)
**HIV positive patients**			
**Using Cotrimoxazole**	104 (63.8)	100 (67.1)	204 (65.4)
**Using anti-retroviral**	139 (85.3)	118 (79.2)	257 (82.4)

For peer review only - http://bmjopen.bmj.com/site/about/guidelines.xhtml

* Frequencies and (percentages) are presented unless otherwise stated
**More variables with the option “declined to answer” are listed in the Supplementary table 1

Table 2 Baseline descriptive alcohol and smoking characteristics by study arm

	Control (N=291) n (%)*	Intervention (N=283) n (%)*	Total (N=574) n (%)*
**In the past month, smoked tobacco**			
Not at all**	110 (37.8)	92 (32.5)	202 (35.2)
Daily	149 (51.2)	149 (52.7)	298 (51.9)
Less than Daily	32 (11.0)	42 (14.8)	74 (12.9)
**Had a drink in the past 12-months**	208 (71.5)	223 (78.8)	431 (75.1)
**AUDIT Score (males) : mean (SD) [max: 19]†**	12.27 (3.98)	13.02 (3.78)	12.66 (3.89)
**AUDIT Score (females): mean (SD) [max: 19]†**	11.32 (4.02)	10.98 (4.02)	11.15 (4.0)
**Hazardous/harmful drinking and Smoking Combined (Constructed)**			
Hazardous/harmful drinking Only‡	110 (37.8)	92 (32.5)	202 (35.2)
Smoking Only	103 (35.4)	77 (27.2)	180 (31.4)
Smoking and hazardous/harmful drinking‡	78 (26.8)	114 (40.3)	192 (33.4)

* Frequencies and (percentages) are presented unless otherwise stated
**Non-smokers were only included if they were harmful or hazardous drinkers
†Only hazardous/harmful drinkers and/or current smokers were included in the study. Therefore, patients with TB were excluded if they were non-current smokers and had an AUDIT score <7 (females) or <8 (males) or >19; however, they were included if they were smokers independent of whether they had a drink in the past year and therefore independent of the AUDIT score. These AUDIT scores are thus representative of the mean AUDIT scores in the entire study sample and differ from the AUDIT score in the harmful/hazardous drinkers whose change in AUDIT score was measured at 3 and 6-months follow-up
‡Harmful/hazardous drinking is defined as Alcohol Use Disorders Identification Test (AUDIT) score ≥ 8 for men or ≥ 7 for women but <20

For peer review only - http://bmjopen.bmj.com/site/about/guidelines.xhtml

There were 372 current smokers (298 daily, 74 less than daily). Seventy eight participants (26.8 %) in the control arm were dual smokers *and* drinkers compared to 114 (40.3 %) in the intervention arm. In the control arm, 110 (37.8%) were hazardous/harmful drinkers only and 103 (35.4%) were smokers only, compared to 92 (32.5%) and 77 (27.2) respectively, in the intervention arm. (Table 2) More details of smoking and drinking history, forms of tobacco use, addiction and quit attempts are presented in Supplementary Table 2.

Primary outcome

Overall, 396 (70%) of participants were classified as treated successfully (treatment completed or cured). The remainder either interrupted treatment, failed treatment, developed drug resistance, were transferred out or had an unknown treatment outcome.(Supplementary table 3) The percentage of successful TB treatment did not differ significantly between the control and intervention arm (70.1% vs. 67.8%), odds ratio (OR) for successful TB treatment 0.90 (95%CI: (0.64,1.27)) comparing the intervention arm to the control arm; and was similar to adjusted ORs (Tables 3 and 4).

Secondary outcomes

Cure rates

Among the 403 participants who had at least one positive bacteriological result at baseline, 168 (41.7%) were recorded as cured; of these, 83/205 (39.9%) were in the control arm compared to 85/195 (43.6%) in the intervention arm. The OR of being cured was 1.16 (95% CI: 0.83,1.63) in the intervention versus the control arm; and was similar to the adjusted OR. (Tables 3 and 4)

Continuous smoking abstinence

Among those who identified as cigarette smokers at baseline (345 (60.1%)), 27 had information (self-report plus biochemical verification) to enable the identification of continuous abstinence at 6-months of which 22 had continuously abstained from smoking. These were similarly distributed across the two study arms: 10 (5.59%) participants in the intervention arm compared to 12 (7.23%) in the control arm, OR 0.76 (95% CI: 0.35,1.63). (Tables 3 and 4) At the 3-month follow-up 20 (11.2%) participants in the intervention arm compared to 27 (16.3%) in the control arm continuously abstained from smoking, OR 0.65 (95% CI: 0.37,1.14). (Tables 3 and 5)

For peer review only - http://bmjopen.bmj.com/site/about/guidelines.xhtml

TABLE 3 Descriptive statistics for primary and secondary outcomes by study arm at baseline (where available), 3-months (where available) and 6-months

	Baseline n (%)*			3-months follow-up n (%)*			6-months follow-up n (%)*		
	**Control**	**Intervention**	**Total**	**Control**	**Intervention**	**Total**	**Control**	**Intervention**	**Total**
**TB treatment status[a]**									
Successful**							204 (70.1)	192 (67.8)	396 (69.0)
Not Successful							87 (29.9)	91 (32.16)	178 (31.01)
**Cured [a, ***]**									
Yes							83 (39.9)	85 (43.6)	168 (41.7)
No							125 (60.1)	110 (56.4)	235 (58.3)
**Continuous smoking abstinence[b]**									
Yes				27 (16.3)	20 (11.2)	47 (13.6)	12 (7.2)	10 (5.6)	22 (6.4)
No				139 (83.7)	159 (88.8)	298 (86.4)	154 (92.8)	169 (94.4)	323 (93.6)
**Harmful/hazardous drinkers[!]: N =**	188†	206†	394†	141†	130†	271†	112†	127†	239†
AUDIT score: mean (SD)	12.76 (3.42)	13.12 (3.47)	12.94 (3.45)	8.28 (6.18)	8.84 (5.38)	8.55 (5.81)	8.79 (6.66)	8.70 (5.83)	8.74 (6.22)
Difference from baseline: mean (SD)				-4.61 (6.26)	-4.07 (5.33)	-4.35 (5.83)	-4.25 (6.56)	-4.17 (6.61)	-4.21 (6.57)
**HIV positive patients**:	163† (56.2)	142† (50.2)	305† (53.2)	122†	83†	205†	100†	83†	183†
**Taking ART medication if HIV positive‡**	139 (85.3)	115 (81.0)	254 (83.3)	91 (74.6)	58 (69.9)	149 (72.7)	80 (80.0)	74 (89.2)	154 (84.2)
**ART medication adherence[b]**									
Optimal adherence				101 (99.0)	64 (98.5)	165 (98.8)	75 (97.4)	64 (97.0)	139 (97.2)
Suboptimal				1 (1.0)	1 (1.54)	2 (1.2)	2 (2.6)	2 (3.0)	4 (2.8)

For peer review only - http://bmjopen.bmj.com/site/about/guidelines.xhtml

adherence						
**TB medication adherence**						
Optimal adherence	181 (92.3)	138 (90.8)	319 (91.7)	61 (89.7)	59 (90.8)	120 (90.2)
Suboptimal adherence	15 (7.6)	14 (9.2)	29 (8.3)	7 (10.3)	6 (9.2)	13 (9.8)

a: only assessed at 6-months

b: assessed at 3 and 6-months; this table refers to *cigarette* smokers only (other forms of tobacco smoking are excluded)

* Frequencies and (percentages) are presented unless otherwise stated

**Primary Outcome: This is a binary variable defined as either successful treatment (cured or treatment completed) or failed treatment, death, acquired drug resistance, loss to follow-up or treatment interrupted for more than 2 months, or outcome not evaluated/unknown.

***Based on having a cured treatment outcome among those who were bacteriologically positive at baseline.

! hazardous/harmful drinkers who are not alcohol dependent= AUDIT score ≥ 8 for men or ≥ 7 for women but < 20; *! Important distinction at baseline for eligibility purposes.

†Denominator for the mean (SD) or denominator for %

‡ Information on HIV-positivity was obtained from information from TB records combined with patient self-report at baseline. True HIV-positivity rates may have been higher.

For peer review only

TABLE 4 Regression analysis results for the primary and secondary outcomes at 6-months

	Crude Odds Ratio (95% CI)*	P Value*	Adjusted Odds Ratio (95% CI)*	P Value*
**Primary outcome**				
TB treatment status: Successful (Ref: Not successful)	0.90 (0.64,1.27)	0.548	0.86‡ (0.60,1.24)	0.421
**Secondary outcomes**				
Cured (Ref: Not Cured)	1.16 (0.83,1.63)	0.374	1.07‡ (0.76,1.51)	0.684
Continuous smoking abstinence (Ref: No)***	0.76 (0.35,1.63)	0.482		
TB medication adherence (Ref: Optimal)	0.89 (0.26,3.07)	0.849		
ART medication adherence (Ref: Optimal)	1.17 (0.14,9.94)	0.884		
Taking ART medication (Ref: No)	2.05! (0.80,5.27)	0.136		
AUDIT	-0.04** (-2,1.91)	0.966	0.02!! (-1.55,1.6)	0.976

* Analyses accounted for potential clustering by centre.
‡ Adjusted for district, sex, and smoking/drinking status and HIV status at baseline. It is worth noting that of the variables in the adjusted model; the only statistically significant result is for the district variable.
! Adjusting for art status at baseline
**Controlling for the AUDIT baseline values. The values represent the study arm regression coefficient.
!! Controlling for the AUDIT baseline values and adjusted for district, sex, and smoking/drinking status and HIV status at baseline. The values represent the study arm regression coefficient.
***Given the limited number of those who were identified as continually abstained, we were only able to adjust for one additional variable at a time. Adding one of the following variables: heaviness of smoking, type of drinker at baseline, age when started smoking, and the duration of smoking at baseline; the adjusted odds ratio of continuous abstinence comparing the intervention to the control arm ranged between 0.73 and 0.76 with similar confidence limits as for the crude estimate.

For peer review only - http://bmjopen.bmj.com/site/about/guidelines.xhtml

**TABLE 5** Regression analysis results for secondary outcomes measured at 3-months				
Secondary outcome	Crude Odds Ratio (95% CI)*	P Value*	Adjusted Odds Ratio (95% CI)*	P Value*
Continuous smoking abstinence (Ref: No)**	0.65 (0.37,1.14)	0.135		
TB medication adherence (Ref: Optimal)	1.22 (0.52,2.87)	0.641		
ART medication adherence (Ref: Optimal)	1.58 (0.10,26.12)	0.750		
Taking ART medication (Ref: No)	0.79[!] (0.38,1.65)	0.53	0.74‡ (0.35,1.58)	0.443
AUDIT	0.55† (-1.01,2.11)	0.474	0.74[!!] (-0.62,2.1)	0.273

*Analyses accounted for clustering.
! Adjusting for art status at baseline
‡ Adjusted for art status at baseline, district, sex, and smoking/drinking status and HIV status at baseline.
†Controlling for the AUDIT baseline values. The values represent the study arm regression coefficient.
!! Controlling for the AUDIT baseline values and adjusted for district, sex, and smoking/drinking status and HIV status at baseline. The values represent the study arm regression coefficient.
** Given the limited number of those who were identified as continually abstained, we were only able to adjust for one additional variable at a time. Adding one of the following variables: heaviness of smoking, type of drinker at baseline, age when started smoking, and the duration of smoking at baseline; the adjusted odds ratio of continuous abstinence comparing the intervention to the control arm ranged between 0.63 and 0.66 with similar confidence limits as for the crude estimate.

For peer review only

Change in harmful/hazardous drinking

AUDIT scores were about 4 points lower at both follow-up times than at baseline, independent of the intervention.(Table 3) In the intervention arm, participants had, on average, a reduction of 0.04 points (95% CI: -2,1.91) on the AUDIT score at 6-months, compared to those in the control arm controlling for baseline scores; whereas an average increase of 0.55 (95% CI: -1.01,2.11) was observed at 3-months. (Tables 4 and 5)

Medication adherence and ART uptake

At 6-months, the OR of taking ART medication was 2.05 (95% CI: 0.80,5.27) comparing the intervention arm to the control arm and controlling for ART baseline medication status; whereas it was 0.79 (95% CI: 0.38,1.65) at 3-months. The proportion of participants who had optimal TB medication adherence was 90.2% (120/133) at 6-months and 91.7% (319/348) at 3-months. Suboptimal TB medication adherence ORs were 0.89 (95%CI: 0.26,3.07) and 1.22 (95%CI: 0.52,2.87) comparing intervention arm to the control arm at 6-months and 3-months, respectively. The proportions of participants on ART who had optimal ART medication adherence were high at both 3-months (165/167, 98.8%) and 6-months follow-up (139/143, 97.2%). Suboptimal ART medication adherence ORs were 1.17 (95%CI: 0.14,9.94) and 1.58 (95%CI: 0.10,26.12) comparing the intervention arm to the control arm at 6-months and 3-months, respectively. (Tables 3, 4 and 5)

Intervention fidelity

Motivational interviewing fidelity

The recordings of 17 counsellors (one each) were transcribed verbatim and then assessed. In terms of the global ratings, the LHWs' counselling sessions were above proficiency levels on all items, namely, cultivating change talk, softening sustain talk, partnership, and empathy (as the mean scores were all above 2). In terms of the summary measures, the LHWs' counselling sessions did not achieve the basic proficiency threshold of 3.5 for the Relational component (Partnership + Empathy) as their mean score was 3.1 (SD=1.19). However, their mean score on the Technical component (Cultivating Change Talk + Softening Sustain Talk) of 3.3 (SD=0.97) was above the threshold of 3. For behavioural counts, "Asking Questions" had the highest mean score (24.2; SD=10.42), followed by "Affirm" with a mean score of 5.5 (SD=3.7). The counsellors were least likely to engage in the following: Persuade with permission and emphasising autonomy. The mean Reflections to Questions Ratio was 0.23 (SD=0.24). The LHWs made on average 9.3 (SD=4.74) MI adherent (Affirm, Emphasize Autonomy and Seek Collaboration) and 1.2 (SD=2.28) MI non-adherent (Confront and Persuade) statements per session. (Supplementary Table 4)

For peer review only - http://bmjopen.bmj.com/site/about/guidelines.xhtml

SMS-delivery

Of the total number of Information-Motivation-Behaviour messages triggered, 3583 (80.4%) were delivered. All due SMS messages were delivered to 95 (41.9%) of the participants who completed the first MI, See Supplementary Table 5 for more details.

Costs and health related quality of life

Unit costs used to estimate the mean costs are presented in the Supplementary Table 6. Incremental cost-utility ratios are not presented since the intervention was not clinically effective. The mean cost of the ProLife intervention was ZAR 2601 (SD 6) ($180.02 (SD $0.42)) per participant in the intervention arm (n=283). The mean cost of usual care was ZAR 681 (SD 357) ($47.13 (SD $24.71)) in the intervention arm (n=122) versus ZAR 706 (SD 302) ($48.86 (SD $20.90)) in the control arm (n=131). The total mean cost of care including the intervention was ZAR 3285 (SD R357) ($227.37 (SD 24.71)) in the intervention arm (n=122). EQ-5D-3L data were available at the 3 time points for 137 intervention and 159 control arm participants. The mean QALYs estimated over six months were 0.442 (SD 0.061) in the intervention arm versus 0.430 (SD 0.074) in the control arm (adjusted mean difference 0.006 (95% CI -0.001 to 0.013)).

DISCUSSION

This RCT did not provide evidence for improved TB treatment success rates in those receiving the ProLife intervention compared to those receiving usual care. We could also not demonstrate significant beneficial effects on any of the secondary outcomes, i.e., smoking, alcohol consumption, medication adherence and ART initiation. To our knowledge there are no other published studies of similar complex interventions that aim to improve TB treatment outcomes in patients who smoke or drink to harmful or hazardous extent. Interventions evaluated by other studies were either complex interventions or SMS-based interventions aimed at improving TB outcomes through the pathway of increasing adherence, but without an alcohol or smoking intervention component [44], [45] or focused on a single behaviour, namely smoking or drinking.[46], [47]. Of the latter studies, a brief smoking cessation intervention was effective in inducing smoking cessation in patients with TB but did not improve TB outcomes.[46] Conversely, in another study in India, intensive counselling for alcohol disorders led to significantly better TB treatment outcomes in the intervention group compared to the control group.[47] Smoking cessation also led to better TB treatment outcomes in a secondary analysis of a large tobacco cessation trial in patients with TB in Bangladesh and Pakistan.[48] Our non-

For peer review only

significant result for smoking-related outcomes is not consistent with findings from our previous TB study, which used a single MI session and found that the chance of sustained smoking cessation was twice as high in the MI intervention group compared to the control group,[14] albeit with a less stringent exhaled CO cut-off point. Evidence on the effectiveness of MI for smoking abstinence in non-TB settings has been equivocal.[49] Self-reported alcohol consumption decreased with about 4 points in both intervention and control arms in our study at both follow-up times. Answering questions on drinking in brief intervention trials may alter subsequent self-reported behaviour: exposing non-intervention control groups to an integral component of the intervention may therefore underestimate the effect of the intervention.[50] There have been few previous studies looking at MI and SMS interventions for the modification of hazardous/harmful drinking in the context of TB. A previous trial of a brief counselling intervention to reduce alcohol consumption in patients with TB did not find a significant effect on alcohol reduction.[51] Outside a TB setting, results have been mixed. A meta-analysis showed a small but significant improvement in outcomes when MI was used in conjunction with cognitive behavioural therapy for co-morbid alcohol use and depression.[52] Self-reported TB and ART medication adherence was high overall in our study population, which is consistent with other studies conducted in South Africa.[53], [54] It is possible that we did not find a difference in treatment adherence due to a ceiling effect.

There were several key strengths in this RCT. This was an individually RCT with a relatively large sample size and a high follow up rate (87%) for the primary outcome. Primary outcome assessment was blinded. This was a novel intervention, which built on previous successes with both MI and mHealth interventions and was aligned with the WHO's call to increase the use of digital technologies to improve TB care.[15] We utilized a validated alcohol consumption questionnaire (AUDIT) [29] and a four-day timeline follow-back for medication adherence to reduce recall bias as self-reports tend to underreport drinking while overestimating adherence behaviour compared with.[55] [35] Smoking cessation was confirmed with exhaled CO using strict cut-off points. Overall, the quality of the counselling was acceptable. The results of our MI analyses suggest that the LHWs trained as counsellors were more proficient in MI than during the feasibility stage, as observed by their global rating scores on cultivating change talk, softening sustain talk, partnership and empathy. (Supplementary Table 4) These results were achieved by ongoing monitoring and training of LHWs during the trial and adapting the training based on feedback from the feasibility stage. Extra counsellors were also appointed to minimise travel distances to clinics. There were some limitations associated with this RCT. Trial recruitment had to be terminated before the planned sample size because of funding and time constraints. Nevertheless, the calculation of sample size was based on

For peer review only

an anticipated 25% LTFU for the primary outcome, while in reality only 13.4 %, of the TB outcomes were not available. As a result, we achieved a slightly higher power to detect the a 10% difference in primary outcomes, than we had aimed for (83% vs. 80%). The smaller sample size did however reduce the power to detect a difference for secondary outcomes for which the LTFU was much higher than 25%. Also, the calculated sample size was not powered for subgroup analysis which was the case for outcomes relating to smoking, drinking, ART and cure rates. In addition, due to COVID-19 lockdown in March 2020, we had to switch to telephonic follow-up of participants using a shortened questionnaire (22 participants) and could not access clinics to retrieve outstanding TB treatment outcomes. The low intervention uptake meant that half of the participants received only one or two MI sessions combined with SMS messages. SMS messages were only used for the first half of the study period, and one quarter of participants did not receive their messages, a commonly occurring problem in developing countries.[20], [56] It could be argued that in the absence of ongoing text messages, the MI and associated text messages were not enough to keep participants focussed for the second three months of the trial. The 2-arm study design did not permit the untangling of the individual effects of SMS and MI. Understanding their separate effects could have important cost implications as SMS-communication would be cheaper and easier to organise than individual counselling.

The lack of effectiveness of our intervention on the primary outcome (TB treatment success) can have a number of possible explanations. Although intervention uptake was high (80.2%) for the first counselling session, many participants did not return for the second (29.7%) and third (47%) sessions. As a result of this only about half of the intervention arm participants received all three MI sessions. Furthermore, about one quarter of all participants did not receive any SMS-messages. Low intervention uptake leads to a dilution of any potential effects. The lack of effectiveness on TB treatment success could perhaps also be explained by the complexity of the ProLife intervention itself: counsellors had to address multiple behaviours, namely medication adherence, tobacco smoking and hazardous/harmful drinking. Despite having established the feasibility and acceptability of this approach [31] and ongoing on site performance monitoring and feedback of counsellors, it is possible that MI for multiple behaviour change in the ProLife study was counterproductive as counsellors may have ended up not focusing on any of the behaviours at optimal levels. Similarly, patients might have found it difficult to change multiple behaviours simultaneously, especially because smoking and drinking are mutually reinforcing. This integrated approach was nevertheless adopted to avoid the need for multiple vertical counselling services (in addition to TB treatment and HIV treatment), to allow the different elements of the programme to reinforce one another, and to improve the affordability, feasibility and acceptability for a future roll-out of the programme. It is also possible that sequential interventions may be better, at least for smoking cessation.[57] More intensive counselling

For peer review only

(more sessions) or a modified counselling method may have been more appropriate, even more so since a recent review of reviews of MI casts doubt on its efficacy.[49], [58], [59] For example, more emphasis on increasing patient knowledge in addition to increasing self-efficacy may have been more effective.[60] The cause of the mobile health message delivery problems (such as poor network coverage, no electricity to charge phones) [61] would need to be investigated in order to increase the effectiveness of future mHealth interventions. Messages may also have to be intensified or modified to be more interactive and/or tailored to specific circumstances of each individual. This would improve the personal value of the intervention to the individual, which is likely to increase the chances of their participation in the intervention.[62] Consistent with the normalisation process theory,[63] cognitive participation in the intervention might have been higher had we been deliberate in the implementation to ensure the TB nurse who would have routinely seen the participants, provided additional support and motivated participants to attend MI sessions with the counsellor. In this way, the intervention would have gained 'legitimacy' but this would have led to unblinding of the nurses to the intervention arm.

In conclusion, we could not demonstrate that the ProLife intervention was effective in improving TB treatment outcomes. This may be due to the lack of effect of the intervention, but the study may also have been underpowered for the intermediary secondary outcomes. Valuable lessons were learnt on challenges relating to training LHWs in MI counselling and delivery, SMS-delivery in a challenging socio-economic context and the reasons for loss to follow-up of TB participants with multiple health problems. Further research is needed to provide answers on how to increase intervention uptake in poor resource settings and whether our complex intervention should have been more intensive. Other important questions are whether another counselling method would have been more effective. Lastly, in the light of the already existing evidence of SMS and the costs and implementation challenges relating to MI, intervention studies limited to an mHealth intervention but using different intensities, duration and type of interventions (one-way, two-way, interactive) are needed.

For peer review only

Acknowledgements

We are grateful to the following people: Ms M Malefo for her work as project manager, Ms D Bell for conducting the MI training, Ms N Kitleli for supervising the lay counsellors, Mesadie Machaea-Malinga for conducting the fidelity assessments, the district and clinic TB managers and TB nurses, the fieldworkers and the lay counsellors. The authors are also grateful to the National Department of Health and the three Provincial Departments of Health for granting permission for this study.

Contributors

MK contributed to study design and the manuscript, led on the sample size determination and did the statistical analysis. NKM contributed to study design, particularly with respect to development of the MI intervention, did the analysis of the MI data and contributed to the manuscript writing. AVZ led the data management and SMS-system, monitored data quality and contributed to data analysis and manuscript writing. ASM contributed to study design and led on the preparation of the manuscript. NDM contributed to study design, intervention development and manuscript preparation. JL and SP led on the economic evaluation. AT contributed to the economic evaluation. JT was site lead for the Bojanala sub-district. OBO was site lead for the Sedibeng sub-district. MOB contributed to study design, data interpretation and the manuscript. KS and OAA are co-principal investigators. Both contributed to study design, data interpretation and manuscript writing. All authors read and approved the final draft of the manuscript.

Funding

This project was funded by the SA-Medical Research/Newton Foundation Grant on TB control implementation science. (UK/South Africa Newton Fund RFA: TB control implementation science (MRC-RFA-02: TB -05-2015)). This UK funded award is part of the EDCTP2 programme supported by the European Union.

The funder had no role in study design; collection, management, analysis, and interpretation of data; writing of the report or the decision to submit the report for publication.

Trial sponsor: Sefako Makgatho Health Sciences University, RSA. Contact: lekan.ayo-yusuf@smu.ac.za

For peer review only

Competing interests

OA Ayo-Yusuf, the principal investigator, received a research grant (MRC-RFA-02: TB - 05-2015) which was allocated to Sefako Makgatho Health Sciences University to pay for the expenses related to the research project. As co-principal investigator K Siddiqi received part of the MRC-Newton research grant (MRC-RFA-02: TB -05-2015) which was allocated to the University of York to support the research by the authors affiliated to the University of York (K Siddiqi, ND Mdege, M Kanaan, S Parrot, J Lee). Goedele Louwagie received a small monthly remuneration per month from 1 October 2018 until 30 June 2020 as academic research coordinator. André van Zyl was employed as a research data manager on a part-time basis from 1 September 2017 and 30 June 2020. OA Ayo-Yusuf received support for attending meetings and for travel by Tax Justice Network Africa. He is the Vice Chair of the Standing Committee on Health, Academy of Science, SA (not remunerated). The remaining authors declare no competing interests.

Ethical considerations

This study received ethics approval from the Research Ethics Committees of: Sefako Makgatho Health Sciences University (SMU; Ref: SMUREC/D/234/2017: IR); the South African Medical Research Council (with the SMU Ethics Committee serving as the Research Ethics Committee Record); the University of Pretoria (Ref: 434/2017); the University of Witwatersrand (M160455); and the University of York (no reference number, approval date 15 January 2017).

Patient Consent for publication

Not applicable

Data availability statement

The study protocol was previously published.(30) The de-identified participant and SMS data sets are stored in labelled Stata files and are accompanied by a statistical analysis plan and metadata explaining each variable. Data will be embargoed for data analysis until 30 June 2023. Thereafter, permission must be obtained from the principal investigators (OA and KS) for any data analysis not yet performed by the primary research group. Data are stored in the institutional data repository at Sefako Makgatho

For peer review only

Health Sciences University called Discover research (https://smu-za.figshare.com/) with a CC-BY 4.0 (Attribution) license (Creative Commons — Attribution 4.0 International — CC BY 4.0). The statistical analysis plan is available as supplementary material.

Open access

This is an open access article distributed in accordance with a Creative Commons Attribution (CC BY 4.0) license, which permits others to copy, redistribute, remix, transform and build upon this work for any purpose, provided the original work is properly cited, a link to the licence is given and an indication of whether changes were made. See: https://creativecommons.org/licenses/by/4.0/.

References

1. World Health Organisation. Global tuberculosis report 2020. Available: https://apps.who.int/iris/bitstream/handle/10665/336069/9789240013131-eng.pdf [Accessed 4 Aug 2021].
2. Fitchett JR, MacPherson P. Implementing the End TB Strategy and the intersection with the Sustainable Development Goals. *Trans R Soc Trop Med Hyg* 2016;110:145–7.
3. Thomas BE, Thiruvengadam K, Rani S, et al. Smoking, alcohol use disorder and tuberculosis treatment outcomes: A dual co-morbidity burdenthat cannot be ignored. *PLoS ONE* 14(7):e0220507.
4. Imtiaz S, Shield KD, Roerecke M, et al. Alcohol consumption as a risk factor for tuberculosis: meta-analyses and burden of disease. *Eur Respir J* 2017;50: 1700216.
5. Ogbo FA, Ogeleka P, Okoro A, et al. Tuberculosis disease burden and attributable risk factors in Nigeria, 1990–2016. *Tropical Medicine and Health* 2018;46:34.
6. Azeez A, Mutambayi R, Odeyemi A, et al. Survival model analysis of tuberculosis treatment among patients with human immunodeficiency virus coinfection. *Int J Mycobacteriol* 2019;8:244-51.
7. Mollel EW, Chilongola JO. Predictors for Mortality among Multidrug-Resistant Tuberculosis Patients in Tanzania. J Trop Med 2017;2017:9241238. doi: 10.1155/2017/9241238.
8. Peltzer K. Conjoint alcohol and tobacco use among tuberculosis patients in public primary healthcare in South Africa. *S Afr J Psychiatr* 2014;20(1):21–6.
9. Ragan EJ, Kleinman MB, Sweigart B, et al. The impact of alcohol use on tuberculosis treatment outcomes: a systematic review and meta-analysis. *Int J Tuberc Lung Dis*

2020;24(1):73–82.

10. Department of Health of South Africa. National Tuberculosis Management Guidelines. Pretoria: 2014. Available from: http://www.tbonline.info/media/uploads/documents/ntcp_adult_tb-guidelines-27.5.2014.pdf [Accessed 4 Aug 2021].

11. Harder VS, Musau AM, Musyimi CW, et al. A randomized clinical trial of mobile phone motivational interviewing for alcohol use problems in Kenya. *Addiction* 2020 ;115:1050-1060.

12. Palacio A, Garay D, Langer B, et al. Motivational Interviewing Improves Medication Adherence: a Systematic Review and Meta-analysis. *Gen Intern Med.* 31:929–40. DOI: 10.1007/s11606-016-3685-3.

13. Madhombiro M, Kidd M, Dube B, et al. Effectiveness of a psychological intervention delivered by general nurses for alcohol use disorders in people living with HIV in Zimbabwe: a cluster randomized controlled trial. *J Int AIDS Soc* 2020 Dec;23:e25641.

14. Louwagie GMC, Okuyemi KS, Ayo-Yusuf OA. Efficacy of brief motivational interviewing on smoking cessation at tuberculosis clinics in Tshwane, South Africa: a randomized controlled trial. *Addiction* 2014;109:1942–52

15. World Health Organization. Handbook for the use of digital technologies to support tuberculosis medication adherence, 2017. Available: https://apps.who.int/iris/handle/10665/259832 [Accessed 4 Aug 2021].

16. Hirsch-Moverman Y, Daftary A, Yuengling KA, et al. Using mhealth for HIV/TB treatment support in lesotho: Enhancing patient-provider communication in the start study. *J Acquir Immune Defic Syndr* 2017;74:S37–43.

17. Shah R, Watson J, Free C. A systematic review and meta-analysis in the effectiveness of mobile phone interventions used to improve adherence to antiretroviral therapy in HIV infection. *BMC Public Health* 2019;19:915.

18. Gashu KD, Gelaye KA, Mekonnen ZA, et al. Does phone messaging improves tuberculosis treatment success? A systematic review and meta-analysis. *BMC Infect Dis* 2020;20:42.

19. Ngwatu BK, Nsengiyumva NP, Oxlade O, et al. The impact of digital health technologies on tuberculosis treatment: a systematic review. Eur Respir J. 2018 11;51(1):1701596. doi: 10.1183/13993003.01596-2017

20. Ggita JM, Ojok C, Meyer AJ, et al. Patterns of usage and preferences of users for tuberculosis-related text messages and voice calls in Uganda. *Int J Tuberc Lung Dis* 2018;22:530-536.

21. Oosterveen E, Tzelepis F, Ashton L, et al. A systematic review of eHealth behavioral interventions targeting smoking, nutrition, alcohol, physical activity and/or obesity for young

For peer review only

adults. *Prev Med* 2017;99:197–206.

22. Haug S, Paz Castro R, Kowatsch T, et al. Efficacy of a technology-based, integrated smoking cessation and alcohol intervention for smoking cessation in adolescents: Results of a cluster-randomised controlled trial. *J Subst Abuse Treat* 2017;82:55–66.

23. Siddiqi K, Stubbs B, Lin Y, et al. TB multimorbidity: a global health challenge demanding urgent attention. *Int J Tuberc Lung Dis* 2021;25:87–90.

24. Moosa S, Derese A, Peersman W. Insights of health district managers on the implementation of primary health care outreach teams in Johannesburg, South Africa: A descriptive study with focus group discussions. *Hum Resour Health* 2017;15:1–9.

25. Marcus TS, Hugo J, Jinabhai CC. Which primary care model? A qualitative analysis of ward-based outreach teams in South Africa. *African J Prim Heal Care Fam Med* 2017;9:1–8.

26. Seidman G, Atun R. Does task shifting yield cost savings and improve efficiency for health systems? A systematic review of evidence from low-income and middle-income countries. *Hum Resour Health* 2017;15:1–13.

27. Heunis C, Wouters E, Kigozi G, et al. TB/HIV-related training, knowledge and attitudes of community health workers in the Free State province, South Africa. *African J AIDS Res* 2013;12:113–9.

28. World Health Organization & Centers for Disease Control (U.S.). Tobacco questions for surveys: a subset of key questions from the Global Adult Tobacco Survey (GATS): global tobacco surveillance system. *World Health Organization* 2011. Available from: https://apps.who.int/iris/handle/10665/87331 [Accessed 16 Nov 2021]

29. Bohn MJ, Babor TF, Kranzler HR. The Alcohol Use Disorders Identification Test (AUDIT): validation of a screening instrument for use in medical settings. *J Stud Alcohol* 1995;56:423–32.

30. Moriarty AS, Louwagie GM, Mdege ND, et al. ImPROving TB outcomes by modifying LIFE-style behaviours through a brief motivational intervention followed by short text messages (ProLife): Study protocol for a randomised controlled trial. *Trials* 2019;20:1–12.

31. Louwagie GM, Morojele N, Siddiqi K, et al. Addressing tobacco smoking and drinking to improve TB treatment outcomes, in South Africa: A feasibility study of the ProLife program. *Transl Behav Med* 2020; 10: 1491–1503.

32. CommCare. Cambridge, MA: Dimagi; 2017. Available from: https://play.google.com/store/apps/details?id=org.commcare.dalvik&hl=en_US&gl=US. [Accessed 15 June 2021].

33. Siddiqi K, Khan A, Ahmad M, et al. Action to stop smoking in suspected tuberculosis (ASSIST)

For peer review only

in Pakistan: a cluster randomized, controlled trial. *Ann Intern Med* 2013;158:667–75.

34. West R, Hajek P, Stead L, et al. Outcome criteria in smoking cessation trials: proposal for a common standard. *Addiction* 2005;100:299–303.

35. van den Boogaard J, Lyimo RA, Boeree MJ, et al. Electronic monitoring of treatment adherence and validation of alternative adherence measures in tuberculosis patients: A pilot study. *Bull World Health Organ* 2011;89:632–9.

36. OECD [Internet]. Available from: https://data.oecd.org/conversion/exchange-rates.htm [Accessed 26 Oct 2021]

37. Augustovski FA, Irazola VE, Velazquez AP, et al. Argentine valuation of the EQ-5D health states. *Value Heal* 2009;12:587–96.

38. StataCorp. Stata Statistical Software: Release 15. College Station, TX: StataCorp LLC. 2017.

39. Team RC. R: A language and environment for statistical computing. Vienna, Austria: R Foundation for Statistical Computing; 2017. Available from: https://www.r-project.org/ [Accessed 04 Aug 2021]

40. Moyers TB, Rowell LN, Manuel JK, et al. The Motivational Interviewing Treatment Integrity Code (MITI 4): Rationale, Preliminary Reliability and Validity. *J Subst Abuse Treat* 2016;65:36-42. doi:10.1016/j.jsat.2016.01.001

41. International Monetary Fund. IMF DataMapper - GDP per capita, current prices (Purchasing power parity; international dollars per capita), 2018. Available from: https://www.imf.org/external/datamapper/datasets [Accessed 04 Aug 2021]

42. Richardson GMA. Calculation of quality adjusted life years in the published literature: a review of methodology and transparency. *Health Econ* 2004;13:1203–10.

43. [Dataset] van Zyl A, Kanaan M, Louwagie GM, et al. Data from: Effect of a brief motivational interview and text message intervention targeting tobacco smoking, alcohol use, and medication adherence to improve tuberculosis treatment outcomes in adult patients with tuberculosis: a multicentre, randomised controlled trial of the ProLife programme in South Africa. Data embargoed until 30 06 2023. Figshare Digital Repository, January 18, 2022. https://doi10.25443/smu-za.15057132

44. Khachadourian V, Truzyan N, Harutyunyan A, et al. People-centred care versus clinic-based DOT for continuation phase TB treatment in Armenia: a cluster randomized trial. *BMC Pulm Med* 2020;20:105.

45. Thiam S, LeFevre AM, Hane F, et al. Effectiveness of a strategy to improve adherence to tuberculosis treatment in a resource-poor setting: a cluster randomized controlled trial. *JAMA* 2007;297:380–6.

For peer review only

46. Goel S, Kathiresan J, Singh P SR. Effect of a brief smoking cessation intervention on adult tobacco smokers with pulmonary tuberculosis: A cluster randomized controlled trial from North India. *Indian J Public Heal* 2017;61(Suppl 1):S47–53
47. Manogaran C, Nagarajan K, Ovung S, et al. Alcohol intervention strategy among tuberculosis patients: a pilot study from South India. *Int J Tuberc Lung Dis* 2017;21:947–52
48. Siddiqi K, Keding A, Marshall AM, et al. Effect of quitting smoking on health outcomes during treatment for tuberculosis: secondary analysis of the TB & Tobacco Trial. *Thorax* 2021 Published Online First Jul 16:thoraxjnl-2020-215926. doi: 10.1136/thoraxjnl-2020-215926.
49. Lindson N, Thompson TP, Ferrey A, et al. Motivational interviewing for smoking cessation. *Cochrane Database Syst Rev*. 2019;2019(7).
50. McCambridge J, Kypri K. Can simply answering research questions change behaviour? Systematic review and meta analyses of brief alcohol intervention trials. *PLoS One* 2011;6(10):e23748.
51. Peltzer K, Naidoo P, Louw J, et al. Screening and brief interventions for hazardous and harmful alcohol use among patients with active tuberculosis attending primary public care clinics in South Africa: results from a cluster randomized controlled trial. *BMC Public Health* 2013;13:699.
52. Riper H, Andersson G, Hunter SB, et al. Treatment of comorbid alcohol use disorders and depression with cognitive-behavioural therapy and motivational interviewing: a meta-analysis. *Addiction* 2014;109:394–406.
53. Moosa A, Gengiah TN, Lewis L, et al. Long-term adherence to antiretroviral therapy in a South African adult patient cohort: a retrospective study. *BMC Infect Dis* 2019;19(1):775. doi: 10.1186/s12879-019-4410-8.
54. Webb Mazinyo E, Kim L, Masuku S, et al. Adherence to Concurrent Tuberculosis Treatment and Antiretroviral Treatment among Co-Infected Persons in South Africa, 2008–2010. *PLoS One* 2016;11(7): e0159317. https://doi.org/10.1371/journal.pone.0159317.
55. Stirratt MJ, Dunbar-Jacob J, Crane HM, et al. Self-report measures of medication adherence behavior: recommendations on optimal use. *Transl Behav Med* 2015;5:470–82.
56. Wanyama JN, Nabaggala SM, Kiragga A, et al. High mobile phone ownership but low internet access and use among young adults attending an urban HIV clinic in Uganda. *Vulnerable Child Youth Stud* 2018;13:3,207-20.
57. Meader N, King K, Wright K et al. Multiple risk behavior interventions: Meta-analyses of RCTs. *Am J Prev Med* 2017;53:e19–e30.
58. Abraham C, Michie S. A taxonomy of behavior change techniques used in interventions.

For peer review only

Health Psychol 2008;27:379-87.

59. Frost H, Campbell P, Maxwell M, et al. Effectiveness of Motivational Interviewing on adult behaviour change in health and social care settings: A systematic review of reviews. *PLoS One* 2018;18:13(10):e0204890

60. Kahwati L, Viswanathan M, Golin CE, et al. Identifying configurations of behavior change techniques in effective medication adherence interventions: a qualitative comparative analysis. *Syst Rev* 2016;5(83).

61. Telerivet. Message Statuses . User Guide, 2021. Available from: https://guide.telerivet.com/hc/en-us/articles/360038959611-Message-Statuses. [Accessed 21 June 2021]

62. Lustria MLA, Noar SM, Cortese J, et al. A meta-analysis of web-delivered tailored health behavior change interventions. *Journal of Health Communication* 2013;18:1039–69.

63. Gallacher K, May CR, Montori V, et al. Understanding Patients' Experiences of Treatment Burden in Chronic Heart Failure Using Normalization Process Theory. *Ann Fam Med* 2011;9:235-243.

For peer review only

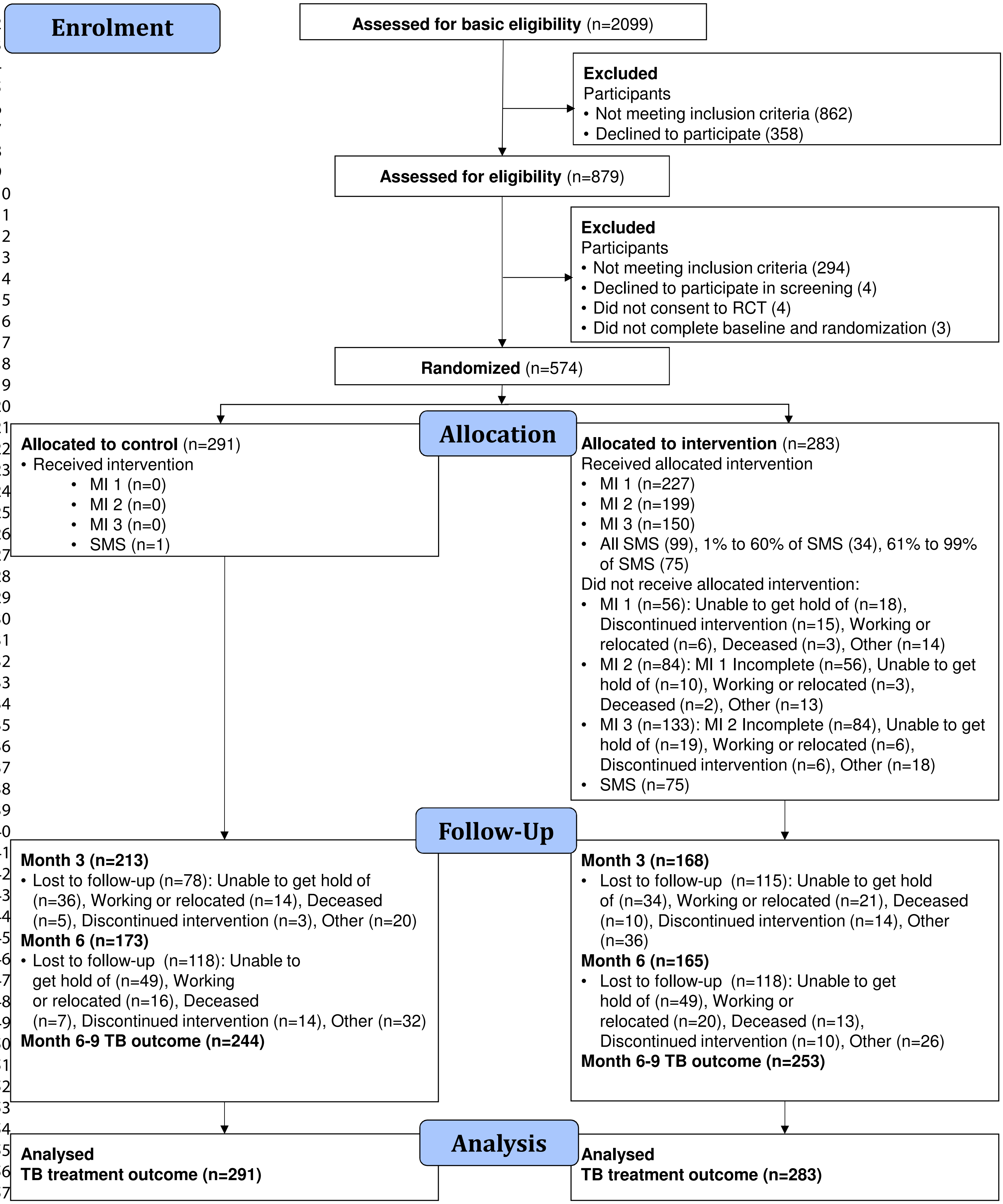
Enrolment
Assessed for basic eligibility (n=2099)
Excluded
Participants
• Not meeting inclusion criteria (862)
• Declined to participate (358)
Assessed for eligibility (n=879)
Excluded
Participants
• Not meeting inclusion criteria (294)
• Declined to participate in screening (4)
• Did not consent to RCT (4)
• Did not complete baseline and randomization (3)
Randomized (n=574)
Allocation
Allocated to control (n=291)
• Received intervention
• MI 1 (n=0)
• MI 2 (n=0)
• MI 3 (n=0)
• SMS (n=1)
Allocated to intervention (n=283)
Received allocated intervention
• MI 1 (n=227)
• MI 2 (n=199)
• MI 3 (n=150)
• All SMS (99), 1% to 60% of SMS (34), 61% to 99% of SMS (75)
Did not receive allocated intervention:
• MI 1 (n=56): Unable to get hold of (n=18), Discontinued intervention (n=15), Working or relocated (n=6), Deceased (n=3), Other (n=14)
• MI 2 (n=84): MI 1 Incomplete (n=56), Unable to get hold of (n=10), Working or relocated (n=3), Deceased (n=2), Other (n=13)
• MI 3 (n=133): MI 2 Incomplete (n=84), Unable to get hold of (n=19), Working or relocated (n=6), Discontinued intervention (n=6), Other (n=18)
• SMS (n=75)
Follow-Up
Month 3 (n=213)
• Lost to follow-up (n=78): Unable to get hold of (n=36), Working or relocated (n=14), Deceased (n=5), Discontinued intervention (n=3), Other (n=20)
Month 6 (n=173)
• Lost to follow-up (n=118): Unable to get hold of (n=49), Working or relocated (n=16), Deceased (n=7), Discontinued intervention (n=14), Other (n=32)
Month 6-9 TB outcome (n=244)
Month 3 (n=168)
• Lost to follow-up (n=115): Unable to get hold of (n=34), Working or relocated (n=21), Deceased (n=10), Discontinued intervention (n=14), Other (n=36)
Month 6 (n=165)
• Lost to follow-up (n=118): Unable to get hold of (n=49), Working or relocated (n=20), Deceased (n=13), Discontinued intervention (n=10), Other (n=26)
Month 6-9 TB outcome (n=253)
Analysis
Analysed
TB treatment outcome (n=291)
Analysed
TB treatment outcome (n=283)

SUPPLEMENTARY TABLES

Supplementary table 1 Detailed baseline descriptive statistics for socio-demographic, socio-economic, and clinical characteristics by study arm

	Control (N = 291) n (%)*	Intervention (N = 283) n (%)*	Total (N=574) n (%)*
**Marital status**			
Married or living together	102 (35.1)	95 (33.6)	197 (34.3)
Divorced/separated	20 (6.9)	18 (6.4)	38 (6.6)
Widowed	10 (3.4)	7 (2.5)	17 (3.0)
Never married and never lived together	144 (49.5)	150 (53.0)	294 (51.2)
Declined to answer	15 (5.2)	13 (4.6)	28 (4.9)
**Employment**			
Self-employed (full-time)	30 (10.3)	36 (12.7)	66 (11.5)
Employed full-time (30 hrs a week or more)	62 (21.3)	54 (19.1)	116 (20.2)
Employed part-time (less than 30 hrs a week)	19 (6.5)	29 (10.2)	48 (8.4)
Retired	17 (5.8)	16 (5.7)	33 (5.7)
Unemployed (but able to work)	125 (43.0)	120 (42.4)	245 (42.7)
Unable to work because of long-term disability or ill health	9 (3.1)	8 (2.8)	17 (3.0)
Full-time student	12 (4.1)	4 (1.4)	16 (2.8)
Caring from my home and family/doing household work/housewife	0 (0.0)	2 (0.7)	2 (0.3)
Occasional work ("piece job")	17 (5.8)	12 (4.2)	29 (5.1)
Declined to answer	0 (0.0)	2 (0.7)	2 (0.3)
**Ever worked or spent time in mines**			
No	244 (83.8)	237 (83.7)	481 (83.8)
Yes	46 (15.8)	45 (15.9)	91 (15.9)
**Household items (Yes is displayed)**			
A radio	249 (85.6)	234 (82.7)	483 (84.1)
A television	255 (87.6)	255 (90.1)	510 (88.9)
A landline telephone	21 (7.2)	22 (7.8)	43 (7.5)
A desktop or laptop computer	67 (23.0)	48 (17.0)	115 (20.0)
A refrigerator	248 (85.2)	240 (84.8)	488 (85.0)
A vacuum cleaner or floor pol	49 (16.8)	35 (12.4)	84 (14.6)
A microwave oven	198 (68.0)	189 (66.8)	387 (67.4)
An electric or gas stove	254 (87.3)	238 (84.1)	492 (85.7)
A washing machine	153 (52.6)	136 (48.1)	289 (50.3)
Total number of assets: mean (SD)	5.14 (1.96)	4.94 (1.77)	5.04 (1.87)
**In the past month, number of days you or people in the household went to bed hungry because there was no food to eat**			
0 days	244 (83.8)	238 (84.1)	482 (84.0)
1-7 days	45 (15.5)	34 (12.0)	79 (13.8)
More than 7 days	2 (0.7)	9 (3.2)	11 (1.9)
Declined to answer	0 (0.0)	2 (0.7)	2 (0.3)
**Number of pre-treatment smear tests recorded?**			

For peer review only

One	197 (92.1)	169 (87.6)	366 (89.9)
Two	17 (7.9)	24 (12.4)	41 (10.1)
**Number of Gene XPert results recorded**			
One	225 (97.8)	210 (97.2)	435 (97.5)
Two	5 (2.2)	6 (2.8)	11 (2.5)
**Number of culture results recorded on the TB Treatment record**			
One	54 (94.7)	42 (97.7)	96 (96.0)
Two	3 (5.3)	1 (2.3)	4 (4.0)
**Co-morbidities**			
Hypertension	19 (6.93)	11 (4.1)	30 (5.54)
Diabetes	5 (1.84)	4 (1.49)	9 (1.66)
Epilepsy	3 (1.09)	4 (1.49)	7 (1.29)
Mental illness	3 (1.09)	0 (0)	3 (0.55)
Liver disease	1 (0.36)	1 (0.38)	2 (0.37)
Renal insufficiency	1 (0.36)	1 (0.38)	2 (0.37)
Allergies	2 (0.76)	0 (0)	2 (0.38)
Other	1 (0.36)	1 (0.38)	2 (0.37)
**Depression score (CESD 10): mean (SD)**	8.44 (4.38)	8.74 (4.8)	8.59 (4.59)

*Counts and percentages unless otherwise indicated

For peer review only

Supplementary table 2 Detailed descriptive statistics for smoking history, alcohol history at baseline by study arm

	Control n (%)*	Intervention n (%)*	Total n (%)*
Smoking History (current smokers only) N=	181	191	372
**On the days that you smoke, how soon after you wake up do you have your first cigarette?**			
After 60 minutes	30 (16.6)	28 (14.7)	58 (15.6)
31-60 minutes	24 (13.3)	16 (8.4)	40 (10.8)
6- 30 minutes	60 (33.1)	65 (34.0)	125 (33.6)
Within 5 minutes	67 (37.0)	82 (42.9)	149 (40.1)
**Duration of smoking in months: mean (SD)**	212.09 (134.03)	224.93 (127.82)	218.68 (130.86)
**Duration of smoking in months: median (IQR)**	186 (110, 282)	206 (135, 294)	200.5 (123, 287)
**Age started smoking in years: mean (SD)**	19.2 (6.3)	19.3 (6.3)	19.3 (6.3)
**Age started smoking in years: median (IQR)**	18 (15-20)	18 (16-21)	18 (15.5-20.5)
**Form of tobacco used**			
**Manufactured cigarettes (Yes)**	166 (91.7)	179 (93.7)	345 (92.7)
Number of days you smoked in the past 7days: mean (SD)	5.3 (2.65)	5.53 (2.33)	5.42 (2.49)
Average number of cigarettes smoked daily: mean (SD)	6.18 (6.43)	6.48 (8.21)	6.34 (7.39)
**Hand-rolled cigarettes (Yes)**	14 (7.7)	21 (11.0)	35 (9.4)
Number of days you smoked in the past 7days: mean (SD)	4.71 (2.84)	3.71 (2.95)	4.11 (2.91)
Average number of handrolled cigarettes smoked daily: mean (SD)	3.71 (3.97)	3.81 (2.82)	3.77 (3.27)
**Pipe (Yes)**	4 (2.2)	2 (1.0)	6 (1.6)
Number of days in the past 7days you smoked: median (IQR)	1 (0, 2.5)	4.5 (2, 7)	2 (0, 3)
Average number of daily sessions: median (IQR)	1 (0, 3.5)	3 (1, 5)	1.5 (0, 5)
Length of one session (on average) in **minutes**: median (IQR)	90 (60, 107.5)	60 (30, 90)	90 (30, 90)
**Cigars, cheroots or cigarillos (Yes)**	1 (0.55)	0 (0)	1(0.27)
**Water pipe (Yes)**	3 (1.7)	2 (1.0)	5 (1.3)
**Other**	10 (5.5)	4 (2.1)	14 (3.8)
**Heaviness of smoking index >= 4**	134(74.03)	158(82.72)	292(78.49)
Smoking inside your home restrictions			
Total: Not allowed	96 (53.0)	108 (56.5)	204 (54.8)
Some rules: where/when it is allowed	61 (33.7)	58 (30.4)	119 (32.0)
No rules	24 (13.3)	25 (13.1)	49 (13.2)

Attempts to quit smoking (current smokers only)			
**Ever attempted to quit in the past (Yes)**	52 (28.7)	64 (33.5)	116 (31.2)
Number of attempts to quit: mean (SD)	2.46 (2.98)	2.64 (1.62)	2.56 (2.32)
Time elapsed since attempt to quit last time, in months: mean (SD)	36.65 (94.62)	25.13 (37.91)	30.29 (69.22)
Longest duration abstinent in previous quit attempts: mean (SD)	6.15 (13.48)	4.22 (8.9)	5.09 (11.18)
**Likelihood to TRY TO QUIT smoking completely and permanently in the next three months**			
Definitely will not	6 (3.3)	5 (2.6)	11 (3.0)
Probably will not	10 (5.5)	12 (6.3)	22 (5.9)
Probably will	104 (57.5)	109 (57.1)	213 (57.3)
Definitely will	61 (33.7)	65 (34.0)	126 (33.9)
**Likelihood that I WILL QUIT smoking completely and permanently in the next three months**			
Definitely will not	6 (3.3)	5 (2.6)	11 (3.0)
Probably will not	11 (6.1)	13 (6.8)	24 (6.5)
Probably will	103 (56.9)	104 (54.5)	207 (55.6)
Definitely will	61 (33.7)	69 (36.1)	130 (34.9)
**Ever used any methods to help you stop smoking tobacco in the past 3-months?** (Yes)	23 (12.7)	16 (8.4)	39 (10.5)
Smokeless tobacco use (all participants)			
**In the past month, have you used smokeless tobacco (Snuff) on a daily basis**			
Not at all	275 (94.5)	275 (97.2)	550 (95.8)
Daily	12 (4.1)	6 (2.1)	18 (3.1)
Less than Daily	4 (1.4)	2 (0.7)	6 (1.0)
**Duration of using ST in months: mean(SD)**	113.69 (112.7)	152 (91.16)	126.46 (105.61)
**Age started using ST in years: mean (SD)**	27.56 (10.57)	25.88 (13.43)	27 (11.33)
**Form of ST used (for SLT users)**			
Snuff (by mouth)	2 (12.5)	0 (0.0)	2 (8.3)
Snuff (by nose)	11 (68.8)	5 (62.5)	16 (66.7)
Chewing tobacco leaves	0(0)	0(0)	0(0)
Other	1 (6.3)	0 (0.0)	1 (4.2)
**Help to stop drinking (N= , had a drink in past 12 months)**	208	223	431
**Ever used any methods to stop drinking alcohol in the past 3-months**	21 (11.2)	22 (10.7)	43 (10.9)

* Frequencies and (percentages) are presented unless otherwise stated

Supplementary table 3 Detailed descriptive statistics for primary outcome by study arm at 6-months

TB treatment status detailed	Control<br>n (%)	Intervention<br>n (%)	Total<br>n (%)
Cured	108 (37.1)	105 (37.1)	213 (37.1)
Treatment completed	96 (33.0)	87 (30.7)	183 (31.9)
Treatment interrupted > 2 months	15 (5.2)	29 (10.2)	44 (7.7)
Treatment failure	5 (1.7)	2 (0.7)	7 (1.2)
Acquired drug resistance	1 (0.3)	4 (1.4)	5 (0.87)
Died	11 (3.8)	15 (5.3)	26 (4.5)
Transfer out	8 (2.75)	11 (3.9)	19 (3.31)
Unknown	42 (14.4)	25 (8.8)	67 (11.7)

For peer review only

Supplementary table 4 MI treatment fidelity scores of counselling sessions delivered by 17 lay health workers*

Ratings	Mean (SD)	Range
**Global ratings****		
**Cultivating change talk**	3.2 (1.17)	1-5
**Softening sustain talk**	3.4 (0.96)	1-4
**Partnership**	3.4 (1.12)	2-5
**Empathy**	2.9 (1.34)	1-5
**Behaviour counts[&]**		
**Giving information**	4.8 (8.13)	0-5
**Persuade**	0.8 (1.48)	0-5
**Persuade with permission**	0.1 (0.24)	0-1
**Question**	24.2 (10.42)	12-51
**Simple reflection**	4.1 (3.21)	0-10
**Complex reflection**	1.6 (2.69)	0-9
**Affirm**	5.5 (3.47)	1-12
**Seeking collaboration**	2.4 (1.46)	0-6
**Emphasising autonomy**	1.4 (1.37)	0-4
**Confront**	0.4 (0.74)	0-3
**Summary measures**		
**Total MI non-adherent †**	1.2 (2.28)	0-7
**Total MI adherent†**	9.3 (4.74)	1-16
**Technical global ratings****	3.3 (0.97)	1.5-4.5
**Relational global ratings****	3.1 (1.19)	1.5-5
**Reflection to question ratio††**	0.23 (0.24)	0-0.83
**Percentage of complex reflections*****	20.4 (21.9)	0-67

*The recordings of 17 counsellors (one each) were transcribed verbatim and then assessed. In order to assess the fidelity of the counsellors' delivery of motivational interviewing during the trial, we are used the validated Motivational Interviewing Treatment Integrity Coding Manual 4.2.1 (MITI) tool. The table shows the results of the fidelity assessment that was conducted by one rater who is proficient in English, seSotho and seTswana and in motivational interviewing. The rater listened to the recordings and coded a randomly selected 20-min portion of the written transcript. In the case of shorter counselling sessions, the entire recording was assessed.

**The "global ratings" involve assessing, on a 5-point Likert scale (1 for low and 5 for high), how well or poorly the counsellor adheres to the MI practice. Ratings are conducted on four items, two each making up Technical Components (Cultivating change talk and Softening sustain talk) and Relational Components (Partnership and Empathy).

**Scores on Cultivating Change Talk and Softening Sustain Talk are averaged to obtain the Technical global scores.

***Scores on Partnership and Empathy are averaged to obtain Relational global scores. The basic competency threshold scores for fair and good proficiency are 3 and 4, respectively for Technical scores and 3.5 and 4, respectively for Relational scores.

[&]The "behaviour counts" involve counting 10 verbal behaviours of the counsellor during the intervention.

† "MI adherence" is determined by adding up the following verbal behaviours: Seeking Collaboration, Affirm and Emphasising autonomy. "MI non-adherence" is determined by summing instances of Confront and Persuade. No thresholds for MI adherence or non-adherence are specified in the MITI 4.2.1.

††The Reflection-to-Question (R:Q) ratio is the total reflections divided by the total questions asked. One reflection to each question is considered a "fair" practice level while two reflections to each question is considered a "good" practice level.

*** The Percentage of Complex Reflections (% CRs) is calculated by dividing the number of complex reflections by the sum of complex reflections and simple reflections. A fair and a good % CRs are 40% and 50%, respectively.

For peer review only

Supplementary table 5 SMS delivery

	Intervention (N=283)		Control (N=291)	
No. of participants who received ALL due IMB messages INDEPENDENT OF WHETHER they completed MI 1	99/283 (35%)		1/291 (0.3%)	
**Completion of first MI and initiation of SMS-sequence**				
Completed first MI	227/283 (80.2%)		0/291 (0%)	
No. of participants who received ALL due messages after receipt of MI1	95/227 (41.9%)		0/0 (0%)	
**SMS delivery for participants for whom the SMS-sequence was initiated (after receipt of first MI)**				
	Mean (SD)	Median (IQR) Range	Mean (SD)	Median (IQR) Range
Average no. adherence messages received per participant (n=227)	7.9 (3.5)	10 (8-10) 0-11	0	0
Average no. tobacco-related messages received (n=153)	5.4 (2.5)	7 (5-7) 0-7	0	0
Average no. alcohol related messages received (n=171)	5.5 (2.5)	7 (5-7) 0-7	0	0
Average no. IMB messages received (n= 227)	15.7 (7.3)	17 (13-22) 0-25		

For peer review only - http://bmjopen.bmj.com/site/about/guidelines.xhtml

**Supplementary table 6** Unit costs used in the analysis (Presented in Rand as sources, $1=R14.448 in 2019)	
**Item**	Unit costs
**Personnel**	LHWs: R3000/month, 160 hours/month, R18.75/hour; District coordinators: R7500/month, 160 hours/month, R46.88/hour; MRC appointed supervisor: R205.79/hour; Trainers: R373.17/hour; Administrative staff: R333.24/hour
**Materials**	Training manual: R3250; Printing: R3000; Additional printing & stationary: R500
**Accommodation / Travel / Refreshments**	LHWs accommodation: R540 000; Trainers accommodation/travel: R36 000; Supervision accommodation/travel: R30 000; Refreshment: R12 500
**SMS system**	Monthly subscription at R433.44 ($30) for 3-months: R1300
**MI sessions**	Session 1 (17 minutes): R5.31; Session 2 (16 minutes): R5.00; Session 3 (17 minutes): R5.31
**Biochemical investigations**	Cost of TB smear microscopy: R28.37; culture: R79.22 and GeneXpert: R201.56 (Source: 2019/20 National Health Laboratory Service pricing schedule)*
**ART**	Atroiza: R3.78/dose; Dumiva: R5.55/dose; Tenemine: R2.34/dose; Zovilam: R1.72/dose; Kavimun: R1.89/dose; Ricovir: R1.36/dose; Zidomat: R1.38/dose; Lazena: R0.55/dose; Efrin:R0.63/dose; Efamat: R0.63/dose; Acriptaz: R0.61/dose (Source: Western Cape Department of Health. Antiretroviral and TB Stockmaster Worksheet. 2019)
**TB medication**	Month’s supply of RHZE (intensive) @ R65.80; RH (continuation) @ R55.56 (Source: Western Cape Department of Health. Antiretroviral and TB Stockmaster Worksheet. 2019)

For peer review only

PROLIFE

Im*pro*ving TB outcomes by modifying *life*-style behaviours through a brief motivational intervention followed by short text messages (Phase II)

A multi-centre randomised controlled trial looking at the effect of a complex behavioural intervention on TB and lifestyle-related outcomes in South Africa

Version 1.2

Trial Registration number/registry: ISRCTN62728852/ISRCTN registry
http://www.isrctn.com/ISRCTN62728852

Version date: ~~14 Feb 2020~~ 25 Oct 2020

Author(s): Mona Kanaan

Department of Health Sciences
University of York
York, YO10 5DD

Principal Investigator: Olalekan Ayo-Yusuf and Kamran Siddiqi
Trial Coordinator: Goedele M Louwagie

**Sponsor**	**Olalekan Ayo-Yusuf, Sefako Makgatho University/ University of Pretoria**
**Funder**	**Newton Fund/MRC**
**Funder Reference Number**	**MRC-RFA-02: TB -05-2015**

For peer review only - http://bmjopen.bmj.com/site/about/guidelines.xhtml

STATISTICAL ANALYSIS PLAN
V1.2

Contents

1. Definition of terms 4
2. Trial Objectives 5
3. Design 6
4. Sample Size 7
5. Randomisation 7
6. Outcomes 8
6.1 Primary outcome 8
6.2 Secondary outcomes 9
6.2.1 Monitoring adverse events 9
6.3 Trial assessment schedule 11
6.4 Other important information 12
6.5 Fidelity of the intervention 12
7. Data 13
7.1 Data collection methods 13
7.2 Data management 16
8. Analysis 17
8.1 Participants Flow 18
8.2 Baseline data 19
8.3 Primary analysis 24
8.3.1 Primary outcome definition: 24
8.3.2 Primary outcome Analysis 24
8.3.3 Primary outcome Analysis Results 25
8.4 Secondary analyses 26
8.4.1 Secondary outcome definitions that involve defining a positive baseline test: 27
8.4.2 Continuous Abstinence 29
8.4.4 Change in harmful or hazardous drinking at month 3 and 6 follow-ups 32
8.4.5 Proportion of ART uptake of HIV-positive participants at month 3 and 6 follow-ups . 34
8.5 Subgroup analyses (See above analyses) 35
8.5 Sensitivity analyses 35
8.6 Adverse events 36
8.7 Planned interim review and analyses 36

For peer review only - http://bmjopen.bmj.com/site/about/guidelines.xhtml

8.8 List of Tables and Graphs 37
9. SAP amendment log 38
10. Signatures of approval 38
11. References 39
12. Appendix A (Results tables)........ 40
Table 1: 40
13. Appendix B (Scoring notes +) .... **Error! Bookmark not defined.**
13. Additional descriptive Statistics 65

This analysis plan deals only with the statistical analysis of the trial.

For peer review only

For peer review only - http://bmjopen.bmj.com/site/about/guidelines.xhtml

1. Definition of terms

**Abbreviation**	**Expansion**
ACTG	AIDS Clinical Trials Group
AE	Adverse Event
ART	Anti-retroviral therapy
AUDIT	Alcohol Use Disorders Identification Test
CI	Confidence Interval
CO	Carbon monoxide
CONSORT	Consolidated Standards of Reporting Trials
CRF	Case Report Form
EPTB	Extra-pulmonary TB
HIV	Human immunodeficiency virus
LHW	Lay health worker
MI	Motivational interviewing
PTB	Pulmonary TB
SMS	Short Message Service
SA	South Africa
SADHS	South Africa Demographic and Health Survey
SAE	Serious Adverse Event
SD	Standard deviation
SOPs	Standard Operating Procedures
TB	Tuberculosis

For peer review only

For peer review only - http://bmjopen.bmj.com/site/about/guidelines.xhtml

2. Trial Objectives

The PROLIFE model is a complex behavioural intervention comprised of a brief motivational interviewing (MI) counselling strategy augmented with subsequent SMS messaging. To be delivered in three brief sessions, the MI intervention will target three main areas, as appropriate:

- Tobacco smoking
- Alcohol drinking
- Tuberculosis (TB) and Anti-retroviral therapy (ART) adherence or ART initiation

Primary objective:

- To assess the effectiveness of the PROLIFE model delivered by lay health workers (LWH) compared to usual care in improving Pulmonary TB (PTB) treatment outcomes

Secondary objective: (**this element will not be addressed in this SAP**)

- To estimate the cost-effectiveness of the PROLIFE model

For peer review only - http://bmjopen.bmj.com/site/about/guidelines.xhtml

3. Design

This is a pragmatic, prospective, multicentre, two-arm, parallel, individual RCT taking place in 27 purposively selected primary care clinics with the highest TB case-load in three districts in South Africa: Welkom in the Free State; Bojanala in the North West province; and Sedibeng in Gauteng province. The intervention will be delivered by LHWs and three district coordinators who will each cover 1–2 clinics.

This is a pragmatic parallel superiority individually randomised controlled trial. There are two treatment arms:

The control arm (Arm1): Intervention arm – participants will receive the PROLIFE programme;

The intervention arm (Arm2): Control arm – participants will receive usual treatment and support provided to TB patients in TB treatment clinics in South Africa ('usual care').

Full details of the background and design of the trial are presented in the protocol (version 1.2 Prolife Protocol_15 Dec 17_with markup) and the published protocol in Moriarty et al (2019) https://doi.org/10.1186/s13063-019-3551-9.

Participants

The inclusion criteria for participants are:

- adult patients (aged ≥ 18 years)
    - with drug-sensitive (bacteriologically or clinically confirmed) PTB;
- initiating TB treatment or on TB treatment for < 1 month (these include both 'new' and 'retreatment' patients);
- current smokers and/or
- hazardous/harmful drinkers who are not alcohol dependent (Alcohol Use Disorders Identification Test [AUDIT] score ≥ 8 for men or ≥ 7 for women but < 20);
- access to a functional mobile phone; and
- understand one of the four languages used for the trial (Sesotho, Setswana, Isizulu or English).

Exclusion criteria:

- alcohol-dependent participants (AUDIT score ≥ 20);
- Extrapulmonary TB without PTB; or
- Resistance to one or more TB drugs at baseline

For peer review only - http://bmjopen.bmj.com/site/about/guidelines.xhtml

4. Sample Size

We will recruit 696 participants (348 per study arm). The sample size calculations were based on the following assumptions:

- Detection a 10% difference in TB treatment success rates (0.86 vs 0.76) in the ProLife group versus the control group
- 80% power,
- a significance level of 0.05, and
- 25% attrition.

The sample size per clinic was in the range of 14–74 participants per clinic with a median of 24. The assumed success rates in the control group are based on actual success rates in TB patients in the studied provinces that were available at the time of sample size calculations in 2015.

5. Randomisation

Patients will be randomised using a randomised sequence generator performed by the trial statistician (MK) who will remain blind to the arm allocation. We will use block randomisation with varying block sizes stratified by the clinic to achieve equal numbers in intervention and control groups within each clinic. Allocation concealment will be done with consecutively numbered, sealed, opaque envelopes.

Lay health workers delivering the intervention, field researchers, and participants cannot be blinded to the intervention. However, the determination of the primary outcome will be completed by TB nurses who are blinded to the intervention status of the participants based on routinely collected data.

The statistician will be blinded to the intervention or control arm allocation of participants during the analysis stage.

For peer review only - http://bmjopen.bmj.com/site/about/guidelines.xhtml

6. Outcomes

6.1 Primary outcome

The primary outcome is TB treatment success at six to nine months of follow-up. This is a binary variable defined as

- Success: cured or treatment completed
- Failure: failed treatment, death, acquired drug resistance, loss to follow-up or 'default', or not outcome evaluated.

The different mutually exclusive treatment outcomes are summarised here

Treatment outcome	Definition
Cure	Patient in whom baseline smear or culture was positive at beginning of treatment AND is smear/culture negative in the last month of treatment and on at least one previous occasion at least 30 days prior<br>According to local protocol, a patient who is diagnosed using Gene Xpert and is sputum negative for TB at 11 and 23 weeks is considered 'Cured'.
Treatment completed	Patient whose baseline smear or culture was positive at the beginning and has completed treatment but does not have a negative smear/culture in the last month of treatment and on at least one previous occasion > 30 days prior. Patients diagnosed with PTB whose baseline smear (or culture) result was negative and who started treatment based on clinical and radiological findings who have shown clinical improvement and completed the prescribed course of treatment.<br>*N.B. The smear examination may not have been done or the results may not be available at the end of treatment.*
Treatment failure	Patient whose baseline smear or culture was positive and remains or becomes positive again at 5 months or later during treatment.<br>Patients who were negative at baseline but were later found to be positive.<br>*N.B. This definition excludes those patients who are diagnosed with RR-TB or MDR-TB during treatment.*
Died	Patient who dies for any reason during the course of TB treatment.
Treatment default	Patient whose treatment was interrupted for two consecutive months or more during the treatment period.
Transfer out	Patient who was referred to a facility in another district to continue treatment and for whom the treatment outcome is not known.
Acquired resistance	Participants who are subsequently referred for MDR treatment.

For peer review only - http://bmjopen.bmj.com/site/about/guidelines.xhtml

6.2 Secondary outcomes

The following outcome measures will be recorded at the six-month follow-up:

- sputum conversion at the end of treatment in the group of participants who had bacteriology confirmed PTB at baseline [1]
- continuous smoking abstinence for identified smokers at baseline[2]

Whereas, the following will be assessed at three and six months follow-up:

- reduction in harmful or hazardous drinking[3]
- TB and ART medication adherence will be measured using a modified version of the AIDS Clinical Trials Group (ACTG) Adherence Questionnaire[4]; using an adherence index calculated by the formula (using the four-day recall table):

  [Total number of doses taken/Total number of doses prescribed] x 100

  Patients with at least 95% of adherence will be considered as having optimal adherence otherwise will be considered as having low (or suboptimal) adherence.
- increase in proportion of HIV-positive participants on ART at three and six months from baseline using standardised questions on the CRF.

6.2.1 Monitoring adverse events

Adverse events (AE) and serious adverse events (SAE) will be defined *apriori* and relevant information will be collected.

A **Serious Adverse Event (SAE)** is actually a special case of an adverse event where adverse outcomes are severe. It includes following events: Death of any of the participants associated with a clinical trial. **Examples of events**: Death, a life-threatening adverse event, inpatient hospitalization or prolongation of existing hospitalization, a persistent or significant.

An **adverse event (AE)** is any untoward medical occurrence in a patient or clinical investigation subject administered a pharmaceutical product and which does not necessarily have a causal relationship with this treatment.

The events are reported to ethics committee within 72 hours

[1] i.e. cure rates in intervention group versus control group for participants who initially had sputum AFB-positive, culture-positive or GeneXpert-positive PTB

[2] defined as a self-report of not smoking > 5 cigarettes six months from the start of the abstinence period, supported by a negative biochemical test CO < 7 ppm

[3] alcohol use will be measured using the AUDIT questionnaire. The questionnaire will be administered at screening (which will take place on the same day or shortly after the baseline assessment) and again at three months and six months.

[4] The questionnaire is a validated tool for measuring adherence specifically to ART and we will use an adapted version to also measure TB medication adherence [40].

For peer review only - http://bmjopen.bmj.com/site/about/guidelines.xhtml

The following information were collected to report the events:

Participants identification number, Gender, Age, Date of Enrollment, Arm (Control or intervention), Date of death notification to staff, Date of death , If death is related or not related to study.

For peer review only

For peer review only - http://bmjopen.bmj.com/site/about/guidelines.xhtml

6.3 Trial assessment schedule

Table 2 details the trial assessment schedule

Table 2: *Trial assessment schedule*

Assessment	*Items*	**Pre randomisation**	**Timeline (post randomisation)**				
		Eligibility (Day 0)	***Baseline (Day 0)***	***2 months***	***3 months***	***6-9 months***	***Trial end***
**ELIGIBILITY**							
Smoking status		X					
Smoking profile			X				
Alcohol profile		X					
Medical eligibility		X					
Eligible, consenting		X					
**MEASURES**							
Trial ID, visit date			X				
Socio-demographic history			X				
Depression screen			X		X	X	
Clinical review of TB Treatment record for disease information			X				X
Smoking history			X				
Smoking abstinence (self-report)					X	X	
Exhaled CO					X	X	
Record sputum culture or smear or Gene Expert result			X	X	X	X	
HIV Status			X				
ART Status (if HIV positive)			X		X	X	
AUDIT		X			X	X	
Modified ACTG (Follow up)					X	X	
Economic evaluation			X		X	X	

For peer review only - http://bmjopen.bmj.com/site/about/guidelines.xhtml

6.4 Other important information

In addition to the above, the following information was collected at baseline. Socio-demographic history included age, gender, marital status, education, employment status, and comorbidities. For details about these variables, see Table 3.

6.5 Fidelity of the intervention

The main fidelity analysis will be published somewhere else. Some descriptive statistics regarding fidelity will be added here once the main analysis is completed and the statistician is unblinded to the treatment arm.

For peer review only - http://bmjopen.bmj.com/site/about/guidelines.xhtml

7. Data

7.1 Data collection methods

The fieldworkers will screen all TB patients for eligibility immediately after the TB nurse at the clinic has initiated TB treatment and opened the TB "blue card". Consent will be obtained for this screening phase as the alcohol related questions are sensitive and the fieldworkers must gain insight in the patient files.

Eligible and consenting patients will be enrolled in the trial and the baseline questionnaire and record review completed. Patients will be given a unique Trial Number that will be used on all research documents. Data will be collected and recorded by field workers equipped with Android phones with a mobile data collection application installed.

Participants in the control arm will continue with the routine TB care. Intervention arm study participants will be referred by the fieldworker to the lay counsellor for motivational interviewing. The first MI session will be on the same day of the completion of the screening questionnaire, where possible (with a 2-week window period). The second and third MI session will be scheduled 4 weeks and 8 weeks from the first counselling session respectively each time with a 2-week window period.

MI counselling and data collection will take place in a well-ventilated private area inside or outside the clinic, and audio-recorded after consent obtained. Fieldworkers and LHWs will be provided with high particulate respirator masks to minimise the risk of infection.

Fieldworkers will follow-up all participants in both arms at 3 and 6 months within a window period of 2-weeks before and 2-weeks after the ideal 3 and 6-month visit. Participants will receive SMS reminders 3 days before each planned visit. Participants will also be in a position to send "please call me "messages to the fieldworkers or district coordinators, who will then call the participant to solve problems that may have arisen with the appointment.

Patients who did not return for the planned 3 and 6 months visit will be contacted by telephone up to 3-times, as needed. Home visits will also be undertaken by existing clinic "tracer teams" or Community Based Outreach teams -where feasible - for participants who cannot be traced by telephone. The data collection process is illustrated in Table 3.

For peer review only - http://bmjopen.bmj.com/site/about/guidelines.xhtml

Table 3: Data Collection Process by time-point and details of data collection method		
**Time-point**	**Information required at time-point**	**Data collection method**
**Baseline interview**	**1. <u>Socioeconomic and demographic status (to include history of mine work)</u>**<br>• Age<br>• Gender<br>• Marital status<br>• Educational level achieved<br>• Employment<br>• Mine work/type of mine work<br><br>2. Clinical information:<br>• Patient category (First episode vs recurrence)<br>• Site of disease<br>• Results of sputum smear, culture and Gene Xpert<br>• HIV status<br>• ART information<br>• Co-morbidities<br><br>3. Current smoking status and quit history, second hand smoke exposure<br><br>4. Alcohol history<br>5. Depression	TB Treatment Record<br><br>CRF – Questions to participant – demographic details<br><br>CRF – Questions to participant and information from the TB Treatment Record (as indicated in CRF)<br><br>Questions adapted from Global Adult Tobacco Survey Questionnaire<br><br>Baseline AUDIT score<br>CES-D
**3 months**	1. ART information<br><br>2. TB and ART medication adherence (modified ACTCG)<br><br>3. Alcohol history (repeat AUDIT)<br><br>4. Smoking history<br>5. SLT use	CRF – Question to patient<br><br>ACTG questionnaire for both TB medication and ART<br><br>AUDIT score at 3 month

For peer review only

For peer review only - http://bmjopen.bmj.com/site/about/guidelines.xhtml

	6. Depression	Follow-up questions as per Russell's Standard and exhaled CO<br><br>CES-D
**6 months**	1. TB treatment status <u>(Primary outcome)</u><br><br>2. Sputum smear or culture result<br><br>3. ART information<br><br>4. TB and ART medication adherence<br><br>5. Alcohol history (repeat AUDIT)<br><br>6. Smoking history and exhaled CO<br><br>7. Depression	TB treatment outcome from TB Treatment Record combined with information from TB record on cultures and smear results.<br><br>CRF- Question to patient<br><br>Follow-up ACTG questionnaire at 6 months<br><br>AUDIT at 6 months<br><br>Questions as per Russell's Standard and exhaled CO<br><br>CES-D

For peer review only - http://bmjopen.bmj.com/site/about/guidelines.xhtml

7.2 Data management

Sefako Makgatho University (SMU) appointed a data manager who will utilise an electronic platform for data collection, ensuring data quality, and facilitating the SMS messages.

Fieldworkers collecting research data will be equipped with Android mobile phones, which will have a mobile application installed on them to allow for data collection in areas with poor internet connectivity. The electronic data captured will be stored on secure and password protected storage servers and mobile phones, which ensure data privacy through only allowing authorised research staff access to the data.

The electronic data collection system used for the study requires an SMS gateway to send and receive messages to the research participants. Consenting participants' phone numbers, participant IDs, and associated SMS messages will be stored on the SMS gateway's secured and password-protected server.

Data quality will be ensured by providing fieldworkers with standard operating procedures (SOPs), training, and ongoing support on the importance of data quality, data collection, and data collection problem-solving. The data manager will continuously monitor the captured data for missing variables and inconsistencies in order to resolve any data problems.

The data manager will export the data from the secured server, conceal the participants study arm allocation, and de-identify the data before sharing the data in STATA and R compatible formats. The exported de-identified data will be stored in Dropbox, a secure cloud storage platform, for sharing with the lead trial statistician at the University of York for analysis.

All research data and documents referring to the PROLIFE trial will be stored and maintained in a secured storage space at SMU for a minimum of 15 years from the end of the PROLIFE trial. Study materials will be destroyed 15 years after the study.

For peer review only

8. Analysis

The computer packages STATA 16 (StataCorp. 2019) and R 3.5.3 (ref) will be used. Significance tests will be two-sided and the significance level is set at 0.05. The statistician will remain blind to allocation until results are finalised. We will follow the CONSORT statement guidelines in reporting.

Below, we detail the analyses that we will carry out for the data collected at baseline, the primary outcome, the secondary outcomes, and adverse events. We also list the sensitivity analyses that we might perform and subgroup analyses.

For peer review only

For peer review only - http://bmjopen.bmj.com/site/about/guidelines.xhtml

8.1 Participants Flow

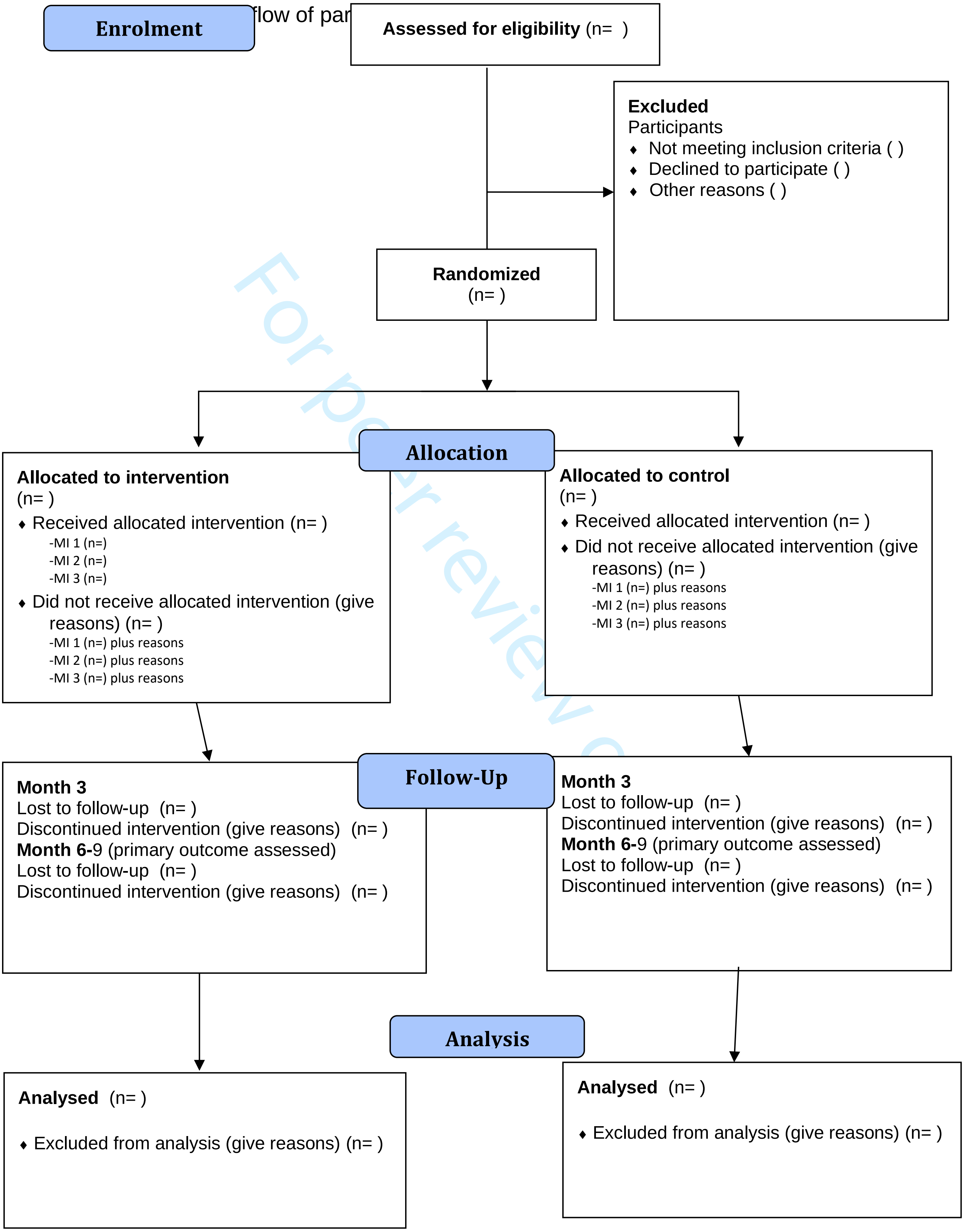

For peer review only - http://bmjopen.bmj.com/site/about/guidelines.xhtml

Figure 1 CONSORT DIAGRAM SHOWING FLOW OF PARTICIPANTS

8.2 Baseline data

Baseline data Analysis Plan:

Baseline data including demographic variables will be summarised descriptively by trial arm, but no formal statistical comparisons will be undertaken. Continuous measures will be reported as means and standard deviations (SD) while categorical data will be reported as counts and percentages, see Tables 1 to 4 in Appendix A. For skewed continuous measures, we will also provide medians and interquartile ranges.

Baseline data results:

Consent:

In the control arm (Arm1) 286 gave written consent and 5 verbal consent for participation in the study and for access to their medical records

In the intervention arm (Arm2) 279 gave written consent and 4 verbal consent for participation in the study and for access to their medical records

Baseline Imbalances

Education:

It seems that for education there is imbalance between the two arms for Grades 8-11/Grade 12/Higher; the control arm (Arm1) had a higher educated group the difference in percentage points 8.2% as opposed to 2.8% in the intervention arm, see Table 2 for further details. There are 9 participants who are not literate in the study (7 in the control arm (Arm1) and 5 in the intervention arm (Arm2)).

Drinking

In the intervention arm, 223 (78.8%) had a drink in the past 12-months compared to 208 (71.5%) in the control arm, see Table 3.

In the control arm (Arm1), 110 (37.8%) were drinkers only, 83 (28.5%) were smokers only, and 98 (33.7%) were smokers and drinkers compared to 92 (32.5%), 60 (21.2%), and 131 (46.3%) in the intervention arm (Arm2), respectively.

For peer review only - http://bmjopen.bmj.com/site/about/guidelines.xhtml

Assets:

The vast majority had a radio (84%), a television (89%), a refrigerator (85%), an electric or gas stove (86%), and a microwave (67%), however, only 50% had a washing machine. Furthermore, a minority had a landline telephone (7.5%), a desktop or laptop computer (20%:), and a vacuum cleaner or floor polisher (15%). Figure 2 is a boxplot of the total number of assets by study arm. The spread in the control arm (Arm1) is greater than that in the intervention arm (Arm2). However, the mean number of assets is similar across the two groups of 5 (SD: 1.87).

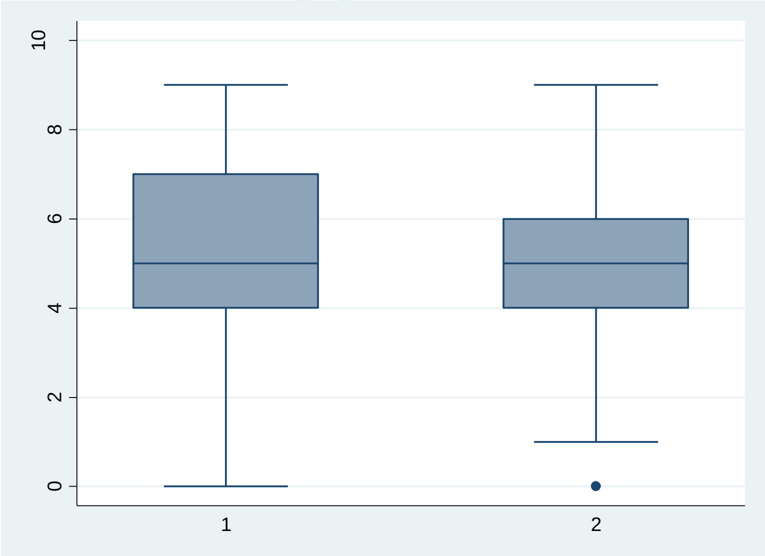

Figure 2: A boxplot of the total number of assets by study arm

For peer review only - http://bmjopen.bmj.com/site/about/guidelines.xhtml

Medical History:

There were 305 (53.2%) **HIV positive** participants out of which 204 (65.38%) were using Cotrimoxazole and 257 (82.37%) were undergoing anti-retroviral therapy, see Table 2.

TB History: The vast majority of participants were New TB patients 513(91.3%), with Pulmonary only (ICD-10 A15) being the site of disease for the vast majority 553 (98.9%). Among those with results known, the majority had one pre-treatment smear result 366 (89.9%), one Gene XPert recorded 435 (97.5%), and one culture result recorded 96 (96%).

Among those with results available, 220 (58.51%) had at least **one positive smear** result,362 (87.23%) had at least **one positive Gene XPert result**, and 35 (47.95%) at least **one positive culture result**.

However, 85 (53.46%) in the control arm had at least one positive smear result within 60 days of the TB treatment start date compared to 96 (61.15%) in the intervention arm.

In addition, only 34 had their culture results within 60 days from the TB start dates of which 21 had positive results; 11 in the control arm compared to 10 in the intervention arm.

The vast majority of participants did not have any **co-morbidities** 525 (96.3%) and 18 (3.3%) had one.

For peer review only

Baseline Smoking Related variables:

Thirty five percent (202) did not smoke in the past month, whereas 52% smoked daily and the remaining 13% smoked in the past month but less frequently than on a daily basis, see Table 3.

In the past 30 days, the vast majority were exposed to smoke for seven days or less at: home 462 (80.5%); cafes/restaurants 464 (80.8%); Shebeens, bars or clubs 432 (75.3%); Bus/train/taxi/ vehicle 448 (78.0%); and Shops/shopping mall 461 (80.3%) and where applicable at the workplace 173 (66.8%).

Among those who smoked in the past month 372: 181 in the control arm (Arm1) vs 191 in the intervention arm (Arm2),

345 (92.7%) **smoked manufactured cigarettes** of which 225(65%) did so on a daily basis in the past seven days whereas 40 (11.6%) did not smoke in the past week. The mean number of days smoked was 5.42 days in the past week (SD: 2.49) with 6.34 cigarettes (SD: 7.39) smoked daily on average.

Hand-Rolled cigarettes were used by 35 participants of which 16 (45.7%) smoked daily in the past week. They smoked on average for 4.11 (SD:2.91) days in the past week and on average smoked 3.77 (SD: 3.27) hand-rolled cigarettes per day.

There was only one person who exclusively smoked waterpipe, two who exclusively smoked pipe, and seven who exclusively used other formats of tobacco other than the ones that are listed here.

For the vast majority the total number of cigarettes smoked is based on their answer to manufactured cigarettes. In 27 cases, they supplemented this with other sources and 8 used only other forms to report the number smoked on average per day.

Over the past 3 months, they **spent** on average 174.58 (SD: 181.61) Rands per week on tobacco products.

For peer review only - http://bmjopen.bmj.com/site/about/guidelines.xhtml

Smoking restrictions

No smoking was allowed **inside home** for 204(54.8%) participants, whereas 119(32.0%) had some rule where/when it is allowed and 49(13.2%) had no rules in place.

Quit Attempts:

Among smokers, 116 (31.2%) made an **attempt to quit**; the mean number of attempts to quit was 2.56 (SD: 2.32).

Furthermore, 213 (57.3%) said they will probably **try to quit** smoking completely and permanently in the next three months and 126 (33.9%) said that they definitely will.

Whereas, 207 (55.6%) said they will probably **quit** smoking completely and permanently in the next three months and 130 (34.9%) said that they definitely will.

Only 39 (10.5%) have ever used any methods in the past 3 months to help them stop smoking tobacco. These spent on average 136.64 (SD: 205.36) Rands on methods to help you stop smoking in the past 3 months.

Smokeless tobacco was used by only 24 participants (4.2%). They have been using ST for an average of 10.5 years (SD: 8.8) and have started using it at the age of 27 (SD: 11.33) years on average.

Heaviness of smoking:

In the control arm (Arm1), 67(37.0%) of smokers reported smoking within 5 minutes of waking up whereas 82(42.9%) did so in the intervention arm (Arm2).

Among those who smoked and who reported the number of cigarettes/pipes/cigars they used on average per day, 134(74.03%) in the control arm (Arm1) and 158(82.72%) in the intervention arm (Arm2) were considered as heavy smokers.

For peer review only - http://bmjopen.bmj.com/site/about/guidelines.xhtml

8.3 Primary analysis

8.3.1 Primary outcome definition:

As per section 6.1, we would define the primary outcome as those who had a successful treatment versus not. Where successful treatment is considered if the patient is considered to have been cured or treatment completed; all other categories will be deemed as not successful. The successful treatment[5] categories are defined as follows:

Cure	Patient in whom baseline smear or culture was positive at beginning of treatment AND is smear/culture negative in the last month of treatment and on at least one previous occasion at least 30 days prior According to local protocol, a patient who is diagnosed using Gene Xpert and is sputum negative for TB at 11 and 23 weeks is considered 'Cured'.
Treatment completed	Patient whose baseline smear or culture was positive at the beginning and has completed treatment but does not have a negative smear/culture in the last month of treatment and on at least one previous occasion > 30 days prior. Patients diagnosed with PTB whose baseline smear (or culture) result was negative and who started treatment based on clinical and radiological findings who have shown clinical improvement and completed the prescribed course of treatment. *N.B. The smear examination may not have been done or the results may not be available at the end of treatment.*

8.3.2 Primary outcome Analysis

For the primary outcome, we will conduct analysis on an intention-to-treat basis. We will use binary logistic regression to compare the main outcome between the

[5] Following discussion with the team, we will take this at face value as it is not possible to query some of the anomalies found in the recording of dates of tests.

intervention and the usual care arm. We will also investigate any potential clustering at the centre level and account for it. We will present the results for this analysis in Table 6.

We will also adjust for HIV status, sex, alcohol versus tobacco versus both, and district; if these differ between trial arms at baseline.

8.3.3 Primary outcome Analysis Results

For peer review only

Table 4 gives the descriptive statistics for the initial outcome and the derived - dichotomised outcome; these indicate that overall, 69% were classified as successful treatment based on the medical professional assessment (cured/treatment completed). This percentage was similar for the two arms with the control arm (Arm1) having a slightly higher percentage of success of 70.1% compared to 67.8% in the intervention arm (Arm2)[6]. Table 9 gives the distribution per centre by study arm. Generally, these are fairly balanced any imbalance observed is most probably due to the early termination of the study.

For 203 participants, the TB treatment outcome date was not available, with those participants more likely to not have been cured (59% not cured), however, the percentage was similar across the two study arms for those with missing TB treatment outcome date.

The odds of successful treatment is 0.9 (95% CI: (0.64,1.27)) in the intervention arm (Arm2) compared to the control arm (Arm1). This estimate is very similar to the estimate adjusting for district and drinking/smoking status. This is also the case if you further adjust for sex and HIV status at baseline where the OR is 0.86 (95% CI: (0.60,1.24)); see Table 6 for further details.

---

[6] Primary outcome Control: 204/291, 95% CI proportion 0.70 (0.64,0.75)
Primary outcome Intervention:192/283, 95% CI proportion 0.68 (0.62,0.73)

For peer review only - http://bmjopen.bmj.com/site/about/guidelines.xhtml

8.4 Secondary analyses

Secondary analyses plan

In the group of participants who had bacteriology confirmed at baseline, we will use logistic regression to compare cured versus not cured, as indicated by the outcome at the end of treatment, between the two study arms. We will also control for baseline characteristics and other covariates such as sex, alcohol use, HIV-status, district, and account for any potential clustering by centre.

We will use a similar approach for the six-months continuous smoking abstinence outcome. This analysis will be performed on the group of participants who were current tobacco smokers at baseline. We will also control for baseline characteristics and other covariates such as age, duration of smoking, alcohol problem (hazardous, harmful, non-drinker/light drinker), heaviness of smoking index[7], depression, and potentially HIV-status.

For the reduction in harmful or hazardous drinking, we will use linear regression to measure difference in total AUDIT score between control and intervention groups accounting for the baseline AUDIT scores. Separate analyses for the AUDIT at 3 and 6 months will be performed.

The AUDIT is a 10-items questionnaire with a range between 0 and 40 where higher values indicate higher dependency.[8] It is worth mentioning that a score of 8 or more

---

[7] [Goedele's Comment on an earlier Version]Definitely a measure of severity of smoking and duration of smoking. For example Heaviness of Smoking Index which can be derived as follows:

HSI=Heaviness of smoking index ≥ 4, calculated based on sum of time to first cigarette (0: 61+min, 1:31-60 min, 2: 6-30 min, 3: ≤5 min) and number of cigarettes smoked per day (0: 0-10 cigarettes per day [CPD]

Also: age, duration of smoking, alcohol problem (hazardous, harmful, non-drinker/light drinker), maybe HIV-status, (adding this may reduce your sample size too much, because of missing HIV-status, unless you include HIVstatus unknown as a category of HIV-status). Depression

[8] Scoring the audit
Scores for each question range from 0 to 4, with the first response for each question (eg never) scoring 0, the second (eg less than monthly) scoring 1, the third (eg monthly) scoring 2, the fourth (eg weekly) scoring 3, and the last response (eg. daily or almost daily) scoring 4. For questions 9 and 10, which only have three responses, the scoring is 0, 2 and 4 (from left to right).
A score of 8 or more is associated with harmful or hazardous drinking, a score of 13 or more in women, and 15 or more in men, is likely to indicate alcohol dependence.

For peer review only - http://bmjopen.bmj.com/site/about/guidelines.xhtml

is associated with harmful or hazardous drinking, a score of 13 or more in women, and 15 or more in men, is likely to indicate alcohol dependence. Eligibility criteria for our study is an AUDIT score ≥ 8 for men or ≥ 7 for women but <20. However, if assumptions of linear regression are not met we will either transform the data or use alternative regression analyses such as ordinal logistic regression.

Adherence to TB and ART medication will be measured using an adherence index based on a modified version of the AIDS Clinical Trials Group (ACTG) Adherence Questionnaire; where patients with at least 95% of adherence will be considered as having optimal adherence otherwise will be considered as having low (or suboptimal) adherence. We will use logistic regression to model patient's characteristics (age, sex, alcohol, smoking status, depression) that might influence adherence at 6-month; we will also compare adherence between study arms. Similar to the previous outcomes, we will account for any potential clustering by centre.

We will also report the proportion of HIV-positive participants on ART at six months and compare these to the baseline using standardised questions on the CRF.

8.4.1 Secondary outcome definitions that involve defining a positive baseline test:

To operationalise the above we need to define how we determine if someone has a positive baseline smear or culture. Each participant might have up to two tests of the following: smear test, GeneExpert test, and culture test.

If a test was administered two times then to be considered negative both tests should be negative, otherwise it is considered positive. If a test was administered only once, then the result of that instance is taken as is.

1Saunders JB, Aasland OG, Babor TF et al. Development of the alcohol use disorders identification test (AUDIT): WHO collaborative project on early detection of persons with harmful alcohol consumption — II. Addiction 1993, 88: 791–803

For peer review only - http://bmjopen.bmj.com/site/about/guidelines.xhtml

If for a patient more than one test was administered, then to be considered negative the result should be negative under both tests.

This is illustrated in the following table.

Baseline	P=+, N = -		
		XXXX Test at run 2 (Result 2)	
XXXX Test run 1 (Result 1)		N	P
	N	NN	NP
	P	PN	PP
XXXX=Smear or Culture or GeneXpert			
Smear test at baseline	N1	NN	
	P1	NP, PN, PP	
Genexpert	N2	NN	
	P2	NP, PN, PP	
culture	N3	NN	
	P3	NP, PN, PP	
Positive at baseline	PB	P1 or P2 or P3	
Negative at baseline	NB	N1 and N2 and N3	

To define a conversion among those who were positive at baseline, we used the primary outcome response category cured to indicate a negative result at month 6 per the nurse's assessment.

Among the 403 participants who were positive at baseline 168(41.69%) were recorded as cured by 6-month of these 83 (39.9%) in the control arm compared to 85 (43.59%) see Table 4. The odds ratio of conversion is 1.16 (95% CI: (0.83,1.63)) comparing the intervention arm to the control arm. When adjusting for district, sex, and smoking/drinking status and HIV status at baseline, the OR reduces to 1.07 (95% CI: (0.76,1.51))

For peer review only

For peer review only - http://bmjopen.bmj.com/site/about/guidelines.xhtml

8.4.2 6-months Continuous Abstinence

For this outcome, those who smoked cigarettes at baseline were considered as the analytic sample. In addition, those where the following could not be ascertained: self-report of not smoking > 5 cigarettes six months from the start of the abstinence period and supported by a negative biochemical test CO < 7 ppm were considered as smokers for the analysis of this variable. The number of participants who identified as cigarette smokers at baseline were 345 (60.1%)[9].

23 (85.19%) out of 27 with three measurements available[10] managed to abstain continuously for six months. These were similarly distributed across the two study arms, see Table 4. Among those who identified as cigarette smokers, 10 (5.59%) participants in the intervention arm continuously abstained. In the control arm there were 12 (7.23%) who continuously abstained for 6-months.

The crude odds of 6-months continuous abstinence is 0.76 (95% CI: (0.35,1.63)) in the intervention arm compared to the control arm among baseline cigarette smokers. Given the limited number of those who were identified as continually abstained, we were only able to adjust for one additional variable at a time. Adding one of the following variables: heaviness of smoking, type of drinker at baseline, age when started smoking, and the duration of smoking at baseline, the adjusted odds ratio of continuous abstinence comparing the intervention to the control arm did not differ much from the crude estimate of 0.76. The adjusted estimate for the various models ranged between 0.73 and 0.76 with similar confidence limits as for the crude estimate. Furthermore, we did not have evidence that any of the adjusting variables were statistically significantly correlated to continuous abstinence in these models.

We carried an additional analysis where those who died and were smokers at baseline, 22 in total, 20 were cigarette smokers and were removed from the analytical sample for the continuous abstinence outcome. This resulted in a crude

[9] Any type of tobacco smoking at baseline 372 (64.8%). The numbers reflect those who used manufactured cigarettes which were the vast majority.

[10] We had only 27 participants who had self-report of not smoking at 3 months, 6 months and a carbon monoxide reading at 6 months. Continuous abstinence was defined as a self-report of not smoking > 5 cigarettes six months from the start of the abstinence period and supported by a negative biochemical test CO < 7 ppm

For peer review only - http://bmjopen.bmj.com/site/about/guidelines.xhtml

OR of 0.78 which is similar to the OR when the larger analytic sample was considered; this was also the case for the associated 95% CI. Similar estimates were observed when adjusting for the aforementioned variables.

We carried an additional analysis where the analytic sample was all those who were smokers regardless of the type used. There were a total of 372 that identified as smokers. The crude odds ratio in this case changes to 0.86 (95% CI: (0.38,1.95)). The 95% CI is similar to that of the smaller analytic sample. When adjusting for the heaviness of smoking, type of drinker at baseline, age when started smoking, and the duration of smoking at baseline, the adjusted odds ratio of continuous abstinence comparing the intervention to the control arm did not differ much from the crude estimate.

3-months Continuous Abstinence

Among those who identified as cigarette smokers, 20 (11.17%) participants in the intervention arm continuously abstained for 3-months while in the control arm there were 27 (16.27%) who continuously abstained for 3-months.

The crude odds of 3-months continuous abstinence is 0.65 (95% CI: (0.37,1.14)) in the intervention arm compared to the control arm among baseline cigarette smokers. Given the limited number of those who were identified as continually abstained, we were only able to adjust for one additional variable at a time. Adding one of the following variables: heaviness of smoking, type of drinker at baseline, age when started smoking, and the duration of smoking at baseline, the adjusted odds ratio of continuous abstinence comparing the intervention to the control arm did not differ much from the crude estimate of 0.65. The adjusted estimate for the various models ranged between 0.63 and 0.66 with similar confidence limits as for the crude estimate.

For peer review only - http://bmjopen.bmj.com/site/about/guidelines.xhtml

Point Abstinence

There were 57 participants who indicated that they stopped smoking tobacco completely at the 3-month follow-up, of which one had more than 5 cigarettes in the past 3 months. There were 81 participants who indicated that they stopped smoking tobacco completely at the 6-month follow-up, of which three had more than 5 cigarettes in the past 3 months. Only 30 participants had information for the entirety of the 6-month period, of which none consumed more than 5 cigarettes over the past 6-month period. Of these, 23 had a confirmed CO < 7 ppm, 4 had these levels >= 7, and 3 were missing.

182 responded that they continued smoking either as usual or at a reduced rate but regularly at month 3; whereas 133 done so at month 6 of these we had 97 that had measurements at both time points thus resulting in a total of 218 where they have smoked on a regular basis over the past 6 month period. 11 had responded as not smoking in the past three months at month 6 but had missing information for the first three months; 9 of these had carbon monoxide readings available at month 6. Of these nine, two had their CO >= 7 (in fact these were 10 & 10.1).

For peer review only

For peer review only - http://bmjopen.bmj.com/site/about/guidelines.xhtml

8.4.4 Change in harmful or hazardous drinking at month 3 and 6 follow-ups

Alcohol use was measured using the AUDIT questionnaire. We will assess whether there has been a reduction in alcohol consumption three months and six months following recruitment. This analysis will be performed on the group of participants who were harmful or hazardous drinkers at baseline. Figure YYY provides histograms of the AUDIT score at baseline, 3-months and 6-months, respectively, among those who were considered as hazardous or harmful drinkers. It also presents scatterplots of the 3-months and 6-months scores versus the baseline scores for this group by trial arm, respectively.

Among those who were harmful or hazardous drinkers at baseline those in the intervention arm had on average a reduction of 0.04 points (95% CI: (-2,1.91)) on the AUDIT score at 6-months compared to those in the control arm controlling for their baseline score, see Table 6. However, when additionally adjusting for district, sex, and smoking/drinking status (which effectively flags smokers/non-smokers who are also drinkers) and HIV status at baseline; the intervention arm had an average increase of 0.02 points on the AUDIT score (95% CI: (-1.55,1.6)) compared to the control arm. It is worth noting that of the variables in the adjusted model; the only statistically significant result is for the district variable. It seems that those in district “S” score on average 5.8 points less than those in “B” (95% CI: (-11.26,-0.35)); similarly those in “L” score 5 points less than those in “B” on AUDIT but we do not have evidence that this difference is statistically significant (95% CI: (-10.35,0.26)), see Table 6 for further details.

At 3-month, the estimates were an average increase of 0.55 (95% CI: (-1.01,2.11)) on the AUDIT score in the intervention arm compared to the control arm when only accounting for the baseline scores; whereas it increased to 0.74 (95% CI: (0.62,2.1)) when adjusting for other covariates in the model, for further details see Table 7.

For peer review only - http://bmjopen.bmj.com/site/about/guidelines.xhtml

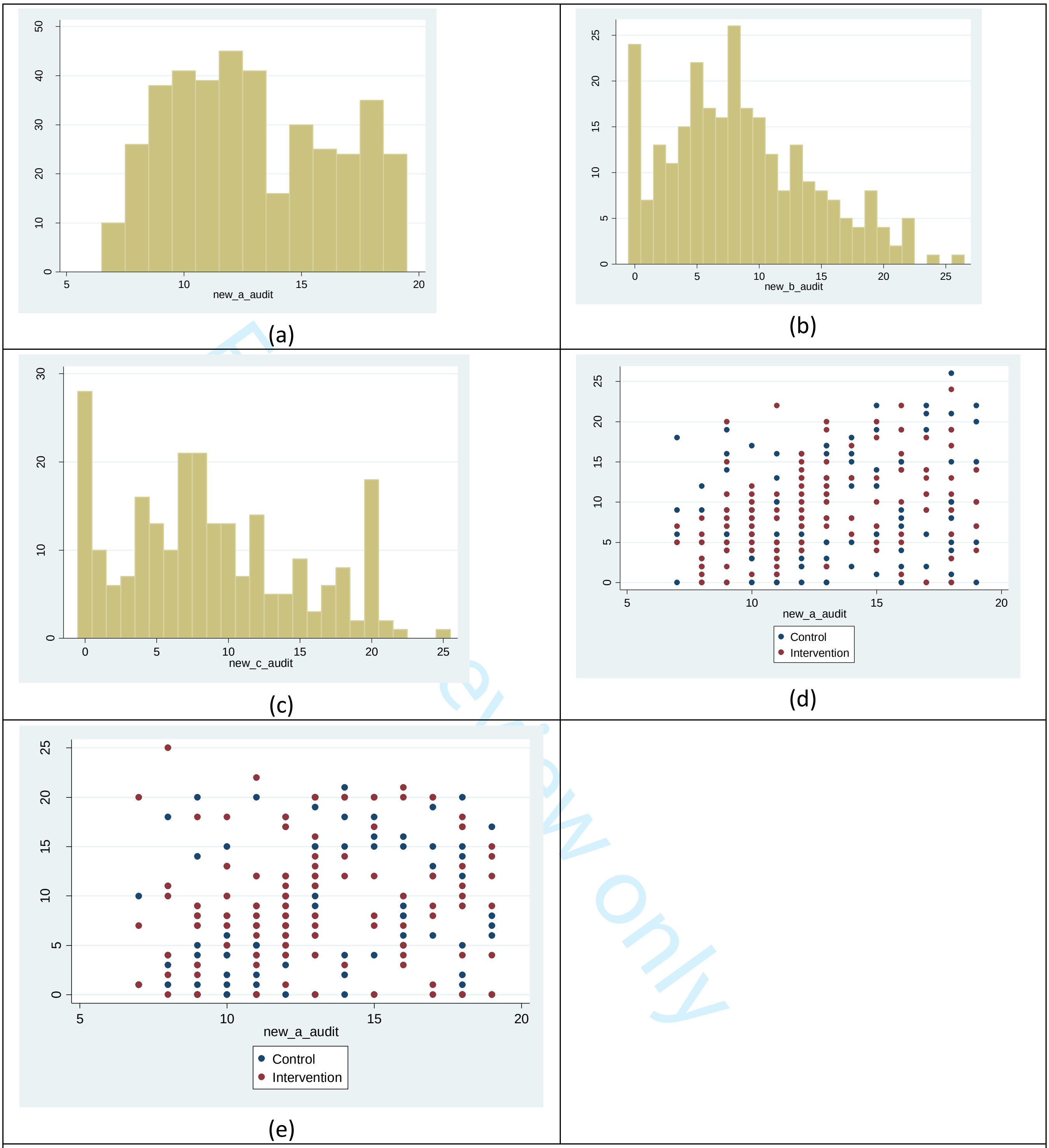

Figure YYY: Graphs (a), (b), and (c) are histograms of the AUDIT score at baseline, 3-months and 6-months, respectively, among those who were considered as hazardous or harmful drinkers. (d) and (e) are scatterplots of the 3-months and 6-months scores versus the baseline scores for this group, respectively.

For peer review only

For peer review only - http://bmjopen.bmj.com/site/about/guidelines.xhtml

8.4.5 Proportion of ART uptake of HIV-positive participants at month 3 and 6 follow-ups

We will assess whether there has been an increase in proportion of HIV-positive participants on ART at three and six months from baseline using standardised questions on the CRF.

There were 171 HIV-positive participants whose baseline ART medication status was known and whose ART medication status was known as well at 6-months. Of these, 123 remained on their medication, 19 took up medication at 6-months compared to not taking medication at baseline, whereas 29 stopped taking their medication at 6-months but were on medication at baseline, see Table 6 for further details.

There were 10 who had an unknown status in terms of medication at baseline and no information was available about them at 6-months, 12 who were initially of unknown medication status who took up medication at 6-months (these were equally distributed between the two arms). Furthermore, there were 10 who were not taking medication at baseline and 102 who were taking medication at baseline whose 6-months medication status was not recorded.

At 6-months, the odds ratio of taking medication at 6-months was 2.05 (95% CI: (0.80,5.27)) in the intervention arm compared to the control arm, controlling for ART baseline medication status.

There were 188 HIV-positive participants whose baseline ART medication status was known and whose ART medication status was known as well at 3-months. Of these, 122 remained on their medication, 16 took up medication at 3-months compared to not taking medication at baseline, whereas 50 stopped taking their medication at 3-months but were on medication at baseline, see Table 7 for further details.

There were 9 who had an unknown status in terms of medication at baseline and no information was available about them at 3-months, 11 who were initially of unknown

For peer review only - http://bmjopen.bmj.com/site/about/guidelines.xhtml

medication status who took up medication at 3-months, and two who were initially of unknown medication status who were not taking medication at 3-months. Furthermore, there were 9 who were not taking medication at baseline and 82 who were taking medication at baseline whose 3-months medication status was not recorded. Furthermore, there were 4 who carried on not taking medication at 3-months.

At 3-months, the odds ratio of taking medication at 6-months was 0.79 (95% CI: (0.38,1.65)) in the intervention arm compared to the control arm, controlling for ART baseline medication status.

Medicine Adherence:

At 3-months 165 (98.8%) of 167 participants had optimal ART medication adherence, whereas 139(97.2%) of 143 had optimal ART medication adherence. These were similar across the two arms.

Similarly, at 3-months 319(91.67%) of 348 participants had optimal TB medication adherence, whereas 120(90.23%) of 133 had optimal TB medication adherence. These were similar across the two arms.

8.5 Subgroup analyses (See above analyses)

We will conduct subgroup analyses to determine whether TB treatment outcomes differ between subgroups, as follows: HIV-positive versus HIV-negative participants; participants with an alcohol problem only versus smokers only versus participants who are conjoint smokers and drinkers; and participants who were GeneXpert positive versus participants who were GeneXpert negative at baseline.

8.5 Sensitivity analyses

In case of missing data, multiple imputations and appropriate sensitivity analyses will be conducted. As it is likely that more than one variable will have missing data we will use multiple imputations using chained equations (**MICE**). A minimum of 10 imputations will be performed; however, the final number of imputations will depend on the missing in the data. We will report the decisions that we make with regard to the number of imputations and the

For peer review only - http://bmjopen.bmj.com/site/about/guidelines.xhtml

variables we use in the imputations. We will also conduct a sensitivity analysis to explore the implications of the missing at random assumption [21p,22p].

8.6 Adverse events

Analysis of adverse events and serious AE will explore whether these differ by treatment arm using Chi-square tests.

8.7 Planned interim review and analyses

No interim analysis is planned. The main analyses will be completed after three months of the data closing.

For peer review only

For peer review only - http://bmjopen.bmj.com/site/about/guidelines.xhtml

8.8 List of Tables and Graphs

Measures of central tendency and percentages will be reported to two decimal places whereas measures of variability and p-values will be reported to three decimal places.

The following is a list of suggested tables and graphs; the templates are included in Appendix A.

TABLE 1: Numbers in the study at Baseline and follow-ups at month 3, and 6 by centre and study arm.

TABLE 2: Descriptive statistics for socio-demographic and socio-economic characteristics at baseline and as analysed by study arm. Frequencies and (percentages) are presented unless otherwise stated

TABLE 3: Descriptive statistics for smoking history, alcohol history, clinical characteristics and depression score at baseline and as analysed by study arm. Frequencies and (percentages) are presented unless otherwise stated.

TABLE 4: Descriptive statistics for primary and secondary outcomes by study arm at baseline (where available), 3 month (where available) and 6 month. Frequencies and (percentages) are presented unless otherwise stated.

TABLE 5: Number and type of adverse events at month 2, 3, and 6 by centre and study arm.

TABLE 6: Regression analysis results for the primary and secondary outcomes at 6 month. Estimates presented with corresponding 95% CI. Crude and adjusted estimates are provided.

TABLE 7: Regression analysis results for the secondary outcomes that are measured at 3 month. Estimates presented with corresponding 95% CI. Crude and adjusted estimates are provided.

For peer review only - http://bmjopen.bmj.com/site/about/guidelines.xhtml

9. SAP amendment log

**Amendment/addition to SAP and reason for change**	**New version number, name and date**
SAP completed and signed-off	*V1.0,*
Updated verion	*V1.1, May 2020*

10. Signatures of approval

Sign-off of the final approved version of the Statistical Analysis Plan by the principle investigator and trial statistician(s) (can also include Trial Manager/Co-ordinator)

**Name**	**Trial Role**	**Signature**	**Date**

For peer review only - http://bmjopen.bmj.com/site/about/guidelines.xhtml

11. References

Moriarty, A.S., Louwagie, G.M., Mdege, N.D. *et al*. ImPROving TB outcomes by modifying LIFE-style behaviours through a brief motivational intervention followed by short text messages (ProLife): study protocol for a randomised controlled trial. *Trials* **20**, 457 (2019) doi:10.1186/s13063-019-3551-9

Department of Health, Republic of South Africa. National Tuberculosis Management Guidelines. 2014. http://www.tbonline.info/media/uploads/documents/ntcp_adult_tb-guidelines-27.5.2014.pdf. Accessed 8 Jan 2018

21p. James R Carpenter and Michael G Kenward. Missing data in clinical trials — a practical guide. Birmingham: National Health Service Co-ordinating Centre for Research Methodology, 2008.

22p. James R Carpenter, Michael G Kenward, and Ian R White. Sensitivity analysis after multiple imputation under missing at random — a weighting approach. Statistical Methods in Medical Research, 16:259–275, 2007.

StataCorp. 2019. *Stata Statistical Software: Release 16*. College Station, TX: StataCorp LLC.

R Core Team (201?). R: A language and environment for statistical computing. R Foundation for Statistical Computing, Vienna, Austria. URL http://www.R-project.org/.

For peer review only - http://bmjopen.bmj.com/site/about/guidelines.xhtml

12. Appendix A (Results tables)

Table 1: Numbers in the study at Baseline and follow-ups at month 3 and 6 by centre and study arm. (SEE FLOWCHART)

TABLE 1: NUMBERS IN THE STUDY AT BASELINE AND FOLLOW-UPS AT MONTH 3 AND 6 BY CENTRE AND STUDY ARM.								
Centre	Baseline		Month 3		Month 6			
	Intervention	Control	Intervention	Control	Intervention	Control		
Total								

For peer review only

TABLE 2: Descriptive statistics for socio-demographic, socio-economic, and clinical characteristics at baseline by study arm. Frequencies and (percentages) are presented unless otherwise stated

TABLE 2: DESCRIPTIVE STATISTICS FOR SOCIO-DEMOGRAPHIC, SOCIO-ECONOMIC, AND CLINICAL CHARACTERISTICS AT BASELINE AND AS ANALYSED BY STUDY ARM. FREQUENCIES AND (PERCENTAGES) ARE PRESENTED UNLESS OTHERWISE STATED				
		Baseline		
	N1/N2	Control	Intervention	Total
		Arm 1	Arm 2	
**Age in years: mean (SD)**	291/283	39.37 (12.60)	38.56 (11.15)	
**Age in years: median (IQR)**				
**Gender**				
Female		69 (23.7)	60 (21.2)	129 (22.5)
Male		222 (76.3)	223 (78.8)	445 (77.5)
Do not want to disclose				
Marital status				
Married or living together		102 (35.1)	95 (33.6)	197 (34.3)
Divorced/separated		20 (6.9)	18 (6.4)	38 (6.6)
Widowed		10 (3.4)	7 (2.5)	17 (3.0)
Never married and never lived together		144 (49.5)	150 (53.0)	294 (51.2)
Declined to answer		15 (5.2)	13 (4.6)	28 (4.9)
**Education**				
No education		7 (2.4)	5 (1.8)	12 (2.1)
Grades 1-5		23 (7.9)	20 (7.1)	43 (7.5)
Grades 6-7		32 (11.0)	35 (12.4)	67 (11.7)

For peer review only - http://bmjopen.bmj.com/site/about/guidelines.xhtml

Grades 8-11		96 (33.0)	128 (45.2)	224 (39.0)
Grade 12		87 (29.9)	70 (24.7)	157 (27.4)
Higher		24 (8.2)	8 (2.8)	32 (5.6)
Declined to answer		22 (7.6)	17 (6.0)	39 (6.8)
**Employment**				
Self-employed (full-time)		30 (10.3)	36 (12.7)	66 (11.5)
Employed full-time (30 hrs a week or more)		62 (21.3)	54 (19.1)	116 (20.2)
Employed part-time (less than 30 hrs a week)		19 (6.5)	29 (10.2)	48 (8.4)
Retired		17 (5.8)	16 (5.7)	33 (5.7)
Unemployed (but able to work)		125 (43.0)	120 (42.4)	245 (42.7)
Unable to work because of long-term disability or ill health		9 (3.1)	8 (2.8)	17 (3.0)
Full-time student		12 (4.1)	4 (1.4)	16 (2.8)
Caring from my home and family/doing household work/housewife		0 (0.0)	2 (0.7)	2 (0.3)
Occasional work ("piece job")		17 (5.8)	12 (4.2)	29 (5.1)
Declined to answer		0 (0.0)	2 (0.7)	2 (0.3)
**Ever worked or spent time in mines**				
No		244 (83.8)	237 (83.7)	481 (83.8)
Yes		46 (15.8)	45 (15.9)	91 (15.9)
**Socioeconomic status**				

For peer review only - http://bmjopen.bmj.com/site/about/guidelines.xhtml

**Household items (Yes is displayed)**				
A radio		249 (85.6)	234 (82.7)	483 (84.1)
A television		255 (87.6)	255 (90.1)	510 (88.9)
A landline telephone		21 (7.2)	22 (7.8)	43 (7.5)
A desktop or laptop computer		67 (23.0)	48 (17.0)	115 (20.0)
A refrigerator		248 (85.2)	240 (84.8)	488 (85.0)
A vacuum cleaner or floor pol		49 (16.8)	35 (12.4)	84 (14.6)
A microwave oven		198 (68.0)	189 (66.8)	387 (67.4)
An electric or gas stove		254 (87.3)	238 (84.1)	492 (85.7)
A washing machine		153 (52.6)	136 (48.1)	289 (50.3)
Total number of assets: mean (SD)		5.14 (1.96)	4.94 (1.77)	5.04 (1.87)
**In the past month, number of days you or people in the household went to bed hungry because there was no food to eat**				
0 days		244 (83.8)	238 (84.1)	482 (84.0)
1-7 days		45 (15.5)	34 (12.0)	79 (13.8)
More than 7 days		2 (0.7)	9 (3.2)	11 (1.9)
Declined to answer		0 (0.0)	2 (0.7)	2 (0.3)
**TB and medical history**				
**TB history**				
**Patient category**				
New patient		264 (92.3)	249 (90.2)	513 (91.3)
Relapse		10 (3.5)	9 (3.3)	19 (3.4)
Re-treatment after default		9 (3.1)	14 (5.1)	23 (4.1)
Re-treatment after failure		1 (0.3)	2 (0.7)	3 (0.5)
Other		2 (0.7)	2 (0.7)	4 (0.7)
**Site of disease**				
Pulmonary and Extra Pulmonary (ICD-10 A17-A19)		3 (1.1)	3 (1.1)	6 (1.1)

For peer review only - http://bmjopen.bmj.com/site/about/guidelines.xhtml

Pulmonary only (ICD-10 A15)		281 (98.9)	272 (98.9)	553 (98.9)
**Number of pre-treatment smear results**				
One		197 (92.1)	169 (87.6)	366 (89.9)
Two		17 (7.9)	24 (12.4)	41 (10.1)
**Smear result**	N =	196	180	376
**At least one positive smear result**		111 (56.63)	109 (60.56)	220 (58.51)
**Number of Gene XPert results recorded**				
One		225 (97.8)	210 (97.2)	435 (97.5)
Two		5 (2.2)	6 (2.8)	11 (2.5)
**Gene XPert result**	N =	211	204	415
**At least one positive Gene XPert result**		184 (87.2)	178 (87.25)	362 (87.23)
**Number of culture results recorded on the TB Treatment record**				
One		54 (94.7)	42 (97.7)	96 (96.0)
Two		3 (5.3)	1 (2.3)	4 (4.0)
**Culture result**	N =	41	32	73
**At least one positive culture result**		20 (48.78)	15 (46.88)	35 (47.95)
**Co-morbidities**				
Hypertension		19 (6.93)	11 (4.1)	30 (5.54)
Diabetes		5 (1.84)	4 (1.49)	9 (1.66)
Epilepsy		3 (1.09)	4 (1.49)	7 (1.29)
Mental illness		3 (1.09)	0 (0)	3 (0.55)
Liver disease		1 (0.36)	1 (0.38)	2 (0.37)
Renal insufficiency		1 (0.36)	1 (0.38)	2 (0.37)
Allergies		2 (0.76)	0 (0)	2 (0.38)

For peer review only - http://bmjopen.bmj.com/site/about/guidelines.xhtml

Other		1 (0.36)	1 (0.38)	2 (0.37)
**Total Number of comorbidities**				
0		265 (96.0)	260 (96.7)	525 (96.3)
1		10 (3.6)	8 (3.0)	18 (3.3)
2		0 (0.0)	1 (0.4)	1 (0.2)
5		1 (0.4)	0 (0.0)	1 (0.2)
**HIV status**				
Negative		118 (40.7)	125 (44.2)	243 (42.4)
Positive		163 (56.2)	142 (50.2)	305 (53.2)
Unknown		9 (3.1)	16 (5.7)	25 (4.4)
**HIV positive patients**				
**CD4 Count: mean(SD)**				
**Using Cotrimoxazole**		104 (63.8)	100(67.11)	204 (65.38)
**Using anti-retroviral**		139 (85.28)	118 (79.19)	257 (82.37)

For peer review only - http://bmjopen.bmj.com/site/about/guidelines.xhtml

TABLE 3: Descriptive statistics for smoking history, alcohol history and depression score at baseline by study arm. Frequencies and (percentages) are presented unless otherwise stated.

TABLE 3: Descriptive statistics for smoking history, alcohol history and depression score at baseline and as analysed by study arm. Frequencies and (percentages) are presented unless otherwise stated.			
	Baseline		
	Control	Intervention	Total
	Arm 1	Arm 2	Total
**In the past month, smoked tobacco**			
Not at all	110 (37.8)	92 (32.5)	202 (35.2)
Daily	149 (51.2)	149 (52.7)	298 (51.9)
Less than Daily	32 (11.0)	42 (14.8)	74 (12.9)
**Had a drink in the past 12-months**	208 (71.5)	223 (78.8)	431 (75.1)
**AUDIT Score (males):** mean (SD) [max :19] [min = 8 if drinkers only]	12.27 (3.98)	13.02 (3.78)	12.66 (3.89)
**AUDIT Score (females):** mean (SD) [max :19] [min = 7 if drinkers only]	11.32 (4.02)	10.98 (4.02)	11.15 (4)
**Drinking and Smoking Combined (Constructed)**			
Drinkers Only	110 (37.8)	92 (32.5)	202 (35.2)
Smokers Only	83 (28.5)	60 (21.2)	143 (24.9)
Smokers and Drinkers	98 (33.7)	131 (46.3)	229 (39.9)

For peer review only - http://bmjopen.bmj.com/site/about/guidelines.xhtml

	Arm 1	Arm 2	Total
**Smoking History (current smokers only)**	181	191	372
On the days that you smoke, how soon after you wake up do you have your first cigarette?			
After 60 minutes	30 (16.6)	28 (14.7)	58 (15.6)
31-60 minutes	24 (13.3)	16 (8.4)	40 (10.8)
6- 30 minutes	60 (33.1)	65 (34.0)	125 (33.6)
Within 5 minutes	67 (37.0)	82 (42.9)	149 (40.1)
Duration of smoking in **months**: mean (SD)	212.09 (134.03)	224.93 (127.82)	218.68 (130.86)
Duration of smoking in **months**: median (IQR)	186 (110, 282)	206 (135, 294)	200.5 (123, 287)
Age started smoking in **years**: mean (SD)	19.2 (6.3)	19.3 (6.3)	19.3 (6.3)
Age started smoking in **years**: median (IQR)	18 (15-20)	18 (16-21)	18 (15.5-20.5)
**Form of tobacco used**			
**Manufactured cigarettes (Yes)**	**166 (91.7)**	**179 (93.7)**	**345 (92.7)**
Number of days in the past 7days you smoked: mean (SD)	5.3 (2.65)	5.53 (2.33)	5.42 (2.49)
Average number of cigarettes smoked daily: mean (SD)	6.18 (6.43)	6.48 (8.21)	6.34 (7.39)
**Hand-rolled cigarettes (Yes)**	**14 (7.7)**	**21 (11.0)**	**35 (9.4)**
Number of days in the past 7days you smoked: mean (SD)	4.71 (2.84)	3.71 (2.95)	4.11 (2.91)
Average number of handrolled cigarettes smoked daily: mean (SD)	3.71 (3.97)	3.81 (2.82)	3.77 (3.27)
**Pipe (Yes)**	**4 (2.2)**	**2 (1.0)**	**6 (1.6)**
Number of days in the past 7days you smoked: median (IQR)	1 (0, 2.5)	4.5 (2, 7)	2 (0, 3)
Average number of daily sessions: median (IQR)	1 (0, 3.5)	3 (1, 5)	1.5 (0, 5)
Length of one session (on average) in **minutes**: median (IQR)	90 (60, 107.5)	60 (30, 90)	90 (30, 90)
**Cigars, cheroots or cigarillos (Yes)**	1 (0.55)	0 (0)	1(0.27)

For peer review only - http://bmjopen.bmj.com/site/about/guidelines.xhtml

**Water pipe (Yes)**	3 (1.7)	2 (1.0)	5 (1.3)
Other	10 (5.5)	4 (2.1)	14 (3.8)
**Heaviness of smoking index >= 4**	134(74.03)	158(82.72)	292(78.49)
**Smoking inside your home restrictions**			
Total: Not allowed	96 (53.0)	108 (56.5)	204 (54.8)
Some rules: where/when it is allowed	61 (33.7)	58 (30.4)	119 (32.0)
No rules	24 (13.3)	25 (13.1)	49 (13.2)
**Attempts to quit smoking (current smokers only) (Yes)**			
Ever attempted to quit in the past			
Yes	52 (28.7)	64 (33.5)	116 (31.2)
No	129 (71.3)	127 (66.5)	256 (68.8)
Number of attempts to quit: mean (SD)	2.46 (2.98)	2.64 (1.62)	2.56 (2.32)
Time elapsed since attempt to quit last time in months: mean (SD)	36.65 (94.62)	25.13 (37.91)	30.29 (69.22)
Longest duration abstinent in previous quit attempts: mean (SD)	6.15 (13.48)	4.22 (8.9)	5.09 (11.18)
Likelihood to TRY TO QUIT smoking completely and permanently in the next three months			
definitely will not	6 (3.3)	5 (2.6)	11 (3.0)
probably will not	10 (5.5)	12 (6.3)	22 (5.9)
probably will	104 (57.5)	109 (57.1)	213 (57.3)
definitely will	61 (33.7)	65 (34.0)	126 (33.9)
	Control	Intervention	

For peer review only - http://bmjopen.bmj.com/site/about/guidelines.xhtml

	Arm 1	Arm 2	Total
Likelihood that I WILL QUIT smoking completely and permanently in the next three months			
definitely will not	6 (3.3)	5 (2.6)	11 (3.0)
probably will not	11 (6.1)	13 (6.8)	24 (6.5)
probably will	103 (56.9)	104 (54.5)	207 (55.6)
definitely will	61 (33.7)	69 (36.1)	130 (34.9)
**Ever used any methods to help you stop smoking tobacco in the past 3 months? (**Yes)	23 (12.7)	16 (8.4)	39 (10.5)
**Out of your pocket spend (in Rands) on methods to help you stop smoking in the past 3 months: mean (SD)**	134.87 (237.58)	139.19 (155.07)	136.64 (205.36)
**Average spend per week on cigarettes over the past 3 months: mean (SD)**	168.77 (178.15)	180.29 (185.27)	174.58 (181.61)
**Smokeless tobacco use (all participants)**			
**In the past month, have you used smokeless tobacco (Snuff) on a daily basis**			
Not at all	275 (94.5)	275 (97.2)	550 (95.8)
Daily	12 (4.1)	6 (2.1)	18 (3.1)
Less than Daily	4 (1.4)	2 (0.7)	6 (1.0)
Duration of using ST in months: mean(SD)	113.69 (112.7)	152 (91.16)	126.46 (105.61)
Age started using ST in **years**: mean (SD)	27.56 (10.57)	25.88 (13.43)	27 (11.33)
**Form of ST used (for SLT users)**			
Snuff (by mouth)	2 (12.5)	0 (0.0)	2 (8.3)
Snuff (by nose)	11 (68.8)	5 (62.5)	16 (66.7)
Chewing tobacco leaves	0(0)	0(0)	0(0)
Other	1 (6.3)	0 (0.0)	1 (4.2)
	Arm 1	Arm 2	Total

For peer review only - http://bmjopen.bmj.com/site/about/guidelines.xhtml

	Control	Intervention	
**Tobacco smoke exposure:** number of days in the past 30 days, you were in a place where someone smoked close to you (all participants?)			
**Home**			
7 days or less	233 (80.1)	229 (80.9)	462 (80.5)
More than 7 days	58 (19.9)	54 (19.1)	112 (19.5)
**Workplace** (Missing 315; probably Not Applicable)			
7 days or less	84 (65.6)	89 (67.9)	173 (66.8)
More than 7 days	44 (34.4)	42 (32.1)	86 (33.2)
**Cafes/restaurants**			
7 days or less	230 (79.0)	234 (82.7)	464 (80.8)
More than 7 days	61 (21.0)	49 (17.3)	110 (19.2)
**Shebeens, bars or clubs**			
7 days or less	219 (75.3)	213 (75.3)	432 (75.3)
More than 7 days	72 (24.7)	70 (24.7)	142 (24.7)
**Bus/train/taxi/ vehicle**			
7 days or less	229 (78.7)	219 (77.4)	448 (78.0)
More than 7 days	62 (21.3)	64 (22.6)	126 (22.0)
**Shops/shopping mall**			
7 days or less	235 (80.8)	226 (79.9)	461 (80.3)
More than 7 days	56 (19.2)	57 (20.1)	113 (19.7)
	Arm 1	Arm 2	Total

For peer review only - http://bmjopen.bmj.com/site/about/guidelines.xhtml

**Had a drink in the past 12-months**	208 (71.5)	223 (78.8)	431 (75.1)
**Help to stop drinking (drinkers only)**			
Ever used any methods to stop drinking alcohol in the past 3 months	21 (11.2)	22 (10.7)	43 (10.9)
Average spend in Rands per week on alcohol over the past 3 months: mean (SD)	363.76 (531.56)	337.03 (387.94)	349.79 (461.66)
**Depression: CESD 10** how often you felt or behaved this way during the past week			
I was bothered by things that usually don't bother me.			
Rarely or None of the Time (Less than 1 day)	180 (61.9)	159 (56.2)	339 (59.1)
Some or a Little of the Time (1-2 days)	71 (24.4)	78 (27.6)	149 (26.0)
Occasionally or a Moderate Amount of the Time (3-4 days)	24 (8.2)	31 (11.0)	55 (9.6)
Most or All of the Time (5-7 days)	16 (5.5)	15 (5.3)	31 (5.4)
I had trouble keeping my mind on what I was doing.			
Rarely or None of the Time (Less than 1 day)	150 (51.5)	136 (48.1)	286 (49.8)
Some or a Little of the Time (1-2 days)	93 (32.0)	85 (30.0)	178 (31.0)
Occasionally or a Moderate Amount of the Time (3-4 days)	31 (10.7)	38 (13.4)	69 (12.0)
Most or All of the Time (5-7 days)	17 (5.8)	24 (8.5)	41 (7.1)
I felt depressed.			
Rarely or None of the Time (Less than 1 day)	152 (52.2)	171 (60.4)	323 (56.3)
Some or a Little of the Time (1-2 days)	94 (32.3)	70 (24.7)	164 (28.6)
Occasionally or a Moderate Amount of the Time (3-4 days)	37 (12.7)	30 (10.6)	67 (11.7)
Most or All of the Time (5-7 days)	8 (2.7)	12 (4.2)	20 (3.5)
I felt that everything I did was an effort.			

For peer review only - http://bmjopen.bmj.com/site/about/guidelines.xhtml

Rarely or None of the Time (Less than 1 day)	140 (48.1)	117 (41.3)	257 (44.8)
Some or a Little of the Time (1-2 days)	77 (26.5)	87 (30.7)	164 (28.6)
Occasionally or a Moderate Amount of the Time (3-4 days)	38 (13.1)	43 (15.2)	81 (14.1)
Most or All of the Time (5-7 days)	36 (12.4)	36 (12.7)	72 (12.5)
I felt hopeful about the future.			
Rarely or None of the Time (Less than 1 day)	85 (29.2)	84 (29.7)	169 (29.4)
Some or a Little of the Time (1-2 days)	78 (26.8)	70 (24.7)	148 (25.8)
Occasionally or a Moderate Amount of the Time (3-4 days)	53 (18.2)	56 (19.8)	109 (19.0)
Most or All of the Time (5-7 days)	75 (25.8)	73 (25.8)	148 (25.8)
I felt fearful.			
Rarely or None of the Time (Less than 1 day)	175 (60.1)	159 (56.2)	334 (58.2)
Some or a Little of the Time (1-2 days)	82 (28.2)	79 (27.9)	161 (28.0)
Occasionally or a Moderate Amount of the Time (3-4 days)	25 (8.6)	32 (11.3)	57 (9.9)
Most or All of the Time (5-7 days)	9 (3.1)	13 (4.6)	22 (3.8)
My sleep was restless.			
Rarely or None of the Time (Less than 1 day)	142 (48.8)	135 (47.7)	277 (48.3)
Some or a Little of the Time (1-2 days)	95 (32.6)	89 (31.4)	184 (32.1)
Occasionally or a Moderate Amount of the Time (3-4 days)	31 (10.7)	33 (11.7)	64 (11.1)
Most or All of the Time (5-7 days)	23 (7.9)	26 (9.2)	49 (8.5)
I was happy.			
Rarely or None of the Time (Less than 1 day)	85 (29.2)	74 (26.1)	159 (27.7)
Some or a Little of the Time (1-2 days)	70 (24.1)	65 (23.0)	135 (23.5)
Occasionally or a Moderate Amount of the Time (3-4 days)	73 (25.1)	86 (30.4)	159 (27.7)
Most or All of the Time (5-7 days)	63 (21.6)	58 (20.5)	121 (21.1)
I felt lonely.			

For peer review only - http://bmjopen.bmj.com/site/about/guidelines.xhtml

Rarely or None of the Time (Less than 1 day)	158 (54.3)	161 (56.9)	319 (55.6)
Some or a Little of the Time (1-2 days)	89 (30.6)	83 (29.3)	172 (30.0)
Occasionally or a Moderate Amount of the Time (3-4 days)	23 (7.9)	26 (9.2)	49 (8.5)
Most or All of the Time (5-7 days)	21 (7.2)	13 (4.6)	34 (5.9)
I could not get "going".			
Rarely or None of the Time (Less than 1 day)	168 (57.7)	174 (61.5)	342 (59.6)
Some or a Little of the Time (1-2 days)	78 (26.8)	69 (24.4)	147 (25.6)
Occasionally or a Moderate Amount of the Time (3-4 days)	35 (12.0)	29 (10.2)	64 (11.1)
Most or All of the Time (5-7 days)	10 (3.4)	11 (3.9)	21 (3.7)
Total CESD 10: mean (SD)	8.44 (4.38)	8.74 (4.8)	8.59 (4.59)

For peer review only - http://bmjopen.bmj.com/site/about/guidelines.xhtml

Table 4: Descriptive statistics for primary and secondary outcomes by study arm at baseline (where available), 3 month (where available) and 6 month. Frequencies and (percentages) are presented unless otherwise stated

For peer review only

For peer review only - http://bmjopen.bmj.com/site/about/guidelines.xhtml

TABLE 4: DESCRIPTIVE STATISTICS FOR PRIMARY AND SECONDARY OUTCOMES BY STUDY ARM AT BASELINE (WHERE AVAILABLE), 3 MONTH (WHERE AVAILABLE) AND 6 MONTH. FREQUENCIES AND (PERCENTAGES) ARE PRESENTED UNLESS OTHERWISE STATED.

	Baseline			Follow-up 3-month			Follow-up 6-month		
	Control	Intervention	Total	Control	Interventio n	Total	Control	Intervention	Total
	Arm1	Arm2	Total	Arm1	Arm2	Total	Arm1	Arm2	Total
**TB treatment status detailed**									
Cured							108 (37.11)	105 (37.1)	213 (37.11)
Treatment completed							96 (33.0)	87 (30.74)	183 (31.88)
Treatment default							15 (5.2)	29 (10.25)	44 (7.67)
Treatment failure							5 (1.7)	2 (0.7)	7 (1.2)
Acquired drug resistance							1 (0.34)	4 (1.41)	5 (0.87)
Died							11 (3.78)	15 (5.30)	26 (4.53)
Transfer out							8 (2.75)	11 (3.89)	19 (3.31)
Unknown							42 (14.43)	25 (8.83)	67 (11.67)
Missing							5 (1.72)	5 (1.77)	10 (1.74)
**TB treatment status binary (Primary outcome**)**									
Not Successful							87 (29.9)	91 (32.16)	178 (31.01)
Successful							204 (70.10)	192 (67.84)	396 (68.99)
**At least one positive smear result**	85 (53.46)	96 (61.15)	181 (57.28)						
**At least one positive Gene XPert result**	184 (87.20)	178 (87.25)	362 (87.23)						
**At least one positive culture result**	11 (57.89)	10 (66.67)	21 (61.76)						

For peer review only - http://bmjopen.bmj.com/site/about/guidelines.xhtml

**Sputum smear, Gene XPert, or culture result**									
Negative	29 (12.45)	32 (14.16)	61 (13.29)						
Positive	204 (87.55)	194 (85.84)	398 (86.71)						
**Conversion from positive to negative*** **									
Yes							83(39.9)	85(43.59)	168(41.69)
No							125(60.1)	110(56.41)	235(58.31)
**Continuous smoking abstinence among cigarette smokers at baseline**									
Yes				27(16.27)	20(11.17)	47(13.62)	12(7.23)	10(5.59)	22(6.38)
No				139(83.73)	159(88.83)	298(86.38)	154(92.77)	169(94.41)	323(93.62)
**Harmful or hazardous drinking[1]**									
**Had a drink in the past 12-months**	208 (71.5)	223 (78.8)	431 (75.1)						
AUDIT score: mean (SD)	12.03 (4)	12.53 (3.93)	12.29 (3.96)						
**Harmful or hazardous drinkers out of those who had a drink in the past 12-months at baseline (%)**	188(90.38)	206(92.38)	394(91.42)	141	130	271	112	127	239
**AUDIT Score (males):** mean (SD) [max :19; min = 8]*!	13.14 (3.31)	13.61 (3.29)	13.39 (3.31)	8.20(6.08)	9.08(4.97)	8.63(5.58)	9.21(6.58)	8.24(5.41)	8.69(5.99)
**AUDIT Score (females):** mean (SD) [max :19; min = 7]*!	11.73 (3.52)	11.55 (3.6)	11.64 (3.54)	8.5(6.52)	8.15(6.44)	8.33(6.44)	7.67(6.84)	9.97(6.79)	8.89(6.86)
AUDIT score: mean (SD)	12.76 (3.42)	13.12 (3.47)	12.94 (3.45)	8.28(6.18)	8.84(5.38)	8.55(5.81)	8.79(6.66)	8.70(5.83)	8.74(6.22)
Difference from baseline				-4.61 (6.26)	-4.07(5.33)	-4.35(5.83)	-4.25(6.56)	-4.17(6.61)	-4.21(6.57)

For peer review only - http://bmjopen.bmj.com/site/about/guidelines.xhtml

**Taking ART medication among** HIV Positive patients	[163 (56.2)]	[142 (50.2)]	[305 (53.2)]	[122]	[83]	[205]	[100]	[83]	[183]
Yes	139 (85.28)	115 (80.99)	254 (83.28)	91 (74.6)	58 (69.9)	149 (72.7)	80 (80.0)	74 (89.2)	154 (84.2)
**ART medication adherence**									
Optimal adherence				101(99.02)	64(98.46)	165(98.8)	75(97.4)	64(96.97)	139(97.2)
Suboptimal adherence				1(0.98)	1(1.54)	2(1.2)	2(2.6)	2(3.03)	4(2.8)
**TB medication adherence**									
Optimal adherence				181(92.35)	138(90.79)	319(91.67)	61(89.71)	59(90.77)	120(90.23)
Suboptimal adherence				15(7.65)	14(9.21)	29(8.33)	7(10.29)	6(9.23)	13(9.77)

[1] hazardous/harmful drinkers who are not alcohol dependent= AUDIT score ≥ 8 for men or ≥ 7 for women but < 20

**Primary Outcome: in the published protocol paper "This is a binary variable defined as either successful treatment (cured or treatment completed) or failed treatment, death, acquired drug resistance, loss to follow-up or 'default', or not outcome evaluated.

*** Conversion from positive to negative: this was based on having a cured treatment outcome among those who were positive at baseline.

*! Important distinction at baseline for eligibility purposes.

For peer review only - http://bmjopen.bmj.com/site/about/guidelines.xhtml

TABLE 5: Number and type of adverse events at month 2, 3, and 6 by centre and study arm (see other document)

TABLE 5: Number and type of adverse events at month 2, 3, and 6 by centre and study arm

Centre		Month 2		Month 3		Month 6	
	TYPE	Intervention	Control	Intervention	Control	Intervention	Control

For peer review only

For peer review only - http://bmjopen.bmj.com/site/about/guidelines.xhtml

TABLE 6: Regression analysis results for the primary and secondary outcomes at 6 months. Estimates presented with corresponding 95% CI. Crude and adjusted estimates are provided.

TABLE 6: Regression analysis results for the primary and secondary outcomes at 6 months. Estimates presented with corresponding 95% CI. Crude and adjusted estimates are provided.

	Crude Odds Ratio (95% CI)*	P Value*	Adjusted Odds Ratio (95% CI)*	P Value*	
**Primary outcome**					
TB treatment status: Successful (**Ref: Not successful**)	0.9 (0.64,1.27)	0.548	0.86 (0.60,1.24)	0.421	
**Secondary outcomes**					
Sputum smear or culture result: converted from positive to negative (Ref: Not converted)	1.16 (0.83,1.63)	0.374	1.07‡ (0.76,1.51)	0.684	
Six-month continuous smoking abstinence among cigarette smokers at baseline (Ref: No)	0.76 (0.35,1.64)	0.482			
Taking ART medication among HIV positive patients[!!]	2.05 (0.80,5.27)	0.136			
TB medication adherence (Reference: Optimal)	0.89 (0.26,3.07)	0.849			
ART medication adherence (Reference: Optimal)	1.17 (0.14,9.94)	0.884			

For peer review only - http://bmjopen.bmj.com/site/about/guidelines.xhtml

	Study arm regression coefficient		Study arm regression coefficient		
**AUDIT for those who were harmful or hazardous drinkers at baseline**:**	-0.04 (-2,1.91)	0.966	0.02! (-1.55,1.6)	0.976	

* analyses accounted for potential clustering by centre.
† Number of participants whose outcome was treatment successful among the total number in the group.
‡ Adjusted for district, sex, and smoking/drinking status and HIV status at baseline
**Controlling for the AUDIT baseline values.
! Controlling for the AUDIT baseline values and adjusted for district, sex, and smoking/drinking status and HIV status at baseline
!! Adjusting for art status at baseline

For peer review only

TABLE 7: Regression analysis results for secondary outcomes measured at 3-months. Estimates presented with corresponding 95% CI. Crude and adjusted estimates are provided.

TABLE 7: Regression analysis results for secondary outcomes measured at 3-months. Estimates presented with corresponding 95% CI. Crude and adjusted estimates are provided.

	Crude Odds Ratio (95% CI)	P Value	Adjusted Odds Ratio (95% CI)	P Value	
**Secondary outcome**					
3-months continuous smoking abstinence among cigarette smokers at baseline	0.65 (0.37,1.14)	0.135			
Taking ART medication among HIV positive patients[!!]	0.79 (0.38,1.65)	0.53	0.74‡ (0.35,1.58)	0.443	
TB medication adherence (Reference: Optimal)	1.22 (0.52,2.87)	0.641	[redacted]		
ART medication adherence (Reference: Optimal)	1.58 (0.10,26.12)	0.750	[redacted]		
	Study arm regression coefficient		Adjusted estimates		
AUDIT for those who were harmful or hazardous drinkers at baseline**:	0.55 (-1.01,2.11)	0.474	0.74 (-0.62,2.1)	0.273	

* analyses accounted for clustering.
‡ Adjusted for district, sex, and smoking/drinking status and HIV status at baseline
**Controlling for the AUDIT baseline values.
! Controlling for the AUDIT baseline values and adjusted for district, sex, and smoking/drinking status and HIV status at baseline
!! Adjusting for art status at baseline

For peer review only

Additional tables
SMS-fidelity

Variable	Intervention (N= ….) (=no. of participants allocated to intervention group, for example 245)	Control (N=…) (=no. participants allocated to control group, for example 248)
No. of participants who received ALL due IMB messages INDEPENDENT OF WHETHER they completed MI 1 (i.o.w this is ITT analysis)	For example 120/245 (49 %)	For example 2/248 (…%)
Completion of first MI and initiation of SMS-sequence		
Completed first MI	n/N (%) (=No. who completed first MI in intervention arm/ no. participants allocated to intervention arm [%] For example 170/245 [69%])	n/N (%) (=No. who completed first MI in control/ no. participants allocated to control [%] For example 2/248)
No. of participants who received ALL due messages after receipt of MI1 and SMS sequence was generated (this is a type of Per Protocol analysis:	For example 120/170 (71%)	2/2

For peer review only - http://bmjopen.bmj.com/site/about/guidelines.xhtml

denominator limited to those who had MI1)				
SMS delivery for participants for whom the SMS-sequence was initiated (after receipt of first MI)				
	Mean (SD)	Median (IQR) Range	Mean (SD)	Median (IQR)
Average no. adherence messages received per participant (n=…) (n = the no. of participants who completed first MI, in this example 170)	For example 8 (3.4)	9 (7-10)<br><br>Range 0-10	Expected to be 0 or close to it	Expected to be 0 or close to it
Average no. tobacco-related messages received (n=…) (n=no of participants who completed first MI AND were current tobacco users at baseline, for example 90)	For example 7 (3.4)	7 (6-7)<br><br>Range 0-7	Expected to be 0 or close to it	Expected to be 0 or close to it
Average no. alcohol related messages received (n=…) (n=no. participants who completed first MI	For example 7 (3.4)	7 (6-7)<br><br>Range 0-7	Expected to be 0 or close to it	Expected to be 0 or close to it

For peer review only - http://bmjopen.bmj.com/site/about/guidelines.xhtml

AND were drinkers at baseline=....for example 100)				
Average no. IMB messages received (n= total no who completed first MI)	For example 16 (4.5)	15 (10-20)<br>Range 0-24	Expected to be 0 or close to it	Expected to be 0 or close to it

Other variables not yet analysed:

CESD at 3 and 6-month FU
Other smoking related questions at 3 and 6 month (quit intentions etc) but also SLT use at 3 and 6 month. (important because participants may have switched from tobacco smoking to SLT)
Other non-HE questions not yet analysed

For peer review only - http://bmjopen.bmj.com/site/about/guidelines.xhtml

13. Additional descriptive Statistics

TABLE 8: Additional Descriptive statistics for characteristics at baseline by study arm. Frequencies and (percentages) are presented unless otherwise stated

		Baseline		
	N1/N2	Intervention	Control	Total
		Arm 1	Arm 2	
TYPE OF MINE WORK	46/45			91
Coal		7 (15.2)	3 (6.7)	10 (11.0)
Diamond		3 (6.5)	1 (2.2)	4 (4.4)
Gold		13 (28.3)	17 (37.8)	30 (33.0)
Platinum and palladium		24 (52.2)	17 (37.8)	41 (45.1)
Chromium		11 (23.9)	12 (26.7)	23 (25.3)
Uranium		1 (2.2)	1 (2.2)	2 (2.2)
Manganese		0(0)	0(0)	0(0)
Other		0(0)	0(0)	0(0)
Total Types of Mines worked in				
1	73			
2	12			
3	3			
4	1			

For peer review only

For peer review only - http://bmjopen.bmj.com/site/about/guidelines.xhtml

Exact Distribution for number smoked in the past seven days				
0		24 (14.5)	16 (8.9)	40 (11.6)
1		4 (2.4)	4 (2.2)	8 (2.3)
2		4 (2.4)	3 (1.7)	7 (2.0)
3		5 (3.0)	13 (7.3)	18 (5.2)
4		13 (7.8)	8 (4.5)	21 (6.1)
5		5 (3.0)	17 (9.5)	22 (6.4)
6		1 (0.6)	3 (1.7)	4 (1.2)
7		110 (66.3)	115 (64.2)	225 (65.2)
new_a_positive_TB				
0		29 (11.98)	33 (14.35)	62 (13.14)
1		122 (50.41)	104 (45.22)	226 (47.88)
2		80 (33.06)	81 (35.22)	161 (34.11)
3		11 (4.55)	12 (5.22)	23 (4.87)

For peer review only - http://bmjopen.bmj.com/site/about/guidelines.xhtml

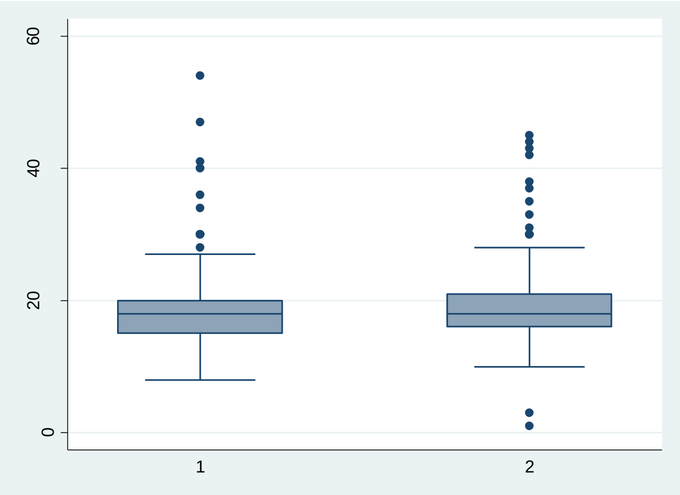

Age started smoking

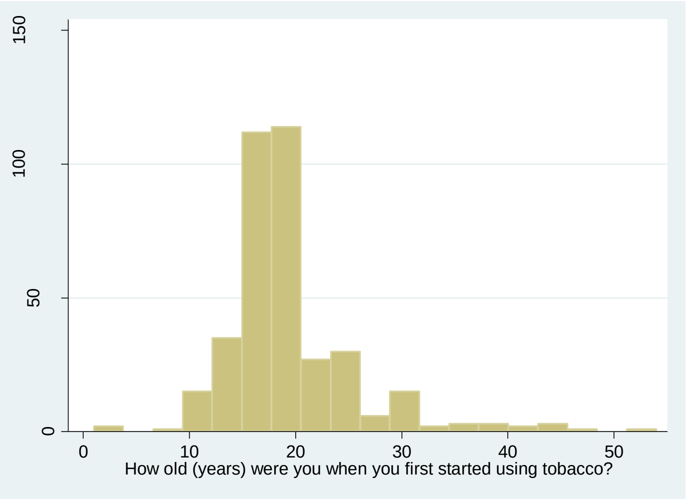

For peer review only

CONSORT 2010 checklist of information to include when reporting a randomised trial*

Section/Topic	Item No	Checklist item	Reported on page No
**Title and abstract**			
	1a	Identification as a randomised trial in the title	1
	1b	Structured summary of trial design, methods, results, and conclusions (for specific guidance see CONSORT for abstracts)	2
**Introduction**			
Background and objectives	2a	Scientific background and explanation of rationale	4-5
	2b	Specific objectives or hypotheses	5-7
**Methods**			
Trial design	3a	Description of trial design (such as parallel, factorial) including allocation ratio	5-6
	3b	Important changes to methods after trial commencement (such as eligibility criteria), with reasons	NA
Participants	4a	Eligibility criteria for participants	5
	4b	Settings and locations where the data were collected	5
Interventions	5	The interventions for each group with sufficient details to allow replication, including how and when they were actually administered	6
Outcomes	6a	Completely defined pre-specified primary and secondary outcome measures, including how and when they were assessed	7-8
	6b	Any changes to trial outcomes after the trial commenced, with reasons	NA
Sample size	7a	How sample size was determined	8
	7b	When applicable, explanation of any interim analyses and stopping guidelines	NA
Randomisation:			
Sequence generation	8a	Method used to generate the random allocation sequence	6
	8b	Type of randomisation; details of any restriction (such as blocking and block size)	6
Allocation concealment mechanism	9	Mechanism used to implement the random allocation sequence (such as sequentially numbered containers), describing any steps taken to conceal the sequence until interventions were assigned	6
Implementation	10	Who generated the random allocation sequence, who enrolled participants, and who assigned participants to interventions	6
Blinding	11a	If done, who was blinded after assignment to interventions (for example, participants, care providers, those	6

For peer review only

For peer review only - http://bmjopen.bmj.com/site/about/guidelines.xhtml

		assessing outcomes) and how	
	11b	If relevant, description of the similarity of interventions	NA
Statistical methods	12a	Statistical methods used to compare groups for primary and secondary outcomes	9
	12b	Methods for additional analyses, such as subgroup analyses and adjusted analyses	9
**Results**			
Participant flow (a diagram is strongly recommended)	13a	For each group, the numbers of participants who were randomly assigned, received intended treatment, and were analysed for the primary outcome	10, Figure 1
	13b	For each group, losses and exclusions after randomisation, together with reasons	10, Fig 1
Recruitment	14a	Dates defining the periods of recruitment and follow-up	5-6
	14b	Why the trial ended or was stopped	10,21
Baseline data	15	A table showing baseline demographic and clinical characteristics for each group	Table 1 and 2, Supp Table 1
Numbers analysed	16	For each group, number of participants (denominator) included in each analysis and whether the analysis was by original assigned groups	Fig 1, Table 3
Outcomes and estimation	17a	For each primary and secondary outcome, results for each group, and the estimated effect size and its precision (such as 95% confidence interval)	Table 3,4,5
	17b	For binary outcomes, presentation of both absolute and relative effect sizes is recommended	Only relative effect
Ancillary analyses	18	Results of any other analyses performed, including subgroup analyses and adjusted analyses, distinguishing pre-specified from exploratory	Table 4, 5
Harms	19	All important harms or unintended effects in each group (for specific guidance see CONSORT for harms)	No harms reported
**Discussion**			
Limitations	20	Trial limitations, addressing sources of potential bias, imprecision, and, if relevant, multiplicity of analyses	21-22
Generalisability	21	Generalisability (external validity, applicability) of the trial findings	NA, no effect
Interpretation	22	Interpretation consistent with results, balancing benefits and harms, and considering other relevant evidence	21
**Other information**			
Registration	23	Registration number and name of trial registry	2
Protocol	24	Where the full trial protocol can be accessed, if available	Published paper referenced

For peer review only

Funding	25	Sources of funding and other support (such as supply of drugs), role of funders	23

*We strongly recommend reading this statement in conjunction with the CONSORT 2010 Explanation and Elaboration for important clarifications on all the items. If relevant, we also recommend reading CONSORT extensions for cluster randomised trials, non-inferiority and equivalence trials, non-pharmacological treatments, herbal interventions, and pragmatic trials. Additional extensions are forthcoming: for those and for up to date references relevant to this checklist, see www.consort-statement.org.

For peer review only
